# Supplementary material for: The Mediating Roles of Lung Function Traits and Inflammatory Factors on the Associations between Measures of Obesity and Risk of Lower Respiratory Tract Infections: A Mendelian Randomization Study
Source: Healthcare (Basel). 2024 Sep 20;12(18):1882. doi: 10.3390/healthcare12181882 (PMC11431809; doi:10.3390/healthcare12181882)
Supplement: Supplementary file 1 [file healthcare-12-01882-s001.zip › healthcare-3144023-supplementary.pdf]

Supplementary Materials

Method S1. The calculation of the *F* statistic.
Method S2. Two-sample Mendelian randomization inverse-variance weighted.
Figure S1. Associations between obesity-related traits and potential mediators estimated using the inverse-variance weighted two-sample Mendelian randomization.
Figure S2. Associations between potential mediators and LRTIs estimated using the inverse-variance weighted two-sample Mendelian randomization.
Table S1. ICD-10 codes for lower respiratory tract infections in this study.
Table S2. Characteristics of the genetic instrument variables for the obesity-related traits at the genome-wide significance level.
Table S3. Characteristics of the genetic instrument variables for the potential mediators at the genome-wide significance level.
Table S4. Effects of obesity-related traits on lower respiratory tract infections estimated using different methods.
Table S5. Results of sensitivity analyses for the effects of obesity-related traits on lower respiratory tract infections.
Table S6. Effects of obesity-related traits on potential mediators estimated using different methods.
Table S7. Results of sensitivity analyses for the effects of obesity-related traits on potential mediators.
Table S8. Effects of potential mediators on lower respiratory tract infections estimated using different methods.
Table S9. Results of sensitivity analyses for the effects of potential mediators on lower respiratory tract infections.
Table S10. Results of mediation analyses.

Method S1. The calculation of the *F* Statistic

The *F* was calculated using the following formula (1):

$$F = \frac{R^2 \times (N-2)}{(1-R^2)}$$

where R^2 is the proportion of variance in the exposure that each single nucleotide polymorphism (SNP) explains.

$$R^2 = \frac{2 \times \beta^2 \times EAF \times (1-EAF)}{2 \times \beta^2 \times EAF \times (1-EAF) + 2 \times SE^2 \times N \times EAF \times (1-EAF)}$$

β represents the effect estimate of the genetic variant in the exposure genome-wide association studies (GWAS); EAF denotes the effect allele frequency; SE indicates standard error; *N* represents the number of individuals in the exposure GWAS.

Method S2. Two-sample Mendelian randomization inverse-variance weighted

We conducted two-sample Mendelian randomization on obesity-related traits and lower respiratory tract infections (LRTIs), obesity-related traits and potential mediators, and potential mediators and LRTIs, respectively. We adopted the inverse-variance weighted (IVW) as the primary method. The IVW estimate of the effect of an obesity-related trait on a type of LRTIs can be expressed as (2):

$$\hat{\beta}_{ivw} = \frac{\Sigma_k X_k Y_k \sigma_{Yk}{}^{-2}}{\Sigma_k X_k^2 \sigma_{Yk}{}^{-2}}$$

where k is the number of SNPs; X_k represents the change in an obesity-related trait (in standard deviation [SD] unit) per additional copy of the effect allele for the kth SNP; Y_k represents the change log-odds of a type of LRTIs per additional copy of the effect allele for the kth SNP; σ_{Yk} is the standard error of the association between the kth SNP and a type of LRTIs; $\hat{\beta}_{ivw}$ means the change in the log-odds of a type of LRTIs associated with per SD increase in an obesity-related trait. The IVW estimates of the effect of an obesity-related trait on a potential mediator and a potential mediator on a type of LRTIs were obtained in a similar way.

In addition, MR-Egger, weighted median, and weighted mode methods were employed as sensitivity analyses. The MR-Egger method provides a consistent effect estimate under a weaker Instrument Strength Independent of Direct Effect assumption (3). The weighted median method provides a reliable effect estimate, assuming that over 50% of the IVs are valid. The mode-based estimator relies on the assumption of zero modal pleiotropy (i.e., the group with the largest number of SNPs is also the group with no horizontal pleiotropy) to provide a consistent estimate (4).

References

1. Palmer TM, Lawlor DA, Harbord RM, Sheehan NA, Tobias JH, Timpson NJ, Davey Smith G, Sterne JA. Using multiple genetic variants as instrumental variables for modifiable risk factors. *Stat Methods Med Res* 2012 21 223-242. (https://doi.org/1010.1177/0962280210394459)
2. Bowden J, Davey Smith G, Haycock PC, Burgess S. Consistent Estimation in Mendelian Randomization with Some Invalid Instruments Using a Weighted Median Estimator. *Genet Epidemiol* 2016 40 304-314. (https://doi.org/10.1002/gepi.21965)
3. Burgess S, Thompson SG. Interpreting findings from Mendelian randomization using the MR-Egger method. *Eur J Epidemiol* 2017 32 377-389. (https://doi.org/10.1007/s10654-017-0255-x)
4. Hartwig FP, Davey Smith G, Bowden J. Robust inference in summary data Mendelian randomization via the zero modal pleiotropy assumption. *Int J Epidemiol* 2017 46 1985-1998. (https://doi.org/10.1093/ije/dyx102)

Obesity-related traits	Potential mediators	No. of SNPs	Change in potential mediator (95% CI)[a]
	FEV1	76	-0.10 (-0.14, -0.05)
	FVC	77	-0.16 (-0.21, -0.11)
	CRP	453	0.37 (0.34, 0.40)
	GlycA	78	0.17 (0.12, 0.22)
	IL-1Ra	373	0.17 (0.10, 0.25)
	IL-6	385	0.19 (0.11, 0.27)
	IL-8	398	0.05 (-0.03, 0.12)
BMI	IL-18	390	-0.01 (-0.08, 0.06)
	IL-27	389	0.02 (-0.05, 0.09)
	Basophil count	73	-0.01 (-0.03, 0.02)
	Eosinophil count	72	0.00 (-0.03, 0.04)
	Lymphocyte count	70	-0.03 (-0.07, 0.01)
	Monocyte count	73	-0.06 (-0.11, -0.02)
	Neutrophil count	72	0.00 (-0.04, 0.04)
	White blood cell count	70	-0.01 (-0.05, 0.03)
	FEV1	41	-0.13 (-0.20, -0.07)
	FVC	40	-0.20 (-0.26, -0.14)
	CRP	153	0.36 (0.30, 0.42)
	GlycA	41	0.20 (0.13, 0.26)
	IL-1Ra	302	0.25 (0.16, 0.33)
	IL-6	301	0.28 (0.18, 0.38)
	IL-8	323	0.09 (0.00, 0.18)
WC	IL-18	300	0.06 (-0.03, 0.15)
	IL-27	302	-0.07 (-0.16, 0.01)
	Basophil count	35	-0.03 (-0.07, 0.02)
	Eosinophil count	35	-0.02 (-0.08, 0.04)
	Lymphocyte count	33	-0.03 (-0.10, 0.04)
	Monocyte count	35	-0.04 (-0.10, 0.03)
	Neutrophil count	35	-0.02 (-0.06, 0.03)
	White blood cell count	35	-0.02 (-0.08, 0.03)
	FEV1	29	-0.07 (-0.14, 0.00)
	FVC	30	-0.19 (-0.26, -0.13)
	CRP	245	0.24 (0.19, 0.29)
	GlycA	29	0.17 (0.04, 0.29)
	IL-1Ra	250	0.14 (0.05, 0.23)
	IL-6	283	0.12 (0.02, 0.22)
	IL-8	271	0.08 (-0.02, 0.17)
WHR	IL-18	276	0.09 (0.01, 0.18)
	IL-27	259	-0.04 (-0.13, 0.04)
	Basophil count	26	0.02 (-0.04, 0.08)
	Eosinophil count	25	0.07 (0.00, 0.15)
	Lymphocyte count	25	0.07 (-0.05, 0.19)
	Monocyte count	26	0.00 (-0.09, 0.10)
	Neutrophil count	26	-0.01 (-0.12, 0.10)
	White blood cell count	26	0.05 (-0.07, 0.18)

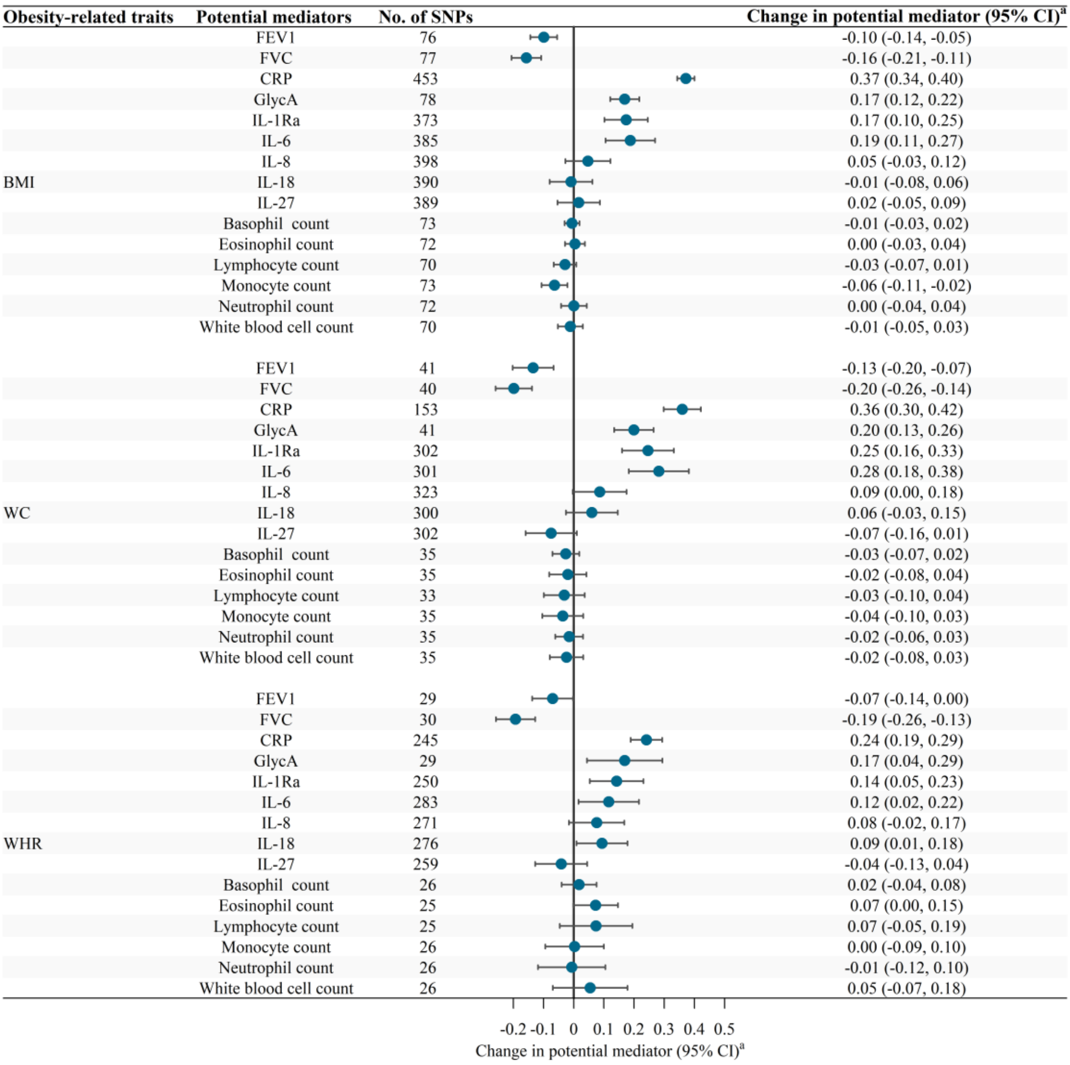

Figure S1. Associations between obesity-related traits and potential mediators estimated using the inverse-variance weighted two-sample Mendelian randomization. The number of SNPs varies within the same obesity-related trait, since the genetic associations with each obesity-related trait were obtained separately from two genome-wide association studies and some SNPs were removed through Steiger filtering. Points are point estimates of the effect of obesity-related traits on potential mediators and lines represent the 95% confidence intervals. [a] Change in a potential mediator (in standard deviation (SD) unit, except for CRP, whose unit was one-unit natural-log-transformed CRP) associated with per SD increase in an obesity-related trait. Abbreviations: BMI, body mass index; WC, waist circumference; WHR, waist-to-hip ratio; FEV1, forced expiratory volume in the first second; FVC, forced vital capacity; CRP, C-reactive protein; GlycA, Glycoprotein acetyls; IL-1Ra, interleukin-1-receptor antagonist; IL-6, interleukin-6; IL-8, interleukin-8; IL-18, interleukin-18; IL-27, interleukin-27.

Potential mediators	LRTIs	No. of SNPs	OR (95% CI)
	Acute bronchitis	253	0.80 (0.73, 0.88)
	Acute bronchiolitis	253	0.69 (0.56, 0.87)
FEV1	Bronchiectasis	253	1.13 (0.91, 1.39)
	Influenza	253	0.97 (0.87, 1.08)
	Pneumonia	253	0.93 (0.88, 0.98)
	Acute bronchitis	229	0.87 (0.79, 0.95)
	Acute bronchiolitis	229	0.83 (0.65, 1.05)
FVC	Bronchiectasis	229	0.90 (0.72, 1.12)
	Influenza	229	0.91 (0.81, 1.03)
	Pneumonia	229	0.94 (0.88, 1.00)
	Acute bronchitis	54	1.14 (1.08, 1.21)
	Acute bronchiolitis	54	1.03 (0.88, 1.20)
CRP	Bronchiectasis	54	1.06 (0.91, 1.23)
	Influenza	54	1.20 (1.09, 1.33)
	Pneumonia	54	1.17 (1.11, 1.24)
	Acute bronchitis	53	1.03 (0.96, 1.11)
	Acute bronchiolitis	53	0.99 (0.80, 1.23)
GlycA	Bronchiectasis	53	0.85 (0.66, 1.10)
	Influenza	53	1.02 (0.92, 1.12)
	Pneumonia	53	1.04 (0.98, 1.09)
	Acute bronchitis	23	1.05 (0.99, 1.11)
	Acute bronchiolitis	23	0.99 (0.85, 1.15)
IL-1Ra	Bronchiectasis	23	1.13 (0.98, 1.31)
	Influenza	23	1.02 (0.94, 1.10)
	Pneumonia	23	0.99 (0.95, 1.03)
	Acute bronchitis	12	1.06 (0.98, 1.15)
	Acute bronchiolitis	12	1.07 (0.85, 1.33)
IL-6	Bronchiectasis	12	0.88 (0.71, 1.08)
	Influenza	12	0.98 (0.88, 1.09)
	Pneumonia	12	1.08 (1.03, 1.13)
	Acute bronchitis	14	0.99 (0.86, 1.15)
	Acute bronchiolitis	14	1.14 (0.85, 1.54)
IL-8	Bronchiectasis	14	0.83 (0.62, 1.09)
	Influenza	14	1.00 (0.86, 1.15)
	Pneumonia	14	0.98 (0.92, 1.04)
	Acute bronchitis	24	1.04 (0.99, 1.09)
	Acute bronchiolitis	24	1.03 (0.89, 1.18)
IL-18	Bronchiectasis	24	1.04 (0.91, 1.19)
	Influenza	24	0.94 (0.88, 1.01)
	Pneumonia	24	0.99 (0.96, 1.02)
	Acute bronchitis	21	0.99 (0.94, 1.05)
	Acute bronchiolitis	21	0.99 (0.85, 1.16)
IL-27	Bronchiectasis	21	1.05 (0.91, 1.21)
	Influenza	21	0.90 (0.83, 0.97)
	Pneumonia	21	0.97 (0.93, 1.01)
	Acute bronchitis	191	0.96 (0.89, 1.04)
	Acute bronchiolitis	191	1.09 (0.88, 1.35)
Basophil count	Bronchiectasis	191	1.15 (0.94, 1.40)
	Influenza	191	1.01 (0.91, 1.12)
	Pneumonia	191	1.06 (1.01, 1.11)
	Acute bronchitis	429	1.11 (1.06, 1.17)
	Acute bronchiolitis	429	1.20 (1.05, 1.38)
Eosinophil count	Bronchiectasis	429	1.19 (1.03, 1.36)
	Influenza	429	1.04 (0.97, 1.11)
	Pneumonia	429	1.06 (1.03, 1.10)
	Acute bronchitis	476	1.07 (1.02, 1.13)
	Acute bronchiolitis	476	0.95 (0.81, 1.11)
Lymphocyte count	Bronchiectasis	476	1.04 (0.91, 1.19)
	Influenza	476	1.05 (0.98, 1.12)
	Pneumonia	476	1.06 (1.02, 1.10)
	Acute bronchitis	479	1.00 (0.96, 1.04)
	Acute bronchiolitis	479	1.02 (0.91, 1.15)
Monocyte count	Bronchiectasis	479	1.03 (0.93, 1.15)
	Influenza	479	1.03 (0.97, 1.09)
	Pneumonia	479	1.04 (1.01, 1.06)
	Acute bronchitis	400	1.04 (0.97, 1.10)
	Acute bronchiolitis	400	1.15 (0.98, 1.34)
Neutrophil count	Bronchiectasis	400	1.05 (0.91, 1.22)
	Influenza	400	1.06 (0.98, 1.14)
	Pneumonia	400	1.04 (1.00, 1.08)
	Acute bronchitis	466	1.07 (1.01, 1.13)
	Acute bronchiolitis	466	1.13 (0.98, 1.31)
White blood cell count	Bronchiectasis	466	1.07 (0.93, 1.23)
	Influenza	466	1.07 (0.99, 1.15)
	Pneumonia	466	1.07 (1.03, 1.10)

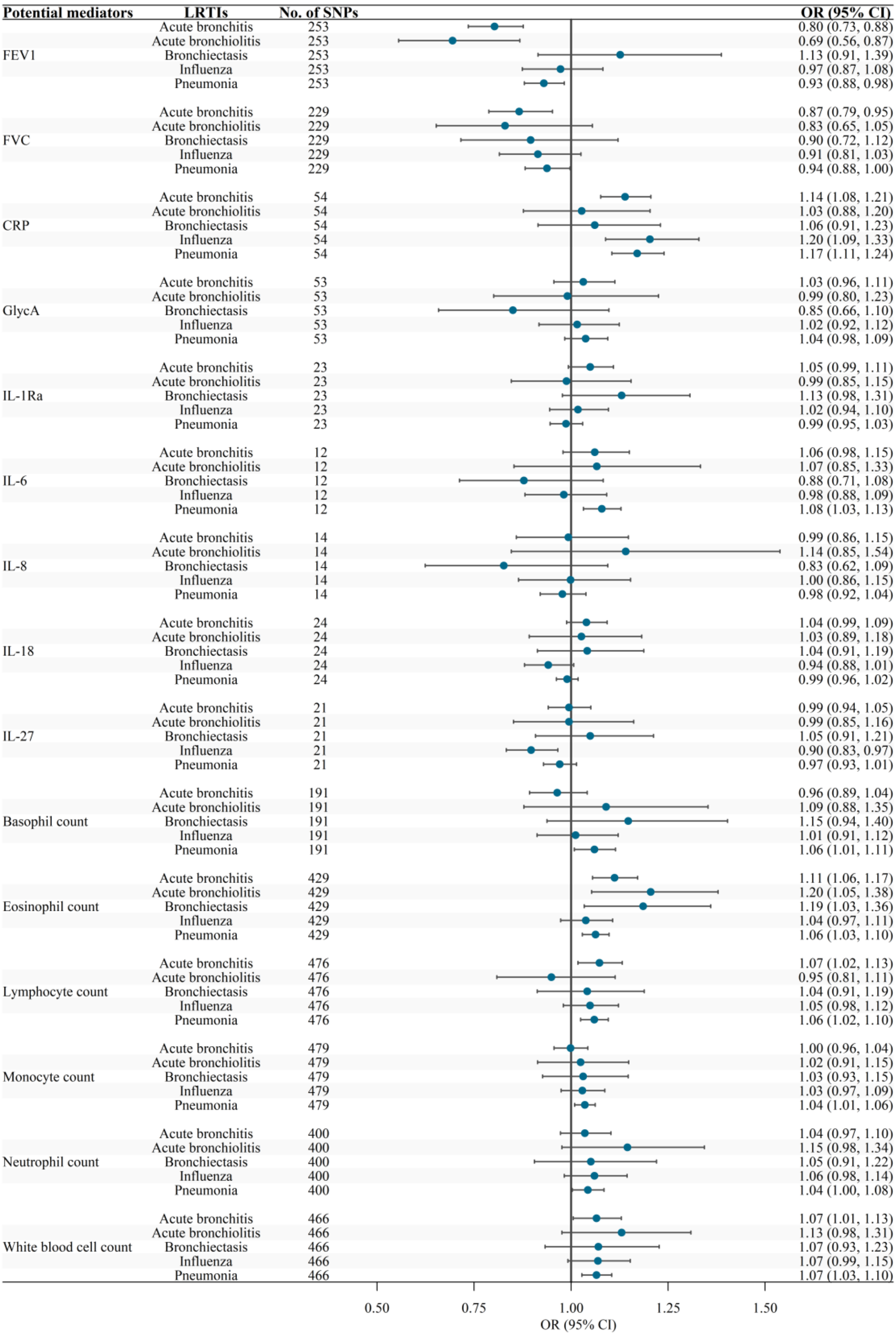

Figure S2. Associations between potential mediators and LRTIs estimated using the inverse-variance weighted two-sample Mendelian randomization. Points are point estimates of the effect of potential mediators on LRTIs and lines represent the 95% confidence intervals. OR represents the odds ratio of the occurrence of a type of LRTIs associated with per standard deviation increase in a potential mediator (except for CRP whose unit was one-unit natural-log-transformed CRP). Abbreviations: LRTIs, lower respiratory tract infections; FEV1, forced expiratory volume in the first second; FVC, forced vital capacity; CRP, C-reactive protein; GlycA, Glycoprotein acetyls; IL-1Ra, interleukin-1-receptor antagonist; IL-6, interleukin-6; IL-8, interleukin-8; IL-18, interleukin-18; IL-27, interleukin-27.

Table S1. ICD-10 codes for lower respiratory tract infections in this study.

LRTIs	ICD codes
Acute bronchitis	ICD-9: 4660; ICD-10: J20
Acute bronchiolitis	ICD-9: 4661; ICD-10: J21
Bronchiectasis	ICD-8: 51899; ICD-9: 494; ICD-10: J47
Influenza	ICD-8: 47 [0-4]; ICD-9: 487; ICD-10: J9, J10, J11
Pneumonia	ICD-8: 47101, 47109, 48099, 48199, 48210, 482, 48399, 4850 [2-9], 486; ICD-9: 480, 4870A, 481, 482, 4822, 483, 485; ICD-10: J10.0, J11.0, J12, B01.2, B06.8, B25.0, J13, J14, J15, J17.0*, J17.0*A01.0, J17.0*A02.2, J17.0*A21.2, J17.0*A22.1, J17.0*A37.9, J17.0*A42.0, J17.0*A43.0, J17.0*A54.8, J16, J17.1, J17 [2-8], J18

* Pneumonia in diseases classified elsewhere in ICD-10.

Abbreviations: LRTIs, lower respiratory tract infections; ICD, International Statistical Classification of Diseases and Related Health Problems.

Table S2. Characteristics of the genetic instrument variables for the obesity-related traits at the genome-wide significance level.

Obesity-related traits (data sources)	SNPs	Effect allele	Other allele	*β*	SE	*P*	EAF	*F*
BMI (GIANT and UKB[a])	rs2803316	A	G	-0.014	0.002	1.88E-16	0.455	69.771
	rs7535528	A	G	-0.015	0.002	1.58E-17	0.372	73.197
	rs6577584	T	G	-0.012	0.002	3.37E-11	0.659	42.975
	rs2791653	A	G	0.013	0.002	9.99E-13	0.240	49.000
	rs9435739	A	G	-0.011	0.002	9.90E-10	0.661	36.670
	rs4655141	T	C	-0.017	0.002	6.20E-15	0.836	61.837
	rs10798888	T	G	0.013	0.002	5.81E-09	0.189	34.917
	rs12022461	A	G	-0.017	0.002	9.78E-14	0.167	57.622
	rs7512146	T	G	-0.010	0.002	1.29E-08	0.527	32.557
	rs11586036	A	C	-0.020	0.004	1.06E-08	0.935	32.003
	rs11206436	T	C	0.015	0.003	4.67E-08	0.893	29.642
	rs1707322	A	G	-0.014	0.002	1.59E-14	0.316	57.086
	rs2984618	T	G	0.017	0.002	3.80E-24	0.441	106.347
	rs657452	A	G	0.019	0.002	3.17E-30	0.404	138.062
	rs17425707	T	C	-0.017	0.003	6.16E-10	0.900	38.175
	rs2481665	T	C	0.016	0.002	3.24E-23	0.582	101.254
	rs2503185	A	G	0.013	0.002	1.33E-14	0.510	58.477
	rs3101336	T	C	-0.025	0.002	4.80E-54	0.383	252.015
	rs12036473	A	T	-0.017	0.002	2.80E-24	0.618	101.180
	rs12049202	T	C	0.024	0.002	3.35E-29	0.188	129.526
	rs2166171	T	C	-0.010	0.002	5.49E-09	0.624	32.744
	rs12034762	T	C	0.013	0.002	6.34E-13	0.391	54.066
	rs11165643	T	C	0.019	0.002	4.49E-30	0.580	133.691
	rs10747488	A	C	-0.013	0.002	1.49E-11	0.780	44.890
	rs17024393	T	C	-0.064	0.005	7.11E-39	0.969	172.734
	rs197374	T	C	0.014	0.002	3.16E-16	0.403	68.792
	rs12120851	T	C	-0.012	0.002	2.87E-08	0.631	30.512
	rs7534091	A	G	-0.012	0.002	6.65E-11	0.718	44.444
	rs6587552	A	G	0.016	0.002	2.17E-15	0.241	61.622
	rs3856261	A	G	0.012	0.002	1.12E-11	0.498	46.560
	rs1750307	A	T	0.013	0.002	2.78E-13	0.366	51.361
	rs10733051	A	G	0.009	0.002	6.96E-09	0.512	33.785
	rs12564992	A	G	-0.019	0.003	2.57E-13	0.888	52.842
	rs543874	A	G	-0.048	0.002	3.06E-125	0.779	573.601
	rs10920678	A	G	0.015	0.002	7.15E-20	0.425	86.722
	rs2400414	T	C	-0.013	0.002	5.52E-13	0.346	49.000
	rs2820295	A	G	0.024	0.002	5.56E-39	0.327	170.447
	rs10920336	A	G	-0.010	0.002	4.20E-09	0.533	35.297

rs1006317	T	G	0.016	0.003	3.75E-10	0.127	37.870
rs6661316	T	C	0.012	0.002	1.72E-13	0.589	56.250
rs10489615	A	G	0.010	0.002	1.33E-08	0.400	33.913
rs946824	T	C	0.020	0.003	4.82E-15	0.136	62.094
rs4577313	A	G	0.016	0.002	2.63E-15	0.241	62.410
rs13021737	A	G	-0.058	0.002	2.89E-161	0.157	757.558
rs10929925	A	C	-0.014	0.002	3.05E-18	0.411	78.765
rs11902450	T	C	0.017	0.003	1.06E-09	0.105	38.256
rs10182181	A	G	-0.033	0.002	2.45E-91	0.505	417.690
rs1260326	T	C	-0.011	0.002	1.16E-10	0.391	39.616
rs17327461	T	C	0.012	0.002	7.65E-14	0.446	58.140
rs10185199	A	G	-0.014	0.002	3.18E-13	0.286	51.122
rs10169594	T	C	-0.012	0.002	1.34E-11	0.648	44.444
rs7561278	T	C	0.017	0.002	4.89E-16	0.767	64.764
rs930295	A	C	0.021	0.002	2.03E-19	0.155	81.784
rs805412	A	G	-0.010	0.002	1.14E-08	0.434	33.232
rs4671328	T	G	0.021	0.002	3.42E-36	0.450	158.463
rs6545714	A	G	-0.019	0.002	4.01E-32	0.611	147.015
rs2861683	A	C	0.014	0.002	1.85E-16	0.597	68.792
rs17020497	A	G	0.014	0.003	2.04E-08	0.134	31.360
rs12714199	T	C	-0.014	0.002	3.22E-16	0.615	68.792
rs4303732	T	C	0.017	0.002	1.26E-22	0.614	98.827
rs264941	A	C	-0.012	0.002	1.84E-13	0.468	53.204
rs10197031	T	C	-0.016	0.002	5.05E-18	0.726	71.803
rs11884795	A	G	0.011	0.002	1.57E-09	0.268	38.716
rs4988235	A	G	0.012	0.002	7.09E-13	0.721	53.204
rs17814208	A	G	-0.013	0.002	7.24E-11	0.758	41.602
rs16824165	A	G	0.016	0.003	2.18E-09	0.116	35.117
rs429343	A	G	0.015	0.002	8.44E-18	0.434	73.758
rs2119753	A	G	0.010	0.002	1.00E-08	0.623	34.602
rs3764835	A	G	-0.013	0.002	3.84E-08	0.151	29.340
rs12692596	T	C	0.012	0.002	1.03E-12	0.362	49.827
rs1521527	C	G	-0.010	0.002	1.77E-09	0.518	36.000
rs3754963	A	T	0.013	0.002	2.43E-11	0.763	46.097
rs2138348	T	G	-0.013	0.002	1.35E-12	0.284	49.739
rs2119137	A	G	-0.013	0.002	2.33E-13	0.662	52.966
rs7588437	A	G	-0.017	0.002	2.32E-22	0.366	94.204
rs6757852	A	G	0.011	0.002	3.76E-10	0.499	38.879
rs7593917	A	G	-0.012	0.002	9.67E-13	0.454	51.660
rs4482463	A	C	-0.031	0.003	4.85E-23	0.917	100.000
rs6715020	T	C	-0.011	0.002	1.28E-09	0.381	38.149

rs11692326	T	C	0.015	0.002	1.69E-14	0.230	59.859
rs7599312	A	G	-0.018	0.002	1.52E-23	0.274	102.234
rs12987009	A	T	-0.011	0.002	1.83E-10	0.585	41.111
rs6725931	T	C	0.019	0.002	2.30E-15	0.848	60.710
rs4973618	A	G	-0.015	0.002	1.25E-16	0.662	67.605
rs6720868	T	C	0.015	0.002	1.82E-17	0.308	73.197
rs7568228	C	G	-0.010	0.002	1.00E-09	0.540	37.426
rs17535749	A	G	0.016	0.003	3.46E-09	0.108	34.679
rs10510419	T	G	-0.017	0.002	2.23E-13	0.147	53.353
rs2600226	T	C	-0.012	0.002	1.42E-10	0.671	41.531
rs1048637	T	G	-0.009	0.002	2.94E-08	0.547	30.574
rs4858193	T	C	0.013	0.002	2.44E-12	0.720	49.000
rs6804842	A	G	-0.014	0.002	7.57E-18	0.430	77.660
rs11921432	T	C	-0.019	0.003	5.38E-12	0.891	49.000
rs1799923	A	G	-0.022	0.003	1.12E-17	0.112	74.225
rs28350	A	G	0.017	0.002	1.07E-14	0.172	61.124
rs4017425	T	C	-0.012	0.002	2.91E-12	0.468	48.180
rs11713193	A	G	0.025	0.002	3.02E-48	0.516	209.397
rs2365389	T	C	-0.017	0.002	6.49E-25	0.400	110.250
rs925018	C	G	-0.013	0.002	4.33E-14	0.667	58.477
rs11915371	A	C	-0.015	0.002	2.29E-13	0.802	53.778
rs12636480	T	G	0.013	0.002	6.96E-13	0.351	50.568
rs9827823	T	C	0.018	0.002	3.39E-15	0.855	62.616
rs9818122	T	C	-0.023	0.002	3.97E-30	0.793	129.960
rs11128021	A	G	-0.018	0.002	1.63E-14	0.162	56.877
rs1492014	T	C	-0.017	0.002	1.44E-23	0.567	101.180
rs1436344	C	G	0.015	0.002	1.07E-17	0.570	74.771
rs7640424	T	C	-0.014	0.002	1.23E-14	0.311	56.250
rs17681451	A	G	-0.023	0.003	7.37E-13	0.076	52.679
rs6808814	T	C	0.012	0.002	1.31E-09	0.734	38.571
rs7631156	A	G	0.022	0.002	3.33E-32	0.309	142.669
rs687339	T	C	0.019	0.002	4.32E-22	0.770	97.906
rs16851483	T	G	0.035	0.003	4.87E-25	0.072	107.183
rs355777	C	G	0.015	0.002	2.13E-18	0.398	78.896
rs9826775	A	G	0.016	0.002	6.61E-11	0.851	41.710
rs2047648	A	T	-0.013	0.002	7.95E-12	0.747	44.222
rs3732927	T	C	0.010	0.002	1.13E-08	0.291	33.913
rs12635553	A	T	0.010	0.002	4.10E-09	0.486	33.913
rs39654	A	G	-0.016	0.002	1.75E-21	0.451	91.934
rs6443750	T	C	-0.015	0.002	7.25E-13	0.199	52.390
rs865809	A	G	0.012	0.002	6.50E-10	0.225	38.440

rs9816226	A	T	-0.032	0.002	1.45E-50	0.176	224.999
rs7616009	A	G	-0.016	0.002	4.33E-11	0.160	42.793
rs2051559	T	C	-0.017	0.003	3.78E-11	0.859	44.622
rs6818414	T	C	-0.010	0.002	4.57E-09	0.516	34.602
rs1477890	A	G	-0.013	0.002	6.12E-14	0.488	56.692
rs9291467	T	C	0.014	0.002	4.62E-17	0.465	69.771
rs6448587	A	C	0.016	0.002	1.65E-12	0.806	50.281
rs337637	A	G	-0.014	0.002	6.24E-16	0.359	64.944
rs10938397	A	G	-0.032	0.002	2.42E-86	0.570	405.015
rs2271046	A	T	-0.012	0.002	3.03E-10	0.695	40.818
rs1492767	T	C	0.010	0.002	3.55E-09	0.468	35.254
rs2192158	A	G	0.014	0.002	7.42E-16	0.449	64.944
rs1346841	A	G	-0.013	0.002	3.18E-13	0.412	54.934
rs10002111	A	G	0.013	0.002	1.26E-09	0.223	35.431
rs10033843	A	G	0.014	0.002	1.07E-11	0.215	44.444
rs17507682	T	G	0.014	0.002	1.91E-08	0.154	31.641
rs4148155	A	G	0.019	0.003	1.34E-13	0.887	54.532
rs4286488	A	G	0.012	0.002	1.70E-09	0.761	36.602
rs7678054	A	G	-0.010	0.002	4.57E-09	0.479	33.913
rs3796432	T	G	-0.011	0.002	2.21E-10	0.367	39.410
rs13107325	T	C	0.047	0.003	3.81E-47	0.082	213.890
rs326893	T	C	0.012	0.002	1.89E-12	0.581	50.661
rs7696649	A	G	0.012	0.002	4.73E-10	0.278	37.274
rs4864201	T	C	0.014	0.002	4.30E-16	0.347	64.944
rs1296328	A	C	0.017	0.002	3.49E-22	0.446	95.349
rs17367750	T	C	-0.012	0.002	1.97E-11	0.312	45.938
rs9992189	C	G	-0.010	0.002	2.04E-08	0.608	32.557
rs3914628	T	C	0.017	0.002	6.91E-13	0.859	51.465
rs750090	T	C	0.011	0.002	2.50E-10	0.626	39.410
rs13110266	A	G	-0.012	0.002	3.96E-14	0.404	60.062
rs7685628	A	T	0.010	0.002	6.15E-09	0.401	35.297
rs1522569	T	G	0.014	0.002	1.60E-10	0.818	41.076
rs1437842	A	G	-0.011	0.002	8.49E-10	0.491	38.879
rs4565118	A	C	-0.011	0.002	7.28E-09	0.502	34.679
rs698147	A	G	0.012	0.002	9.67E-12	0.447	46.560
rs6890310	A	G	-0.012	0.002	3.29E-10	0.292	39.227
rs7730004	T	C	0.014	0.002	1.46E-14	0.661	59.633
rs7704281	A	G	0.025	0.004	1.17E-09	0.046	36.588
rs13186194	T	C	0.010	0.002	2.20E-09	0.617	34.602
rs4700608	T	C	-0.016	0.002	4.32E-20	0.524	83.131
rs6886740	T	C	0.011	0.002	3.06E-10	0.662	40.111

rs1159692	A	C	0.014	0.002	5.54E-15	0.475	63.062
rs2112347	T	G	0.028	0.002	1.17E-61	0.630	263.584
rs10942267	A	G	0.015	0.002	6.53E-16	0.693	67.605
rs2962334	T	G	0.040	0.006	1.78E-11	0.027	45.049
rs1501673	A	G	0.029	0.003	2.73E-31	0.136	133.633
rs12652212	A	G	-0.013	0.002	1.63E-15	0.561	67.035
rs7713317	A	G	-0.017	0.002	1.96E-20	0.720	85.049
rs6882366	T	C	-0.013	0.002	4.39E-14	0.396	59.380
rs11739877	T	C	0.012	0.002	4.01E-11	0.623	41.531
rs6888194	T	C	-0.013	0.002	3.58E-08	0.844	30.490
rs40067	A	G	-0.025	0.002	9.73E-29	0.170	120.045
rs459552	A	T	-0.013	0.002	8.36E-12	0.775	49.000
rs6893539	A	C	-0.012	0.002	6.17E-11	0.698	41.230
rs6864049	A	G	-0.012	0.002	1.48E-13	0.489	57.191
rs6886072	T	C	-0.010	0.002	4.13E-09	0.467	34.602
rs13174863	A	G	-0.020	0.002	1.94E-17	0.854	73.363
rs17405603	A	T	-0.013	0.002	4.43E-11	0.706	43.282
rs2973157	A	G	-0.011	0.002	4.24E-08	0.671	28.444
rs7715256	T	G	-0.016	0.002	3.98E-22	0.564	97.515
rs7734385	A	G	-0.010	0.002	6.08E-10	0.445	39.848
rs2861089	A	T	0.011	0.002	1.55E-09	0.381	38.149
rs7727781	T	C	0.009	0.002	4.27E-08	0.516	29.927
rs2053682	A	C	0.017	0.002	2.59E-20	0.678	89.197
rs6556301	T	G	-0.011	0.002	8.14E-11	0.371	44.183
rs2228213	A	G	-0.014	0.002	5.50E-17	0.337	71.751
rs11757278	T	C	0.013	0.002	6.92E-13	0.696	49.000
rs3806114	A	G	-0.012	0.002	1.46E-11	0.692	44.444
rs2066295	A	G	0.014	0.002	2.29E-12	0.758	50.410
rs3115667	T	C	-0.018	0.002	5.92E-21	0.282	88.756
rs2281819	A	T	-0.015	0.002	2.09E-14	0.229	59.290
rs2744974	T	C	0.026	0.002	1.28E-51	0.324	235.712
rs2436728	A	G	0.019	0.002	1.97E-29	0.407	123.602
rs1358980	T	C	-0.013	0.002	5.15E-15	0.474	57.581
rs2206277	T	C	0.041	0.002	1.82E-83	0.156	377.468
rs1327259	A	G	0.016	0.002	1.47E-19	0.610	85.290
rs6915002	T	C	0.010	0.002	8.71E-09	0.411	33.913
rs9370410	A	G	0.011	0.002	2.58E-08	0.729	30.540
rs2622274	T	G	-0.011	0.002	3.23E-10	0.455	39.616
rs13213867	A	G	0.012	0.002	1.03E-08	0.803	31.258
rs6921533	T	C	0.010	0.002	1.37E-08	0.293	33.383
rs9294260	A	G	0.014	0.002	8.16E-18	0.469	76.562

rs6909685	T	C	-0.015	0.002	2.78E-16	0.330	68.521
rs9320823	T	C	-0.017	0.002	2.07E-21	0.412	94.204
rs12530388	A	C	0.010	0.002	4.23E-09	0.474	34.602
rs156201	C	G	0.012	0.002	1.65E-10	0.746	42.593
rs768023	A	G	0.016	0.002	1.13E-22	0.603	101.254
rs2357760	A	G	0.014	0.002	2.11E-16	0.674	70.758
rs2875762	C	G	0.013	0.002	1.07E-10	0.252	41.602
rs1268065	A	G	-0.010	0.002	7.12E-10	0.500	39.062
rs9375702	T	C	-0.011	0.002	5.52E-09	0.679	34.679
rs2246012	T	C	-0.016	0.002	1.15E-13	0.851	53.556
rs6922607	A	G	-0.013	0.002	1.85E-09	0.808	34.917
rs765875	T	C	-0.013	0.002	1.08E-14	0.469	60.290
rs7760482	A	G	-0.010	0.002	6.25E-09	0.616	32.111
rs12527426	A	G	0.015	0.002	5.54E-16	0.300	62.327
rs9478496	T	C	-0.016	0.002	8.17E-12	0.845	46.595
rs13191362	A	G	0.024	0.003	4.08E-21	0.857	88.360
rs9458814	T	C	-0.011	0.002	2.33E-08	0.771	31.360
rs6461115	A	G	0.013	0.002	1.47E-12	0.762	49.739
rs6463489	T	C	0.017	0.003	2.50E-10	0.096	41.256
rs7779296	A	G	-0.010	0.002	4.59E-09	0.716	32.744
rs4307239	A	G	-0.012	0.002	1.47E-11	0.536	45.761
rs213518	T	C	-0.015	0.002	2.49E-10	0.850	40.641
rs215669	A	G	-0.015	0.002	8.94E-18	0.602	76.820
rs2108719	A	G	0.011	0.002	2.28E-08	0.727	31.715
rs217433	T	C	-0.012	0.002	3.00E-08	0.803	29.989
rs2289379	T	C	-0.014	0.002	7.02E-15	0.392	57.929
rs10499694	A	G	0.013	0.002	1.29E-15	0.494	66.015
rs11772246	T	C	0.015	0.002	3.69E-11	0.823	43.440
rs17207196	T	C	-0.022	0.002	1.58E-36	0.420	167.474
rs17149254	T	C	0.024	0.002	2.99E-25	0.205	107.077
rs6973656	A	G	-0.010	0.002	6.31E-09	0.584	35.297
rs1965529	A	G	0.016	0.002	7.66E-14	0.773	53.556
rs274628	A	C	-0.010	0.002	1.36E-08	0.337	32.111
rs2283006	A	G	0.013	0.002	8.06E-15	0.488	60.290
rs13240600	A	G	0.018	0.002	7.90E-16	0.834	66.942
rs1721447	T	G	-0.010	0.002	3.79E-09	0.510	34.602
rs2396625	A	T	-0.018	0.002	2.81E-24	0.418	107.183
rs13245051	A	G	0.015	0.002	1.12E-18	0.456	77.854
rs10261050	T	C	0.011	0.002	4.53E-11	0.475	44.183
rs2283093	T	C	0.012	0.002	1.10E-08	0.195	33.199
rs7802342	T	G	-0.012	0.002	6.23E-11	0.705	42.593

rs17160760	A	T	-0.017	0.003	1.27E-09	0.105	37.346
rs11773362	T	C	-0.011	0.002	6.39E-09	0.340	34.028
rs2907948	A	G	-0.015	0.002	1.95E-14	0.244	58.241
rs1700082	C	G	0.009	0.002	3.38E-08	0.659	30.574
rs4240673	T	C	0.018	0.002	1.58E-26	0.455	119.629
rs13263601	A	C	-0.015	0.002	4.87E-16	0.659	64.892
rs10110189	T	C	-0.016	0.003	2.20E-08	0.106	31.041
rs10101364	T	C	0.012	0.002	5.61E-11	0.680	44.444
rs1421334	A	C	0.014	0.002	3.11E-15	0.455	63.062
rs2466103	T	G	-0.012	0.002	8.00E-12	0.693	45.188
rs6468266	A	T	-0.011	0.002	7.71E-11	0.418	44.183
rs12681792	A	C	0.015	0.002	2.88E-12	0.202	51.020
rs12334877	A	G	-0.014	0.002	2.17E-11	0.191	47.020
rs1431659	A	G	0.019	0.002	2.33E-23	0.274	101.055
rs17405819	T	C	0.021	0.002	6.04E-33	0.685	137.410
rs2196618	A	G	-0.014	0.002	1.49E-12	0.263	51.992
rs2120710	A	G	0.010	0.002	1.98E-08	0.653	31.484
rs12680842	A	G	0.014	0.002	3.41E-16	0.684	69.771
rs6469351	T	C	0.011	0.002	3.72E-10	0.672	38.028
rs3808477	T	C	-0.018	0.002	8.73E-22	0.274	91.756
rs11781699	T	C	-0.014	0.002	1.56E-11	0.813	45.082
rs6470144	T	G	0.010	0.002	3.06E-08	0.650	29.642
rs7842934	T	C	-0.018	0.003	1.55E-08	0.921	32.970
rs305256	T	C	-0.012	0.002	2.79E-08	0.226	29.989
rs16906838	T	C	-0.025	0.004	4.31E-10	0.049	38.131
rs11782074	T	G	0.012	0.002	4.24E-12	0.368	47.457
rs10099330	A	G	-0.012	0.002	3.22E-12	0.538	49.000
rs10975933	C	G	0.011	0.002	2.07E-10	0.663	40.111
rs1948080	T	G	0.014	0.002	1.13E-14	0.630	57.086
rs10961649	T	C	0.010	0.002	1.51E-08	0.324	33.383
rs4740619	T	C	0.019	0.002	3.15E-31	0.544	139.535
rs10962550	C	G	0.019	0.002	5.68E-17	0.176	71.479
rs10811868	A	G	-0.010	0.002	3.18E-08	0.320	30.864
rs10968114	A	C	0.011	0.002	3.30E-11	0.533	44.183
rs1412235	C	G	0.024	0.002	2.28E-42	0.315	194.356
rs10121187	C	G	0.010	0.002	2.02E-09	0.509	36.000
rs2275003	A	G	0.011	0.002	9.44E-12	0.501	48.129
rs13296413	T	C	-0.015	0.002	2.49E-17	0.381	75.792
rs2134858	T	C	-0.012	0.002	5.87E-12	0.512	47.367
rs7861160	T	C	0.010	0.002	3.68E-08	0.594	31.228
rs1999433	T	C	-0.011	0.002	2.90E-10	0.440	39.616

rs2777768	A	G	0.012	0.002	6.38E-10	0.723	39.227
rs7357754	A	G	-0.012	0.002	1.81E-12	0.499	49.827
rs10992867	A	G	0.016	0.002	3.20E-17	0.269	72.698
rs7025938	C	G	-0.016	0.002	1.47E-19	0.680	81.000
rs7024334	T	G	0.014	0.002	4.71E-12	0.225	45.562
rs1928295	T	C	0.013	0.002	2.23E-16	0.571	70.140
rs1877875	T	C	-0.011	0.002	2.51E-10	0.434	41.111
rs10733682	A	G	0.015	0.002	1.76E-19	0.459	85.562
rs2267958	A	G	-0.013	0.002	2.01E-13	0.509	52.160
rs4740383	A	G	0.013	0.002	7.03E-14	0.419	52.966
rs10858334	C	G	-0.015	0.003	4.91E-09	0.848	35.046
rs7907470	A	G	-0.018	0.003	7.36E-09	0.916	32.600
rs7893571	T	G	0.013	0.002	5.83E-12	0.674	48.225
rs7084454	A	G	0.020	0.002	4.51E-27	0.309	121.000
rs10829164	T	C	0.015	0.002	2.37E-10	0.147	39.585
rs4097319	T	G	0.011	0.002	4.32E-10	0.564	39.616
rs12765914	T	C	0.023	0.003	1.96E-13	0.080	53.149
rs1624134	C	G	0.010	0.002	6.51E-09	0.393	34.602
rs12259464	A	G	0.011	0.002	1.48E-10	0.481	41.111
rs10761785	T	G	-0.013	0.002	3.47E-16	0.514	69.097
rs12098284	T	C	0.018	0.003	9.87E-13	0.121	50.083
rs11001259	A	T	-0.014	0.002	1.22E-08	0.177	33.062
rs7899106	A	G	-0.033	0.004	1.72E-18	0.950	78.107
rs10788494	C	G	0.013	0.002	5.51E-15	0.484	60.290
rs2439823	A	G	-0.017	0.002	6.51E-22	0.451	94.204
rs17094222	T	C	-0.017	0.002	4.04E-18	0.791	74.822
rs1886276	A	G	-0.011	0.002	2.95E-10	0.571	40.360
rs7903146	T	C	-0.018	0.002	1.67E-23	0.279	97.790
rs2257791	A	G	-0.014	0.002	1.63E-12	0.756	48.302
rs845084	A	G	0.014	0.002	3.22E-12	0.267	51.235
rs17636031	T	C	-0.015	0.002	3.87E-17	0.724	73.197
rs4880341	T	C	-0.013	0.002	3.06E-14	0.573	58.477
rs12416812	A	G	0.011	0.002	5.21E-11	0.507	43.891
rs4256980	C	G	-0.019	0.002	8.63E-29	0.331	121.000
rs900144	T	C	0.015	0.002	1.53E-18	0.578	75.792
rs2074314	T	C	0.011	0.002	1.37E-09	0.636	38.149
rs6265	T	C	-0.041	0.002	7.40E-89	0.184	386.777
rs570463	A	C	-0.012	0.002	4.33E-11	0.327	45.188
rs11030618	T	C	0.011	0.002	1.67E-10	0.579	41.868
rs2065418	T	G	0.014	0.002	6.07E-15	0.646	59.633
rs2862996	T	G	-0.022	0.002	3.60E-35	0.701	161.439

rs10838202	C	G	0.011	0.002	5.54E-11	0.556	44.183
rs10742752	T	C	-0.012	0.002	1.20E-13	0.376	52.349
rs7124681	A	C	0.026	0.002	3.96E-55	0.416	258.003
rs6591407	A	C	-0.012	0.002	3.58E-09	0.198	34.866
rs562664	T	C	-0.014	0.002	4.15E-10	0.187	39.347
rs7102454	T	C	-0.017	0.002	3.84E-21	0.644	87.111
rs592483	T	C	-0.014	0.002	1.53E-16	0.593	64.944
rs737185	A	G	0.014	0.002	4.23E-11	0.781	42.560
rs349088	A	C	-0.013	0.002	3.50E-14	0.478	58.477
rs10741329	A	G	0.012	0.002	3.91E-10	0.694	40.818
rs3019466	T	C	-0.013	0.002	4.91E-08	0.168	28.444
rs2605603	A	G	-0.010	0.002	2.04E-10	0.481	41.441
rs4937870	A	G	0.011	0.002	1.56E-09	0.694	34.748
rs10891549	T	C	0.010	0.002	1.93E-08	0.465	31.228
rs12286929	A	G	-0.018	0.002	1.93E-27	0.501	122.379
rs3825061	T	C	0.014	0.002	6.15E-16	0.386	67.820
rs6589936	A	C	0.011	0.002	2.18E-10	0.387	38.716
rs7944782	T	G	-0.014	0.002	3.61E-17	0.502	71.751
rs2007518	A	G	-0.013	0.002	1.96E-14	0.562	58.477
rs1941213	A	C	0.011	0.002	1.59E-08	0.711	32.310
rs329651	T	G	0.016	0.002	2.13E-14	0.806	58.050
rs12364470	T	G	-0.019	0.002	2.18E-17	0.854	72.250
rs11611246	T	G	0.022	0.002	2.04E-28	0.199	124.322
rs765125	T	C	-0.010	0.002	1.27E-08	0.586	33.232
rs10772983	T	C	-0.010	0.002	6.21E-10	0.539	39.062
rs11044430	A	T	0.016	0.002	3.77E-12	0.844	49.000
rs11046972	T	C	0.018	0.003	3.72E-08	0.071	30.250
rs10842240	C	G	0.020	0.003	1.21E-14	0.126	60.840
rs11170468	A	C	0.013	0.002	1.12E-11	0.783	46.814
rs1350430	T	C	-0.013	0.002	2.08E-13	0.533	54.066
rs7138803	A	G	0.030	0.002	3.10E-71	0.387	305.221
rs2271189	A	G	-0.014	0.002	9.26E-16	0.396	61.361
rs7975187	A	G	-0.014	0.002	3.86E-11	0.771	42.560
rs650198	T	C	-0.014	0.002	6.43E-13	0.729	51.992
rs11115176	T	C	0.013	0.002	6.79E-12	0.784	47.537
rs10506971	A	G	-0.014	0.002	4.69E-17	0.552	69.771
rs11105839	A	T	-0.011	0.002	1.25E-11	0.373	44.183
rs2712665	T	C	-0.011	0.002	5.40E-09	0.697	32.310
rs6539064	C	G	0.019	0.002	1.14E-23	0.747	104.255
rs1860561	A	G	0.016	0.002	1.71E-16	0.209	70.030
rs11066188	A	G	-0.011	0.002	3.05E-12	0.376	50.765

rs2707183	T	G	-0.009	0.002	4.89E-08	0.532	29.927
rs11615578	T	C	0.012	0.002	3.04E-09	0.258	34.222
rs12369179	T	C	-0.034	0.003	2.32E-28	0.085	120.291
rs7306544	T	C	-0.014	0.003	4.04E-08	0.881	29.828
rs11614340	T	C	-0.012	0.002	1.87E-10	0.707	42.250
rs9512648	A	G	0.010	0.002	4.11E-08	0.474	31.228
rs7323	C	G	-0.017	0.002	2.18E-18	0.268	76.332
rs4771218	A	G	-0.014	0.002	3.18E-15	0.624	61.361
rs9595908	T	C	0.015	0.002	3.73E-20	0.643	82.062
rs9603697	T	C	0.013	0.002	1.69E-13	0.318	55.420
rs12429545	A	G	0.031	0.002	1.42E-37	0.122	170.085
rs962796	T	C	0.014	0.002	1.88E-11	0.194	44.444
rs9569777	T	G	-0.020	0.002	3.43E-21	0.187	91.612
rs9527895	T	C	-0.016	0.002	3.69E-12	0.819	47.191
rs8181823	A	C	-0.013	0.002	4.36E-10	0.234	39.062
rs9599161	T	C	0.010	0.002	2.32E-09	0.570	37.516
rs1441264	A	G	0.017	0.002	7.60E-25	0.585	104.761
rs9602902	T	C	0.013	0.002	1.57E-14	0.379	62.131
rs1927790	T	C	-0.014	0.002	1.57E-17	0.606	76.562
rs9168	A	C	-0.014	0.002	1.08E-13	0.275	53.521
rs9559022	A	G	-0.012	0.002	4.86E-08	0.816	30.250
rs1536053	T	C	-0.012	0.002	8.02E-11	0.310	41.230
rs12868881	A	T	0.014	0.002	1.40E-15	0.407	65.896
rs10132280	A	C	-0.021	0.002	2.28E-33	0.311	141.345
rs4981693	A	G	0.020	0.002	7.89E-24	0.774	102.010
rs225882	T	C	0.011	0.002	8.56E-10	0.751	39.410
rs1958898	C	G	-0.015	0.002	4.23E-12	0.210	48.335
rs4900714	T	G	0.015	0.002	1.00E-18	0.478	77.854
rs217671	A	G	-0.015	0.002	4.09E-15	0.726	62.327
rs4430672	T	C	0.012	0.002	5.00E-09	0.196	34.866
rs3902951	T	G	-0.013	0.002	2.88E-12	0.769	49.739
rs17182027	A	G	-0.011	0.002	1.46E-10	0.565	41.868
rs7144011	T	G	0.026	0.002	2.37E-40	0.233	172.922
rs4517716	C	G	-0.012	0.002	4.27E-09	0.792	34.222
rs12888545	A	G	-0.013	0.002	1.76E-11	0.746	44.222
rs1951455	T	C	-0.015	0.002	6.05E-15	0.281	60.676
rs9989141	T	C	0.017	0.002	8.16E-23	0.618	97.661
rs8008285	T	C	-0.014	0.002	1.69E-08	0.849	31.641
rs7161194	A	G	0.019	0.002	2.23E-24	0.344	100.000
rs4906263	C	G	-0.018	0.002	8.11E-23	0.656	95.605
rs2273175	T	C	-0.012	0.002	3.69E-11	0.679	45.188

rs12594043	C	G	0.010	0.002	1.31E-09	0.521	36.000
rs7172627	A	G	-0.011	0.002	1.81E-11	0.523	44.969
rs11636611	T	C	0.010	0.002	8.85E-10	0.498	37.426
rs9944219	A	G	-0.012	0.002	8.14E-13	0.604	49.000
rs12912198	T	C	-0.010	0.002	3.44E-08	0.273	30.864
rs6493498	T	C	0.014	0.002	4.84E-17	0.447	73.316
rs10518694	A	C	0.014	0.002	3.43E-09	0.139	36.000
rs12439632	C	G	0.015	0.002	3.68E-10	0.168	39.745
rs339991	A	G	-0.013	0.002	3.51E-13	0.420	54.066
rs1559673	A	C	0.036	0.005	2.21E-13	0.969	53.678
rs2241423	A	G	-0.030	0.002	3.60E-54	0.229	245.994
rs7171864	A	G	0.013	0.002	2.60E-14	0.694	60.291
rs2470893	T	C	0.011	0.002	9.43E-10	0.305	39.616
rs12914623	C	G	-0.016	0.002	2.00E-16	0.272	70.030
rs16946314	A	G	-0.014	0.002	6.85E-11	0.212	42.560
rs11633626	A	C	-0.016	0.002	7.27E-19	0.631	76.077
rs6496248	A	T	0.010	0.002	2.93E-09	0.635	33.383
rs2715423	A	G	-0.012	0.002	1.90E-09	0.281	36.634
rs214249	T	G	0.014	0.002	2.72E-15	0.607	65.896
rs12448257	A	G	0.016	0.002	9.04E-16	0.219	64.802
rs879620	T	C	0.023	0.002	8.45E-39	0.597	176.733
rs2058527	T	G	-0.012	0.002	1.80E-09	0.272	36.634
rs9926784	T	C	0.024	0.002	1.08E-30	0.801	127.367
rs194809	A	G	0.013	0.002	4.86E-09	0.187	32.802
rs2342892	T	G	0.013	0.002	1.33E-13	0.486	54.934
rs7498665	A	G	-0.029	0.002	1.14E-66	0.618	281.055
rs3814883	T	C	0.023	0.002	1.47E-40	0.480	178.301
rs6500208	A	G	0.015	0.002	3.21E-13	0.202	53.290
rs1477199	A	G	-0.022	0.002	5.15E-21	0.843	92.327
rs10852521	T	C	-0.061	0.002	1.00E-200	0.471	1448.750
rs2962449	T	C	0.011	0.002	6.66E-11	0.471	42.633
rs889398	T	C	-0.020	0.002	3.23E-32	0.415	148.535
rs17604662	A	G	-0.018	0.003	1.04E-10	0.890	40.869
rs756717	A	G	-0.013	0.002	2.38E-15	0.394	62.131
rs4390583	A	C	-0.010	0.002	1.63E-08	0.571	30.864
rs12922346	C	G	0.013	0.002	1.48E-11	0.264	44.222
rs7206608	C	G	-0.013	0.002	1.20E-12	0.675	52.160
rs3923783	A	C	-0.022	0.002	4.12E-23	0.178	101.826
rs11078883	C	G	-0.013	0.002	1.30E-12	0.654	49.000
rs5862	A	G	-0.010	0.002	2.33E-09	0.542	36.000
rs1075901	T	C	-0.012	0.002	4.43E-13	0.452	54.390

rs4986044	T	C	-0.018	0.002	1.27E-27	0.455	122.379
rs8065172	A	G	-0.012	0.002	5.29E-10	0.238	38.440
rs12150665	T	C	0.017	0.002	1.74E-24	0.593	110.250
rs6607337	T	C	-0.012	0.002	2.55E-11	0.298	42.593
rs7222349	A	G	0.010	0.002	1.71E-08	0.356	30.864
rs208015	T	C	0.035	0.003	7.17E-26	0.070	113.777
rs7207087	C	G	0.013	0.002	1.82E-12	0.684	52.160
rs11649864	A	G	0.019	0.003	2.52E-10	0.086	40.960
rs12602912	T	C	0.017	0.002	2.90E-16	0.212	68.890
rs312750	A	G	0.010	0.002	2.57E-09	0.499	36.754
rs12939549	A	G	0.018	0.002	3.68E-28	0.557	126.562
rs4075482	A	C	-0.013	0.002	2.04E-13	0.368	53.778
rs8097672	A	T	-0.021	0.002	3.26E-18	0.855	75.835
rs477805	A	G	-0.013	0.002	3.26E-10	0.204	40.716
rs891387	T	C	0.021	0.002	9.26E-35	0.495	149.702
rs1945160	A	G	-0.010	0.002	5.65E-09	0.382	33.383
rs1941696	A	G	0.011	0.002	4.74E-11	0.517	43.405
rs474605	A	G	-0.013	0.002	2.72E-13	0.468	54.066
rs1356506	T	C	0.014	0.002	8.39E-15	0.629	57.929
rs2612576	A	T	-0.011	0.002	8.49E-09	0.295	32.310
rs784257	T	C	-0.015	0.002	3.08E-11	0.188	44.647
rs9951619	T	G	-0.015	0.002	2.33E-15	0.224	64.000
rs6567160	T	C	-0.055	0.002	7.82E-184	0.752	844.053
rs17066856	T	C	0.035	0.003	7.10E-38	0.892	168.038
rs543892	A	G	0.010	0.002	6.99E-09	0.608	31.484
rs17783165	T	C	-0.013	0.002	2.74E-13	0.671	56.692
rs11150911	A	C	0.012	0.002	3.72E-11	0.285	42.975
rs12981256	A	G	0.015	0.002	1.43E-18	0.528	78.896
rs895330	C	G	0.020	0.002	1.59E-19	0.806	79.372
rs273512	T	C	0.016	0.002	4.48E-19	0.409	75.111
rs17724992	A	G	0.017	0.002	5.23E-21	0.720	91.308
rs7258722	A	T	-0.020	0.002	1.81E-30	0.413	138.408
rs12611148	A	C	-0.014	0.002	1.42E-08	0.143	32.585
rs12462975	A	G	0.019	0.002	1.47E-25	0.324	114.966
rs7245985	T	G	0.012	0.002	1.88E-08	0.796	32.111
rs11882409	A	C	0.012	0.002	3.31E-10	0.303	40.557
rs185350	T	C	0.014	0.002	9.10E-17	0.490	73.316
rs769449	A	G	-0.026	0.003	4.44E-21	0.107	91.308
rs11672660	T	C	-0.034	0.002	6.83E-60	0.187	259.056
rs9304665	A	T	0.023	0.002	2.20E-31	0.742	132.250
rs12608738	A	G	0.013	0.002	3.45E-11	0.712	44.679

	rs1884389	T	C	-0.011	0.002	3.72E-10	0.439	40.360
	rs4813619	T	G	-0.011	0.002	1.70E-09	0.531	34.679
	rs1884897	A	G	-0.018	0.002	2.70E-28	0.375	117.148
	rs2423668	T	C	0.011	0.002	7.84E-09	0.420	34.679
	rs852056	T	C	0.012	0.002	2.15E-10	0.254	41.908
	rs1409818	T	C	0.020	0.003	2.59E-12	0.107	48.501
	rs8122855	A	G	0.014	0.002	4.11E-14	0.338	57.929
	rs6059578	C	G	0.010	0.002	6.03E-09	0.668	33.383
	rs16989232	A	G	0.012	0.002	6.19E-12	0.386	45.761
	rs11904898	A	G	-0.014	0.002	3.18E-11	0.234	46.240
	rs2143624	A	G	0.009	0.002	2.10E-08	0.361	30.574
	rs17201143	T	C	-0.011	0.002	1.10E-09	0.305	38.716
	rs17806224	A	G	-0.026	0.002	7.91E-32	0.181	139.669
	rs1304549	A	G	-0.012	0.002	1.35E-08	0.226	31.574
	rs6010784	T	C	0.011	0.002	6.91E-11	0.506	43.891
	rs6512302	C	G	0.013	0.002	1.52E-11	0.739	44.890
	rs2064044	A	C	-0.012	0.002	1.02E-08	0.806	34.306
	rs2832283	A	G	0.012	0.002	4.72E-09	0.225	33.062
	rs13047416	C	G	0.015	0.002	6.93E-18	0.626	71.308
	rs13049280	C	G	0.010	0.002	6.96E-09	0.643	32.744
	rs2183588	A	G	-0.011	0.002	1.91E-10	0.345	40.111
	rs427943	A	C	-0.018	0.002	3.60E-25	0.429	108.405
	rs11538	A	G	-0.014	0.002	1.09E-09	0.828	36.000
	rs2238799	A	G	0.010	0.002	8.65E-09	0.619	32.111
	rs12628891	T	C	-0.012	0.002	5.85E-10	0.319	36.634
	rs12628051	T	C	0.016	0.002	2.90E-19	0.641	80.003
	rs738140	A	G	0.013	0.002	3.08E-12	0.710	51.361
BMI (GIANT)	rs977747	G	T	-0.0168	0.003	2.18E-08	0.533	31.360
	rs17381664	C	T	0.0201	0.0031	4.57E-11	0.425	42.040
	rs7550711	T	C	0.0659	0.0087	5.06E-14	0.034	57.376
	rs2820292	C	A	0.0181	0.0029	5.45E-10	0.508	38.955
	rs7531118	C	T	0.0331	0.003	1.88E-28	0.608	121.734
	rs543874	G	A	0.0497	0.0037	2.29E-40	0.267	180.429
	rs657452	G	A	-0.0227	0.0031	2.12E-13	0.583	53.620
	rs11165643	T	C	0.0221	0.003	1.43E-13	0.575	54.267
	rs1460676	C	T	0.0209	0.0038	4.98E-08	0.217	30.250
	rs1528435	T	C	0.0175	0.003	4.77E-09	0.583	34.028
	rs1016287	C	T	-0.0228	0.0033	4.36E-12	0.675	47.735
	rs2890652	C	T	0.0279	0.0049	1.24E-08	0.125	32.420
	rs13021737	G	A	0.0604	0.0039	5.44E-54	0.875	239.851
	rs6713510	A	G	0.0164	0.0029	1.97E-08	0.483	31.981

rs10182181	G	A	0.0309	0.0029	8.07E-26	0.500	113.532
rs12986742	C	T	0.0207	0.0036	8.92E-09	0.500	33.062
rs17203016	G	A	0.0211	0.0038	3.41E-08	0.200	30.832
rs7599312	A	G	-0.0214	0.0033	4.73E-11	0.292	42.053
rs3849570	A	C	0.0183	0.0033	1.93E-08	0.367	30.752
rs6804842	G	A	0.0183	0.003	8.02E-10	0.575	37.210
rs2365389	T	C	-0.0195	0.003	1.35E-10	0.342	42.250
rs13078960	G	T	0.029	0.0038	1.42E-14	0.183	58.241
rs16851483	T	G	0.0478	0.0075	1.85E-10	0.092	40.619
rs1516725	C	T	0.0448	0.0044	1.39E-24	0.908	103.669
rs17001654	G	C	0.0304	0.0052	5.03E-09	0.158	34.177
rs13130484	T	C	0.0398	0.003	8.01E-41	0.433	176.003
rs13107325	T	C	0.0472	0.0066	1.06E-12	0.117	51.144
rs11727676	C	T	-0.0365	0.0063	6.25E-09	0.075	33.566
rs2112347	G	T	-0.0254	0.003	1.96E-17	0.375	71.684
rs7715256	T	G	-0.0168	0.0029	8.85E-09	0.550	33.560
rs6457796	C	T	0.0209	0.0033	2.54E-10	0.258	40.111
rs943005	T	C	0.0444	0.0038	4.52E-31	0.100	136.520
rs2033529	G	A	0.0183	0.0032	1.45E-08	0.258	32.704
rs13201877	G	A	0.0236	0.0043	4.28E-08	0.083	30.122
rs13191362	G	A	-0.0285	0.0047	1.09E-09	0.200	36.770
rs9374842	T	C	0.0196	0.0034	7.20E-09	0.742	33.232
rs3800229	T	G	0.0175	0.0032	4.95E-08	0.692	29.907
rs1167827	G	A	0.02	0.0031	1.98E-10	0.542	41.623
rs2060604	C	T	-0.0203	0.003	9.46E-12	0.442	45.788
rs2183825	C	T	0.0241	0.0032	2.22E-14	0.292	56.719
rs4740619	C	T	-0.017	0.0029	6.36E-09	0.467	34.364
rs1928295	C	T	-0.0182	0.0029	4.32E-10	0.425	39.386
rs6477694	T	C	-0.0169	0.003	1.70E-08	0.642	31.734
rs10733682	G	A	-0.0188	0.003	2.45E-10	0.575	39.271
rs7903146	T	C	-0.0235	0.0033	1.10E-12	0.250	50.711
rs7899106	G	A	0.0379	0.0067	1.27E-08	0.050	31.998
rs17094222	C	T	0.0249	0.0037	2.19E-11	0.208	45.289
rs2176598	C	T	-0.0185	0.0033	3.47E-08	0.800	31.428
rs3817334	T	C	0.0256	0.003	1.17E-17	0.450	72.817
rs12286929	G	A	0.0211	0.0029	5.44E-13	0.433	52.938
rs10840100	G	A	0.0206	0.003	6.67E-12	0.725	47.151
rs11030104	G	A	-0.0416	0.0037	6.66E-30	0.200	126.410
rs7138803	A	G	0.032	0.003	5.11E-26	0.442	113.777
rs11057405	A	G	-0.0304	0.0053	1.22E-08	0.092	32.900
rs1441264	A	G	0.0172	0.0031	2.96E-08	0.550	30.784

	rs9579083	C	G	0.0295	0.0046	1.43E-10	0.233	41.127
	rs12429545	A	G	0.0324	0.0044	3.15E-13	0.100	54.223
	rs9540493	G	A	-0.0182	0.0031	3.95E-09	0.550	34.468
	rs7144011	T	G	0.0274	0.0035	6.05E-15	0.275	61.286
	rs10132280	A	C	-0.0221	0.0033	1.40E-11	0.333	44.849
	rs13329567	T	C	-0.0307	0.0035	1.53E-18	0.217	76.938
	rs3736485	G	A	-0.016	0.0029	4.52E-08	0.575	30.440
	rs12448257	A	G	0.0246	0.0037	3.90E-11	0.225	44.204
	rs879620	T	C	0.0244	0.0039	3.94E-10	0.592	39.142
	rs9926784	C	T	-0.0249	0.0038	8.55E-11	0.208	42.937
	rs4889606	G	A	-0.0187	0.003	6.58E-10	0.358	38.854
	rs1421085	C	T	0.0803	0.003	2.17E-158	0.450	716.450
	rs3888190	A	C	0.0311	0.003	3.45E-25	0.358	107.467
	rs12940622	A	G	-0.0183	0.0029	3.64E-10	0.458	39.820
	rs1000940	G	A	0.0184	0.0033	1.81E-08	0.225	31.089
	rs6567160	C	T	0.0562	0.0035	6.68E-59	0.283	257.830
	rs17066856	C	T	-0.0371	0.005	2.00E-13	0.133	55.056
	rs891389	T	C	0.0209	0.0037	1.62E-08	0.325	31.907
	rs14810	G	C	0.0183	0.0033	1.92E-08	0.675	30.752
	rs11672660	T	C	-0.0339	0.0038	7.91E-19	0.175	79.585
	rs9304665	A	T	0.0243	0.0043	1.59E-08	0.700	31.935
	rs17724992	G	A	-0.0196	0.0034	7.79E-09	0.308	33.232
	rs6091540	T	C	-0.0185	0.0033	2.14E-08	0.275	31.428
	rs2836754	C	T	0.0169	0.003	1.60E-08	0.650	31.734
WC (UKB)	rs72634826	A	G	-0.015	0.002	5.40E-13	0.260	52.055
	rs7537581	A	C	0.011	0.002	2.60E-09	0.532	35.478
	rs3866805	A	C	0.010	0.002	2.50E-08	0.356	31.048
	rs4908672	T	C	0.011	0.002	4.70E-10	0.393	38.780
	rs156902	T	G	-0.014	0.002	1.40E-08	0.267	32.152
	rs649458	A	T	-0.018	0.003	1.50E-12	0.860	50.054
	rs945211	C	G	0.010	0.002	4.10E-08	0.616	30.125
	rs6682438	C	T	0.012	0.002	5.40E-11	0.673	43.032
	rs3768321	T	G	0.018	0.002	3.80E-15	0.197	61.776
	rs6669341	G	A	-0.013	0.002	4.50E-12	0.583	47.873
	rs6693294	G	A	-0.017	0.002	1.10E-18	0.689	77.827
	rs587271	T	C	0.012	0.002	4.10E-09	0.687	34.568
	rs12140153	T	G	-0.027	0.003	2.10E-17	0.094	72.076
	rs7519259	A	G	0.013	0.002	1.70E-12	0.528	49.750
	rs2568958	A	G	0.017	0.002	1.60E-20	0.604	86.260
	rs3845344	T	C	0.011	0.002	4.30E-09	0.391	34.483
	rs34517439	A	C	0.031	0.003	1.90E-28	0.122	122.365

rs11162968	C	T	0.012	0.002	2.10E-10	0.316	40.389
rs11165493	A	G	0.011	0.002	1.30E-08	0.343	32.282
rs588660	A	G	0.016	0.002	1.00E-17	0.584	73.454
rs12072739	G	A	0.016	0.002	3.90E-14	0.224	57.207
rs41279738	G	T	0.052	0.006	1.70E-20	0.026	86.057
rs2618039	T	A	0.012	0.002	5.20E-11	0.381	43.096
rs61813324	T	C	0.022	0.003	9.50E-17	0.136	69.062
rs35216639	A	C	0.017	0.003	3.30E-09	0.111	35.010
rs75035127	G	A	-0.033	0.005	1.50E-10	0.030	41.060
rs539515	C	A	0.038	0.002	1.50E-65	0.205	292.414
rs815163	C	T	-0.013	0.002	2.70E-13	0.563	53.387
rs2678204	G	T	0.016	0.002	7.90E-17	0.340	69.445
rs4562625	G	C	-0.011	0.002	6.10E-09	0.615	33.787
rs1507331	G	C	0.016	0.003	1.50E-09	0.132	36.531
rs1108548	G	A	0.012	0.002	1.20E-09	0.277	37.035
rs12042959	G	A	-0.015	0.003	5.20E-09	0.144	34.114
rs77165542	T	C	-0.070	0.005	9.90E-47	0.035	206.073
rs12463617	C	A	0.043	0.002	1.80E-74	0.828	333.317
rs1405261	A	T	-0.010	0.002	4.80E-08	0.434	29.794
rs76286777	C	T	0.023	0.002	1.80E-27	0.218	117.872
rs1731246	T	G	-0.012	0.002	1.30E-08	0.757	32.328
rs10194302	C	T	-0.010	0.002	3.50E-08	0.564	30.414
rs11898037	C	T	0.011	0.002	1.20E-08	0.368	32.526
rs13410783	G	A	0.014	0.002	2.40E-14	0.370	58.149
rs10185199	A	G	-0.011	0.002	2.00E-08	0.281	31.503
rs72618637	A	T	-0.013	0.002	2.10E-08	0.189	31.397
rs13420048	A	C	-0.013	0.002	7.00E-13	0.365	51.542
rs1861410	T	C	-0.016	0.002	6.40E-19	0.555	78.926
rs6739755	G	A	-0.016	0.002	3.10E-18	0.603	75.814
rs2861692	C	T	-0.017	0.002	7.70E-17	0.275	69.478
rs35681682	C	T	-0.010	0.002	1.00E-08	0.408	32.807
rs308911	G	A	-0.011	0.002	6.90E-09	0.714	33.568
rs4851283	G	C	-0.017	0.002	2.50E-19	0.685	80.803
rs1441098	T	A	-0.010	0.002	3.50E-08	0.544	30.396
rs3087523	A	G	0.017	0.003	1.00E-09	0.125	37.242
rs13033310	A	G	0.013	0.002	1.30E-09	0.253	36.842
rs114964326	A	G	-0.030	0.005	2.10E-08	0.029	31.445
rs429343	G	A	-0.012	0.002	5.90E-12	0.577	47.356
rs10803762	A	G	0.012	0.002	4.40E-10	0.677	38.911
rs62174721	G	A	-0.011	0.002	4.90E-08	0.240	29.761
rs10184230	T	C	-0.012	0.002	8.90E-11	0.648	42.044

rs34234296	A	G	-0.013	0.002	1.30E-12	0.392	50.396
rs1609303	A	T	0.015	0.002	2.10E-16	0.631	67.525
rs12478299	C	T	-0.012	0.002	1.90E-08	0.252	31.546
rs11675464	G	A	0.011	0.002	3.70E-10	0.563	39.279
rs4482463	A	C	-0.026	0.003	1.30E-14	0.923	59.439
rs73985439	C	A	0.012	0.002	1.60E-10	0.307	40.925
rs13427822	G	A	-0.014	0.002	3.20E-12	0.271	48.560
rs72617140	C	A	0.015	0.002	3.00E-12	0.214	48.712
rs35882248	T	C	0.016	0.002	2.80E-16	0.317	66.970
rs62246311	A	G	0.020	0.003	3.50E-12	0.102	48.371
rs1801282	G	C	0.019	0.003	2.60E-12	0.120	48.968
rs361222	T	A	-0.012	0.002	4.40E-11	0.632	43.413
rs2470549	C	T	-0.012	0.002	5.20E-11	0.598	43.091
rs6791983	A	C	0.012	0.002	2.30E-09	0.750	35.741
rs10490869	T	A	0.016	0.002	1.20E-13	0.210	54.969
rs1799923	G	A	0.017	0.003	1.20E-09	0.887	36.968
rs28350	G	A	-0.014	0.002	2.50E-09	0.821	35.556
rs1554654	T	C	-0.010	0.002	1.90E-08	0.470	31.589
rs9843653	C	T	0.020	0.002	6.70E-28	0.512	119.885
rs74395133	C	T	-0.016	0.003	6.60E-10	0.134	38.133
rs62243489	G	T	-0.016	0.002	3.00E-14	0.259	57.717
rs557951	G	T	0.012	0.002	4.00E-10	0.313	39.103
rs7630382	T	C	0.013	0.002	9.30E-14	0.532	55.514
rs62261725	G	A	-0.015	0.002	6.70E-15	0.326	60.691
rs6551304	G	A	0.017	0.002	2.10E-12	0.832	49.398
rs1454687	G	C	-0.016	0.002	1.50E-19	0.515	81.788
rs1436348	G	A	0.012	0.002	5.40E-12	0.583	47.542
rs654060	A	T	0.010	0.002	1.70E-08	0.463	31.791
rs9814758	G	T	-0.011	0.002	2.70E-09	0.356	35.388
rs1320903	A	G	0.017	0.002	5.20E-19	0.320	79.349
rs1471740	C	T	0.018	0.002	1.60E-18	0.740	77.121
rs9289630	C	G	0.014	0.002	3.50E-15	0.389	61.963
rs12107172	G	A	0.016	0.003	1.80E-09	0.136	36.145
rs9835303	A	C	0.011	0.002	6.20E-10	0.570	38.242
rs13322435	G	A	-0.017	0.002	1.90E-20	0.404	85.885
rs4856717	C	G	0.011	0.002	2.00E-10	0.541	40.448
rs8192675	C	T	0.016	0.002	4.50E-16	0.289	65.995
rs1117619	G	C	-0.012	0.002	5.00E-09	0.250	34.208
rs73052033	C	T	-0.021	0.002	7.90E-20	0.185	83.070
rs7372674	A	C	0.012	0.002	1.70E-10	0.357	40.747
rs1051613	A	G	-0.010	0.002	3.10E-08	0.545	30.656

rs11731255	G	A	0.011	0.002	1.30E-09	0.474	36.822
rs35800195	T	A	0.012	0.002	1.30E-10	0.320	41.314
rs6849518	T	C	0.022	0.003	7.10E-16	0.124	65.101
rs4527444	G	A	0.011	0.002	4.50E-09	0.541	34.402
rs10938398	A	G	0.022	0.002	1.00E-34	0.434	150.997
rs3113509	T	C	-0.012	0.002	1.00E-09	0.732	37.263
rs1346841	A	G	-0.011	0.002	6.40E-09	0.405	33.717
rs923994	G	A	-0.013	0.002	2.20E-09	0.783	35.793
rs4148155	G	A	-0.019	0.003	1.50E-11	0.113	45.522
rs4419475	T	A	0.011	0.002	6.00E-10	0.407	38.316
rs1229984	C	T	0.030	0.005	2.80E-08	0.973	30.868
rs4525978	T	C	-0.011	0.002	2.10E-08	0.735	31.398
rs7377083	A	C	0.014	0.002	3.20E-15	0.431	62.136
rs59068084	T	G	0.010	0.002	2.50E-08	0.410	31.064
rs11099020	T	C	-0.011	0.002	1.70E-09	0.641	36.313
rs1296328	C	A	-0.013	0.002	3.10E-13	0.559	53.168
rs6536575	C	T	0.011	0.002	1.40E-09	0.519	36.697
rs1037702	A	G	-0.010	0.002	3.60E-08	0.622	30.350
rs9654453	C	T	0.017	0.003	1.70E-10	0.129	40.762
rs400031	G	A	0.012	0.002	2.10E-08	0.756	31.425
rs3936510	T	G	0.014	0.002	7.30E-10	0.201	37.932
rs10471636	A	G	-0.010	0.002	3.00E-08	0.509	30.733
rs36140	C	A	0.011	0.002	2.10E-09	0.635	35.834
rs2307111	C	T	-0.024	0.002	3.30E-39	0.395	171.617
rs876605	G	A	-0.011	0.002	4.20E-08	0.740	30.064
rs67002381	G	A	-0.011	0.002	2.90E-08	0.327	30.759
rs7442885	G	C	-0.020	0.002	6.80E-21	0.214	87.917
rs34483452	A	C	0.027	0.003	7.30E-25	0.136	106.021
rs67632512	A	C	0.017	0.003	3.80E-09	0.117	34.729
rs2161097	T	C	0.014	0.002	2.20E-15	0.438	62.911
rs40067	A	G	-0.016	0.002	6.10E-11	0.170	42.793
rs1625623	T	C	0.011	0.002	9.80E-09	0.372	32.882
rs347551	G	C	0.013	0.002	3.90E-12	0.472	48.174
rs1582931	A	G	-0.014	0.002	1.70E-14	0.473	58.860
rs4706004	G	A	-0.013	0.002	5.40E-10	0.217	38.530
rs13163306	A	G	-0.010	0.002	3.50E-08	0.466	30.405
rs2133561	T	A	-0.012	0.002	3.90E-11	0.611	43.669
rs852983	A	G	-0.010	0.002	4.20E-08	0.460	30.058
rs4469245	T	A	-0.012	0.002	1.10E-09	0.663	37.109
rs7708584	G	A	-0.012	0.002	1.40E-11	0.572	45.649
rs4552632	A	G	-0.010	0.002	3.30E-08	0.617	30.504

rs245767	G	A	0.015	0.002	4.50E-13	0.730	52.412
rs9378676	C	A	0.013	0.002	7.00E-10	0.234	38.017
rs10947793	G	A	-0.013	0.002	8.10E-12	0.372	46.751
rs11757278	C	T	-0.013	0.002	4.90E-11	0.304	43.233
rs112266013	A	G	-0.015	0.003	5.20E-09	0.144	34.129
rs3806114	A	G	-0.011	0.002	1.80E-08	0.668	31.723
rs2183947	A	G	-0.022	0.002	4.90E-25	0.225	106.810
rs28366156	C	T	-0.019	0.003	1.50E-12	0.131	50.092
rs7752202	T	C	0.018	0.003	2.70E-12	0.145	48.878
rs2744938	G	A	0.032	0.003	5.40E-37	0.148	161.460
rs34045288	T	C	0.020	0.002	5.80E-27	0.334	115.618
rs72892910	T	G	0.030	0.002	5.20E-37	0.172	161.531
rs1327259	G	A	-0.012	0.002	3.10E-10	0.388	39.639
rs1321519	G	A	0.014	0.002	5.20E-14	0.345	56.660
rs9370243	T	G	0.020	0.003	1.90E-09	0.082	36.045
rs1570298	T	A	0.012	0.002	3.80E-09	0.743	34.709
rs1902066	C	T	0.011	0.002	1.40E-09	0.562	36.601
rs9294260	A	G	0.013	0.002	2.40E-13	0.477	53.644
rs10499014	G	C	-0.013	0.002	5.90E-11	0.269	42.858
rs6938973	C	T	0.012	0.002	4.90E-11	0.601	43.217
rs2253310	G	C	0.018	0.002	6.50E-23	0.626	97.135
rs520478	T	G	-0.013	0.002	2.30E-10	0.701	40.187
rs577721086	C	T	0.031	0.004	2.00E-13	0.050	53.968
rs765876	G	A	-0.010	0.002	4.10E-08	0.489	30.099
rs7755574	T	G	0.011	0.002	3.90E-08	0.283	30.191
rs9478496	C	T	0.015	0.002	1.90E-09	0.164	36.115
rs36007635	A	G	-0.017	0.003	1.70E-10	0.138	40.752
rs1182199	A	C	-0.014	0.002	3.60E-12	0.304	48.331
rs4722398	T	C	0.017	0.003	1.00E-10	0.136	41.740
rs73068448	T	C	-0.015	0.002	2.00E-10	0.171	40.430
rs215669	A	G	-0.013	0.002	1.10E-11	0.612	46.098
rs2237403	T	C	-0.011	0.002	6.60E-09	0.338	33.650
rs3807566	T	G	-0.012	0.002	1.70E-11	0.438	45.261
rs4718964	T	G	0.012	0.002	1.80E-11	0.413	45.190
rs11767811	A	G	-0.015	0.002	8.90E-11	0.181	42.041
rs58862095	T	C	-0.017	0.002	7.10E-20	0.419	83.296
rs7792176	C	T	-0.016	0.002	2.20E-10	0.842	40.251
rs3729628	T	C	0.012	0.002	1.20E-08	0.756	32.563
rs10269774	A	G	0.012	0.002	3.60E-10	0.326	39.301
rs883403	C	T	-0.018	0.002	9.50E-13	0.154	50.952
rs12375196	A	C	0.013	0.002	3.80E-13	0.424	52.726

rs2470946	T	G	0.012	0.002	1.50E-10	0.401	41.021
rs4344019	G	A	0.020	0.003	5.70E-10	0.917	38.422
rs10248298	A	C	0.013	0.002	8.80E-13	0.366	51.101
rs55794894	A	G	-0.015	0.003	2.80E-08	0.135	30.857
rs11773362	T	C	-0.010	0.002	3.20E-08	0.336	30.610
rs10236214	T	C	0.014	0.002	1.90E-13	0.642	54.126
rs1559900	T	C	0.013	0.002	1.10E-10	0.286	41.668
rs59104534	T	C	0.011	0.002	4.70E-08	0.299	29.848
rs17446091	C	T	0.015	0.002	2.30E-11	0.202	44.737
rs2725371	G	A	-0.015	0.002	2.60E-15	0.696	62.572
rs36061954	T	C	0.011	0.002	3.60E-10	0.399	39.341
rs10957087	A	T	0.014	0.002	1.20E-08	0.160	32.560
rs13264909	T	A	-0.012	0.002	1.20E-11	0.429	46.028
rs7845090	A	G	-0.020	0.002	6.30E-23	0.709	97.182
rs1609010	G	A	0.015	0.002	1.30E-16	0.566	68.380
rs10111287	T	C	-0.011	0.002	4.60E-08	0.284	29.871
rs13273726	A	C	0.013	0.002	7.10E-09	0.219	33.500
rs11778934	G	C	-0.012	0.002	1.10E-11	0.536	46.093
rs4876611	G	A	0.015	0.002	5.30E-14	0.720	56.613
rs12549000	A	T	0.016	0.003	1.90E-08	0.106	31.548
rs746839	G	C	0.012	0.002	2.40E-10	0.375	40.133
rs4072917	A	G	0.012	0.002	7.40E-11	0.474	42.415
rs4740627	C	T	-0.015	0.002	3.40E-16	0.442	66.538
rs10962550	C	G	0.014	0.002	8.40E-10	0.183	37.674
rs1111817	G	C	-0.011	0.002	2.00E-08	0.365	31.514
rs13288841	A	G	0.019	0.002	2.20E-23	0.322	99.240
rs10969298	C	T	-0.010	0.002	5.00E-08	0.421	29.701
rs12001437	C	T	0.011	0.002	3.80E-09	0.368	34.721
rs7034554	G	A	-0.011	0.002	1.10E-09	0.374	37.149
rs1360201	T	C	0.010	0.002	4.40E-08	0.482	29.964
rs1619442	C	T	-0.016	0.003	2.40E-10	0.859	40.149
rs1475535	A	T	0.011	0.002	1.80E-10	0.498	40.637
rs2482704	T	G	-0.012	0.002	1.50E-10	0.427	40.993
rs10992854	C	T	-0.011	0.002	5.40E-09	0.682	34.041
rs4742782	G	C	0.012	0.002	8.00E-11	0.316	42.250
rs1019240	T	A	0.012	0.002	3.70E-11	0.643	43.774
rs1752169	A	C	0.014	0.002	4.50E-12	0.251	47.914
rs113132247	A	G	0.015	0.002	8.40E-10	0.153	37.666
rs7094644	A	G	0.013	0.002	2.70E-11	0.678	44.380
rs11012732	G	A	0.019	0.002	2.40E-24	0.332	103.629
rs71495038	A	G	0.022	0.003	2.60E-11	0.077	44.445

rs10827380	T	C	0.012	0.002	1.80E-09	0.314	36.182
rs2696309	C	T	0.011	0.002	1.60E-08	0.720	31.895
rs7070670	T	C	-0.012	0.002	3.50E-10	0.328	39.377
rs10824211	T	C	0.015	0.003	6.30E-09	0.140	33.751
rs703983	G	A	-0.011	0.002	3.10E-10	0.415	39.581
rs12245654	C	A	0.023	0.003	3.20E-11	0.073	44.073
rs10887578	C	G	0.011	0.002	3.10E-09	0.498	35.137
rs2439823	G	A	0.016	0.002	6.80E-18	0.546	74.274
rs4075353	A	G	-0.011	0.002	2.20E-08	0.344	31.316
rs4290163	T	G	0.011	0.002	5.40E-10	0.393	38.526
rs11196657	C	T	0.012	0.002	3.40E-09	0.237	34.921
rs10787738	T	C	0.015	0.002	2.10E-13	0.255	53.865
rs67609008	C	T	0.011	0.002	2.50E-08	0.284	31.051
rs2172131	C	T	-0.012	0.002	1.90E-11	0.579	45.091
rs1502317	T	C	-0.017	0.002	4.30E-18	0.277	75.177
rs11824092	C	T	0.012	0.002	2.40E-11	0.636	44.585
rs1013402	G	A	0.025	0.002	3.90E-39	0.318	171.266
rs1765133	T	C	-0.016	0.002	1.40E-13	0.226	54.710
rs10835676	G	C	0.012	0.002	4.00E-09	0.240	34.644
rs7928523	T	C	0.017	0.002	1.70E-19	0.317	81.608
rs7115013	T	C	-0.011	0.002	3.70E-09	0.443	34.759
rs12287076	C	G	0.021	0.002	1.30E-26	0.707	114.049
rs12225345	G	A	0.015	0.002	1.30E-09	0.166	36.857
rs7952436	T	C	-0.029	0.003	5.90E-19	0.082	79.103
rs11603984	T	G	-0.017	0.003	8.90E-11	0.135	42.042
rs61903695	G	A	0.013	0.002	5.90E-11	0.255	42.847
rs35023999	C	A	-0.011	0.002	2.40E-10	0.508	40.151
rs11215381	C	T	0.011	0.002	4.60E-10	0.526	38.824
rs12273545	T	C	0.022	0.004	9.40E-09	0.056	32.963
rs7925100	A	G	0.014	0.002	1.70E-14	0.396	58.897
rs11218510	A	G	-0.012	0.002	2.90E-10	0.400	39.768
rs7933085	G	A	0.011	0.002	1.40E-09	0.508	36.641
rs11223204	G	A	0.012	0.002	1.50E-11	0.434	45.598
rs111258054	T	C	0.016	0.002	9.50E-12	0.184	46.436
rs55726687	A	G	0.020	0.002	1.10E-19	0.210	82.476
rs76895963	G	T	0.048	0.007	2.80E-12	0.021	48.807
rs10772985	A	G	-0.011	0.002	1.50E-08	0.301	31.992
rs10505836	C	A	0.015	0.003	4.10E-09	0.860	34.555
rs10842240	C	G	0.015	0.003	3.00E-08	0.117	30.729
rs1458156	T	C	0.015	0.002	3.50E-16	0.488	66.482
rs7132908	A	G	0.022	0.002	1.40E-31	0.384	136.700

rs894736	G	A	0.016	0.002	3.60E-17	0.363	70.969
rs704061	C	T	0.015	0.002	4.00E-16	0.455	66.242
rs735033	G	A	-0.010	0.002	2.50E-08	0.605	31.022
rs2373980	A	T	-0.012	0.002	1.30E-11	0.576	45.746
rs3764002	T	C	-0.016	0.002	2.80E-15	0.262	62.412
rs76653871	C	T	-0.021	0.004	1.90E-08	0.063	31.552
rs145350287	A	T	-0.032	0.005	2.00E-12	0.040	49.476
rs36165342	C	T	0.011	0.002	2.10E-09	0.479	35.834
rs11058233	G	A	0.016	0.002	1.20E-14	0.751	59.610
rs900448	G	C	0.013	0.002	5.80E-12	0.307	47.410
rs9512696	G	A	0.012	0.002	4.20E-11	0.662	43.519
rs11842871	T	G	-0.013	0.002	4.00E-10	0.260	39.126
rs1336486	G	T	0.012	0.002	6.00E-11	0.329	42.836
rs9568867	A	G	0.023	0.003	4.20E-17	0.129	70.687
rs9316661	C	T	-0.016	0.002	3.80E-12	0.801	48.245
rs34140906	C	T	-0.018	0.002	5.40E-14	0.170	56.581
rs7324067	C	T	0.012	0.002	7.70E-09	0.761	33.342
rs1441264	A	G	0.015	0.002	7.60E-16	0.594	64.977
rs61969511	A	G	0.012	0.002	3.80E-09	0.279	34.705
rs12877270	A	G	0.012	0.002	1.10E-10	0.442	41.689
rs9584870	C	T	-0.011	0.002	1.10E-08	0.366	32.656
rs9888533	T	C	0.011	0.002	3.00E-09	0.538	35.210
rs484455	A	G	-0.012	0.002	1.40E-10	0.481	41.222
rs1183668	G	C	-0.012	0.002	9.30E-11	0.370	41.955
rs7154982	A	G	-0.017	0.002	1.80E-16	0.270	67.832
rs2180454	C	T	0.018	0.002	3.70E-17	0.772	70.914
rs1191600	A	C	-0.011	0.002	3.80E-09	0.594	34.701
rs4900715	A	G	-0.011	0.002	1.90E-10	0.507	40.609
rs1188209	G	A	0.010	0.002	1.60E-08	0.552	31.957
rs217672	C	A	0.013	0.002	2.80E-10	0.272	39.816
rs10150482	A	G	0.022	0.002	4.50E-24	0.220	102.427
rs6575340	A	G	0.016	0.002	4.60E-18	0.636	75.063
rs11160600	G	A	0.018	0.003	7.10E-09	0.092	33.511
rs61992671	G	A	-0.013	0.002	3.80E-12	0.492	48.247
rs12880641	G	T	-0.014	0.002	2.40E-13	0.662	53.680
rs3212038	G	A	0.013	0.002	3.50E-11	0.329	43.863
rs8024137	T	A	0.014	0.003	4.60E-08	0.848	29.895
rs11636611	T	C	0.011	0.002	2.30E-09	0.503	35.710
rs6493498	C	T	-0.013	0.002	4.20E-13	0.545	52.528
rs80243702	A	G	0.015	0.002	6.70E-10	0.161	38.118
rs1657930	A	G	-0.014	0.002	2.30E-10	0.803	40.166

rs3784692	T	C	0.019	0.002	2.60E-24	0.602	103.492
rs7171864	A	G	0.013	0.002	1.10E-11	0.660	46.070
rs34994596	C	T	-0.015	0.002	1.10E-13	0.297	55.251
rs17296856	C	A	-0.016	0.002	5.00E-15	0.281	61.245
rs7498044	A	G	-0.015	0.002	4.90E-12	0.217	47.725
rs7169847	T	G	-0.010	0.002	3.80E-08	0.636	30.237
rs56803094	G	A	-0.013	0.002	2.60E-09	0.227	35.435
rs12926311	C	G	-0.012	0.002	3.20E-11	0.354	44.043
rs13333747	C	T	-0.023	0.002	1.90E-22	0.183	95.028
rs879620	T	C	0.019	0.002	4.90E-26	0.613	111.389
rs28375268	T	G	-0.013	0.002	3.30E-12	0.645	48.518
rs11639596	C	A	-0.012	0.002	1.10E-08	0.251	32.638
rs9926784	C	T	-0.013	0.002	8.70E-09	0.185	33.101
rs12103006	G	A	0.012	0.002	5.10E-12	0.569	47.640
rs7498665	G	A	0.027	0.002	4.90E-48	0.400	212.070
rs3814883	T	C	0.024	0.002	1.10E-40	0.482	178.425
rs16952396	A	C	0.014	0.002	5.80E-09	0.163	33.912
rs7193144	C	T	0.056	0.002	1.00E-200	0.395	927.794
rs862320	T	C	-0.018	0.002	4.40E-23	0.410	97.907
rs756717	A	G	-0.011	0.002	6.80E-09	0.399	33.585
rs862227	G	A	-0.011	0.002	8.80E-10	0.458	37.566
rs9673839	G	A	0.011	0.002	1.30E-09	0.491	36.768
rs1025065	G	T	-0.010	0.002	3.60E-08	0.639	30.366
rs7206608	G	C	0.012	0.002	3.80E-10	0.322	39.211
rs2376885	A	G	-0.011	0.002	2.80E-08	0.324	30.848
rs4790841	T	C	-0.021	0.002	1.30E-17	0.154	73.007
rs9902846	T	C	0.013	0.002	4.20E-12	0.316	48.033
rs3826408	T	C	0.011	0.002	4.10E-10	0.457	39.071
rs17681738	T	C	0.011	0.002	3.30E-08	0.329	30.507
rs8078135	T	C	-0.010	0.002	1.60E-08	0.490	31.893
rs2020942	T	C	0.011	0.002	3.40E-09	0.395	34.913
rs319775	C	T	0.010	0.002	3.00E-08	0.609	30.679
rs2306593	T	C	-0.015	0.002	1.60E-17	0.488	72.569
rs9916444	G	C	0.011	0.002	1.30E-09	0.342	36.847
rs11653367	G	A	-0.015	0.002	2.50E-15	0.328	62.603
rs113866544	C	T	0.030	0.004	1.40E-17	0.068	72.884
rs62072003	T	C	0.014	0.003	1.80E-08	0.143	31.710
rs7218014	C	T	0.022	0.002	2.70E-23	0.197	98.846
rs11150745	G	A	-0.016	0.002	6.80E-17	0.318	69.743
rs3935190	A	G	-0.013	0.002	2.80E-12	0.537	48.812
rs8097672	T	A	0.017	0.003	5.00E-11	0.145	43.165

	rs2584205	A	G	0.011	0.002	3.70E-08	0.733	30.319
	rs1788808	G	A	-0.021	0.002	3.60E-31	0.495	134.833
	rs559231	T	G	0.011	0.002	5.20E-09	0.393	34.105
	rs1834144	A	C	-0.015	0.002	3.40E-15	0.373	62.003
	rs784257	C	T	0.016	0.002	2.30E-12	0.813	49.215
	rs6567160	C	T	0.045	0.002	7.19E-101	0.233	454.611
	rs57636386	C	T	-0.031	0.003	1.50E-21	0.084	90.945
	rs1942826	A	G	0.017	0.003	4.90E-10	0.126	38.700
	rs11666480	G	C	0.013	0.002	1.90E-13	0.536	54.115
	rs2074881	T	C	-0.015	0.002	8.00E-10	0.168	37.768
	rs72976986	A	G	-0.016	0.002	7.80E-12	0.190	46.808
	rs2302209	T	C	0.020	0.002	1.10E-23	0.289	100.624
	rs12983532	T	C	-0.015	0.002	7.60E-13	0.251	51.391
	rs8112818	G	A	-0.016	0.002	3.80E-18	0.400	75.421
	rs12462975	A	G	0.018	0.002	5.90E-20	0.330	83.650
	rs2903738	T	A	-0.013	0.002	6.80E-10	0.221	38.067
	rs10406327	G	C	0.010	0.002	8.00E-09	0.479	33.273
	rs429358	C	T	-0.027	0.002	7.50E-28	0.154	119.659
	rs10423928	A	T	-0.027	0.002	6.80E-32	0.194	138.131
	rs7259070	C	T	0.015	0.002	6.60E-17	0.596	69.777
	rs1101124	G	A	-0.012	0.002	3.00E-08	0.759	30.694
	rs4456769	T	C	0.013	0.002	1.80E-12	0.333	49.724
	rs6030803	C	T	-0.018	0.003	6.10E-11	0.127	42.778
	rs73142879	T	C	-0.024	0.002	2.80E-26	0.192	112.498
	rs6069037	A	C	-0.011	0.002	3.60E-08	0.731	30.331
	rs1056441	C	T	0.013	0.002	3.10E-11	0.675	44.094
	rs13047416	G	C	-0.014	0.002	6.60E-14	0.377	56.194
	rs76040172	A	G	-0.029	0.004	1.50E-13	0.054	54.600
	rs11704728	T	C	0.013	0.002	3.90E-09	0.196	34.660
	rs165656	C	G	0.010	0.002	1.10E-08	0.517	32.574
	rs4470425	A	C	-0.011	0.002	2.40E-08	0.318	31.152
	rs28489620	A	G	-0.012	0.002	4.40E-10	0.290	38.949
WC (GIANT)	rs7550711	T	C	0.058	0.0098	3.40E-09	0.034	35.027
	rs7531118	C	T	0.027	0.0035	1.50E-14	0.608	59.510
	rs3127553	A	G	-0.023	0.0035	1.60E-10	0.633	43.183
	rs2820292	C	A	0.019	0.0034	2.40E-08	0.508	31.228
	rs17381664	C	T	0.022	0.0035	4.20E-10	0.425	39.510
	rs11165623	A	G	0.02	0.0034	5.20E-09	0.483	34.602
	rs633715	C	T	0.043	0.0043	3.30E-23	0.267	99.999
	rs6545714	A	G	-0.022	0.0035	1.90E-10	0.625	39.510
	rs6755502	C	T	0.051	0.0045	2.00E-30	0.875	128.443

	rs929641	G	A	-0.021	0.0034	1.20E-09	0.383	38.148
	rs3849570	A	C	0.021	0.0038	2.20E-08	0.367	30.540
	rs6440003	A	G	0.021	0.0034	2.90E-10	0.483	38.148
	rs2325036	C	A	-0.023	0.0035	2.10E-11	0.408	43.183
	rs1516725	C	T	0.031	0.0051	1.70E-09	0.908	36.947
	rs10938397	G	A	0.032	0.0035	6.10E-20	0.433	83.591
	rs2112347	G	T	-0.025	0.0035	3.20E-13	0.375	51.020
	rs806794	G	A	-0.022	0.0037	2.10E-09	0.275	35.354
	rs9400239	C	T	0.024	0.0036	1.90E-11	0.700	44.444
	rs2489623	C	A	0.019	0.0034	3.40E-08	0.558	31.228
	rs2033529	G	A	0.021	0.0037	1.70E-08	0.258	32.213
	rs943005	T	C	0.039	0.0044	7.20E-19	0.100	78.563
	rs16894959	C	T	0.026	0.0048	3.40E-08	0.100	29.340
	rs10968576	G	A	0.025	0.0036	1.20E-11	0.292	48.225
	rs6163	A	C	0.019	0.0035	3.70E-08	0.392	29.469
	rs7903146	T	C	-0.022	0.0037	3.90E-09	0.250	35.354
	rs2293576	A	G	-0.022	0.0036	9.40E-10	0.367	37.345
	rs10840100	G	A	0.02	0.0035	5.40E-09	0.725	32.653
	rs10767658	G	C	-0.031	0.0037	3.30E-17	0.642	70.197
	rs7138803	A	G	0.028	0.0035	1.60E-15	0.442	63.999
	rs12429545	A	G	0.031	0.0052	2.50E-09	0.100	35.540
	rs7144011	T	G	0.033	0.0041	9.40E-16	0.275	64.782
	rs10132280	A	C	-0.022	0.0037	2.20E-09	0.333	35.354
	rs4776970	T	A	-0.02	0.0035	2.30E-08	0.342	32.653
	rs749671	A	G	-0.019	0.0035	3.20E-08	0.375	29.469
	rs1558902	A	T	0.074	0.0035	3.70E-101	0.450	447.017
	rs2531992	G	A	0.028	0.0048	3.00E-09	0.833	34.027
	rs7498665	G	A	0.034	0.0035	1.40E-22	0.358	94.367
	rs17066856	C	T	-0.037	0.006	9.00E-10	0.133	38.027
	rs6567160	C	T	0.048	0.004	2.60E-33	0.283	143.999
	rs7239883	A	G	-0.021	0.0035	2.30E-09	0.683	36.000
	rs2287019	T	C	-0.035	0.0046	1.70E-14	0.150	57.892
	rs16996700	C	T	-0.023	0.0037	1.50E-09	0.300	38.641
WHR (GIANT and UKB[a])	rs113212351	T	C	0.012	0.002	4.11E-08	0.641	29.752
	rs2993481	A	T	-0.015	0.002	1.32E-10	0.668	39.745
	rs1891216	T	G	-0.010	0.002	2.03E-08	0.631	33.383
	rs6688233	T	C	0.019	0.002	3.02E-19	0.239	78.449
	rs12024554	T	C	-0.015	0.002	1.08E-10	0.235	41.968
	rs2298632	T	C	-0.014	0.002	3.49E-17	0.486	71.751
	rs11247735	A	G	0.011	0.002	7.14E-09	0.483	32.911
	rs4660808	T	C	0.015	0.002	7.58E-12	0.223	47.676

rs115092994	A	G	0.028	0.005	3.62E-08	0.961	29.713
rs7531656	A	G	0.016	0.002	1.41E-19	0.330	82.003
rs587271	T	C	0.011	0.002	3.18E-08	0.690	31.125
rs2481665	T	C	0.011	0.002	3.78E-11	0.582	43.405
rs2815749	A	G	-0.017	0.002	6.37E-15	0.185	62.485
rs380654	C	G	-0.012	0.002	1.22E-11	0.597	45.938
rs2802536	T	C	0.015	0.002	7.79E-11	0.181	43.102
rs2061708	C	G	0.015	0.002	2.13E-14	0.588	57.760
rs12033257	A	G	0.014	0.002	5.02E-13	0.630	51.235
rs3789615	T	C	-0.013	0.002	5.81E-15	0.428	61.207
rs10923724	T	C	0.024	0.002	1.81E-45	0.557	199.307
rs905938	T	C	0.013	0.002	7.83E-11	0.724	43.978
rs10919388	A	C	-0.027	0.002	6.40E-43	0.273	195.999
rs12125882	A	T	0.011	0.002	1.46E-09	0.476	36.670
rs9425301	A	C	0.018	0.002	2.41E-25	0.360	102.234
rs7528123	T	C	-0.010	0.002	1.42E-08	0.618	32.744
rs12069549	T	C	-0.016	0.003	3.58E-10	0.133	39.942
rs12048743	C	G	-0.013	0.002	2.31E-13	0.550	52.160
rs2791558	T	C	0.013	0.002	4.06E-10	0.262	40.960
rs1563355	T	C	-0.028	0.002	1.59E-49	0.328	212.545
rs10158345	C	G	0.012	0.002	1.57E-09	0.699	37.920
rs12042959	A	G	0.016	0.003	9.25E-10	0.863	38.440
rs77165542	T	C	-0.042	0.006	1.61E-14	0.034	58.036
rs6743060	A	C	0.027	0.002	1.80E-32	0.842	146.190
rs1815892	T	G	0.011	0.002	3.90E-09	0.581	34.028
rs711869	A	G	-0.017	0.002	2.38E-21	0.557	90.250
rs6749646	A	T	-0.021	0.002	5.99E-22	0.780	91.115
rs7587522	C	G	-0.011	0.002	5.49E-09	0.346	35.336
rs7591387	T	C	0.018	0.003	9.30E-11	0.102	40.869
rs17326656	T	G	0.015	0.002	4.17E-13	0.235	53.081
rs6719428	T	C	-0.017	0.002	3.76E-18	0.705	72.250
rs10865355	A	G	0.013	0.002	6.61E-15	0.409	61.207
rs12470785	A	G	-0.011	0.002	9.10E-09	0.700	34.130
rs7598832	T	C	-0.019	0.002	1.22E-26	0.333	110.250
rs7594568	A	G	-0.011	0.002	1.47E-08	0.646	31.125
rs1202	T	G	0.025	0.004	6.10E-09	0.610	33.802
rs4851283	C	G	0.016	0.002	1.54E-14	0.307	60.988
rs1345203	T	C	0.019	0.002	3.07E-15	0.783	60.710
rs4372913	A	G	-0.014	0.002	8.60E-11	0.788	40.496
rs332105	A	G	-0.014	0.002	1.12E-14	0.554	59.633
rs6430168	T	G	0.012	0.002	1.38E-08	0.209	31.258

rs55920843	T	G	0.062	0.009	1.55E-11	0.989	45.856
rs1563575	A	G	0.012	0.002	8.55E-10	0.712	37.274
rs10195252	T	C	0.023	0.002	2.86E-41	0.584	179.875
rs3754963	A	T	0.011	0.002	2.13E-08	0.762	31.922
rs6433219	A	G	0.014	0.002	2.25E-11	0.266	46.240
rs1569135	A	G	0.022	0.002	2.76E-37	0.537	159.948
rs1124639	T	C	-0.011	0.002	7.48E-11	0.439	44.183
rs4673616	T	G	-0.012	0.002	2.70E-09	0.637	36.602
rs7599312	A	G	-0.011	0.002	2.36E-09	0.273	36.000
rs1017698	A	G	-0.011	0.002	5.13E-10	0.597	39.410
rs4283409	A	G	-0.010	0.002	2.34E-08	0.406	32.111
rs17324331	C	G	0.012	0.002	1.79E-10	0.314	39.889
rs3891424	A	G	-0.028	0.004	4.00E-10	0.053	39.919
rs9872031	A	G	0.020	0.002	3.57E-29	0.429	125.938
rs11718898	T	C	0.014	0.002	2.17E-13	0.323	51.992
rs924814	A	G	0.011	0.002	6.05E-11	0.368	44.969
rs4635727	A	G	0.012	0.002	2.35E-09	0.723	34.810
rs10490869	A	T	-0.018	0.002	9.34E-17	0.796	69.192
rs525731	A	G	0.013	0.002	7.84E-13	0.589	51.361
rs13316065	T	C	0.017	0.002	1.34E-20	0.319	89.197
rs11711934	T	C	0.018	0.002	2.64E-15	0.824	63.275
rs17289049	A	G	-0.016	0.003	3.06E-08	0.911	29.884
rs1452075	T	C	0.012	0.002	1.32E-10	0.732	41.908
rs2371767	C	G	-0.029	0.002	1.00E-54	0.270	234.573
rs7631052	A	C	0.011	0.002	2.45E-08	0.332	30.540
rs6785245	T	C	-0.011	0.002	7.21E-10	0.607	38.716
rs12495178	T	C	0.012	0.002	1.32E-12	0.642	47.457
rs1609906	A	G	-0.013	0.002	1.22E-10	0.510	39.690
rs793456	A	G	-0.011	0.002	3.56E-09	0.605	34.679
rs16853606	A	G	-0.013	0.002	1.22E-08	0.841	32.938
rs6795831	A	C	0.029	0.002	2.09E-37	0.814	156.793
rs12489576	T	C	0.042	0.007	6.34E-09	0.019	33.576
rs13063979	T	G	-0.014	0.002	1.19E-12	0.732	50.485
rs645040	T	G	0.016	0.002	1.15E-14	0.770	60.840
rs10049088	T	C	-0.027	0.002	1.47E-53	0.381	226.669
rs998749	A	G	0.013	0.002	2.16E-14	0.502	58.477
rs4894803	A	G	0.015	0.002	3.74E-16	0.600	68.521
rs11705729	A	T	-0.011	0.002	1.70E-08	0.679	31.125
rs7647305	T	C	-0.014	0.002	8.28E-12	0.210	45.723
rs11724804	A	G	-0.017	0.002	4.98E-20	0.444	84.028
rs113928896	T	C	-0.018	0.003	1.25E-09	0.132	38.099

rs4586926	A	C	0.011	0.002	1.00E-08	0.636	34.679
rs10019888	A	G	-0.021	0.002	8.05E-20	0.813	81.784
rs17644283	A	G	0.015	0.002	3.24E-17	0.388	67.605
rs13130484	T	C	0.015	0.002	7.83E-18	0.429	74.771
rs11133377	A	G	0.014	0.002	8.92E-13	0.724	51.122
rs12643960	A	G	-0.017	0.003	1.51E-09	0.850	37.346
rs2167750	T	C	0.020	0.002	6.47E-29	0.473	122.225
rs1789882	A	G	0.016	0.002	9.97E-12	0.169	48.393
rs3804381	A	G	0.012	0.002	5.00E-10	0.275	38.440
rs7680787	T	C	0.010	0.002	4.40E-08	0.648	29.040
rs414865	A	T	0.011	0.002	1.19E-08	0.680	32.911
rs809955	A	G	-0.016	0.002	2.48E-14	0.367	60.062
rs789351	T	C	0.012	0.002	7.57E-13	0.420	52.349
rs1464454	A	G	-0.010	0.002	4.15E-08	0.396	29.040
rs2333496	T	C	0.010	0.002	1.08E-08	0.686	33.383
rs10475249	C	G	0.013	0.002	1.29E-11	0.549	44.890
rs6898870	A	G	-0.016	0.002	1.03E-14	0.261	60.840
rs459193	A	G	0.024	0.002	1.07E-35	0.250	158.229
rs142989338	T	C	-0.037	0.007	4.30E-08	0.977	29.767
rs1896686	A	G	-0.010	0.002	3.12E-08	0.494	30.250
rs2112347	T	G	0.015	0.002	8.41E-18	0.630	77.854
rs6870983	T	C	-0.016	0.002	2.08E-14	0.209	60.062
rs2161228	T	C	0.019	0.003	4.32E-11	0.102	43.833
rs2161097	T	C	0.013	0.002	2.11E-12	0.441	49.000
rs4395620	T	C	0.011	0.002	3.57E-09	0.576	34.679
rs11956399	T	G	0.016	0.002	1.26E-12	0.193	51.578
rs6874848	A	T	-0.014	0.002	1.75E-12	0.700	47.610
rs17764730	T	C	-0.011	0.002	4.86E-08	0.236	29.469
rs11747001	A	G	0.015	0.002	8.39E-15	0.764	59.290
rs2964006	T	G	0.013	0.002	3.57E-11	0.457	42.250
rs4454042	T	C	0.013	0.002	1.37E-10	0.273	40.322
rs4868256	T	C	0.011	0.002	8.66E-09	0.501	32.310
rs6861681	A	G	0.018	0.002	1.38E-22	0.292	100.000
rs244722	A	C	-0.013	0.002	5.08E-11	0.497	42.250
rs1294410	T	C	-0.025	0.002	5.49E-46	0.382	211.103
rs2714341	T	G	-0.011	0.002	6.43E-10	0.548	38.149
rs6932767	T	G	0.016	0.002	2.20E-12	0.793	49.638
rs7744833	A	G	0.013	0.002	6.88E-11	0.682	43.282
rs2524137	T	C	0.023	0.002	8.66E-29	0.640	129.960
rs117108573	T	C	0.027	0.004	3.90E-12	0.076	49.000
rs28417075	A	T	-0.025	0.004	2.18E-11	0.918	44.327

rs575662827	T	C	-0.029	0.004	2.96E-16	0.902	66.241
rs147627829	A	G	0.061	0.005	1.87E-35	0.042	155.994
rs7754297	A	G	0.014	0.002	3.38E-09	0.234	34.964
rs998584	A	C	0.035	0.002	7.42E-92	0.480	426.300
rs4714704	A	G	-0.017	0.002	1.13E-16	0.274	69.722
rs987237	A	G	-0.020	0.002	9.98E-20	0.844	82.644
rs12527712	T	C	0.028	0.003	3.38E-18	0.088	74.064
rs10499013	A	G	-0.013	0.002	5.10E-10	0.280	39.062
rs901630	T	C	-0.012	0.002	6.81E-12	0.411	49.000
rs6941962	A	T	0.024	0.003	5.28E-20	0.156	83.090
rs2022464	A	C	-0.013	0.002	1.67E-11	0.306	46.097
rs377436	A	G	-0.011	0.002	1.78E-08	0.293	32.911
rs17078048	A	G	-0.014	0.003	3.90E-08	0.850	29.594
rs11752928	C	G	-0.011	0.002	1.69E-08	0.487	30.540
rs11154378	A	T	0.019	0.002	2.20E-27	0.499	114.966
rs72959041	A	G	0.126	0.004	4.56E-183	0.041	820.038
rs62424543	A	G	-0.022	0.004	2.22E-08	0.930	32.012
rs605066	T	C	-0.019	0.002	2.38E-26	0.583	113.777
rs7453812	T	G	-0.024	0.004	3.33E-09	0.048	35.106
rs668871	T	C	-0.013	0.002	7.19E-12	0.469	44.890
rs852425	A	G	-0.010	0.002	8.40E-09	0.650	32.111
rs2391168	A	C	0.026	0.002	1.38E-34	0.195	153.288
rs1534696	A	C	-0.023	0.002	3.67E-39	0.569	176.733
rs860262	A	C	-0.012	0.002	1.54E-09	0.490	34.810
rs2715135	T	G	0.011	0.002	4.09E-09	0.371	36.670
rs1718618	A	G	-0.024	0.004	2.19E-10	0.064	39.889
rs4718966	T	C	0.010	0.002	1.69E-08	0.412	32.111
rs55747707	A	G	-0.015	0.002	3.40E-11	0.177	44.832
rs12669521	A	G	0.013	0.002	1.29E-09	0.673	36.574
rs13232789	T	G	-0.011	0.002	5.09E-09	0.661	33.518
rs11976018	A	G	-0.015	0.002	3.69E-11	0.171	43.102
rs1142	T	C	0.015	0.002	2.06E-17	0.334	68.521
rs39312	A	C	-0.015	0.002	3.50E-18	0.621	73.197
rs6942652	C	G	-0.013	0.002	2.18E-12	0.429	49.000
rs13229637	T	C	0.016	0.003	1.91E-10	0.854	40.449
rs2069443	T	G	-0.012	0.002	1.40E-08	0.742	32.111
rs9969455	A	G	0.011	0.002	7.10E-10	0.616	38.028
rs15285	T	C	-0.012	0.002	1.62E-09	0.263	37.920
rs9644033	A	T	0.019	0.002	2.10E-19	0.755	80.145
rs11992444	T	G	0.019	0.002	4.99E-21	0.509	85.562
rs11574218	T	G	0.014	0.002	4.36E-14	0.387	58.778

rs881301	T	C	-0.013	0.002	1.18E-13	0.584	55.420
rs62506196	A	C	-0.016	0.003	2.70E-09	0.838	34.679
rs10504486	A	G	0.018	0.003	3.06E-08	0.923	29.752
rs4738141	A	G	-0.020	0.002	7.34E-25	0.744	110.803
rs1431659	A	G	0.014	0.002	1.12E-12	0.274	51.122
rs4145698	A	T	0.011	0.002	7.71E-09	0.658	32.310
rs35708461	T	C	0.014	0.002	1.73E-08	0.215	31.640
rs13256367	A	C	0.014	0.002	5.42E-14	0.653	55.072
rs11782074	T	G	0.011	0.002	6.32E-09	0.368	32.911
rs7861458	A	C	0.013	0.002	3.55E-08	0.769	30.972
rs6474945	T	G	-0.010	0.002	2.67E-09	0.454	35.297
rs10968576	A	G	-0.014	0.002	2.25E-15	0.691	64.000
rs1680490	A	G	-0.012	0.002	2.41E-09	0.595	34.810
rs10797116	T	C	-0.010	0.002	2.05E-08	0.465	31.484
rs753804	C	G	0.017	0.002	2.06E-13	0.175	55.924
rs10761254	T	C	0.012	0.002	3.20E-11	0.344	42.593
rs2398893	A	G	0.016	0.002	9.01E-17	0.714	68.280
rs10991433	T	C	-0.025	0.003	5.73E-20	0.896	82.291
rs111874795	T	C	0.038	0.005	7.00E-16	0.956	64.000
rs10817158	A	C	0.014	0.002	4.08E-13	0.316	52.753
rs4837261	T	C	0.013	0.002	1.45E-08	0.179	31.457
rs2799465	T	C	-0.015	0.003	3.07E-09	0.848	34.574
rs4382592	T	G	0.012	0.002	2.71E-10	0.306	39.889
rs28647893	T	C	-0.012	0.002	2.91E-09	0.482	33.640
rs11792069	A	G	0.014	0.002	2.43E-08	0.827	32.111
rs10795055	A	G	0.011	0.002	5.43E-09	0.393	34.679
rs7907173	A	G	-0.011	0.002	9.43E-10	0.451	36.670
rs1243188	T	C	-0.015	0.002	2.37E-14	0.706	60.676
rs2808104	T	C	-0.012	0.002	2.29E-09	0.224	34.866
rs145952040	T	C	-0.033	0.005	2.66E-10	0.961	40.274
rs10827252	A	G	-0.011	0.002	2.03E-10	0.493	39.410
rs1757471	T	C	0.013	0.002	5.89E-13	0.508	50.568
rs2696309	T	C	-0.011	0.002	4.11E-08	0.277	29.160
rs7070670	T	C	-0.013	0.002	3.64E-10	0.323	39.510
rs7919055	T	C	-0.028	0.005	4.81E-10	0.958	39.271
rs10761785	T	G	-0.015	0.002	6.43E-18	0.514	74.771
rs7898903	T	C	-0.015	0.003	3.68E-08	0.132	30.047
rs780159	A	G	-0.013	0.002	2.86E-14	0.429	61.207
rs1250552	A	G	-0.009	0.002	3.62E-08	0.520	30.574
rs10887759	A	G	0.016	0.002	2.32E-11	0.160	45.002
rs1437	A	G	0.013	0.002	4.50E-13	0.629	50.568

rs11187537	C	G	0.013	0.002	1.73E-10	0.263	42.250
rs4075353	A	G	-0.012	0.002	9.32E-09	0.345	32.111
rs12777288	T	C	-0.014	0.002	6.88E-11	0.771	43.812
rs2254069	A	G	0.021	0.003	4.77E-15	0.128	59.919
rs61876729	A	G	0.021	0.003	5.55E-10	0.909	39.616
rs12575252	C	G	-0.015	0.002	2.32E-18	0.334	73.197
rs12419064	A	G	-0.011	0.002	5.77E-10	0.456	40.111
rs747601	A	T	-0.013	0.002	7.27E-11	0.275	40.960
rs11029441	T	C	-0.020	0.004	1.73E-08	0.916	32.327
rs11030107	A	G	-0.018	0.002	4.08E-20	0.733	87.767
rs13642	A	T	0.011	0.002	9.49E-10	0.645	35.371
rs67184556	T	C	-0.012	0.002	4.93E-08	0.322	31.041
rs2176598	T	C	0.013	0.002	5.51E-11	0.236	45.385
rs12287076	C	G	0.019	0.002	3.53E-21	0.625	92.160
rs2509963	T	C	-0.014	0.002	2.81E-12	0.261	51.122
rs35169799	T	C	0.037	0.004	6.26E-20	0.062	82.765
rs72940579	T	C	-0.012	0.002	3.53E-09	0.331	34.866
rs7395513	A	G	-0.017	0.002	2.46E-18	0.439	74.822
rs536665	A	G	-0.017	0.002	2.18E-12	0.798	50.765
rs647248	A	G	0.011	0.002	6.49E-09	0.541	34.028
rs1488541	T	C	-0.013	0.002	5.02E-09	0.277	33.851
rs2276390	T	G	-0.021	0.002	5.76E-28	0.338	122.160
rs11214589	A	G	-0.011	0.002	1.77E-10	0.496	40.111
rs3825061	T	C	0.014	0.002	5.45E-15	0.387	62.234
rs579682	T	C	-0.013	0.002	2.02E-11	0.714	44.222
rs747249	A	G	0.011	0.002	2.65E-09	0.359	34.130
rs11223224	C	G	-0.010	0.002	3.72E-08	0.477	29.040
rs6590683	T	C	0.011	0.002	3.29E-10	0.479	39.410
rs12828016	T	G	-0.011	0.002	4.08E-10	0.391	40.111
rs7222	T	C	0.011	0.002	1.17E-09	0.499	36.670
rs11055887	A	G	-0.013	0.002	4.28E-09	0.193	33.438
rs11048456	T	C	-0.027	0.002	3.00E-43	0.765	195.999
rs10506110	A	C	0.014	0.002	1.31E-13	0.635	52.753
rs1026462	A	G	-0.013	0.002	1.52E-12	0.515	49.000
rs55677077	T	C	-0.010	0.002	2.77E-08	0.453	32.111
rs1443512	A	C	0.028	0.002	1.06E-45	0.222	198.809
rs35643724	A	G	-0.025	0.003	2.88E-19	0.144	81.000
rs11176015	T	C	0.015	0.002	7.21E-14	0.293	57.002
rs317646	A	G	-0.011	0.002	2.56E-08	0.731	30.250
rs10745659	C	G	-0.013	0.002	1.43E-12	0.561	49.781
rs7311622	T	C	-0.012	0.002	2.96E-11	0.453	43.707

rs3764002	T	C	-0.021	0.002	3.91E-27	0.264	111.302
rs148589464	T	C	0.021	0.004	4.43E-08	0.069	29.828
rs74628422	A	G	0.014	0.002	9.10E-09	0.204	34.515
rs1969354	T	C	-0.017	0.002	2.52E-15	0.222	61.124
rs2178663	T	C	-0.026	0.002	2.79E-43	0.320	191.603
rs863750	T	C	0.026	0.002	6.20E-51	0.584	232.114
rs1360485	T	C	0.015	0.002	2.01E-16	0.679	71.308
rs10507524	T	C	-0.017	0.003	1.79E-08	0.905	32.490
rs797486	A	C	0.032	0.003	1.09E-34	0.894	147.716
rs7982447	T	C	-0.013	0.002	6.07E-09	0.802	33.851
rs1056114	A	G	-0.010	0.002	1.30E-08	0.527	32.111
rs1441264	A	G	0.011	0.002	3.16E-10	0.586	38.028
rs7350648	T	G	0.014	0.003	1.99E-08	0.801	30.470
rs12430764	A	G	0.011	0.002	1.87E-09	0.516	35.336
rs9556979	T	G	-0.011	0.002	6.63E-09	0.685	35.371
rs9515201	A	C	-0.012	0.002	6.63E-11	0.307	45.188
rs4773395	A	G	0.011	0.002	3.42E-09	0.458	34.679
rs10144278	A	G	0.010	0.002	3.10E-08	0.434	31.484
rs10132280	A	C	-0.012	0.002	3.11E-10	0.311	41.531
rs61986159	T	C	0.014	0.002	4.85E-09	0.231	34.964
rs1190982	T	C	0.016	0.002	5.10E-16	0.303	68.280
rs4902632	A	T	0.016	0.002	1.78E-10	0.178	41.710
rs2526886	T	G	0.013	0.002	3.65E-09	0.690	35.431
rs7101	T	C	-0.011	0.002	1.97E-08	0.753	30.802
rs17109256	A	G	0.017	0.002	2.40E-16	0.232	64.764
rs7492628	C	G	-0.013	0.002	7.89E-11	0.680	43.282
rs7143963	T	C	0.014	0.002	3.31E-10	0.169	38.215
rs4779526	A	T	0.013	0.002	2.09E-10	0.757	39.510
rs12440605	A	G	0.012	0.002	4.02E-11	0.524	42.975
rs11630842	A	G	-0.011	0.002	6.37E-10	0.510	38.028
rs12595496	A	G	-0.018	0.003	2.08E-12	0.866	50.694
rs1657930	A	G	-0.013	0.002	5.62E-10	0.793	37.099
rs8043060	A	G	-0.017	0.002	8.93E-17	0.222	68.890
rs876383	A	G	-0.013	0.002	2.13E-13	0.451	53.778
rs1992145	A	G	0.011	0.002	5.77E-10	0.551	38.028
rs12593088	A	G	-0.013	0.002	5.16E-12	0.311	45.385
rs12101393	C	G	0.014	0.002	1.19E-10	0.787	40.496
rs2061007	C	G	-0.011	0.002	4.65E-09	0.588	34.028
rs71385734	T	G	0.019	0.003	1.06E-13	0.827	54.532
rs220381	A	G	-0.011	0.002	2.79E-08	0.673	31.125
rs3747579	T	C	-0.015	0.002	4.07E-16	0.711	69.444

rs35154326	A	G	0.014	0.002	4.85E-10	0.722	39.347
rs2008514	A	G	0.017	0.002	8.56E-23	0.384	100.000
rs4788204	A	G	0.017	0.002	2.64E-21	0.459	88.151
rs8054299	C	G	0.011	0.002	2.46E-08	0.686	31.715
rs1558902	A	T	0.040	0.002	3.99E-118	0.409	548.109
rs889398	T	C	-0.017	0.002	1.99E-23	0.415	101.180
rs4243130	T	G	-0.012	0.002	1.68E-08	0.808	33.199
rs2925979	T	C	0.022	0.002	1.62E-31	0.296	142.669
rs7206608	C	G	-0.013	0.002	2.48E-11	0.675	44.679
rs7217226	T	G	-0.014	0.002	9.94E-15	0.656	57.929
rs8070737	T	G	0.016	0.002	7.39E-12	0.168	48.393
rs7209595	C	G	0.014	0.002	2.35E-14	0.453	57.086
rs7213608	T	C	-0.017	0.002	6.95E-19	0.683	81.000
rs868340	A	T	0.012	0.002	6.34E-11	0.528	42.250
rs591939	A	G	-0.018	0.002	9.11E-15	0.755	59.223
rs62063286	T	C	0.024	0.002	2.49E-23	0.781	101.673
rs8071778	C	G	-0.016	0.003	6.62E-11	0.154	40.960
rs11654395	T	C	-0.018	0.003	3.75E-08	0.919	30.083
rs2727331	T	G	0.013	0.002	1.35E-12	0.323	49.000
rs12602912	T	C	0.018	0.002	1.23E-16	0.211	70.240
rs11654387	C	G	0.016	0.002	1.45E-19	0.488	80.003
rs4239275	T	C	0.012	0.002	3.26E-11	0.413	42.975
rs11664106	A	T	0.020	0.002	8.69E-22	0.630	90.703
rs693301	T	C	0.011	0.002	4.02E-09	0.441	34.028
rs62095889	A	G	-0.017	0.002	1.62E-15	0.343	62.485
rs10164099	T	C	-0.015	0.003	1.17E-09	0.874	35.521
rs1158805	A	C	-0.013	0.002	4.95E-13	0.380	54.596
rs9951872	A	G	0.021	0.002	1.24E-17	0.160	73.673
rs6567160	T	C	-0.026	0.002	2.53E-39	0.753	172.922
rs17066856	T	C	0.018	0.003	3.79E-10	0.892	39.960
rs1942831	C	G	0.011	0.002	2.93E-08	0.297	32.310
rs2981423	T	C	0.019	0.004	3.64E-08	0.938	30.723
rs12459350	A	G	0.013	0.002	2.23E-15	0.542	62.131
rs1035942	A	G	0.012	0.002	1.50E-09	0.275	36.602
rs12608504	A	G	0.025	0.002	4.68E-46	0.359	194.447
rs17724992	A	G	0.014	0.002	1.29E-13	0.721	56.645
rs60570301	A	G	-0.017	0.003	1.48E-08	0.118	31.505
rs998732	A	G	0.017	0.002	1.04E-12	0.834	51.465
rs7250389	T	G	0.013	0.002	1.33E-11	0.307	46.814
rs3786897	A	G	-0.024	0.002	1.45E-43	0.581	194.356
rs429358	T	C	0.035	0.003	4.21E-37	0.846	164.219

	rs1800437	C	G	-0.021	0.002	6.23E-22	0.188	90.250
	rs11084399	T	C	-0.011	0.002	1.81E-09	0.441	35.371
	rs805770	T	C	0.018	0.002	3.42E-22	0.399	97.790
	rs979012	T	C	0.012	0.002	7.86E-11	0.364	41.531
	rs11699316	A	G	0.014	0.003	3.75E-08	0.143	30.250
	rs143384	A	G	0.016	0.002	1.73E-19	0.587	83.131
	rs6130360	A	G	0.015	0.003	1.13E-09	0.849	36.481
	rs2236519	A	G	0.021	0.002	1.89E-30	0.369	138.716
	rs3092781	T	C	-0.015	0.002	1.92E-17	0.451	72.751
	rs6021889	A	G	0.020	0.002	2.46E-27	0.710	114.152
	rs62206548	T	G	-0.017	0.003	5.05E-11	0.173	43.256
	rs1328757	T	C	0.011	0.002	1.69E-10	0.474	40.111
	rs2823096	A	G	0.013	0.002	3.17E-08	0.812	30.972
	rs28451064	A	G	0.018	0.003	4.23E-09	0.127	34.810
	rs2836179	A	G	-0.013	0.002	2.44E-13	0.414	53.778
	rs2838006	T	C	-0.011	0.002	4.97E-09	0.356	32.911
	rs2294239	A	G	0.020	0.002	3.17E-31	0.568	138.408
	rs2235148	T	C	0.012	0.002	9.23E-11	0.362	43.707
	rs34848977	A	G	0.012	0.002	2.85E-08	0.295	32.111
	rs8141715	T	G	-0.014	0.002	1.45E-11	0.755	46.240
WHR (GIANT)	rs1106529	A	G	0.028	0.0038	2.20E-13	0.725	54.293
	rs1563355	C	T	0.031	0.0044	1.70E-12	0.686	49.638
	rs714515	A	G	-0.019	0.0033	5.90E-09	0.542	33.149
	rs6743060	A	C	0.025	0.0043	1.50E-08	0.875	33.802
	rs6736025	T	G	-0.024	0.0042	7.20E-09	0.408	32.653
	rs1128249	T	G	-0.02	0.0033	7.80E-10	0.442	36.731
	rs13424740	C	T	-0.024	0.0032	2.60E-13	0.467	56.250
	rs17451107	C	T	-0.021	0.0034	1.20E-09	0.375	38.148
	rs2972164	C	T	0.019	0.0033	1.00E-08	0.500	33.149
	rs2371767	C	G	-0.024	0.0037	1.20E-10	0.208	42.074
	rs13130484	T	C	0.019	0.0033	1.50E-08	0.433	33.149
	rs459193	G	A	-0.027	0.0037	3.40E-13	0.783	53.250
	rs1294410	C	T	0.025	0.0033	2.00E-14	0.625	57.392
	rs998584	A	C	0.029	0.0036	4.80E-15	0.475	64.891
	rs9491696	G	C	0.037	0.0032	1.40E-30	0.525	133.690
	rs10245353	A	C	0.027	0.0041	3.50E-11	0.183	43.367
	rs12549058	G	T	0.037	0.006	8.30E-10	0.058	38.027
	rs2398893	G	A	-0.02	0.0036	4.00E-08	0.317	30.864
	rs4929927	G	A	0.019	0.0033	1.60E-08	0.725	33.149
	rs879048	A	C	0.022	0.004	3.60E-08	0.800	30.250
	rs10783615	A	G	-0.034	0.0046	3.70E-13	0.867	54.631

rs10842708	A	G	-0.024	0.0036	3.10E-11	0.817	44.444
rs7973683	A	C	-0.02	0.0034	3.30E-09	0.383	34.602
rs1440372	C	T	0.022	0.0036	2.70E-09	0.742	37.345
rs1421085	C	T	0.043	0.0033	4.30E-38	0.450	169.787
rs4640244	G	A	0.02	0.0037	3.00E-08	0.375	29.218
rs11663816	C	T	0.026	0.0037	3.10E-12	0.317	49.379
rs3786897	G	A	0.022	0.0033	2.50E-11	0.408	44.444
rs2287019	T	C	-0.025	0.0044	1.10E-08	0.150	32.283
rs16996700	C	T	-0.021	0.0036	8.80E-09	0.300	34.027
rs4823006	G	A	-0.019	0.0033	3.30E-09	0.467	33.149

β represents the change in an obesity-related trait (in standard deviation unit) per additional copy of the effect allele.

[a] The data were originated from a meta-analysis combining the GWAS data provided by GIANT and UKB.

Abbreviations: SNPs, single nucleotide polymorphisms; SE, standard error; EAF, effect allele frequency; GIANT, Genetic Investigation of ANthropometric Traits; UKB, UK Biobank.

Table S3. Characteristics of the genetic instrument variables for the potential mediators at the genome-wide significance level.

Potential mediators	SNPs	Effect allele	Other allele	*β*	SE	*P*	EAF	*F*
FEV1	rs1392140	A	G	-0.015	0.003	4.28E-09	0.726	35.083
	rs35750964	C	T	0.020	0.003	1.87E-13	0.244	54.869
	rs944190	T	G	-0.016	0.002	5.71E-11	0.591	42.250
	rs1008833	G	A	0.029	0.003	4.55E-18	0.146	75.637
	rs1106370	A	G	0.014	0.002	4.33E-09	0.421	33.062
	rs12737805	G	A	-0.020	0.003	6.57E-13	0.221	52.562
	rs12096239	C	G	-0.019	0.003	2.08E-12	0.253	47.968
	rs6697620	G	A	-0.023	0.002	1.10E-21	0.566	88.673
	rs12403980	C	G	-0.020	0.002	6.11E-18	0.486	77.134
	rs17395720	C	T	-0.016	0.003	5.35E-09	0.232	33.062
	rs2885697	T	G	-0.014	0.002	2.26E-08	0.666	32.585
	rs6692738	A	G	0.019	0.003	8.67E-13	0.285	50.628
	rs72737267	T	C	0.021	0.004	3.27E-09	0.123	34.352
	rs1416685	C	G	0.016	0.002	6.12E-12	0.405	45.562
	rs16859007	A	G	0.018	0.003	4.64E-08	0.152	29.094
	rs7517716	C	A	0.017	0.002	2.09E-13	0.532	55.924
	rs16824937	A	G	-0.034	0.005	5.81E-14	0.070	57.422
	rs755249	T	C	-0.020	0.003	1.52E-13	0.233	55.972
	rs1968027	C	T	0.018	0.003	1.57E-10	0.791	40.257
	rs9970286	A	G	0.017	0.003	5.91E-12	0.329	47.334
	rs6425501	G	A	0.018	0.002	1.04E-13	0.352	57.507
	rs7516060	A	G	0.014	0.002	1.99E-08	0.636	33.062
	rs6697759	C	T	0.013	0.002	2.60E-08	0.561	30.710
	rs2808218	C	T	0.021	0.003	1.09E-16	0.322	69.222
	rs2562008	G	A	-0.013	0.002	3.90E-08	0.466	29.793
	rs3795948	C	T	0.016	0.002	1.10E-11	0.482	46.595
	rs2544535	C	T	0.022	0.002	1.08E-20	0.512	88.196
	rs11125611	T	A	-0.019	0.002	1.06E-14	0.372	60.710
	rs72902177	T	C	-0.034	0.003	1.76E-22	0.135	97.080
	rs2304340	G	A	0.014	0.002	3.72E-09	0.592	34.028
	rs13036142	T	C	0.021	0.003	1.21E-12	0.197	50.950
	rs10490551	A	T	0.019	0.003	1.45E-13	0.277	53.966
	rs72894568	G	A	0.043	0.008	2.23E-08	0.025	31.566
	rs7424771	A	G	-0.017	0.002	6.57E-13	0.447	49.585
	rs1249096	A	G	0.018	0.002	2.18E-14	0.563	56.876
	rs62191107	C	T	0.029	0.003	1.65E-22	0.199	97.260
	rs12695002	T	C	-0.015	0.002	1.15E-09	0.589	37.007
	rs62126364	A	G	0.030	0.003	3.76E-27	0.219	116.331
	rs4952571	G	C	0.016	0.003	4.94E-10	0.322	38.440

rs17033254	C	T	0.018	0.003	2.58E-09	0.198	36.415
rs62147658	A	G	-0.025	0.004	2.42E-12	0.126	49.803
rs1530555	T	C	0.019	0.002	1.31E-14	0.374	60.062
rs10193804	G	C	0.014	0.003	1.91E-08	0.652	30.470
rs1840168	T	C	0.017	0.003	3.16E-10	0.247	39.643
rs72995681	G	T	-0.017	0.002	5.20E-12	0.371	48.418
rs13008330	T	C	-0.021	0.004	1.09E-09	0.131	36.689
rs2571445	G	A	0.029	0.002	7.24E-33	0.603	141.015
rs10929647	A	C	-0.013	0.002	2.98E-08	0.513	31.457
rs6723572	C	T	0.026	0.003	1.46E-17	0.182	72.817
rs6740092	A	T	0.023	0.003	1.07E-11	0.855	47.317
rs1436163	A	G	0.019	0.004	2.86E-08	0.132	30.093
rs13069228	C	T	-0.016	0.002	9.59E-11	0.361	43.340
rs6807089	C	T	-0.013	0.002	2.86E-08	0.464	29.793
rs4955548	C	A	-0.016	0.003	2.01E-10	0.668	40.449
rs881755	C	T	0.025	0.002	9.06E-26	0.516	114.396
rs78101726	G	A	-0.033	0.003	7.72E-25	0.154	108.941
rs12107414	C	G	-0.018	0.003	3.01E-09	0.189	35.204
rs3856802	T	C	0.014	0.002	2.97E-09	0.545	36.523
rs1286645	A	G	0.017	0.003	3.05E-08	0.182	30.988
rs9819463	C	T	-0.022	0.003	1.14E-14	0.204	59.662
rs6780171	A	T	-0.017	0.003	6.18E-12	0.313	48.441
rs13433809	G	A	-0.021	0.002	1.43E-18	0.439	80.219
rs6445932	G	T	0.029	0.003	3.82E-26	0.248	112.988
rs7639737	C	T	-0.033	0.003	4.18E-29	0.205	125.594
rs1610263	A	T	0.030	0.004	7.10E-13	0.912	52.474
rs2811416	C	T	-0.021	0.004	1.61E-08	0.883	32.744
rs10489880	G	A	0.016	0.003	2.73E-09	0.279	35.540
rs73154306	C	T	-0.025	0.003	2.27E-21	0.272	92.455
rs73182224	A	G	-0.018	0.003	6.00E-10	0.239	37.674
rs2974389	G	A	-0.017	0.002	1.70E-12	0.574	51.465
rs1352658	T	C	0.015	0.002	1.86E-10	0.575	40.111
rs1499894	T	C	0.020	0.002	1.02E-16	0.440	66.694
rs2153997	G	A	0.015	0.003	6.71E-09	0.297	34.574
rs62316310	A	G	0.018	0.003	4.53E-11	0.260	42.491
rs2125787	G	A	-0.013	0.002	4.26E-08	0.400	29.793
rs1909121	G	A	-0.028	0.004	2.10E-10	0.924	40.785
rs308381	A	G	0.033	0.006	3.12E-08	0.959	30.906
rs7663740	T	G	0.041	0.002	1.17E-66	0.544	310.064
rs59831071	A	G	-0.013	0.002	3.62E-08	0.468	29.340
rs10005540	T	C	0.014	0.002	3.08E-09	0.615	36.000

rs1054661	C	T	0.014	0.002	1.09E-08	0.347	33.543
rs34712979	A	G	-0.057	0.003	9.32E-97	0.256	440.997
rs57389113	A	G	-0.019	0.003	1.07E-09	0.170	37.961
rs4834214	A	G	-0.015	0.002	5.64E-10	0.552	37.007
rs3106209	C	A	-0.014	0.003	3.65E-08	0.671	30.470
rs4478172	C	A	-0.018	0.003	3.19E-12	0.261	50.083
rs12331869	G	A	0.018	0.003	3.17E-09	0.821	34.090
rs6830774	C	T	-0.015	0.002	6.76E-10	0.467	39.745
rs7700595	G	A	0.023	0.004	1.94E-10	0.124	39.760
rs11747434	C	T	0.016	0.003	2.76E-09	0.278	36.000
rs11739847	A	G	-0.021	0.003	4.30E-13	0.199	52.437
rs6450095	A	T	-0.016	0.003	4.30E-08	0.201	30.440
rs13183337	C	A	-0.017	0.003	7.89E-09	0.204	33.961
rs55747751	A	G	-0.025	0.004	1.37E-08	0.081	32.995
rs13361953	C	T	-0.026	0.003	2.02E-25	0.336	106.502
rs2545675	T	C	0.015	0.002	6.75E-11	0.521	40.640
rs4836246	A	G	-0.014	0.003	1.96E-08	0.339	30.913
rs425102	G	T	-0.016	0.003	6.55E-09	0.240	34.679
rs7733410	A	G	0.034	0.002	7.12E-47	0.441	203.061
rs4490572	A	G	-0.018	0.002	9.24E-15	0.463	58.777
rs979453	G	A	-0.019	0.003	3.60E-14	0.326	57.153
rs10059996	G	T	0.014	0.003	3.73E-08	0.645	30.913
rs268717	T	C	-0.031	0.004	1.67E-14	0.907	58.905
rs79904209	T	C	-0.032	0.004	1.02E-15	0.097	63.202
rs3212656	C	T	0.024	0.003	1.72E-17	0.211	70.791
rs11241689	T	C	-0.017	0.002	2.16E-13	0.439	51.960
rs72643433	A	G	-0.019	0.003	2.10E-12	0.251	49.520
rs12204336	A	G	-0.014	0.002	5.72E-09	0.374	34.028
rs1543310	C	T	0.016	0.002	1.53E-11	0.541	44.444
rs185774696	T	C	-0.033	0.003	4.28E-25	0.290	109.911
rs141127771	A	G	-0.027	0.003	6.95E-17	0.252	68.438
rs221615	G	A	0.017	0.003	1.15E-10	0.731	42.751
rs661857	C	T	0.015	0.002	3.08E-10	0.484	41.406
rs10498672	G	C	-0.031	0.003	1.90E-24	0.177	106.089
rs9262120	A	G	-0.042	0.003	8.95E-40	0.163	173.909
rs75104038	A	G	0.037	0.005	9.24E-14	0.060	56.097
rs2894837	G	A	-0.018	0.002	9.22E-13	0.360	53.168
rs9385988	G	A	0.028	0.003	1.40E-26	0.277	115.149
rs3253	T	C	0.016	0.003	1.28E-10	0.314	41.990
rs9350191	T	C	-0.024	0.003	4.68E-13	0.848	51.144
rs13216424	A	G	-0.021	0.003	2.13E-12	0.177	50.884

rs2806356	C	T	-0.032	0.003	1.09E-26	0.185	115.204
rs1102077	C	A	-0.022	0.003	4.21E-15	0.243	63.408
rs9392172	G	C	-0.022	0.002	1.40E-20	0.469	88.196
rs615850	T	G	0.015	0.003	2.67E-08	0.743	29.858
rs7753563	G	A	0.020	0.003	4.14E-13	0.768	51.532
rs6904757	G	A	-0.016	0.002	1.58E-10	0.366	42.793
rs17812980	T	C	0.014	0.002	7.55E-09	0.503	34.452
rs12212123	C	T	-0.026	0.004	9.42E-12	0.104	45.476
rs2723574	T	C	-0.014	0.002	1.92E-09	0.423	34.515
rs7806296	A	G	-0.019	0.002	4.19E-15	0.360	62.673
rs12700186	C	T	0.015	0.002	4.36E-10	0.417	37.515
rs6462481	T	C	-0.016	0.003	5.28E-09	0.231	33.062
rs12707691	G	C	0.021	0.003	1.67E-16	0.336	67.240
rs4726715	G	T	0.014	0.002	4.66E-09	0.521	33.062
rs12698403	A	G	-0.027	0.002	6.42E-31	0.442	130.339
rs11766096	T	A	0.019	0.002	1.28E-15	0.504	66.104
rs28719767	C	G	0.019	0.003	7.22E-13	0.288	50.628
rs4724960	G	A	-0.016	0.003	1.03E-10	0.337	40.449
rs849135	A	G	0.020	0.002	1.05E-17	0.500	74.109
rs4730073	A	C	0.014	0.002	1.05E-08	0.643	34.028
rs659398	C	T	0.018	0.003	4.35E-11	0.728	42.009
rs6470697	C	A	0.028	0.004	6.55E-13	0.099	52.655
rs55915240	A	T	-0.019	0.003	5.05E-10	0.186	39.271
rs12679101	C	T	-0.015	0.003	2.61E-08	0.253	30.864
rs7838717	C	T	0.022	0.003	1.83E-18	0.637	75.342
rs330925	A	G	-0.017	0.003	2.54E-11	0.309	43.256
rs4737183	G	A	0.015	0.002	3.21E-10	0.474	38.028
rs1441207	C	G	0.022	0.003	4.04E-16	0.260	64.594
rs4741893	C	G	-0.023	0.002	4.98E-22	0.415	91.043
rs201191	A	C	0.017	0.002	5.07E-13	0.486	53.353
rs7028528	C	A	0.015	0.003	2.39E-09	0.328	35.521
rs10125279	A	G	0.014	0.002	6.84E-09	0.384	33.543
rs2095024	A	G	0.017	0.002	2.27E-12	0.588	47.840
rs1360235	A	G	-0.015	0.002	3.87E-10	0.518	40.295
rs803909	G	T	-0.021	0.002	2.08E-19	0.541	78.027
rs61482805	G	C	-0.018	0.003	1.03E-12	0.330	50.694
rs750188	A	T	-0.014	0.002	8.67E-10	0.504	39.198
rs3118008	A	C	0.016	0.003	6.70E-10	0.277	38.822
rs72718111	C	A	0.016	0.003	2.85E-09	0.258	35.116
rs12115436	A	G	0.023	0.003	5.09E-13	0.167	53.148
rs10819770	A	T	-0.014	0.002	5.10E-09	0.494	35.480

rs10781518	G	A	-0.014	0.002	2.88E-09	0.451	34.028
rs211401	T	C	-0.015	0.002	1.81E-09	0.634	36.502
rs2579762	C	A	-0.029	0.002	6.20E-35	0.472	157.884
rs4918429	G	C	-0.015	0.002	7.40E-10	0.376	38.543
rs4748734	T	G	-0.014	0.003	2.61E-08	0.693	31.360
rs7092539	T	C	-0.022	0.003	1.79E-13	0.815	54.760
rs7098573	A	G	-0.025	0.003	2.75E-21	0.718	89.520
rs2489009	T	C	-0.013	0.002	1.85E-08	0.548	31.173
rs11191841	C	T	0.017	0.002	6.21E-13	0.509	53.353
rs12357270	G	C	0.014	0.002	1.99E-08	0.359	32.585
rs7906816	G	A	0.031	0.005	5.74E-10	0.057	38.937
rs2271804	A	G	0.034	0.002	4.74E-48	0.530	215.961
rs2393729	T	C	-0.014	0.002	4.78E-09	0.421	33.543
rs10997955	T	C	-0.018	0.002	1.20E-13	0.589	54.390
rs7116641	G	T	-0.019	0.003	1.21E-13	0.317	54.760
rs7949728	G	C	-0.015	0.002	1.70E-10	0.410	40.111
rs11234767	C	A	-0.024	0.003	1.53E-13	0.154	55.782
rs4936101	G	A	-0.014	0.002	7.79E-09	0.595	33.062
rs10838435	G	C	-0.021	0.003	1.46E-10	0.856	40.882
rs149333984	A	G	0.036	0.006	1.64E-09	0.041	36.000
rs2167356	G	C	0.013	0.002	3.12E-08	0.421	29.793
rs2521564	A	G	-0.014	0.002	1.58E-09	0.593	36.000
rs2027761	T	C	0.028	0.004	2.54E-14	0.113	58.089
rs3133084	G	A	-0.019	0.003	9.73E-12	0.234	45.081
rs2896630	T	A	-0.013	0.002	1.47E-08	0.544	32.937
rs71490394	A	G	0.026	0.002	1.66E-27	0.367	120.999
rs10444582	C	A	-0.016	0.003	1.25E-10	0.345	40.449
rs4074726	T	C	0.015	0.003	7.06E-09	0.292	32.842
rs7139311	T	C	0.013	0.002	1.69E-08	0.535	32.440
rs12721420	T	C	-0.018	0.003	6.42E-10	0.204	38.098
rs12825748	C	G	0.020	0.003	6.27E-15	0.309	62.726
rs2162319	G	A	0.026	0.003	9.28E-17	0.176	68.195
rs35506	A	T	0.020	0.003	3.38E-14	0.716	57.409
rs10843015	T	C	0.021	0.004	9.51E-09	0.126	33.383
rs10850377	A	G	0.020	0.003	4.02E-15	0.341	60.840
rs56196860	A	C	-0.045	0.007	7.82E-11	0.031	41.780
rs2098332	G	C	0.014	0.002	1.88E-08	0.346	32.585
rs676274	G	A	-0.014	0.002	2.47E-09	0.418	34.028
rs10771754	A	G	0.016	0.002	1.99E-11	0.593	43.340
rs11176001	A	C	-0.029	0.004	4.88E-17	0.131	69.127
rs7977418	C	T	-0.032	0.002	1.42E-43	0.459	195.999

rs12313454	G	A	0.025	0.004	6.54E-12	0.117	47.075
rs2195242	G	A	-0.017	0.003	3.83E-10	0.738	37.799
rs10773000	T	G	-0.014	0.003	2.63E-08	0.333	30.913
rs2812208	C	G	0.061	0.008	4.95E-14	0.021	57.086
rs4885681	T	C	0.019	0.003	1.83E-12	0.725	51.177
rs2875610	A	G	0.020	0.002	6.48E-16	0.656	67.376
rs12894780	C	T	-0.024	0.004	1.52E-11	0.126	46.629
rs10141786	G	A	-0.018	0.002	3.05E-14	0.598	57.507
rs55951163	A	G	0.018	0.002	4.55E-13	0.379	53.168
rs8006310	G	A	-0.014	0.002	4.74E-09	0.448	33.543
rs11621587	C	G	0.034	0.003	9.57E-30	0.182	130.720
rs8030071	A	C	0.014	0.002	1.95E-08	0.355	32.585
rs2304645	C	G	-0.015	0.002	2.93E-11	0.518	44.831
rs8033889	T	G	-0.030	0.003	7.08E-27	0.219	115.562
rs8040868	C	T	-0.019	0.002	7.92E-15	0.392	60.062
rs1896797	A	G	0.022	0.002	1.59E-20	0.490	89.837
rs2445772	G	C	0.015	0.003	1.46E-09	0.691	37.454
rs2137053	A	C	0.021	0.004	2.57E-08	0.109	30.540
rs731052	T	C	0.038	0.005	2.06E-16	0.067	68.601
rs3751837	T	C	-0.024	0.003	6.35E-17	0.220	70.440
rs6500126	A	G	0.014	0.003	4.91E-09	0.339	33.177
rs11075744	A	G	-0.021	0.002	9.91E-19	0.573	75.835
rs12928722	T	A	-0.023	0.002	6.26E-22	0.595	91.840
rs78579285	T	C	-0.021	0.003	1.85E-11	0.182	46.694
rs12928404	C	T	-0.014	0.002	4.49E-09	0.410	33.543
rs6539952	A	C	-0.017	0.003	3.50E-10	0.262	39.643
rs4889599	T	C	0.022	0.002	2.43E-20	0.628	85.562
rs7198383	T	C	0.016	0.003	6.78E-10	0.683	38.440
rs76219171	A	G	-0.028	0.005	1.26E-08	0.060	32.035
rs2345443	G	A	-0.022	0.003	3.03E-18	0.689	78.145
rs35568625	C	T	0.016	0.002	1.48E-11	0.535	46.126
rs4968200	G	C	0.022	0.003	4.54E-11	0.858	44.041
rs62091797	G	T	0.026	0.003	5.52E-14	0.137	57.581
rs11657029	A	G	-0.025	0.003	1.39E-16	0.189	67.240
rs74944644	C	T	-0.043	0.003	4.19E-49	0.216	214.773
rs28519449	T	C	0.021	0.002	3.51E-18	0.404	72.960
rs930527	C	T	-0.013	0.002	3.12E-08	0.586	29.793
rs11078321	C	T	-0.014	0.002	4.75E-09	0.528	35.480
rs8067252	T	C	0.019	0.003	1.26E-11	0.229	47.020
rs886444	A	G	0.014	0.002	8.37E-09	0.405	32.111
rs227728	A	T	-0.016	0.003	9.65E-11	0.334	40.960

	rs6501455	G	A	-0.030	0.002	1.28E-36	0.499	165.625
	rs513953	G	A	0.027	0.003	1.24E-24	0.746	102.234
	rs303752	A	G	-0.015	0.002	1.29E-10	0.409	41.173
	rs9807668	T	C	0.029	0.004	1.39E-13	0.095	54.022
	rs8089099	A	G	0.016	0.003	4.35E-10	0.275	39.303
	rs55916581	C	T	0.015	0.003	1.12E-08	0.264	33.729
	rs4800410	C	A	0.020	0.002	1.25E-16	0.406	67.376
	rs2202572	C	A	-0.014	0.003	5.78E-09	0.671	33.177
	rs11085744	T	C	-0.015	0.002	7.83E-11	0.560	40.640
	rs552320	G	A	0.016	0.003	7.97E-11	0.691	43.033
	rs2909	G	A	-0.024	0.004	1.49E-11	0.126	46.240
	rs4803982	C	T	-0.017	0.003	9.23E-09	0.814	32.871
	rs6103666	A	G	-0.018	0.003	4.83E-12	0.271	48.463
	rs6066089	C	G	0.022	0.003	3.39E-18	0.306	77.440
	rs2206925	C	T	0.021	0.003	3.07E-17	0.638	68.558
	rs2236180	C	T	-0.021	0.003	1.02E-12	0.185	50.410
	rs3746429	T	C	-0.021	0.003	2.18E-11	0.164	45.453
	rs4809327	T	C	-0.027	0.003	1.33E-26	0.676	114.062
	rs6032942	C	G	0.017	0.003	3.47E-10	0.233	41.055
	rs6042162	T	C	0.014	0.003	8.59E-09	0.685	33.177
	rs11702251	T	G	-0.017	0.003	9.75E-10	0.223	37.297
	rs113111175	T	C	0.022	0.004	1.11E-09	0.122	37.345
	rs116894149	T	C	-0.029	0.005	6.66E-10	0.065	38.598
	rs415170	G	C	-0.016	0.003	2.89E-10	0.330	38.937
	rs35089989	C	T	0.031	0.003	1.15E-27	0.221	119.433
	rs2023681	G	A	-0.036	0.004	1.10E-19	0.907	82.809
	rs3827382	A	G	-0.023	0.004	1.85E-08	0.093	31.640
FVC	rs2885697	T	G	-0.017	0.003	2.67E-12	0.666	47.334
	rs1392140	A	G	-0.018	0.003	7.02E-12	0.726	48.463
	rs2999158	C	T	-0.015	0.003	6.24E-09	0.665	33.640
	rs2146098	G	A	0.018	0.002	2.03E-13	0.355	56.250
	rs555520	C	G	-0.016	0.003	6.51E-10	0.658	38.440
	rs17009288	C	A	0.025	0.003	3.68E-22	0.294	93.196
	rs1459222	T	G	-0.014	0.003	6.43E-09	0.358	32.262
	rs9435733	C	T	0.023	0.002	3.35E-23	0.518	100.871
	rs17261915	C	T	-0.015	0.003	1.23E-08	0.253	31.693
	rs4652333	C	G	0.017	0.003	1.19E-11	0.312	47.334
	rs1008833	G	A	0.024	0.003	2.41E-13	0.146	54.670
	rs2807339	C	T	0.016	0.003	1.74E-09	0.759	36.894
	rs1192414	G	A	-0.020	0.003	2.69E-11	0.821	43.305
	rs878471	A	G	-0.028	0.002	1.50E-31	0.580	134.173

rs2702200	A	G	-0.013	0.002	1.34E-08	0.556	31.173
rs2816992	G	A	0.016	0.002	7.33E-12	0.412	46.694
rs76109497	A	G	0.017	0.003	3.45E-08	0.174	30.784
rs7414828	A	G	-0.034	0.005	2.14E-13	0.070	53.671
rs12737805	G	A	-0.021	0.003	3.58E-13	0.221	54.127
rs2502136	C	T	0.013	0.002	2.37E-08	0.531	30.250
rs12727617	T	C	0.024	0.003	2.31E-18	0.243	79.012
rs10922140	A	G	0.014	0.002	7.18E-09	0.448	33.543
rs6658835	G	A	0.022	0.003	2.09E-16	0.268	65.790
rs4952564	G	A	0.017	0.003	6.97E-12	0.319	47.334
rs732132	T	C	-0.033	0.003	6.13E-35	0.258	151.198
rs66726071	G	A	-0.025	0.003	2.01E-13	0.137	55.371
rs6755553	T	C	-0.015	0.002	6.14E-10	0.546	37.515
rs13430465	T	C	0.037	0.004	1.87E-17	0.080	73.241
rs13009582	A	G	0.016	0.002	1.52E-11	0.451	47.191
rs34393323	C	G	0.014	0.002	5.69E-09	0.460	33.062
rs1249093	G	A	-0.021	0.002	3.93E-18	0.462	73.673
rs10929647	A	C	-0.014	0.002	5.64E-09	0.513	34.964
rs62213409	T	C	-0.021	0.003	1.48E-15	0.294	62.775
rs6431620	G	T	-0.019	0.003	1.40E-10	0.211	40.695
rs34752570	A	G	0.016	0.002	2.46E-11	0.454	43.890
rs7572476	T	C	-0.019	0.002	2.96E-16	0.455	64.668
rs17855177	T	C	0.017	0.003	9.49E-12	0.337	46.240
rs6754311	C	T	0.023	0.003	7.35E-17	0.266	70.684
rs6740092	A	T	0.026	0.003	9.66E-15	0.855	58.477
rs62193248	T	A	-0.015	0.003	1.79E-09	0.340	36.481
rs1554902	A	C	0.015	0.002	1.22E-09	0.457	36.502
rs12713676	T	C	-0.016	0.003	2.58E-10	0.656	40.449
rs2304340	G	A	0.016	0.002	4.06E-11	0.592	43.340
rs13035369	C	T	-0.020	0.004	8.66E-09	0.131	32.980
rs2571445	G	A	0.022	0.002	4.53E-20	0.603	84.027
rs13433809	G	A	-0.020	0.002	6.80E-18	0.439	71.543
rs7613360	T	C	-0.014	0.002	3.40E-09	0.393	35.502
rs2369796	T	A	-0.013	0.002	3.76E-08	0.464	29.340
rs10489880	G	A	0.017	0.003	2.38E-10	0.279	40.763
rs2974389	G	A	-0.016	0.002	4.73E-11	0.574	41.710
rs4132748	C	T	0.020	0.003	6.96E-15	0.693	59.171
rs35480566	G	A	0.022	0.002	1.25E-20	0.440	84.793
rs56094673	T	C	-0.038	0.004	1.56E-17	0.077	73.024
rs7648255	A	T	-0.015	0.003	2.19E-08	0.268	30.046
rs6775611	T	C	0.015	0.003	2.23E-09	0.338	36.000

rs78101726	G	A	-0.027	0.003	4.61E-16	0.154	64.485
rs6806825	C	T	0.015	0.003	1.87E-09	0.649	35.046
rs12497779	T	G	-0.032	0.003	1.85E-30	0.233	129.796
rs11718554	G	A	-0.026	0.003	1.68E-26	0.344	110.670
rs13137088	C	T	0.014	0.002	7.26E-09	0.495	32.585
rs17767210	T	C	-0.023	0.002	3.45E-21	0.592	88.673
rs11727676	C	T	-0.033	0.004	8.05E-16	0.095	66.015
rs10005540	T	C	0.017	0.002	1.53E-12	0.615	51.960
rs34811474	A	G	0.018	0.003	3.32E-10	0.230	39.510
rs11722225	C	T	0.056	0.005	1.78E-32	0.067	142.471
rs1154862	C	T	0.014	0.003	1.68E-08	0.322	31.809
rs6554237	T	C	0.015	0.002	7.53E-10	0.486	36.502
rs58507021	C	T	0.015	0.003	1.45E-08	0.304	31.102
rs7664805	C	G	-0.032	0.004	6.63E-16	0.103	65.205
rs62347029	A	T	0.016	0.003	1.72E-08	0.226	32.653
rs1054661	C	T	0.017	0.002	4.61E-12	0.347	49.585
rs2609261	G	A	0.025	0.003	5.24E-18	0.785	73.132
rs6886191	G	A	0.015	0.002	1.42E-10	0.558	40.111
rs4490572	A	G	-0.016	0.002	8.54E-12	0.463	46.126
rs715285	G	A	-0.019	0.002	5.46E-15	0.458	59.418
rs11747434	C	T	0.015	0.003	1.23E-08	0.278	33.284
rs6867471	T	C	0.013	0.002	4.90E-08	0.363	30.710
rs156838	G	A	0.015	0.003	1.08E-09	0.642	36.481
rs521074	T	C	0.014	0.003	2.85E-08	0.669	30.913
rs72776472	G	A	-0.021	0.003	1.65E-14	0.234	57.868
rs11241692	C	T	-0.014	0.002	9.46E-09	0.440	32.111
rs152197	T	C	0.018	0.003	8.32E-09	0.176	32.970
rs1862342	T	C	0.016	0.002	3.83E-11	0.448	42.793
rs1423127	T	C	0.017	0.002	8.39E-12	0.382	47.265
rs268717	T	C	-0.034	0.004	3.23E-17	0.907	72.250
rs6859730	T	A	-0.021	0.003	8.76E-17	0.672	67.897
rs6595403	C	T	-0.016	0.002	8.79E-11	0.424	41.710
rs891903	A	G	-0.023	0.003	9.60E-18	0.254	74.470
rs12204336	A	G	-0.016	0.002	1.46E-10	0.374	41.710
rs4959021	C	T	-0.030	0.003	5.67E-21	0.812	90.556
rs9472541	A	T	-0.015	0.003	2.47E-09	0.285	35.083
rs6938343	T	C	0.016	0.003	1.80E-08	0.232	31.842
rs723588	C	T	0.024	0.003	2.62E-12	0.143	47.772
rs314262	A	G	0.016	0.002	3.84E-12	0.546	46.694
rs3253	T	C	0.018	0.003	7.83E-13	0.314	52.998
rs55775505	T	C	-0.017	0.003	2.32E-11	0.312	45.697

rs148561062	A	G	-0.031	0.005	1.82E-09	0.055	36.231
rs9321170	A	G	0.015	0.003	6.60E-09	0.725	34.628
rs13216391	G	A	-0.035	0.003	6.63E-31	0.179	136.889
rs7738842	C	G	0.016	0.003	2.05E-08	0.231	31.041
rs661857	C	T	0.016	0.002	8.28E-12	0.484	45.001
rs9392172	G	C	-0.023	0.002	6.18E-23	0.469	99.999
rs10214450	T	C	0.038	0.006	1.20E-11	0.046	45.562
rs13220614	C	T	-0.014	0.002	7.14E-09	0.600	33.543
rs72845046	A	T	-0.038	0.004	1.26E-24	0.115	103.269
rs13198515	G	A	-0.017	0.003	4.71E-11	0.268	44.787
rs1490384	T	C	0.021	0.002	3.13E-19	0.498	77.293
rs17280293	G	A	-0.070	0.007	8.57E-22	0.027	91.687
rs6956494	T	C	0.013	0.002	4.24E-08	0.403	29.793
rs11767893	T	C	0.016	0.003	1.60E-09	0.314	36.446
rs6949451	T	C	0.013	0.002	1.17E-08	0.461	33.438
rs1708302	T	C	0.019	0.002	1.56E-15	0.500	65.398
rs10251085	A	G	0.017	0.003	2.31E-09	0.243	35.148
rs13227429	C	T	-0.016	0.002	8.83E-12	0.561	46.126
rs11980271	G	C	-0.016	0.003	2.37E-10	0.331	39.438
rs7806296	A	G	-0.018	0.002	2.87E-13	0.360	54.390
rs6968518	C	T	-0.019	0.002	5.98E-15	0.566	60.062
rs1534566	T	C	0.017	0.003	1.05E-11	0.338	46.240
rs10951992	C	T	-0.014	0.002	1.72E-08	0.440	31.640
rs12698403	A	G	-0.017	0.002	2.37E-12	0.442	48.418
rs1516874	A	C	0.016	0.003	4.81E-09	0.751	33.889
rs1543852	G	A	-0.013	0.002	4.85E-08	0.598	29.793
rs62511595	G	A	0.014	0.002	2.86E-08	0.376	31.640
rs4737183	G	A	0.017	0.002	1.67E-13	0.474	52.562
rs7838717	C	T	0.023	0.003	6.47E-21	0.637	87.609
rs10089933	C	T	-0.017	0.003	1.81E-09	0.243	37.345
rs12543555	G	A	-0.023	0.003	4.91E-15	0.207	62.355
rs9643242	A	G	0.019	0.003	6.61E-09	0.154	32.801
rs2976602	T	C	0.013	0.002	2.27E-08	0.460	30.710
rs8181164	T	C	-0.014	0.002	9.29E-09	0.502	32.111
rs1887667	G	T	0.013	0.002	1.41E-08	0.441	30.710
rs62579839	C	T	0.016	0.003	1.91E-09	0.284	36.463
rs55695529	T	A	0.022	0.003	4.82E-11	0.146	44.444
rs2095024	A	G	0.016	0.002	1.43E-11	0.588	45.001
rs7029491	G	C	0.017	0.003	5.79E-12	0.333	47.886
rs7041139	T	C	-0.015	0.003	2.23E-09	0.322	36.000
rs4877815	G	A	0.015	0.002	1.67E-10	0.475	39.585

rs60150206	A	G	0.045	0.004	1.63E-27	0.091	118.862
rs11103381	C	A	-0.024	0.003	4.86E-21	0.327	90.630
rs10448340	G	T	-0.015	0.003	1.88E-09	0.321	36.966
rs10997955	T	C	-0.018	0.002	3.32E-14	0.589	57.507
rs12413039	G	C	0.019	0.003	7.09E-13	0.270	53.402
rs12571363	A	C	-0.031	0.004	2.06E-16	0.107	68.279
rs10761722	G	C	-0.019	0.003	4.97E-10	0.815	39.271
rs2637254	A	G	-0.022	0.002	1.68E-21	0.511	87.111
rs17466747	A	G	-0.014	0.003	1.14E-08	0.339	32.262
rs55942359	A	G	0.025	0.004	1.28E-08	0.080	32.025
rs41310284	A	C	0.028	0.004	1.70E-12	0.101	48.650
rs1954179	G	A	0.015	0.003	4.85E-09	0.355	33.640
rs10508438	A	G	-0.017	0.003	3.08E-12	0.336	47.886
rs2393729	T	C	-0.017	0.002	2.04E-12	0.421	49.000
rs1776634	T	G	0.016	0.003	1.17E-09	0.733	38.344
rs7943381	G	A	0.016	0.003	3.37E-09	0.255	34.244
rs7949728	G	C	-0.019	0.002	9.53E-15	0.410	59.418
rs1801144	C	G	0.024	0.003	8.72E-22	0.362	89.113
rs2256154	T	C	-0.015	0.003	7.35E-10	0.659	37.454
rs6485443	T	C	-0.020	0.003	3.53E-15	0.317	62.726
rs10741306	T	A	-0.014	0.003	5.09E-09	0.357	33.177
rs4936101	G	A	-0.015	0.002	1.26E-09	0.595	37.007
rs4938337	T	A	0.020	0.004	1.83E-08	0.126	32.111
rs7946009	C	T	-0.017	0.003	3.20E-11	0.659	43.560
rs1047912	T	C	0.017	0.003	4.85E-11	0.299	44.622
rs1109391	T	G	0.017	0.002	2.42E-13	0.568	51.361
rs35506	A	T	0.023	0.003	6.88E-19	0.716	79.621
rs3924361	A	G	0.018	0.002	4.90E-14	0.553	54.390
rs10492367	T	G	0.024	0.003	2.88E-15	0.188	61.884
rs1125909	A	G	0.014	0.003	3.61E-08	0.694	30.913
rs12825748	C	G	0.020	0.003	1.68E-14	0.309	61.465
rs11051013	T	C	0.016	0.002	5.11E-12	0.592	46.694
rs10850377	A	G	0.016	0.003	1.82E-10	0.341	40.449
rs56196860	A	C	-0.053	0.007	1.45E-14	0.031	59.223
rs7977418	C	T	-0.038	0.002	9.57E-59	0.459	271.531
rs1383304	G	A	-0.024	0.004	2.59E-12	0.132	48.203
rs2195242	G	A	-0.016	0.003	4.60E-09	0.738	33.812
rs1247943	A	G	0.014	0.002	1.02E-09	0.533	36.000
rs803751	T	C	-0.025	0.003	1.13E-23	0.345	96.825
rs4885681	T	C	0.018	0.003	1.31E-11	0.725	44.444
rs2812208	C	G	0.062	0.008	2.30E-14	0.021	57.723

rs2209237	T	A	0.014	0.003	2.65E-08	0.355	30.030
rs11159090	T	C	0.014	0.002	8.73E-09	0.600	33.543
rs6571778	A	G	-0.014	0.002	2.12E-08	0.353	32.585
rs2286425	T	C	-0.021	0.002	6.53E-19	0.526	75.835
rs1052939	A	G	-0.015	0.003	4.82E-08	0.262	29.642
rs2474032	C	T	0.013	0.002	2.29E-08	0.437	30.710
rs10498635	T	C	0.036	0.003	1.34E-32	0.183	145.604
rs736281	C	T	-0.014	0.002	2.14E-09	0.600	36.000
rs931794	A	G	0.017	0.003	4.60E-12	0.664	47.334
rs7182990	G	A	0.017	0.002	8.10E-13	0.506	53.353
rs35189772	G	A	-0.019	0.004	2.41E-08	0.141	30.407
rs4924525	A	C	-0.017	0.002	3.44E-13	0.522	55.276
rs2445772	G	C	0.016	0.003	2.52E-10	0.691	41.473
rs35688953	C	T	-0.023	0.003	4.30E-17	0.230	69.841
rs8027022	A	G	0.017	0.002	2.77E-13	0.494	54.631
rs28472312	T	C	-0.016	0.003	1.00E-09	0.701	37.398
rs76219171	A	G	-0.035	0.005	2.09E-12	0.060	49.280
rs3790076	T	G	-0.024	0.002	1.23E-23	0.573	98.340
rs9927855	C	T	-0.017	0.003	7.69E-11	0.696	41.751
rs7198383	T	C	0.018	0.003	1.73E-12	0.682	50.694
rs8062719	G	A	0.019	0.002	3.19E-15	0.636	64.000
rs3751837	T	C	-0.031	0.003	9.07E-28	0.220	120.999
rs11074546	T	G	0.017	0.003	1.99E-10	0.262	39.178
rs7203231	G	T	-0.013	0.002	4.93E-08	0.489	28.890
rs370240672	C	T	-0.039	0.007	3.52E-09	0.036	35.096
rs2345443	G	A	-0.018	0.003	1.36E-12	0.689	52.417
rs1991556	A	G	-0.041	0.003	1.21E-46	0.222	202.816
rs28519449	T	C	0.021	0.002	1.27E-18	0.404	75.835
rs2521879	G	A	-0.020	0.003	1.16E-11	0.804	45.787
rs1215	G	A	-0.022	0.003	9.64E-11	0.143	43.240
rs719615	A	G	-0.022	0.002	7.02E-20	0.477	80.251
rs7212620	A	T	-0.015	0.003	7.25E-09	0.712	33.729
rs8067252	T	C	0.023	0.003	7.94E-16	0.229	62.901
rs1972503	A	G	-0.015	0.002	4.07E-10	0.525	40.295
rs12945803	C	T	-0.020	0.003	1.88E-12	0.218	51.020
rs59606152	T	C	0.037	0.004	9.15E-21	0.112	88.552
rs9947743	A	G	-0.019	0.003	1.13E-10	0.787	40.695
rs2202572	C	A	-0.016	0.003	7.01E-11	0.671	41.990
rs470450	A	G	0.016	0.003	4.82E-10	0.686	39.438
rs9950661	C	T	0.014	0.002	1.47E-09	0.499	37.582
rs77862575	C	T	0.035	0.006	3.01E-08	0.036	30.688

	rs116336057	C	G	-0.018	0.003	5.65E-09	0.182	32.970
	rs1668091	C	T	0.017	0.003	5.24E-12	0.319	47.886
	rs7233091	A	G	-0.014	0.002	1.78E-09	0.454	37.582
	rs8104651	C	T	0.014	0.002	4.91E-09	0.438	33.062
	rs62621197	T	C	-0.041	0.007	4.85E-10	0.037	39.157
	rs1864078	C	G	0.021	0.002	1.19E-18	0.380	78.027
	rs4803981	T	C	-0.017	0.003	9.48E-09	0.814	33.254
	rs2240147	A	C	0.021	0.004	2.44E-08	0.117	30.998
	rs274792	T	C	0.018	0.003	4.19E-13	0.687	53.582
	rs2967516	G	A	0.015	0.003	3.72E-09	0.291	34.177
	rs143384	G	A	-0.024	0.002	1.03E-23	0.404	99.168
	rs1007330	T	C	0.024	0.002	2.17E-23	0.383	99.168
	rs6016084	C	G	0.013	0.002	3.58E-08	0.545	31.457
	rs6090040	C	A	0.013	0.002	4.60E-08	0.521	29.340
	rs979012	C	T	0.026	0.002	1.20E-26	0.637	117.360
	rs2273101	T	C	0.018	0.003	1.71E-11	0.247	45.437
	rs8125514	A	G	-0.029	0.003	3.37E-31	0.676	136.422
	rs11702251	T	G	-0.018	0.003	5.90E-11	0.223	43.183
	rs5992134	T	G	0.021	0.003	3.65E-14	0.239	59.347
	rs509317	T	A	0.018	0.003	6.44E-09	0.828	33.715
	rs4396807	C	G	-0.014	0.003	4.19E-08	0.323	30.030
	rs5763825	G	A	0.020	0.004	4.01E-08	0.116	30.864
CRP	rs3027061	G	A	0.033	0.004	3.32E-18	0.365	75.803
	rs4655802	A	G	-0.025	0.004	1.88E-09	0.615	36.150
	rs4129267	T	C	-0.088	0.004	1.20E-129	0.386	587.090
	rs2794520	T	C	-0.182	0.004	1.00E-200	0.334	2408.850
	rs1816732	C	T	-0.019	0.004	4.98E-07	0.636	25.310
	rs1932965	A	T	-0.031	0.006	7.56E-07	0.897	24.501
	rs1805096	A	G	-0.104	0.004	2.17E-183	0.386	834.185
	rs6672627	A	C	-0.037	0.005	2.89E-13	0.146	53.373
	rs2293476	C	G	0.030	0.004	8.27E-13	0.226	51.302
	rs4660586	T	C	0.019	0.004	2.31E-06	0.730	22.347
	rs7516611	C	A	-0.032	0.007	7.99E-07	0.079	24.391
	rs12742784	T	C	-0.022	0.005	7.27E-07	0.199	24.579
	rs7547076	C	T	-0.021	0.004	6.32E-07	0.277	24.848
	rs6686560	A	G	0.028	0.006	3.45E-06	0.112	21.578
	rs10925027	C	T	-0.036	0.004	4.25E-21	0.600	88.985
	rs469772	T	C	-0.031	0.005	5.54E-12	0.194	47.571
	rs7521729	A	C	0.037	0.006	9.67E-10	0.130	37.450
	rs1260326	C	T	-0.073	0.004	2.72E-92	0.607	415.481
	rs13409371	A	G	0.048	0.004	5.07E-36	0.430	157.189

rs1441169	G	A	-0.025	0.004	2.27E-11	0.533	44.776
rs9284725	A	C	-0.027	0.004	7.34E-11	0.761	42.483
rs1592	C	A	-0.018	0.004	3.93E-06	0.449	21.328
rs10208608	C	T	0.046	0.010	1.32E-06	0.042	23.425
rs12995480	C	T	0.031	0.005	1.24E-10	0.831	41.459
rs4246598	A	C	0.022	0.004	5.11E-10	0.463	38.690
rs6724667	A	G	0.023	0.004	7.51E-08	0.702	28.966
rs6761029	A	C	0.021	0.004	1.44E-07	0.254	27.714
rs1509394	T	C	0.026	0.004	6.05E-10	0.543	38.361
rs7575523	G	T	-0.018	0.004	3.00E-06	0.619	21.850
rs2352975	C	T	0.025	0.004	6.43E-10	0.304	38.242
rs2596937	C	T	0.023	0.005	3.91E-07	0.198	25.773
rs2606228	C	A	-0.019	0.004	1.86E-06	0.643	22.766
rs7640424	T	C	-0.019	0.004	3.85E-06	0.310	21.363
rs6792725	G	A	-0.023	0.004	7.17E-08	0.680	29.066
rs931929	A	G	-0.019	0.004	4.09E-06	0.763	21.254
rs2324999	T	C	0.022	0.005	2.73E-06	0.203	22.032
rs1514895	G	A	0.021	0.004	1.18E-07	0.288	28.102
rs118182	G	A	0.037	0.007	2.40E-07	0.920	26.718
rs2884401	C	G	-0.018	0.004	4.16E-06	0.740	21.224
rs1012793	C	G	-0.021	0.004	1.35E-06	0.211	23.387
rs490928	T	G	-0.023	0.005	2.68E-06	0.211	22.064
rs17658229	C	T	0.056	0.010	5.50E-09	0.046	34.056
rs1062326	G	T	0.019	0.004	3.95E-07	0.619	25.755
rs12202641	T	C	-0.023	0.004	3.00E-10	0.394	39.749
rs9271608	G	A	0.042	0.005	2.33E-17	0.215	71.948
rs2327832	G	A	0.023	0.005	4.02E-07	0.202	25.719
rs1490384	T	C	-0.025	0.004	2.65E-12	0.512	49.003
rs3134899	T	C	0.023	0.004	4.93E-08	0.767	29.793
rs3843540	C	T	-0.026	0.005	2.11E-07	0.153	26.968
rs10240168	G	C	-0.029	0.004	4.11E-11	0.221	43.621
rs11769828	T	C	-0.020	0.004	9.93E-07	0.239	23.972
rs13233571	T	C	-0.057	0.005	2.95E-25	0.120	107.949
rs1880241	G	A	-0.028	0.004	8.41E-14	0.483	55.781
rs4526310	T	A	-0.030	0.006	5.68E-07	0.128	25.055
rs2710804	C	T	0.021	0.004	1.30E-08	0.367	32.371
rs7010484	T	C	0.019	0.004	1.49E-06	0.673	23.203
rs2064009	T	C	0.027	0.004	2.28E-14	0.581	58.355
rs2891677	T	C	0.020	0.004	1.59E-08	0.539	31.993
rs4841132	G	A	0.065	0.006	2.00E-25	0.909	108.719
rs6601302	G	T	-0.031	0.004	9.80E-12	0.737	46.445

rs10819190	A	G	0.020	0.004	5.22E-07	0.371	25.219
rs644234	G	T	0.023	0.004	1.13E-09	0.369	37.138
rs10969334	A	C	-0.018	0.004	6.04E-07	0.402	24.939
rs6482190	G	A	-0.020	0.004	1.43E-06	0.718	23.272
rs2265185	C	A	-0.017	0.004	4.64E-06	0.592	21.002
rs7083516	C	T	-0.017	0.004	2.21E-06	0.383	22.430
rs1051338	G	T	0.024	0.004	2.27E-09	0.308	35.787
rs1994721	A	G	-0.028	0.005	4.17E-07	0.139	25.647
rs2679053	G	A	-0.020	0.004	3.88E-07	0.609	25.787
rs1582763	A	G	-0.022	0.004	2.37E-09	0.372	35.699
rs10832027	A	G	0.026	0.004	4.43E-12	0.669	47.992
rs6485751	C	G	-0.031	0.004	1.43E-12	0.218	50.218
rs10898885	C	T	-0.022	0.004	1.00E-07	0.759	28.422
rs4767920	A	G	-0.039	0.005	4.00E-15	0.842	61.795
rs7310409	G	A	0.137	0.004	1.00E-200	0.608	1368.048
rs553144	C	G	0.018	0.004	4.50E-07	0.490	25.498
rs11108056	G	C	-0.028	0.004	5.42E-14	0.424	56.643
rs10778215	A	T	-0.033	0.004	1.86E-20	0.489	86.075
rs6573778	C	T	0.024	0.005	1.28E-07	0.533	27.939
rs2239222	G	A	0.035	0.004	9.87E-20	0.364	82.739
rs7144423	T	C	0.026	0.005	1.10E-07	0.171	28.240
rs12587622	A	G	-0.021	0.004	8.52E-09	0.501	33.210
rs340005	A	G	0.030	0.004	1.01E-15	0.621	64.510
rs882829	G	C	-0.042	0.009	9.16E-07	0.068	24.130
rs906308	A	T	0.017	0.004	3.38E-06	0.496	21.618
rs6498011	G	A	-0.017	0.004	3.61E-06	0.617	21.494
rs7342694	C	T	0.018	0.004	1.04E-06	0.610	23.897
rs10521222	T	C	-0.104	0.011	2.06E-22	0.045	94.970
rs1558902	A	T	0.034	0.004	5.20E-20	0.406	84.028
rs178810	T	C	0.020	0.004	2.95E-08	0.563	30.792
rs17138478	A	C	0.036	0.007	1.30E-06	0.118	23.463
rs8074524	T	C	-0.021	0.004	2.93E-06	0.195	21.887
rs10512597	C	T	0.037	0.005	4.44E-14	0.817	57.037
rs11871028	T	C	0.028	0.006	1.86E-06	0.101	22.770
rs1292061	G	A	0.018	0.004	3.86E-07	0.539	25.795
rs4092465	G	A	0.027	0.004	3.11E-10	0.651	39.660
rs12960928	C	T	0.024	0.004	1.91E-09	0.267	36.126
rs2852151	A	G	0.025	0.004	1.36E-11	0.405	45.798
rs17356664	T	C	0.019	0.004	2.39E-06	0.301	22.289
rs4420638	G	A	-0.229	0.006	1.00E-200	0.180	1404.816
rs387976	C	A	0.026	0.004	1.05E-10	0.332	41.783

	rs1560117	T	C	0.022	0.004	5.15E-08	0.468	29.707
	rs6128397	G	A	0.022	0.004	6.38E-08	0.702	29.281
	rs1800961	T	C	-0.112	0.011	4.63E-23	0.034	97.933
	rs2315008	G	T	0.023	0.004	5.36E-10	0.688	38.603
	rs2836878	A	G	-0.043	0.004	7.71E-26	0.274	110.622
	rs2211860	T	C	-0.018	0.004	1.96E-06	0.401	22.663
	rs2277844	A	G	-0.018	0.004	7.46E-07	0.552	24.530
	rs6001193	G	A	-0.028	0.004	6.53E-14	0.351	56.306
	rs138342	T	C	0.019	0.004	2.78E-07	0.454	26.436
GlycA	rs12239736	A	T	-0.039	0.004	2.90E-19	0.341	80.530
	rs6698842	T	C	0.031	0.004	2.00E-13	0.631	53.976
	rs56188865	C	T	-0.028	0.004	1.70E-11	0.372	45.262
	rs12032372	C	T	-0.030	0.005	1.50E-09	0.209	36.586
	rs12073837	T	C	-0.026	0.005	1.50E-08	0.277	32.042
	rs182050989	T	C	0.073	0.012	3.60E-09	0.028	34.832
	rs6424109	A	C	0.036	0.006	5.90E-09	0.875	33.872
	rs6734238	G	A	0.025	0.004	3.10E-09	0.403	35.120
	rs676210	A	G	-0.028	0.005	2.40E-08	0.206	31.137
	rs1260326	C	T	-0.101	0.004	8.20E-130	0.604	587.608
	rs6732543	T	C	-0.031	0.006	4.60E-08	0.852	29.871
	rs79287178	A	G	0.099	0.012	1.40E-15	0.031	63.700
	rs62259939	A	G	0.025	0.004	8.40E-10	0.435	37.659
	rs2070634	G	T	0.037	0.004	1.20E-19	0.514	82.202
	rs7697204	T	C	0.031	0.005	5.60E-11	0.741	42.944
	rs13108218	G	A	-0.039	0.004	4.60E-20	0.615	84.134
	rs11429307	GT	G	0.028	0.005	4.50E-08	0.192	29.920
	rs72801474	A	G	-0.042	0.007	2.20E-09	0.093	35.798
	rs6452937	G	A	-0.033	0.006	2.80E-08	0.139	30.857
	rs1801020	G	A	0.027	0.005	1.70E-08	0.747	31.788
	rs1005599	C	G	0.053	0.005	4.40E-32	0.711	138.992
	rs117733303	G	A	-0.121	0.015	1.20E-15	0.019	64.061
	rs10455872	G	A	-0.078	0.008	1.10E-24	0.079	105.225
	rs9258357	C	T	0.031	0.006	2.20E-08	0.838	31.332
	rs12208357	T	C	0.047	0.008	3.70E-09	0.070	34.797
	rs9270074	C	T	0.048	0.006	1.90E-17	0.844	72.266
	rs4717804	C	A	-0.048	0.008	7.90E-09	0.930	33.289
	rs62466318	T	C	-0.085	0.005	4.90E-63	0.204	280.829
	rs59296513	A	G	-0.024	0.004	2.90E-08	0.320	30.782
	rs55780214	A	T	-0.032	0.005	2.00E-12	0.288	49.441
	rs325	C	T	-0.088	0.007	3.40E-38	0.101	166.955
	rs1561748	C	G	-0.039	0.005	7.00E-15	0.267	60.601

	rs6601299	C	T	0.067	0.007	5.20E-23	0.899	97.555
	rs112875651	A	G	-0.071	0.004	5.40E-63	0.392	280.652
	rs113354603	A	G	0.128	0.008	1.50E-51	0.071	228.171
	rs635634	T	C	0.033	0.005	4.80E-10	0.184	38.758
	rs7924036	T	G	-0.028	0.004	9.90E-12	0.504	46.357
	rs35166446	GT	G	-0.024	0.004	3.40E-09	0.469	34.912
	rs141469619	G	A	0.129	0.022	2.00E-09	0.010	36.013
	rs78689694	C	G	0.035	0.006	5.00E-09	0.133	34.193
	rs964184	C	G	-0.106	0.006	7.20E-69	0.867	307.618
	rs12421055	C	A	0.027	0.005	1.30E-08	0.247	32.382
	rs12313762	T	C	-0.027	0.005	1.70E-08	0.239	31.765
	rs71435622	AT	A	0.026	0.005	2.90E-08	0.288	30.793
	rs17105232	A	G	0.030	0.005	2.50E-10	0.248	40.014
	rs28929474	T	C	-0.281	0.015	3.20E-82	0.020	368.944
	rs150844304	C	A	0.073	0.013	1.50E-08	0.026	32.005
	rs1132812	G	A	-0.023	0.004	1.30E-08	0.519	32.287
	rs77303550	T	C	0.164	0.005	1.00E-200	0.193	1007.231
	rs151083835	G	C	0.102	0.015	6.90E-12	0.020	47.063
	rs2925979	C	T	-0.030	0.004	2.50E-11	0.699	44.510
	rs143578173	T	C	-0.139	0.013	4.60E-28	0.028	120.618
	rs113839872	CCTGTCGACTTGG	C	0.045	0.008	4.70E-08	0.067	29.844
	rs117155836	A	G	0.137	0.012	4.40E-29	0.028	125.266
	rs1801689	C	A	-0.073	0.012	8.20E-10	0.031	37.712
	rs2445818	G	A	0.045	0.008	4.10E-08	0.932	30.094
	rs62128087	T	C	-0.041	0.006	3.30E-13	0.161	53.043
	rs116843064	A	G	-0.099	0.015	1.20E-11	0.020	45.949
	rs59774409	T	C	0.086	0.007	2.00E-31	0.083	135.957
	rs58542926	T	C	-0.057	0.008	3.40E-13	0.074	52.962
	rs149807892	T	C	-0.121	0.017	3.20E-13	0.016	53.094
	rs370692925	CTG	C	0.023	0.004	2.80E-08	0.478	30.864
	rs2294915	T	C	-0.034	0.005	4.70E-12	0.232	47.791
IL-1Ra	rs74835839	C	T	-0.068	0.014	9.67E-07	0.173	23.930
	rs532024	G	A	-0.085	0.018	1.91E-06	0.885	22.707
	rs139008239	A	G	-0.272	0.057	1.87E-06	0.017	22.706
	rs6734238	G	A	-0.204	0.010	2.46E-85	0.388	384.728
	rs4849192	A	G	0.056	0.012	2.35E-06	0.354	22.200
	rs7569284	A	G	-0.146	0.011	2.26E-39	0.698	170.847
	rs76351462	G	A	0.234	0.051	3.82E-06	0.016	21.348
	rs1684468	T	A	-0.074	0.016	3.43E-06	0.744	21.542
	rs150226685	T	C	-0.290	0.062	2.43E-06	0.012	22.187

	rs142329455	T	C	0.388	0.085	4.40E-06	0.008	21.082
	rs11103413	G	A	0.279	0.059	2.31E-06	0.018	22.280
	rs10964541	C	G	0.065	0.014	2.61E-06	0.219	22.047
	rs78078342	G	A	0.160	0.034	3.08E-06	0.034	21.703
	rs143966031	G	A	-0.263	0.056	3.00E-06	0.012	21.815
	rs563595624	C	G	-0.195	0.042	3.71E-06	0.037	21.350
	rs9542868	C	G	-0.080	0.016	3.09E-07	0.840	26.230
	rs291032	G	C	0.118	0.023	1.87E-07	0.068	27.213
	rs12908565	C	A	0.054	0.012	4.97E-06	0.499	20.896
	rs113087075	T	C	-0.151	0.033	4.27E-06	0.039	21.209
	rs147302029	A	G	0.161	0.032	6.75E-07	0.038	24.598
	rs3935280	G	A	-0.060	0.011	1.01E-08	0.617	32.977
	rs13342087	A	G	0.206	0.045	3.67E-06	0.035	21.394
	rs12947042	A	C	0.075	0.015	4.26E-07	0.221	25.683
	rs62143194	G	C	0.223	0.013	5.52E-63	0.218	281.356
	rs2075803	G	A	0.059	0.012	2.89E-07	0.579	26.141
IL-6	rs111572787	T	C	0.410	0.089	4.44E-06	0.011	21.067
	rs2228145	C	A	0.175	0.012	3.34E-45	0.379	198.473
	rs148924545	G	A	-0.182	0.038	1.51E-06	0.038	23.083
	rs1444691	G	A	-0.057	0.012	2.69E-06	0.402	22.057
	rs9811834	T	A	-0.068	0.015	4.11E-06	0.454	21.334
	rs80316479	T	C	-0.196	0.042	2.92E-06	0.027	21.895
	rs34032964	T	A	-0.410	0.088	3.57E-06	0.013	21.457
	rs7448500	G	C	0.063	0.013	8.68E-07	0.694	24.300
	rs4959106	C	T	0.082	0.014	2.37E-09	0.462	35.563
	rs76598619	C	T	-0.251	0.051	7.01E-07	0.024	24.624
	rs144530765	G	A	-0.362	0.073	7.16E-07	0.013	24.562
	rs1530088	T	C	0.068	0.015	3.95E-06	0.771	21.397
	rs2288477	G	A	-0.062	0.013	3.74E-06	0.337	21.519
	rs4802241	C	A	-0.079	0.017	1.85E-06	0.177	22.704
	rs2651097	A	G	-0.071	0.016	4.52E-06	0.251	20.980
IL-8	rs11165658	G	A	-0.058	0.012	2.69E-06	0.374	22.157
	rs79522806	T	C	0.239	0.052	4.73E-06	0.015	20.916
	rs374090093	C	T	-0.208	0.043	1.53E-06	0.097	23.096
	rs7655660	A	G	-0.188	0.024	2.67E-15	0.068	62.651
	rs972492	C	A	0.111	0.020	3.83E-08	0.857	30.084
	rs78759345	T	C	-0.083	0.018	2.13E-06	0.354	22.384
	rs2787927	G	A	0.054	0.012	3.78E-06	0.518	21.508
	rs117516143	G	A	-0.205	0.044	3.46E-06	0.026	21.523
	rs2043963	C	T	-0.057	0.012	4.67E-06	0.312	21.098
	rs6585420	C	T	-0.163	0.032	2.72E-07	0.041	26.372

	rs78741217	A	G	0.083	0.017	1.88E-06	0.127	22.752
	rs7986191	C	T	-0.161	0.034	2.08E-06	0.965	22.469
	rs142438235	G	T	0.316	0.068	3.81E-06	0.013	21.341
	rs4887995	A	G	-0.084	0.016	8.67E-08	0.789	28.760
	rs35592645	A	C	-0.060	0.013	3.03E-06	0.241	21.631
	rs72886502	T	G	-0.426	0.085	4.72E-07	0.010	25.342
IL-18	rs12049589	T	C	-0.109	0.022	1.24E-06	0.069	23.503
	rs112579775	T	C	0.231	0.051	4.76E-06	0.020	20.912
	rs1218551	T	C	-0.053	0.011	3.16E-06	0.647	21.831
	rs13391772	G	A	0.058	0.013	4.21E-06	0.544	21.187
	rs6744838	T	C	-0.159	0.035	4.35E-06	0.970	21.131
	rs385076	C	T	0.185	0.012	3.19E-55	0.627	244.449
	rs9867857	T	C	0.060	0.010	5.12E-09	0.469	34.157
	rs62251794	C	T	-0.111	0.024	4.34E-06	0.067	21.135
	rs116028368	A	G	-0.229	0.048	1.82E-06	0.022	22.739
	rs35877346	G	A	0.099	0.020	1.17E-06	0.073	23.740
	rs55872908	A	G	0.208	0.045	3.40E-06	0.022	21.554
	rs17229943	C	A	0.182	0.028	6.41E-11	0.059	42.596
	rs149607284	C	T	0.306	0.064	1.96E-06	0.022	22.616
	rs181716581	C	T	-0.227	0.049	3.91E-06	0.028	21.335
	rs17187117	C	T	0.102	0.022	4.25E-06	0.078	21.084
	rs3131005	A	G	-0.065	0.012	1.64E-07	0.729	27.581
	rs38302	A	G	0.093	0.019	7.41E-07	0.312	24.625
	rs916964	G	C	-0.074	0.013	6.41E-09	0.204	33.765
	rs4606077	C	T	0.088	0.012	4.57E-13	0.756	52.143
	rs139416129	A	G	-0.232	0.047	8.34E-07	0.019	24.259
	rs558315	G	C	-0.079	0.014	9.04E-09	0.168	33.018
	rs5744249	C	A	-0.218	0.012	3.74E-74	0.235	330.906
	rs3184504	C	T	-0.055	0.011	1.09E-06	0.538	23.938
	rs10424405	G	A	0.236	0.013	1.24E-73	0.218	329.532
IL-27	rs145363361	A	G	-0.240	0.052	3.10E-06	0.017	21.788
	rs1257169	A	C	-0.097	0.011	1.30E-17	0.528	73.376
	rs10164423	A	G	-0.129	0.026	1.16E-06	0.048	23.727
	rs113732805	A	G	0.096	0.021	3.79E-06	0.158	21.461
	rs11711157	T	C	-0.090	0.014	7.59E-11	0.289	42.529
	rs9715769	C	A	0.097	0.015	4.53E-11	0.178	43.090
	rs2844803	C	T	-0.052	0.010	3.66E-07	0.518	25.988
	rs191919970	G	A	-0.154	0.032	1.38E-06	0.048	23.334
	rs4959847	C	T	-0.064	0.012	2.18E-07	0.285	26.970
	rs11599750	T	C	-0.179	0.011	8.03E-66	0.378	291.569
	rs10883439	G	A	0.086	0.018	1.85E-06	0.889	22.626

Basophil count	rs10843390	T	C	0.062	0.011	3.09E-08	0.285	30.491
	rs10774624	A	G	-0.076	0.010	2.24E-13	0.535	54.010
	rs2291855	A	G	0.088	0.018	1.51E-06	0.088	23.112
	rs150460588	C	T	0.117	0.025	3.59E-06	0.052	21.517
	rs704	A	G	-0.135	0.010	1.45E-39	0.473	174.898
	rs111072793	A	C	0.098	0.015	2.92E-11	0.202	44.169
	rs2042717	T	C	0.052	0.011	4.03E-06	0.594	21.389
	rs116888401	C	G	-0.241	0.038	1.60E-10	0.027	40.895
	rs8102855	C	T	-0.418	0.011	1.00E-200	0.505	1468.372
	rs4807575	C	T	0.163	0.011	5.27E-51	0.448	224.154
	rs73011399	C	T	-0.070	0.013	1.50E-07	0.246	27.443
	rs8110225	G	A	0.153	0.012	1.16E-40	0.436	177.916
	rs12625762	A	G	0.082	0.015	3.22E-08	0.138	30.506
	rs1537061	C	T	-0.025	0.003	2.55E-13	0.099	53.616
	rs73078376	A	C	-0.025	0.005	2.94E-08	0.056	30.795
	rs56043070	A	G	-0.026	0.004	6.04E-11	0.071	42.865
	rs11121246	G	T	0.013	0.002	4.59E-10	0.502	38.905
	rs1804999	G	A	0.011	0.002	3.48E-08	0.506	30.473
	rs1086893	C	T	0.030	0.002	2.41E-43	0.343	190.815
	rs4060971	T	C	-0.021	0.002	1.85E-23	0.551	99.710
	rs12143614	A	T	-0.023	0.003	4.72E-13	0.117	52.395
	rs6671847	A	G	0.014	0.002	4.44E-11	0.508	43.478
	rs10927074	C	T	0.079	0.003	3.00E-127	0.892	576.230
	rs80194822	G	A	0.061	0.010	6.66E-09	0.011	33.683
	rs7515985	G	A	0.014	0.002	6.05E-11	0.416	42.865
	rs12075	A	G	0.028	0.002	6.02E-41	0.580	179.813
	rs17613339	T	C	-0.017	0.003	4.71E-08	0.128	29.888
	rs142405270	G	A	0.051	0.008	6.41E-10	0.019	38.250
	rs17625587	A	G	0.015	0.002	1.25E-10	0.264	41.461
	rs12123922	A	G	-0.028	0.002	1.73E-41	0.441	182.282
	rs13386606	A	G	-0.012	0.002	2.15E-08	0.358	31.399
	rs13419763	T	C	0.016	0.002	4.43E-15	0.562	61.599
	rs62105478	A	G	-0.032	0.005	1.09E-11	0.052	46.218
	rs6543144	G	A	0.014	0.002	7.43E-10	0.313	37.945
	rs6734238	G	A	0.012	0.002	7.37E-09	0.402	33.479
	rs7573465	T	G	0.015	0.002	6.26E-14	0.556	56.381
	rs73987603	A	G	0.024	0.003	2.64E-18	0.166	76.276
	rs2860773	T	G	0.019	0.002	5.03E-20	0.408	84.129
	rs2028900	T	C	-0.014	0.002	1.25E-11	0.449	45.968
	rs1598207	G	A	0.020	0.002	1.41E-19	0.303	82.037
	rs4602187	T	C	0.014	0.002	2.53E-10	0.683	40.052

rs79140637	A	G	-0.027	0.005	2.09E-09	0.054	35.942
rs62160676	C	T	-0.017	0.002	3.57E-13	0.296	52.942
rs1427499	G	A	0.021	0.002	1.57E-20	0.710	86.416
rs77785849	A	C	0.036	0.006	4.94E-10	0.033	38.755
rs12497690	C	A	0.015	0.002	6.48E-12	0.371	47.247
rs34850939	A	G	0.014	0.002	7.75E-10	0.349	37.886
rs11710737	G	A	0.012	0.002	2.15E-08	0.439	31.392
rs74535412	A	G	0.038	0.006	2.31E-11	0.035	44.749
rs13089722	A	G	-0.088	0.003	1.44E-156	0.108	710.930
rs4324460	G	T	0.024	0.003	6.15E-22	0.219	92.777
rs1669340	T	G	-0.034	0.003	3.09E-34	0.839	149.006
rs7613595	C	A	-0.036	0.002	1.55E-48	0.218	214.563
rs3181077	T	C	-0.037	0.002	2.23E-59	0.718	264.261
rs2089979	G	A	-0.014	0.002	2.59E-11	0.416	44.538
rs59107033	T	C	-0.014	0.002	1.17E-08	0.225	32.589
rs9819371	T	C	-0.030	0.004	7.05E-13	0.065	51.606
rs6780544	A	G	-0.016	0.002	9.32E-15	0.623	60.139
rs10006833	C	T	-0.016	0.003	6.22E-10	0.209	38.321
rs7684939	A	G	-0.012	0.002	7.35E-09	0.489	33.502
rs2298989	C	T	-0.012	0.002	2.65E-08	0.425	30.996
rs28647824	A	C	-0.018	0.002	3.84E-14	0.267	57.345
rs11097787	T	C	-0.015	0.002	3.83E-12	0.403	48.278
rs56406125	T	G	-0.035	0.003	6.04E-41	0.190	179.685
rs4912807	C	G	0.013	0.002	1.02E-09	0.617	37.333
rs357613	A	G	-0.015	0.002	3.26E-12	0.507	48.596
rs2594836	A	G	-0.018	0.002	1.87E-14	0.722	58.764
rs13188960	T	G	-0.019	0.002	5.08E-17	0.279	70.426
rs112352373	G	C	-0.014	0.003	4.71E-08	0.203	29.886
rs3857286	T	C	-0.012	0.002	3.97E-08	0.695	30.198
rs34500	A	G	-0.026	0.004	1.67E-12	0.084	49.911
rs2271352	C	G	0.042	0.002	1.44E-65	0.226	292.797
rs4705908	A	G	-0.012	0.002	2.43E-08	0.420	31.154
rs377763	A	C	-0.027	0.002	9.38E-28	0.214	119.324
rs915125	T	C	-0.029	0.002	2.18E-37	0.281	163.477
rs35045014	A	C	-0.011	0.002	3.87E-08	0.456	30.268
rs2524079	A	G	0.031	0.002	5.46E-51	0.419	225.780
rs10806232	T	A	-0.015	0.002	2.17E-12	0.598	49.401
rs11756802	A	T	-0.029	0.005	2.80E-09	0.048	35.373
rs6927569	C	T	0.022	0.002	4.22E-26	0.523	111.868
rs1100578	C	G	0.022	0.002	2.02E-27	0.543	117.874
rs9274351	A	T	0.020	0.003	2.79E-12	0.197	48.911

rs2229092	C	A	0.026	0.004	1.48E-09	0.062	36.610
rs905670	A	G	-0.014	0.002	2.49E-11	0.350	44.612
rs9376098	A	T	0.023	0.002	4.15E-27	0.349	116.432
rs1186222	T	C	-0.022	0.002	3.21E-26	0.462	112.347
rs74524365	T	A	0.023	0.004	1.54E-08	0.070	32.054
rs2282986	C	T	-0.089	0.008	1.98E-30	0.018	131.598
rs71562671	C	T	0.021	0.004	4.84E-08	0.079	29.830
rs2158799	G	C	0.014	0.002	2.57E-11	0.610	44.567
rs56388170	T	G	0.031	0.002	3.34E-43	0.293	190.153
rs42030	A	G	0.030	0.004	6.09E-15	0.076	60.976
rs73049252	A	G	-0.037	0.004	2.42E-17	0.059	71.855
rs149007767	T	C	0.027	0.003	3.48E-20	0.162	84.790
rs3731332	T	C	-0.104	0.007	5.36E-49	0.022	216.690
rs182090955	A	G	-0.088	0.011	2.62E-16	0.010	67.166
rs56179563	A	G	0.015	0.002	6.10E-12	0.389	47.359
rs11772895	C	G	0.034	0.002	9.23E-50	0.278	220.160
rs55690609	A	G	-0.035	0.002	8.93E-57	0.323	252.231
rs45577137	G	A	-0.045	0.005	2.08E-17	0.045	72.168
rs7819602	G	C	0.016	0.002	1.98E-14	0.613	58.640
rs2738104	A	G	0.018	0.002	4.01E-17	0.672	70.907
rs6557615	A	G	-0.019	0.002	1.90E-17	0.681	72.334
rs4876400	G	A	-0.017	0.002	6.00E-16	0.626	65.509
rs7832357	G	A	-0.015	0.002	8.90E-13	0.342	51.131
rs2977799	G	A	0.027	0.002	1.17E-40	0.509	178.417
rs16923637	T	G	0.014	0.002	4.79E-10	0.286	38.818
rs6993770	T	A	-0.018	0.002	7.50E-16	0.287	65.109
rs13267723	A	G	-0.014	0.002	8.46E-09	0.232	33.224
rs2811708	T	G	-0.017	0.002	1.32E-13	0.266	54.906
rs12376511	C	T	-0.021	0.003	1.03E-13	0.163	55.387
rs1633768	T	C	-0.017	0.002	7.02E-13	0.275	51.620
rs2273770	T	C	0.012	0.002	4.16E-08	0.399	30.131
rs7044519	G	T	0.017	0.003	2.37E-09	0.161	35.700
rs2150052	T	A	-0.013	0.002	8.76E-11	0.506	42.152
rs10906375	G	A	-0.012	0.002	2.29E-08	0.301	31.268
rs2998286	C	T	0.021	0.002	1.31E-17	0.770	73.070
rs2805921	A	G	0.013	0.002	4.92E-08	0.248	29.791
rs3071	C	A	-0.016	0.002	2.95E-13	0.298	53.324
rs34377578	C	A	0.013	0.002	1.56E-08	0.252	32.023
rs748113	C	T	-0.012	0.002	2.19E-08	0.436	31.363
rs72786903	T	C	-0.014	0.002	1.48E-08	0.222	32.117
rs7078507	G	A	-0.016	0.002	2.54E-14	0.609	58.177

rs10883359	G	A	-0.013	0.002	7.30E-09	0.285	33.515
rs1539174	G	C	0.046	0.002	1.51E-82	0.237	370.649
rs71521681	A	C	0.024	0.002	1.23E-29	0.403	128.004
rs11187969	A	G	-0.017	0.003	4.24E-08	0.130	30.077
rs3781452	T	C	0.020	0.002	1.34E-20	0.632	86.740
rs695113	T	C	0.017	0.002	3.93E-14	0.696	57.292
rs10734121	A	G	-0.025	0.003	3.28E-19	0.845	80.360
rs2606724	A	G	0.016	0.002	1.33E-13	0.453	54.874
rs6421984	C	T	0.018	0.002	6.97E-19	0.514	78.873
rs10835333	G	A	0.015	0.002	1.68E-11	0.349	45.364
rs2276066	G	A	0.014	0.002	9.38E-11	0.648	42.011
rs74472890	C	T	0.103	0.005	1.02E-103	0.049	467.925
rs10893844	C	G	-0.015	0.002	4.22E-14	0.501	57.165
rs16928084	G	A	-0.028	0.003	1.24E-17	0.113	73.181
rs742631	T	C	-0.017	0.002	4.83E-13	0.284	52.353
rs10896064	C	G	0.013	0.002	8.01E-11	0.532	42.313
rs17860282	T	C	0.105	0.010	2.62E-26	0.011	112.763
rs2286599	A	G	0.023	0.003	1.15E-14	0.142	59.708
rs2118140	G	A	0.018	0.002	4.03E-19	0.528	79.942
rs10844657	T	C	0.012	0.002	1.18E-08	0.340	32.571
rs75084335	G	C	0.028	0.004	3.75E-12	0.069	48.325
rs4475963	G	T	-0.014	0.002	2.11E-10	0.370	40.427
rs4763817	T	C	0.013	0.002	1.18E-08	0.739	32.566
rs146970669	A	G	0.022	0.004	2.43E-10	0.094	40.147
rs10746147	G	A	-0.022	0.004	1.20E-08	0.926	32.534
rs11064881	A	G	-0.032	0.004	2.05E-16	0.074	67.656
rs3184504	C	T	-0.028	0.002	5.08E-43	0.519	189.313
rs76428106	C	T	0.082	0.009	2.78E-18	0.013	76.147
rs3892360	G	C	0.043	0.006	4.46E-13	0.307	52.514
rs4941839	A	G	-0.013	0.002	5.43E-09	0.714	34.088
rs2239635	C	G	0.034	0.002	1.89E-52	0.704	232.551
rs72721631	A	C	-0.019	0.002	1.60E-15	0.237	63.604
rs11158159	C	G	0.028	0.003	4.59E-28	0.770	120.785
rs34638654	C	G	0.012	0.002	1.66E-08	0.354	31.922
rs2289511	A	G	-0.016	0.002	3.30E-15	0.582	62.180
rs72697295	C	G	-0.018	0.003	8.00E-11	0.181	42.337
rs2074585	A	G	-0.031	0.002	2.71E-51	0.515	227.111
rs7496362	G	C	-0.013	0.002	2.71E-09	0.366	35.423
rs11633707	A	G	0.012	0.002	6.66E-09	0.636	33.674
rs67175901	T	C	0.026	0.003	8.24E-15	0.108	60.367
rs28364390	A	T	0.020	0.003	1.69E-11	0.132	45.383

rs2070596	A	T	0.023	0.003	3.30E-19	0.211	80.389
rs62021606	G	T	-0.034	0.005	2.00E-13	0.051	54.076
rs12443468	G	A	0.015	0.002	3.04E-09	0.226	35.211
rs7196129	C	T	0.017	0.002	7.19E-16	0.529	65.181
rs247833	A	G	0.016	0.002	1.42E-11	0.249	45.715
rs9928015	T	G	-0.015	0.002	2.01E-11	0.302	45.033
rs875740	A	C	-0.018	0.002	1.52E-16	0.665	68.252
rs12921873	T	C	0.016	0.003	5.19E-10	0.211	38.667
rs12447180	C	T	0.023	0.002	2.06E-26	0.324	113.223
rs71391089	T	G	0.016	0.003	5.34E-09	0.170	34.113
rs7503461	T	C	-0.013	0.002	9.60E-10	0.319	37.448
rs12941811	C	T	-0.046	0.002	7.71E-111	0.578	500.949
rs1295927	G	A	-0.025	0.002	5.83E-34	0.448	147.702
rs12453682	T	C	-0.018	0.002	3.60E-16	0.695	66.524
rs34097845	T	C	0.043	0.005	4.58E-21	0.057	88.838
rs7420	T	C	-0.013	0.002	1.81E-10	0.538	40.709
rs8178414	T	C	0.074	0.009	4.48E-16	0.013	66.104
rs17758695	T	C	-0.125	0.006	1.47E-91	0.030	412.078
rs561102	T	C	-0.014	0.002	8.12E-11	0.655	42.292
rs2959356	G	A	0.014	0.002	4.14E-10	0.298	39.122
rs602229	C	G	0.013	0.002	1.03E-08	0.693	32.841
rs76427287	C	T	-0.029	0.002	2.07E-31	0.254	136.120
rs2007774	A	T	0.016	0.002	2.36E-12	0.278	49.244
rs2967595	T	C	-0.026	0.003	5.59E-21	0.168	88.414
rs73022294	T	C	-0.057	0.010	1.29E-08	0.011	32.401
rs12459419	T	C	-0.017	0.002	2.44E-14	0.323	58.207
rs7250849	T	G	0.022	0.003	1.01E-10	0.107	41.860
rs2607278	T	C	0.023	0.002	1.79E-24	0.306	104.370
rs8113682	G	T	-0.022	0.002	6.41E-20	0.747	83.578
rs118013485	A	G	0.024	0.004	1.65E-08	0.067	31.918
rs78744187	T	C	-0.130	0.004	1.00E-200	0.082	1201.225
rs7253094	C	G	-0.016	0.002	5.51E-12	0.671	47.584
rs1128099	A	G	-0.015	0.002	1.41E-10	0.285	41.224
rs34158728	A	G	0.095	0.006	3.24E-53	0.028	235.984
rs6045612	T	C	-0.017	0.002	7.18E-13	0.264	51.581
rs6091176	T	C	0.014	0.002	4.80E-09	0.261	34.315
rs73110111	G	A	-0.037	0.007	2.29E-08	0.027	31.277
rs6141781	T	C	0.033	0.003	4.12E-29	0.147	125.600
rs6029234	C	G	0.013	0.002	3.46E-09	0.626	34.961
rs16989483	C	T	-0.021	0.002	3.15E-20	0.274	85.023
rs4911102	T	C	-0.022	0.002	6.46E-19	0.234	79.049

Eosinophil count	rs310631	A	C	0.012	0.002	4.42E-09	0.412	34.495
	rs138595256	G	C	0.064	0.007	1.82E-20	0.026	86.109
	rs2834670	G	A	0.028	0.003	1.69E-18	0.172	77.115
	rs2836241	C	T	0.014	0.002	2.37E-11	0.405	44.690
	rs7285377	T	G	0.014	0.002	1.86E-10	0.284	40.666
	rs5750494	G	T	-0.013	0.002	6.46E-10	0.629	38.246
	rs34288539	T	C	-0.015	0.002	6.34E-11	0.274	42.793
	rs34780507	G	A	0.035	0.004	2.96E-17	0.066	71.478
	rs2294199	C	T	-0.016	0.002	1.81E-15	0.470	63.361
	rs9430574	A	G	-0.020	0.002	2.35E-20	0.325	85.614
	rs35249183	G	A	0.040	0.003	1.46E-32	0.100	141.322
	rs3218148	A	G	-0.018	0.002	7.08E-19	0.540	78.836
	rs77625297	C	G	-0.046	0.006	2.79E-16	0.035	67.049
	rs111759324	T	C	-0.043	0.003	3.56E-42	0.111	185.373
	rs1414517	G	C	-0.025	0.003	1.36E-22	0.187	95.789
	rs4148757	C	A	-0.017	0.002	2.24E-12	0.210	49.345
	rs61798836	C	T	0.015	0.003	3.75E-08	0.170	30.323
	rs146078144	A	G	0.086	0.010	2.00E-16	0.010	67.705
	rs34448954	T	C	-0.025	0.003	3.87E-15	0.106	61.851
	rs1099448	T	C	0.018	0.002	8.32E-19	0.466	78.497
	rs17849501	T	C	-0.027	0.004	2.10E-09	0.052	35.933
	rs1036332	C	A	-0.033	0.002	1.61E-47	0.737	209.857
	rs4908835	C	T	0.019	0.003	1.80E-12	0.161	49.780
	rs2282718	A	G	-0.011	0.002	3.95E-08	0.373	30.213
	rs6670664	A	T	-0.015	0.002	7.38E-13	0.663	51.519
	rs7556106	A	G	-0.033	0.004	1.57E-16	0.064	68.200
	rs1004870	T	C	0.026	0.002	1.27E-38	0.589	169.025
	rs71628184	T	C	0.021	0.003	8.32E-10	0.094	37.736
	rs4652560	T	A	-0.015	0.002	5.23E-14	0.618	56.743
	rs17668272	T	G	-0.042	0.003	7.48E-42	0.118	183.870
	rs159963	A	C	0.019	0.002	9.88E-22	0.582	91.844
	rs12408934	A	G	-0.041	0.003	2.36E-36	0.103	158.690
	rs41313381	A	C	0.050	0.006	7.05E-19	0.031	78.865
	rs556063	G	A	0.013	0.002	1.68E-10	0.574	40.850
	rs2242193	A	G	0.012	0.002	1.20E-09	0.421	37.024
	rs10900595	A	C	0.015	0.002	1.59E-12	0.713	50.022
	rs4149909	G	A	-0.033	0.006	2.07E-09	0.033	35.970
	rs12097268	A	T	-0.013	0.002	3.23E-11	0.482	44.089
	rs2502995	C	T	0.022	0.002	1.06E-27	0.570	119.149
	rs4310436	A	G	0.019	0.003	9.15E-10	0.120	37.563
	rs6684992	T	A	0.038	0.003	6.06E-35	0.119	152.253

rs10782957	T	C	0.023	0.002	2.81E-28	0.622	121.820
rs201883150	T	C	-0.014	0.002	6.41E-10	0.722	38.252
rs9428321	C	T	0.016	0.002	8.56E-13	0.667	51.222
rs11204682	T	G	-0.041	0.002	4.89E-67	0.223	299.558
rs6672031	G	C	0.020	0.002	1.90E-23	0.486	99.652
rs11120043	T	C	0.011	0.002	4.93E-08	0.608	29.798
rs708776	T	G	0.027	0.004	2.91E-10	0.941	39.796
rs62105489	T	C	-0.030	0.004	1.13E-11	0.053	46.152
rs11695281	T	C	-0.023	0.002	4.09E-31	0.514	134.665
rs778756	G	A	-0.016	0.002	1.57E-15	0.580	63.609
rs7593992	C	T	0.030	0.002	1.52E-38	0.768	168.689
rs10195713	T	C	0.025	0.003	2.74E-17	0.864	71.619
rs6741307	T	C	0.014	0.002	6.32E-11	0.640	42.773
rs2713548	T	C	0.013	0.002	1.02E-09	0.633	37.355
rs17682575	T	C	-0.018	0.002	1.33E-12	0.201	50.349
rs11684770	G	A	-0.015	0.002	3.10E-11	0.270	44.173
rs11888323	G	A	-0.017	0.002	4.18E-13	0.778	52.643
rs13033333	G	C	0.012	0.002	4.62E-09	0.323	34.399
rs1427499	G	A	0.019	0.002	1.30E-17	0.710	73.113
rs76908370	G	A	-0.032	0.006	6.57E-09	0.036	33.702
rs62183994	T	C	0.027	0.005	2.65E-09	0.051	35.476
rs35409523	A	G	0.050	0.004	1.43E-40	0.076	178.080
rs4675190	T	C	-0.012	0.002	7.57E-09	0.591	33.453
rs80066203	T	C	-0.024	0.004	8.53E-10	0.068	37.689
rs113542380	A	G	-0.027	0.004	1.55E-12	0.075	50.049
rs11886928	G	T	-0.014	0.002	1.86E-11	0.308	45.197
rs10208293	A	G	-0.079	0.002	1.00E-200	0.287	1302.887
rs140509806	A	G	0.099	0.012	8.74E-16	0.009	64.789
rs1257192	G	A	0.015	0.003	8.81E-09	0.812	33.147
rs925966	G	C	-0.018	0.002	1.32E-14	0.759	59.438
rs79716587	A	G	-0.024	0.003	3.99E-16	0.127	66.317
rs7423615	T	C	0.023	0.003	5.10E-20	0.187	84.031
rs11495865	A	G	0.028	0.004	1.21E-14	0.087	59.596
rs346835	T	C	-0.027	0.002	7.55E-37	0.328	160.918
rs11890306	T	C	0.013	0.002	1.91E-10	0.423	40.623
rs10165678	A	G	-0.015	0.002	1.90E-10	0.758	40.639
rs7591231	G	C	0.013	0.002	1.30E-08	0.238	32.379
rs7569084	T	C	0.019	0.002	1.19E-20	0.584	86.965
rs699664	T	C	-0.016	0.002	1.00E-13	0.330	55.468
rs10174238	A	G	0.013	0.002	3.41E-08	0.766	30.498
rs1047891	A	C	0.017	0.002	1.55E-15	0.315	63.632

rs6750754	G	T	-0.064	0.002	8.36E-179	0.264	813.556
rs1057258	T	C	-0.032	0.003	2.52E-35	0.178	153.974
rs34290285	A	G	-0.046	0.002	4.73E-91	0.257	409.690
rs2579506	A	G	0.047	0.002	1.17E-120	0.386	545.629
rs4849748	C	T	-0.014	0.003	1.09E-08	0.267	32.714
rs1406449	C	T	-0.012	0.002	8.69E-10	0.462	37.662
rs10930337	T	C	0.015	0.002	2.03E-11	0.285	45.034
rs6731125	C	T	0.017	0.002	1.04E-17	0.565	73.520
rs1672753	T	C	-0.017	0.003	6.74E-12	0.811	47.174
rs9872485	G	T	-0.018	0.003	9.26E-12	0.164	46.554
rs9840310	G	A	-0.013	0.002	1.30E-08	0.735	32.387
rs2399441	C	T	-0.019	0.002	8.08E-20	0.351	83.163
rs112255429	A	G	-0.046	0.008	7.05E-09	0.017	33.576
rs73205140	C	T	0.037	0.003	2.91E-30	0.105	130.858
rs900382	T	C	-0.051	0.006	1.00E-16	0.027	69.061
rs4680250	G	C	-0.016	0.002	4.85E-13	0.693	52.331
rs3950296	G	C	0.024	0.002	8.30E-25	0.244	105.900
rs7636495	A	G	-0.024	0.003	1.84E-20	0.181	86.093
rs6787336	A	G	0.036	0.002	3.29E-59	0.287	263.590
rs12487980	A	C	0.013	0.002	1.50E-09	0.634	36.587
rs1131199	G	C	-0.025	0.002	9.55E-36	0.530	155.953
rs2942062	G	A	-0.023	0.004	2.65E-08	0.937	30.992
rs115697464	C	T	-0.065	0.008	1.61E-15	0.015	63.590
rs76830965	A	C	-0.023	0.003	3.27E-14	0.118	57.642
rs4074672	T	C	0.015	0.002	1.48E-12	0.369	50.140
rs9815874	T	C	0.028	0.002	1.93E-39	0.300	172.854
rs9818987	T	C	0.014	0.002	2.60E-10	0.306	40.023
rs2920505	A	G	-0.030	0.002	9.47E-49	0.602	215.575
rs13073683	C	T	0.016	0.002	1.32E-14	0.399	59.422
rs7646283	T	C	0.026	0.002	2.33E-36	0.368	158.786
rs73072498	C	T	0.060	0.010	9.63E-09	0.009	32.966
rs7646695	T	C	0.018	0.003	1.82E-11	0.180	45.213
rs2683913	G	A	-0.015	0.003	8.68E-09	0.827	33.157
rs1353286	G	T	0.020	0.002	3.09E-24	0.453	103.303
rs7649812	G	C	0.016	0.002	3.74E-12	0.243	48.328
rs4857909	G	A	0.121	0.003	1.00E-200	0.880	1563.493
rs73238201	T	C	-0.016	0.003	1.52E-10	0.179	41.053
rs73046033	G	T	-0.022	0.003	3.29E-12	0.117	48.584
rs2089979	G	A	-0.015	0.002	1.25E-13	0.416	55.016
rs9837045	A	G	0.011	0.002	1.41E-08	0.455	32.233
rs155266	A	T	0.019	0.002	1.68E-20	0.571	86.286

rs2228467	C	T	0.062	0.004	8.83E-52	0.062	229.450
rs9835307	C	T	0.026	0.002	2.69E-33	0.658	144.681
rs4618204	C	T	0.018	0.002	1.09E-19	0.444	82.572
rs1516527	C	T	-0.032	0.005	2.25E-12	0.951	49.317
rs1828803	A	C	0.013	0.002	1.28E-10	0.392	41.388
rs7687708	G	T	-0.015	0.002	1.56E-09	0.220	36.514
rs1479918	T	A	-0.019	0.002	1.05E-16	0.260	68.961
rs13139941	G	A	-0.014	0.002	2.84E-08	0.803	30.869
rs7441808	G	A	0.014	0.002	1.22E-10	0.301	41.474
rs62308111	T	G	0.015	0.002	1.50E-10	0.221	41.099
rs13105682	G	T	-0.027	0.004	8.22E-10	0.060	37.770
rs113473633	G	A	-0.063	0.007	3.47E-22	0.026	93.952
rs11931711	T	C	0.019	0.002	3.48E-18	0.282	75.689
rs76981581	G	C	-0.026	0.005	2.22E-08	0.048	31.344
rs13138355	T	C	-0.055	0.003	2.35E-102	0.186	461.852
rs2566133	C	T	-0.014	0.002	1.06E-12	0.584	50.823
rs13120371	G	A	0.015	0.002	3.00E-13	0.327	53.273
rs1365623	T	C	0.012	0.002	1.71E-08	0.372	31.857
rs73232881	C	T	0.067	0.002	8.22E-171	0.213	776.695
rs746550	T	C	0.023	0.002	9.03E-23	0.236	96.579
rs16903574	G	C	0.029	0.004	1.17E-13	0.078	55.149
rs34495	T	G	-0.020	0.002	3.67E-20	0.304	84.674
rs10059018	T	G	-0.023	0.002	3.38E-21	0.201	89.428
rs60646393	A	G	-0.024	0.004	2.45E-09	0.066	35.631
rs6556313	G	A	0.019	0.002	2.16E-20	0.332	85.769
rs10472984	G	C	-0.035	0.002	7.62E-63	0.340	280.201
rs139640694	G	A	0.025	0.003	3.54E-13	0.093	52.967
rs79881201	T	C	0.040	0.002	4.72E-85	0.360	382.075
rs73272842	A	G	-0.025	0.003	4.84E-17	0.123	70.529
rs2431097	T	C	0.015	0.002	5.95E-14	0.486	56.479
rs4703730	T	C	-0.016	0.002	6.91E-16	0.517	65.248
rs244689	G	A	-0.021	0.003	2.46E-12	0.877	49.139
rs3756374	T	G	0.017	0.003	3.71E-08	0.119	30.343
rs10062687	G	T	0.024	0.002	9.47E-25	0.233	105.654
rs66735324	G	A	-0.014	0.002	7.30E-09	0.221	33.510
rs152197	T	C	0.050	0.003	5.48E-82	0.175	368.079
rs12515180	T	C	-0.072	0.002	1.00E-200	0.426	1294.342
rs4703589	C	T	0.014	0.002	3.26E-13	0.533	53.107
rs2568928	G	A	0.015	0.002	4.38E-11	0.757	43.492
rs7700687	T	C	0.040	0.002	4.82E-86	0.617	386.802
rs56330463	C	T	0.039	0.002	5.38E-86	0.553	386.646

rs62385501	A	T	-0.014	0.002	1.68E-11	0.308	45.364
rs9392525	C	T	0.013	0.002	6.95E-11	0.576	42.597
rs3812206	T	C	-0.012	0.002	2.07E-08	0.300	31.485
rs7382061	C	T	-0.055	0.002	1.13E-161	0.591	734.396
rs6924350	C	A	0.050	0.003	1.76E-86	0.184	388.774
rs62395833	C	G	0.037	0.005	5.27E-14	0.043	56.721
rs28383314	C	T	0.062	0.002	6.38E-199	0.624	905.718
rs6930635	C	T	-0.038	0.004	6.83E-24	0.078	101.729
rs7764278	T	G	0.017	0.002	1.84E-12	0.205	49.715
rs783646	C	G	-0.019	0.003	2.41E-13	0.817	53.701
rs12209219	C	T	-0.025	0.002	8.13E-37	0.475	160.862
rs6924387	G	A	0.017	0.002	2.19E-16	0.410	67.544
rs149110519	T	C	0.038	0.005	1.39E-12	0.036	50.279
rs3093023	A	G	0.013	0.002	2.27E-10	0.434	40.257
rs2788211	C	T	0.016	0.003	4.79E-10	0.817	38.809
rs1611236	A	G	-0.043	0.002	8.34E-91	0.323	408.457
rs73428834	T	C	0.030	0.004	2.72E-15	0.075	62.545
rs9349322	C	A	0.022	0.003	4.04E-15	0.147	61.765
rs2025489	G	A	0.016	0.002	2.42E-15	0.526	62.791
rs62408224	G	A	-0.042	0.002	5.69E-92	0.350	413.992
rs12208103	T	C	-0.031	0.002	6.83E-50	0.378	220.685
rs9389268	G	A	-0.045	0.002	3.69E-87	0.256	391.786
rs113496608	A	G	-0.045	0.006	1.13E-14	0.030	59.746
rs6904506	C	T	-0.052	0.004	2.25E-49	0.088	218.445
rs3846855	A	G	0.028	0.003	5.01E-28	0.183	120.631
rs2894401	G	A	-0.021	0.002	6.16E-21	0.705	88.215
rs526369	G	A	0.019	0.003	3.39E-11	0.143	44.023
rs13207791	G	A	0.026	0.004	4.30E-13	0.089	52.567
rs56195338	A	G	-0.030	0.004	1.88E-12	0.058	49.678
rs71525665	A	G	-0.019	0.002	4.15E-20	0.363	84.431
rs60600003	G	T	0.043	0.003	3.05E-38	0.100	167.318
rs12154498	C	A	-0.033	0.003	7.76E-31	0.854	133.448
rs116888884	A	G	-0.058	0.010	1.54E-08	0.010	32.052
rs7797428	T	C	0.012	0.002	5.85E-10	0.469	38.429
rs12530946	G	A	0.043	0.002	3.99E-97	0.613	437.532
rs1037674	T	G	0.013	0.002	1.63E-09	0.292	36.418
rs55879743	T	C	0.078	0.004	7.07E-82	0.066	367.528
rs12705849	A	G	-0.021	0.002	2.55E-24	0.407	103.664
rs12540285	G	A	0.015	0.002	1.01E-09	0.221	37.365
rs73086541	A	C	-0.012	0.002	7.07E-09	0.395	33.560
rs13226583	T	A	-0.062	0.003	1.59E-88	0.116	398.258

rs8	T	C	0.022	0.003	7.63E-18	0.204	74.176
rs3823536	A	G	-0.018	0.002	3.16E-19	0.467	80.493
rs3110791	C	T	0.015	0.002	7.49E-14	0.623	56.006
rs6956283	T	C	-0.016	0.002	2.48E-12	0.733	49.131
rs10243429	C	T	0.017	0.002	8.82E-15	0.290	60.258
rs6979947	G	A	0.013	0.002	3.75E-09	0.266	34.790
rs6971710	A	G	0.028	0.002	2.75E-30	0.201	130.975
rs57834782	A	T	-0.058	0.002	7.78E-141	0.245	638.842
rs4722171	G	A	-0.027	0.002	1.83E-41	0.592	182.202
rs10275896	C	T	-0.014	0.002	2.32E-09	0.244	35.735
rs150640087	T	G	0.100	0.008	1.80E-35	0.016	154.670
rs73118830	C	T	-0.029	0.004	1.08E-16	0.086	68.902
rs62473720	G	A	0.014	0.002	2.64E-11	0.317	44.488
rs56179563	A	G	0.018	0.002	3.87E-18	0.389	75.516
rs4240624	A	G	-0.020	0.003	3.62E-09	0.909	34.872
rs295	C	A	0.016	0.002	1.16E-11	0.235	46.108
rs62539154	A	G	0.027	0.003	7.20E-16	0.097	65.171
rs6986109	T	G	-0.016	0.002	4.24E-14	0.704	57.137
rs11786536	A	G	-0.027	0.003	5.11E-23	0.165	97.765
rs7840212	T	C	-0.040	0.002	7.11E-81	0.337	363.054
rs574183	G	A	-0.014	0.002	1.20E-11	0.389	46.028
rs6999452	A	G	0.012	0.002	5.33E-09	0.518	34.132
rs4870977	C	G	-0.019	0.003	2.40E-10	0.871	40.179
rs4236746	G	A	0.057	0.006	6.78E-19	0.975	78.940
rs117961539	A	G	-0.031	0.005	2.63E-09	0.040	35.494
rs12545733	C	T	-0.031	0.002	8.90E-45	0.703	197.294
rs2979489	A	G	0.014	0.002	1.33E-09	0.742	36.831
rs6989099	C	T	-0.018	0.002	8.58E-17	0.317	69.350
rs10100356	A	G	-0.013	0.002	1.91E-08	0.227	31.618
rs4739140	T	C	-0.015	0.002	1.53E-12	0.611	50.060
rs12681644	T	C	-0.019	0.002	1.23E-15	0.212	64.125
rs34173062	A	G	0.055	0.004	1.55E-40	0.073	177.857
rs7839946	C	T	0.024	0.004	2.00E-08	0.057	31.541
rs45577137	G	A	-0.060	0.005	5.10E-31	0.045	134.275
rs7846314	T	A	-0.033	0.003	3.92E-38	0.187	166.879
rs7009579	G	A	-0.012	0.002	1.22E-09	0.429	37.006
rs13251643	T	A	0.030	0.005	3.54E-10	0.044	39.417
rs4366169	C	A	0.012	0.002	8.70E-09	0.310	33.171
rs7026022	C	A	0.012	0.002	3.38E-09	0.378	35.003
rs72766638	A	C	-0.024	0.003	9.94E-20	0.164	82.719
rs884634	C	T	0.012	0.002	6.83E-10	0.415	38.118

rs496475	G	T	0.035	0.002	1.83E-66	0.387	296.675
rs911603	A	C	-0.025	0.002	1.42E-33	0.404	145.944
rs466547	G	A	0.019	0.003	1.95E-11	0.858	45.082
rs72695582	A	G	-0.050	0.006	2.40E-17	0.030	71.888
rs6479336	A	T	-0.029	0.003	3.44E-30	0.185	130.465
rs114741563	C	T	0.120	0.012	2.94E-25	0.008	107.960
rs74612091	A	T	0.061	0.004	1.23E-49	0.063	219.612
rs7868130	T	C	0.032	0.002	3.35E-46	0.259	203.775
rs3731211	A	T	0.018	0.002	2.34E-15	0.720	62.855
rs2095044	C	T	-0.094	0.002	1.00E-200	0.743	1717.098
rs295273	A	G	0.021	0.002	1.50E-19	0.256	81.897
rs143491704	G	C	-0.031	0.006	1.47E-08	0.036	32.146
rs33982662	A	C	0.020	0.002	1.19E-20	0.285	86.909
rs17482472	A	G	-0.031	0.003	5.34E-21	0.099	88.520
rs4746153	C	G	0.016	0.003	8.58E-10	0.185	37.669
rs7897422	C	T	0.021	0.003	1.85E-16	0.192	67.864
rs2497318	T	C	-0.030	0.002	1.96E-49	0.443	218.570
rs2505521	T	C	-0.015	0.003	1.45E-08	0.829	32.165
rs495149	T	C	0.024	0.003	1.15E-19	0.164	82.413
rs2419313	A	G	-0.021	0.003	5.21E-13	0.143	52.193
rs1539174	G	C	0.038	0.002	1.12E-59	0.237	265.764
rs1323650	T	G	-0.014	0.002	2.53E-10	0.681	40.058
rs10995240	C	G	-0.044	0.002	1.55E-101	0.368	457.737
rs2807740	T	C	0.035	0.002	1.05E-50	0.770	224.427
rs72834751	T	C	-0.072	0.009	1.50E-14	0.013	59.192
rs71508968	A	G	0.036	0.006	1.28E-08	0.026	32.417
rs3747869	C	A	0.020	0.003	2.31E-09	0.901	35.743
rs7080536	A	G	-0.044	0.005	1.17E-18	0.043	77.854
rs11255507	G	T	0.020	0.003	1.05E-14	0.178	59.875
rs2646438	A	G	-0.021	0.002	1.20E-24	0.566	105.121
rs962993	T	C	-0.040	0.002	4.92E-90	0.422	405.072
rs2992333	A	G	-0.034	0.002	1.35E-63	0.599	283.609
rs634534	G	A	0.033	0.002	5.83E-61	0.542	271.424
rs77331334	A	G	0.038	0.007	4.12E-08	0.021	30.134
rs58833930	T	C	-0.023	0.003	1.03E-13	0.113	55.395
rs11024646	T	C	0.017	0.002	1.45E-12	0.219	50.215
rs4243951	G	A	-0.014	0.002	2.10E-09	0.756	35.933
rs473739	G	T	-0.014	0.002	7.07E-11	0.319	42.556
rs11236813	C	G	-0.030	0.003	4.78E-20	0.102	84.205
rs7123726	C	T	0.017	0.002	3.95E-12	0.210	48.210
rs9666598	G	C	-0.020	0.003	1.36E-12	0.843	50.319

rs520461	G	A	-0.014	0.002	9.08E-09	0.222	33.089
rs668248	C	G	-0.017	0.002	5.49E-16	0.616	65.685
rs214080	G	A	0.013	0.002	1.37E-10	0.580	41.282
rs34439695	T	C	-0.038	0.005	3.67E-12	0.035	48.365
rs174548	G	C	-0.023	0.002	4.08E-27	0.314	116.421
rs637064	T	C	0.024	0.002	5.43E-34	0.556	147.913
rs1059091	G	A	0.034	0.002	3.08E-56	0.321	249.834
rs3824867	G	A	0.017	0.002	1.77E-14	0.712	58.868
rs11228990	T	A	0.029	0.005	3.56E-09	0.045	34.899
rs7936434	C	G	0.043	0.002	4.08E-104	0.478	470.018
rs4409785	C	T	0.016	0.003	4.25E-10	0.172	39.050
rs964184	C	G	0.029	0.003	4.39E-23	0.867	98.032
rs10893844	C	G	0.025	0.002	3.49E-35	0.502	153.365
rs1689510	C	G	0.027	0.002	1.06E-36	0.338	160.287
rs10745763	T	G	0.013	0.002	2.41E-11	0.423	44.687
rs2255531	A	G	-0.016	0.002	3.39E-15	0.351	62.113
rs3759332	C	T	-0.012	0.002	7.54E-09	0.391	33.437
rs10876550	A	G	0.014	0.002	4.92E-12	0.559	47.781
rs1471816	A	G	0.011	0.002	3.94E-08	0.503	30.214
rs6490291	A	T	-0.066	0.006	1.11E-29	0.964	128.177
rs12581511	G	C	-0.018	0.003	2.07E-11	0.173	44.968
rs1861489	A	T	0.013	0.002	4.14E-08	0.782	30.136
rs73191842	G	T	0.054	0.007	4.90E-15	0.021	61.384
rs4931002	A	C	-0.022	0.002	1.73E-19	0.779	81.664
rs7299446	A	T	-0.012	0.002	6.24E-09	0.524	33.799
rs10777378	A	G	-0.022	0.002	1.28E-29	0.536	127.829
rs1265564	C	A	0.080	0.002	1.00E-200	0.443	1548.252
rs115647629	A	G	-0.067	0.007	6.69E-25	0.026	106.329
rs12820863	T	C	0.022	0.002	1.07E-25	0.352	110.005
rs1800692	G	A	-0.018	0.002	8.92E-19	0.588	78.373
rs12827988	G	A	-0.016	0.002	1.57E-15	0.457	63.654
rs3024971	G	T	-0.040	0.003	5.04E-36	0.107	157.182
rs28532037	A	G	-0.030	0.003	4.19E-18	0.907	75.353
rs12861824	C	T	-0.021	0.002	2.59E-18	0.275	76.267
rs7986796	T	G	0.024	0.002	4.91E-32	0.628	138.994
rs7327960	C	T	0.018	0.003	1.91E-11	0.827	45.118
rs34363176	G	C	0.013	0.002	8.43E-09	0.244	33.238
rs201798	A	G	0.020	0.002	5.71E-23	0.615	97.510
rs2182885	A	G	0.026	0.002	2.05E-38	0.605	168.181
rs12100034	A	G	-0.017	0.002	2.60E-16	0.359	67.218
rs71429414	A	G	-0.019	0.002	1.03E-13	0.196	55.382

rs17061503	A	G	0.036	0.002	8.04E-61	0.307	270.791
rs12882281	C	T	-0.014	0.002	7.37E-09	0.239	33.492
rs2239633	A	G	0.035	0.002	1.50E-68	0.484	306.531
rs7141943	G	A	0.016	0.002	7.00E-15	0.395	60.707
rs67856193	G	C	0.024	0.002	1.63E-27	0.308	118.284
rs8012643	T	C	0.015	0.002	1.91E-12	0.286	49.659
rs3742704	C	A	0.019	0.003	1.54E-08	0.092	32.048
rs11555542	C	T	0.064	0.004	2.12E-54	0.063	241.503
rs113859409	A	G	-0.029	0.005	4.75E-08	0.040	29.862
rs11628569	G	A	-0.017	0.002	4.70E-14	0.290	56.955
rs7158239	A	G	0.016	0.002	2.28E-14	0.395	58.349
rs12878610	C	T	-0.013	0.002	3.95E-11	0.476	43.710
rs117068593	T	C	-0.026	0.003	1.17E-24	0.190	105.266
rs7975	A	G	-0.012	0.002	1.17E-08	0.324	32.577
rs8020739	T	G	0.025	0.002	2.57E-32	0.644	140.184
rs941616	T	C	-0.012	0.002	6.69E-09	0.610	33.662
rs6573020	T	C	0.021	0.002	2.31E-24	0.433	103.840
rs1296535	T	C	-0.019	0.002	3.35E-15	0.777	62.134
rs175705	G	C	0.038	0.002	8.80E-67	0.719	298.275
rs62006172	A	G	-0.038	0.005	4.62E-14	0.042	56.971
rs34212866	G	A	0.020	0.002	5.74E-17	0.221	70.187
rs62005030	T	C	-0.017	0.003	4.26E-08	0.124	30.072
rs62011287	G	A	-0.013	0.002	1.32E-09	0.344	36.831
rs28362902	A	G	0.017	0.003	1.66E-08	0.133	31.908
rs8026803	C	T	-0.031	0.002	1.67E-43	0.261	191.440
rs7257	A	G	0.034	0.002	3.60E-63	0.567	281.725
rs11071559	T	C	-0.022	0.003	4.94E-14	0.131	56.824
rs13511	C	T	-0.016	0.002	4.13E-16	0.577	66.273
rs6496717	T	C	0.019	0.002	4.42E-16	0.760	66.134
rs7173571	C	T	0.014	0.002	2.40E-12	0.527	49.197
rs17293632	T	C	0.030	0.002	8.92E-37	0.236	160.639
rs74331768	A	G	0.019	0.003	7.49E-09	0.098	33.463
rs17689024	C	G	-0.014	0.002	2.31E-11	0.314	44.749
rs4280242	T	C	-0.033	0.002	1.03E-45	0.754	201.607
rs72800841	A	G	-0.016	0.002	5.63E-12	0.250	47.527
rs8050508	T	C	0.035	0.006	1.01E-09	0.031	37.367
rs3850107	G	C	-0.014	0.002	3.94E-10	0.294	39.210
rs11648292	A	C	-0.013	0.002	1.59E-09	0.693	36.491
rs61426394	C	G	-0.025	0.004	6.99E-10	0.067	38.090
rs1684578	T	G	0.016	0.002	5.15E-16	0.416	65.805
rs3096309	T	C	0.015	0.003	1.36E-08	0.824	32.304

rs121564	T	G	-0.017	0.002	1.26E-13	0.270	55.024
rs8044920	T	C	0.012	0.002	3.59E-09	0.376	34.884
rs301162	G	A	0.026	0.003	1.16E-20	0.853	86.962
rs17175830	A	G	0.032	0.002	4.13E-42	0.238	185.132
rs725613	G	T	-0.047	0.002	4.78E-116	0.356	524.852
rs3785356	T	C	0.030	0.002	1.70E-43	0.298	191.504
rs9939774	T	C	-0.029	0.002	1.98E-47	0.405	209.475
rs1120275	G	T	-0.024	0.002	7.53E-29	0.311	124.423
rs16956811	G	T	-0.029	0.004	1.75E-15	0.081	63.428
rs12928503	T	C	-0.049	0.007	1.92E-11	0.020	45.120
rs1170439	C	T	0.023	0.002	7.86E-21	0.779	87.757
rs35850753	T	C	0.044	0.007	2.14E-09	0.020	35.898
rs74480102	A	G	-0.083	0.005	2.52E-63	0.043	282.416
rs397187	C	T	-0.012	0.002	1.28E-09	0.430	36.890
rs145947882	C	A	-0.060	0.006	2.98E-21	0.026	89.673
rs11869228	T	C	0.021	0.003	3.45E-14	0.841	57.557
rs8076052	C	A	-0.018	0.002	4.33E-17	0.296	70.737
rs58745116	A	G	-0.015	0.002	8.21E-14	0.391	55.852
rs9894839	C	T	0.012	0.002	1.33E-08	0.635	32.356
rs12941068	A	G	-0.014	0.002	9.59E-10	0.286	37.456
rs35689157	A	G	-0.069	0.005	3.28E-37	0.035	162.672
rs62061733	G	A	-0.032	0.002	1.92E-42	0.229	186.638
rs180506	A	G	-0.023	0.002	5.82E-22	0.776	92.918
rs55868524	A	G	0.013	0.002	9.91E-11	0.606	41.908
rs9889262	A	T	0.028	0.002	4.18E-43	0.364	189.564
rs62086903	C	T	0.030	0.002	2.27E-37	0.232	163.388
rs7220649	T	C	-0.028	0.005	1.23E-08	0.043	32.495
rs9709216	G	T	0.018	0.002	1.21E-16	0.487	68.669
rs117577334	A	G	0.017	0.003	2.75E-09	0.144	35.397
rs34210653	A	G	-0.176	0.007	8.11E-140	0.021	633.920
rs11079340	C	T	0.012	0.002	7.49E-09	0.322	33.472
rs7215391	T	C	-0.013	0.002	2.49E-08	0.257	31.108
rs9675999	A	G	0.016	0.002	1.59E-15	0.627	63.588
rs8083368	A	G	0.015	0.002	5.99E-11	0.230	42.890
rs73963711	T	C	0.022	0.002	3.28E-19	0.211	80.358
rs57633475	G	A	-0.025	0.003	6.36E-16	0.122	65.404
rs954954	C	A	-0.035	0.003	3.57E-27	0.105	116.719
rs17758695	T	C	-0.122	0.006	1.32E-93	0.030	421.552
rs2847266	T	C	-0.014	0.002	1.22E-10	0.717	41.494
rs200766223	A	T	-0.015	0.002	1.10E-09	0.219	37.195
rs9965489	A	G	0.016	0.002	3.30E-16	0.533	66.728

rs75737219	A	C	0.052	0.009	1.79E-09	0.014	36.245
rs350836	T	C	-0.017	0.002	6.41E-15	0.710	60.854
rs410867	G	A	-0.059	0.002	4.12E-134	0.217	607.773
rs36084354	A	G	-0.045	0.003	1.17E-39	0.092	173.824
rs14713	A	G	-0.014	0.002	3.53E-08	0.205	30.438
rs140411271	A	G	0.030	0.005	1.03E-10	0.056	41.825
rs412884	C	T	0.058	0.002	2.22E-164	0.672	746.797
rs62117160	A	G	-0.048	0.005	2.15E-23	0.045	99.468
rs76793172	T	C	-0.042	0.003	8.07E-34	0.092	147.117
rs34466956	T	C	-0.014	0.002	2.60E-11	0.568	44.532
rs3786586	G	A	0.032	0.003	4.42E-31	0.155	134.593
rs117481629	C	T	0.036	0.006	8.25E-09	0.028	33.263
rs72987040	A	C	-0.022	0.003	4.07E-13	0.126	52.694
rs118013485	A	G	-0.034	0.004	1.54E-16	0.067	68.229
rs117955557	T	G	-0.030	0.002	1.44E-40	0.256	178.070
rs2053927	T	G	-0.014	0.003	1.02E-08	0.803	32.845
rs8113367	G	A	0.017	0.002	6.68E-17	0.632	69.864
rs2043293	A	C	-0.015	0.003	1.36E-08	0.826	32.284
rs1178016	T	C	0.011	0.002	1.17E-08	0.495	32.582
rs6141755	T	G	-0.017	0.002	8.30E-13	0.236	51.298
rs2253427	C	T	-0.014	0.002	8.68E-09	0.778	33.169
rs3746420	C	G	-0.032	0.004	2.26E-14	0.061	58.368
rs73322872	T	C	0.015	0.002	3.91E-10	0.242	39.198
rs7267752	C	T	0.021	0.004	1.04E-08	0.083	32.819
rs80054178	C	T	-0.082	0.007	8.57E-34	0.022	146.993
rs290430	T	C	-0.012	0.002	8.31E-09	0.345	33.253
rs6139104	T	A	-0.019	0.003	1.78E-08	0.093	31.760
rs3790163	G	A	-0.015	0.003	1.35E-09	0.792	36.794
rs6103572	C	T	-0.024	0.002	5.96E-27	0.729	115.689
rs28421324	T	A	0.036	0.003	1.57E-27	0.102	118.334
rs56117721	A	T	-0.104	0.004	6.64E-160	0.072	726.323
rs7354779	C	T	0.018	0.002	2.71E-15	0.271	62.562
rs2223043	G	A	0.019	0.002	3.23E-17	0.307	71.323
rs114152720	A	G	-0.036	0.006	8.08E-10	0.030	37.792
rs9979383	T	C	0.028	0.002	8.00E-43	0.630	188.394
rs11088236	T	C	0.018	0.002	5.17E-20	0.453	84.037
rs2838317	C	A	0.013	0.002	1.20E-10	0.550	41.549
rs11701475	C	T	0.015	0.002	1.06E-09	0.223	37.264
rs5747308	C	A	0.016	0.002	4.82E-15	0.505	61.441
rs9607764	C	G	0.016	0.002	5.39E-16	0.402	65.748
rs34505104	G	A	-0.026	0.002	1.22E-33	0.306	146.309

	rs11702918	T	C	-0.022	0.003	1.06E-11	0.103	46.290
	rs739427	C	G	-0.022	0.002	1.06E-29	0.512	128.322
	rs55955211	C	A	0.031	0.004	2.42E-16	0.074	67.337
	rs743002	C	T	-0.059	0.004	2.90E-48	0.066	213.297
	rs60175411	A	G	-0.030	0.003	1.25E-28	0.167	123.391
	rs145823386	A	G	0.061	0.010	3.81E-09	0.010	34.776
Lymphocyte count	rs114323011	G	A	0.029	0.005	3.53E-09	0.040	34.916
	rs12567173	C	T	0.013	0.002	1.78E-09	0.235	36.237
	rs1320562	C	G	0.013	0.002	8.86E-09	0.758	33.130
	rs12059564	T	C	0.016	0.003	7.96E-10	0.150	37.837
	rs12038019	C	G	-0.016	0.002	1.01E-14	0.268	59.969
	rs10494783	A	G	-0.054	0.004	8.95E-37	0.052	160.644
	rs4619033	G	A	-0.019	0.002	5.73E-19	0.268	79.259
	rs12745411	T	G	0.017	0.002	6.32E-18	0.600	74.516
	rs2294630	G	A	0.013	0.002	8.15E-12	0.493	46.806
	rs2282718	A	G	-0.018	0.002	3.25E-18	0.372	75.861
	rs12088882	T	C	0.034	0.002	1.30E-70	0.408	316.036
	rs10875371	T	C	-0.021	0.002	3.37E-27	0.418	116.790
	rs1493694	T	C	0.020	0.003	4.78E-11	0.108	43.339
	rs11800059	T	C	-0.026	0.002	3.88E-42	0.510	185.217
	rs12045893	T	C	0.027	0.002	9.70E-35	0.248	151.356
	rs113116201	C	T	0.041	0.007	1.93E-09	0.020	36.097
	rs4925547	T	A	0.012	0.002	4.55E-10	0.402	38.913
	rs72634819	T	C	-0.014	0.002	4.67E-10	0.264	38.872
	rs77954449	G	A	-0.037	0.006	9.78E-11	0.028	41.934
	rs7529794	T	G	0.016	0.002	7.57E-14	0.262	56.009
	rs2275902	C	G	-0.016	0.002	5.22E-15	0.298	61.240
	rs144520705	A	C	0.067	0.011	5.93E-09	0.008	33.907
	rs71519213	G	C	0.013	0.002	2.94E-11	0.565	44.300
	rs1894692	A	G	-0.038	0.007	1.12E-08	0.978	32.661
	rs12021809	A	G	-0.026	0.003	2.69E-18	0.110	76.194
	rs113466616	A	G	0.012	0.002	1.79E-09	0.333	36.257
	rs11247712	T	C	-0.020	0.002	3.38E-24	0.369	103.105
	rs6700896	T	C	-0.017	0.002	3.67E-19	0.379	80.176
	rs60633358	C	T	-0.022	0.003	7.19E-15	0.132	60.661
	rs12133917	A	C	0.016	0.002	2.15E-16	0.436	67.549
	rs72740550	A	G	-0.019	0.002	1.58E-16	0.222	68.155
	rs35993840	C	A	0.038	0.004	1.50E-23	0.070	100.141
	rs533483	A	G	-0.014	0.002	1.58E-10	0.244	40.982
	rs74227709	A	G	-0.029	0.004	2.51E-15	0.072	62.704
	rs7527389	G	A	-0.019	0.002	6.31E-23	0.631	97.323

rs9662343	C	G	-0.015	0.002	4.61E-12	0.737	47.914
rs61778219	T	C	0.023	0.003	2.67E-17	0.141	71.694
rs12142033	A	G	-0.013	0.002	1.73E-10	0.333	40.819
rs2294752	G	T	0.012	0.002	1.95E-08	0.238	31.598
rs4319365	A	G	-0.019	0.002	3.26E-24	0.545	103.176
rs12127284	T	C	-0.017	0.002	1.11E-15	0.710	64.306
rs2755253	T	C	-0.031	0.002	2.87E-50	0.709	222.444
rs6679677	A	C	-0.054	0.003	2.96E-66	0.100	295.894
rs716723	T	A	-0.017	0.003	7.76E-10	0.134	37.888
rs494091	C	T	0.027	0.002	6.35E-46	0.426	202.623
rs2489279	G	C	0.018	0.002	1.28E-18	0.334	77.690
rs7548606	G	A	0.016	0.002	9.26E-17	0.626	69.245
rs4660128	C	T	-0.025	0.003	3.12E-15	0.897	62.293
rs7572278	A	T	0.020	0.002	1.04E-17	0.207	73.550
rs35002856	C	G	0.012	0.002	4.03E-10	0.373	39.161
rs113542380	A	G	-0.068	0.004	4.90E-77	0.075	345.300
rs2542573	C	T	-0.015	0.002	1.52E-13	0.331	54.627
rs6546881	T	C	0.013	0.002	2.17E-10	0.657	40.353
rs4907236	T	C	0.011	0.002	4.41E-08	0.283	30.011
rs1976055	A	C	-0.017	0.003	4.05E-11	0.156	43.671
rs11688303	T	C	0.016	0.003	2.74E-08	0.120	30.931
rs10179705	A	G	-0.021	0.002	1.29E-24	0.294	104.976
rs10932018	A	G	-0.015	0.002	5.65E-12	0.730	47.517
rs4972950	A	C	0.022	0.003	1.42E-12	0.108	50.231
rs1260326	C	T	-0.024	0.002	1.44E-35	0.602	155.050
rs10865331	G	A	0.013	0.002	7.38E-11	0.625	42.469
rs6755786	T	C	0.027	0.002	4.20E-32	0.773	139.233
rs4849169	C	A	0.021	0.002	4.14E-28	0.482	120.976
rs73961715	C	T	-0.049	0.003	2.79E-67	0.128	300.664
rs10174056	A	T	0.014	0.002	6.38E-13	0.384	51.829
rs6743068	G	A	0.031	0.002	1.69E-49	0.730	218.872
rs6721663	A	G	-0.026	0.003	9.83E-24	0.159	101.025
rs17366643	C	T	0.015	0.002	1.35E-14	0.538	59.385
rs55982282	A	G	0.062	0.003	8.71E-110	0.135	495.995
rs4407213	C	G	0.042	0.006	1.49E-14	0.032	59.197
rs10191329	A	C	-0.014	0.003	1.39E-08	0.175	32.258
rs10206961	T	C	-0.011	0.002	3.10E-08	0.418	30.690
rs71420836	T	C	0.030	0.003	1.67E-19	0.097	81.685
rs148601577	G	A	-0.049	0.007	3.79E-12	0.020	48.306
rs34030812	C	T	-0.018	0.002	1.49E-20	0.367	86.530
rs6742799	C	A	-0.039	0.002	3.54E-62	0.200	277.150

	rs11684463	T	A	-0.047	0.002	1.30E-121	0.327	550.325
	rs2305591	T	A	0.011	0.002	8.67E-09	0.557	33.158
	rs11676298	G	C	0.027	0.002	2.86E-29	0.191	126.262
	rs2276566	C	G	-0.025	0.003	1.40E-15	0.100	63.873
	rs11688289	C	T	-0.015	0.002	6.98E-10	0.189	38.090
	rs2968811	T	A	0.013	0.002	7.76E-12	0.421	46.918
	rs1177268	G	A	-0.014	0.002	5.45E-12	0.566	47.592
	rs1533298	T	C	0.031	0.002	1.19E-49	0.715	219.632
	rs78394891	A	T	-0.026	0.004	1.14E-08	0.047	32.627
	rs12692735	T	G	-0.012	0.002	2.17E-10	0.361	40.367
	rs529002	G	T	-0.011	0.002	4.46E-09	0.551	34.465
	rs11897252	T	C	-0.014	0.002	4.69E-10	0.234	38.851
	rs1448318	A	G	0.018	0.002	5.49E-20	0.615	83.869
	rs4497886	A	G	-0.072	0.004	3.47E-73	0.075	327.725
	rs62160910	C	G	-0.024	0.004	4.53E-09	0.058	34.430
	rs62180255	A	T	0.034	0.002	1.81E-72	0.441	324.378
	rs78058190	A	G	0.047	0.005	1.36E-22	0.052	95.788
	rs7596846	T	G	-0.021	0.004	2.27E-09	0.921	35.790
	rs6755895	C	T	-0.023	0.002	1.65E-24	0.231	104.528
	rs1822534	G	A	-0.034	0.002	7.22E-71	0.394	317.178
	rs12330493	G	T	0.019	0.002	2.36E-28	0.365	122.176
	rs55938093	T	G	0.016	0.002	3.16E-17	0.530	71.371
	rs140763487	T	C	-0.049	0.008	9.26E-10	0.014	37.536
	rs9864216	A	G	0.024	0.002	1.57E-25	0.776	109.252
	rs10212190	T	A	-0.012	0.002	1.32E-09	0.379	36.830
	rs2322830	C	T	-0.011	0.002	2.42E-08	0.580	31.187
	rs34423734	A	C	-0.011	0.002	3.80E-08	0.266	30.292
	rs34850939	A	G	0.048	0.002	1.63E-117	0.346	531.139
	rs6445826	C	T	0.020	0.002	7.68E-25	0.498	106.044
	rs35592432	C	G	0.109	0.006	1.68E-68	0.027	306.173
	rs77101566	A	G	0.032	0.005	4.94E-10	0.037	38.766
	rs2100139	C	T	0.015	0.002	2.32E-14	0.658	58.349
	rs9835571	G	T	0.022	0.002	3.49E-27	0.690	116.792
	rs77265382	T	C	-0.033	0.005	1.17E-12	0.045	50.620
	rs4685105	G	A	-0.011	0.002	3.21E-08	0.415	30.614
	rs11706384	T	G	0.027	0.002	7.44E-34	0.236	147.278
	rs77057307	A	G	0.028	0.005	2.08E-08	0.040	31.462
	rs332507	C	T	-0.015	0.002	3.12E-11	0.222	44.155
	rs56850780	T	G	-0.021	0.004	3.55E-09	0.078	34.916
	rs721377	T	C	0.020	0.003	5.23E-10	0.102	38.653
	rs1991431	A	G	0.024	0.002	8.91E-36	0.441	156.098

rs2270968	G	T	-0.013	0.002	2.46E-09	0.738	35.638
rs6808949	C	T	0.023	0.003	9.16E-14	0.111	55.611
rs74280151	T	C	0.029	0.002	2.22E-38	0.175	167.950
rs62262391	T	C	-0.026	0.002	3.52E-30	0.225	130.440
rs11712335	C	T	0.015	0.002	1.26E-12	0.269	50.454
rs12636926	C	T	-0.014	0.002	1.92E-10	0.730	40.616
rs9864087	C	T	0.014	0.002	2.40E-13	0.576	53.752
rs76830965	A	C	-0.021	0.003	2.13E-12	0.117	49.432
rs2291900	A	G	0.017	0.002	1.49E-15	0.291	63.735
rs1491664	A	G	-0.015	0.002	1.54E-10	0.201	41.056
rs11505019	G	T	-0.013	0.002	3.18E-12	0.411	48.636
rs2089979	G	A	-0.035	0.002	4.10E-72	0.414	322.932
rs12640189	T	C	0.016	0.003	3.40E-10	0.176	39.485
rs17005891	A	G	-0.024	0.002	4.43E-43	0.184	189.521
rs34499378	A	G	0.011	0.002	3.27E-08	0.330	30.570
rs7672879	A	T	0.013	0.002	1.08E-11	0.353	46.262
rs62334097	G	A	-0.019	0.003	3.38E-10	0.113	39.505
rs3822030	T	G	0.011	0.002	3.48E-08	0.571	30.460
rs6554158	A	G	0.014	0.002	2.34E-10	0.231	40.203
rs58408429	C	T	-0.024	0.002	2.42E-22	0.186	94.621
rs1431545	T	C	0.021	0.002	5.05E-25	0.653	106.872
rs7661349	C	T	0.025	0.002	7.87E-38	0.637	165.530
rs7696969	G	T	-0.012	0.002	7.36E-10	0.620	37.972
rs74408817	A	C	-0.023	0.003	2.10E-11	0.081	44.931
rs73142294	T	C	0.015	0.002	7.51E-12	0.248	46.958
rs2282680	T	C	-0.014	0.002	4.98E-11	0.289	43.247
rs17703261	T	A	0.010	0.002	2.67E-08	0.189	30.982
rs5026470	G	A	0.037	0.002	1.45E-84	0.517	380.042
rs2298979	A	G	-0.020	0.002	3.20E-24	0.406	103.234
rs309375	A	C	0.019	0.002	7.84E-23	0.575	96.908
rs1344603	C	T	0.029	0.002	1.19E-47	0.676	210.479
rs4833031	C	T	0.018	0.003	5.70E-11	0.139	42.977
rs13133642	C	T	0.066	0.002	1.00E-200	0.681	1073.482
rs10027415	T	C	-0.016	0.001	6.33E-33	0.529	142.965
rs13105987	A	G	0.046	0.002	3.64E-103	0.274	465.278
rs4696314	C	T	0.014	0.002	9.33E-14	0.515	55.575
rs419470	G	A	0.015	0.002	9.50E-13	0.717	51.010
rs369540605	G	A	0.019	0.002	3.34E-16	0.208	66.682
rs28710104	G	A	-0.014	0.002	1.75E-12	0.683	49.803
rs251391	T	C	0.020	0.002	1.21E-19	0.747	82.343
rs2161118	C	T	0.011	0.002	4.74E-08	0.328	29.883

rs464609	A	G	0.012	0.002	5.30E-10	0.544	38.618
rs78270096	A	G	-0.034	0.004	7.79E-19	0.070	78.646
rs3992621	C	T	-0.010	0.002	4.31E-08	0.536	30.059
rs10076701	C	T	-0.023	0.002	5.52E-33	0.557	143.283
rs79237520	T	C	0.085	0.006	4.14E-40	0.023	175.932
rs444613	G	A	0.015	0.002	1.00E-15	0.425	64.537
rs17055818	C	T	-0.030	0.002	2.15E-42	0.252	186.447
rs55977949	A	G	-0.030	0.003	1.15E-21	0.098	91.589
rs4976642	A	T	0.024	0.003	3.44E-22	0.831	93.972
rs2910580	A	T	-0.014	0.002	3.57E-12	0.659	48.419
rs251398	C	T	-0.017	0.002	2.39E-19	0.447	81.037
rs73239280	A	G	-0.032	0.002	4.72E-41	0.203	180.231
rs58265751	C	T	-0.012	0.002	1.61E-10	0.521	40.942
rs360017	G	A	-0.021	0.002	1.69E-20	0.775	86.221
rs11567701	T	G	0.054	0.002	2.40E-141	0.262	641.005
rs678393	G	T	-0.015	0.002	2.32E-12	0.262	49.281
rs6874316	A	T	0.010	0.002	3.80E-08	0.465	30.305
rs304154	C	T	0.012	0.002	5.00E-09	0.308	34.232
rs2548256	T	G	0.014	0.002	6.49E-13	0.620	51.779
rs3797727	A	T	0.020	0.003	4.94E-11	0.112	43.268
rs11742873	T	C	-0.017	0.003	1.75E-10	0.837	40.781
rs4406174	T	C	0.018	0.003	2.27E-08	0.101	31.296
rs246524	C	G	0.012	0.002	8.79E-10	0.318	37.632
rs247557	G	T	-0.011	0.002	6.05E-09	0.524	33.855
rs11134475	G	A	-0.014	0.002	4.42E-13	0.636	52.500
rs1050979	G	A	0.025	0.002	3.97E-38	0.525	166.883
rs2853951	T	C	0.076	0.002	1.00E-200	0.597	1551.717
rs71536554	C	T	-0.049	0.004	1.38E-40	0.154	178.067
rs6929523	G	A	0.017	0.002	2.58E-16	0.707	67.194
rs74526869	A	G	0.035	0.003	3.60E-34	0.125	148.718
rs12190136	A	G	0.012	0.002	3.99E-10	0.526	39.162
rs2327531	G	A	0.017	0.002	9.93E-13	0.207	50.925
rs4869745	T	C	0.013	0.002	1.61E-09	0.289	36.445
rs17710008	A	G	0.021	0.002	4.45E-17	0.182	70.667
rs75628831	A	G	0.030	0.003	1.38E-18	0.086	77.543
rs2442718	T	C	-0.029	0.002	5.38E-48	0.370	212.154
rs12526696	A	G	0.037	0.003	6.06E-44	0.161	193.455
rs6926219	A	G	0.027	0.002	2.23E-45	0.547	200.007
rs9375486	T	C	-0.014	0.002	1.40E-12	0.380	50.244
rs72835315	C	T	0.035	0.004	7.09E-17	0.060	69.741
rs9328393	A	C	0.023	0.002	5.74E-31	0.668	134.059

rs62389639	G	A	0.018	0.003	2.03E-11	0.166	45.008
rs1611236	A	G	-0.042	0.002	9.76E-97	0.323	436.083
rs7757316	G	A	-0.049	0.004	8.70E-45	0.079	197.323
rs2294421	C	T	-0.011	0.002	2.07E-08	0.432	31.474
rs4368798	G	A	0.012	0.002	1.07E-10	0.427	41.748
rs3134754	G	A	-0.050	0.002	8.67E-155	0.446	702.791
rs72928038	A	G	-0.033	0.002	4.24E-41	0.177	180.388
rs13199519	A	G	-0.014	0.002	5.37E-11	0.298	43.090
rs3012415	T	C	0.014	0.003	2.65E-08	0.823	30.981
rs2273215	A	G	-0.015	0.002	5.04E-14	0.458	56.826
rs607465	G	A	0.034	0.004	4.83E-19	0.066	79.628
rs6911448	C	G	-0.013	0.002	1.11E-11	0.511	46.192
rs707833	A	G	0.015	0.002	2.51E-14	0.636	58.198
rs1291414	T	C	0.012	0.002	3.18E-09	0.298	35.138
rs1132352	C	A	-0.024	0.002	1.15E-36	0.539	160.092
rs4714431	C	A	-0.011	0.002	4.15E-08	0.371	30.130
rs2294861	T	C	0.019	0.002	3.84E-20	0.294	84.579
rs7776054	G	A	-0.040	0.002	9.81E-78	0.261	348.559
rs41267765	T	C	-0.046	0.006	1.24E-12	0.022	50.497
rs80006387	G	A	-0.032	0.005	6.59E-09	0.032	33.705
rs9920	C	T	0.029	0.003	2.15E-21	0.105	90.348
rs62621812	A	G	0.056	0.007	2.73E-16	0.021	67.091
rs2727487	C	A	-0.013	0.002	1.08E-10	0.629	41.743
rs62485905	T	C	-0.018	0.002	1.97E-20	0.551	85.967
rs73165514	A	G	0.021	0.003	5.47E-10	0.086	38.570
rs2347784	G	C	0.028	0.002	6.95E-39	0.268	170.366
rs17138740	G	A	-0.013	0.002	6.13E-09	0.215	33.843
rs9648346	G	C	-0.024	0.002	2.63E-25	0.203	108.206
rs10230506	A	T	0.021	0.003	2.86E-16	0.161	67.013
rs7790229	A	G	0.012	0.002	1.71E-10	0.493	40.822
rs7780328	G	A	0.037	0.004	4.75E-23	0.067	97.864
rs35114152	G	A	0.025	0.002	1.73E-27	0.226	118.167
rs10480003	G	A	-0.013	0.002	4.97E-12	0.447	47.760
rs35345753	G	C	-0.027	0.002	6.95E-30	0.207	129.057
rs35121828	A	G	0.013	0.002	1.22E-09	0.259	36.978
rs3735485	G	A	0.044	0.003	3.56E-63	0.845	281.713
rs10245472	A	G	-0.016	0.002	1.65E-10	0.177	40.918
rs10237524	A	G	-0.019	0.002	1.35E-21	0.330	91.255
rs4718976	C	T	-0.018	0.002	1.10E-17	0.695	73.449
rs3812316	G	C	-0.017	0.003	4.63E-09	0.127	34.396
rs75049211	T	C	-0.031	0.005	3.42E-10	0.040	39.476

rs6955702	G	A	-0.022	0.002	3.36E-31	0.522	135.170
rs2908425	A	G	0.014	0.002	3.78E-12	0.332	48.297
rs2710804	C	T	0.014	0.002	1.63E-12	0.376	49.943
rs6966893	A	G	0.019	0.002	3.91E-14	0.179	57.320
rs149984502	T	C	0.048	0.007	1.18E-11	0.019	46.070
rs1637366	G	C	0.014	0.002	1.40E-10	0.289	41.239
rs78976959	A	G	0.033	0.005	8.41E-12	0.041	46.733
rs11761199	G	A	-0.011	0.002	1.84E-08	0.454	31.709
rs7802739	T	C	-0.022	0.002	1.85E-21	0.793	90.588
rs6967605	G	C	-0.013	0.002	1.68E-11	0.427	45.391
rs7839516	C	T	0.015	0.002	3.08E-15	0.369	62.288
rs117934175	A	G	-0.045	0.005	1.12E-19	0.041	82.511
rs1384804	A	C	-0.050	0.002	1.08E-116	0.748	527.422
rs2453628	A	G	0.011	0.002	1.39E-08	0.526	32.240
rs1264593	T	G	0.015	0.002	2.15E-14	0.495	58.491
rs17180155	C	T	-0.011	0.002	9.21E-09	0.394	33.050
rs10107362	G	C	0.013	0.002	8.36E-11	0.361	42.247
rs13276352	C	T	0.014	0.002	2.15E-09	0.209	35.882
rs2980888	C	T	-0.015	0.002	1.21E-13	0.697	55.077
rs10503147	T	C	0.021	0.003	7.48E-10	0.089	37.946
rs6586777	A	C	-0.013	0.002	1.79E-11	0.438	45.241
rs1533065	T	C	0.017	0.002	4.95E-17	0.345	70.468
rs113142693	C	T	-0.017	0.002	1.46E-14	0.247	59.241
rs6474359	C	T	-0.051	0.005	1.13E-24	0.038	105.306
rs4737010	A	G	0.027	0.002	2.47E-32	0.229	140.324
rs12542907	G	C	-0.011	0.002	7.87E-09	0.399	33.358
rs2055101	T	C	-0.013	0.002	1.84E-12	0.527	49.739
rs10956401	A	G	-0.025	0.002	5.05E-37	0.345	161.795
rs28588745	T	A	0.022	0.002	1.25E-21	0.206	91.419
rs56094005	G	A	-0.040	0.005	1.61E-17	0.043	72.677
rs2980821	A	C	-0.013	0.002	4.97E-12	0.425	47.787
rs13262073	G	C	-0.012	0.002	7.10E-09	0.688	33.557
rs10109370	A	C	-0.019	0.002	2.26E-17	0.238	72.040
rs75653581	T	C	0.061	0.009	5.45E-12	0.013	47.597
rs881492	G	C	-0.019	0.002	6.56E-23	0.467	97.236
rs7007986	A	G	-0.022	0.002	8.50E-28	0.601	119.571
rs6475476	G	A	0.011	0.002	3.12E-08	0.373	30.673
rs2065500	G	A	-0.048	0.003	3.29E-77	0.160	346.172
rs10814138	A	C	-0.014	0.002	2.55E-11	0.314	44.551
rs61750929	T	C	-0.070	0.004	1.36E-65	0.056	292.819
rs1002607	C	T	-0.013	0.002	1.93E-11	0.593	45.092

rs3761846	T	C	0.021	0.002	6.26E-27	0.570	115.571
rs616154	T	C	-0.010	0.002	4.19E-08	0.515	30.096
rs4880192	G	A	0.029	0.002	1.16E-40	0.721	178.376
rs1971429	A	C	0.013	0.002	2.01E-09	0.729	36.034
rs501461	T	G	-0.018	0.002	6.76E-20	0.600	83.523
rs3731211	A	T	0.044	0.002	5.14E-95	0.720	428.156
rs2807303	A	G	0.022	0.002	2.79E-27	0.346	117.200
rs10761129	T	C	0.011	0.002	1.62E-08	0.669	31.957
rs3739756	C	T	-0.032	0.005	2.88E-10	0.036	39.812
rs10986338	A	G	-0.012	0.002	7.60E-09	0.650	33.424
rs10965124	G	T	-0.023	0.003	3.42E-12	0.095	48.505
rs2295797	C	T	0.020	0.002	4.99E-22	0.295	93.220
rs10973700	C	G	-0.019	0.002	1.11E-23	0.487	100.782
rs10985915	G	A	-0.023	0.003	6.90E-14	0.111	56.173
rs35010780	A	G	-0.021	0.003	1.30E-13	0.131	54.930
rs7855586	G	A	0.015	0.002	1.03E-12	0.290	50.863
rs2313532	A	G	-0.014	0.002	1.87E-13	0.401	54.204
rs3118469	T	A	0.029	0.002	1.50E-45	0.300	200.895
rs748113	C	T	-0.034	0.002	1.53E-65	0.436	292.607
rs10748526	C	T	0.015	0.002	7.88E-11	0.794	42.342
rs34664889	A	C	0.020	0.003	2.47E-15	0.160	62.732
rs58667319	T	C	-0.023	0.003	9.91E-16	0.123	64.555
rs41317270	T	C	-0.018	0.002	9.82E-14	0.177	55.505
rs8181326	A	G	0.012	0.002	6.99E-10	0.659	38.069
rs2992263	A	G	-0.020	0.002	1.58E-25	0.610	109.230
rs4948822	G	T	0.013	0.002	1.32E-08	0.232	32.365
rs780669	T	C	-0.022	0.003	5.95E-13	0.887	51.929
rs10828725	T	G	-0.032	0.002	1.29E-59	0.366	265.417
rs4948492	T	C	-0.022	0.002	5.69E-28	0.662	120.340
rs71476119	G	A	0.022	0.003	1.03E-16	0.157	68.988
rs80177247	G	A	-0.014	0.003	1.76E-08	0.168	31.793
rs1399180	C	T	0.026	0.003	1.82E-24	0.829	104.380
rs7893800	A	G	0.011	0.002	3.43E-08	0.390	30.510
rs2462017	A	G	0.014	0.002	2.87E-12	0.652	48.867
rs7093481	G	A	-0.017	0.002	6.84E-16	0.307	65.284
rs61850684	A	G	-0.032	0.002	2.59E-47	0.234	209.030
rs10762264	A	G	-0.020	0.002	1.06E-23	0.681	100.818
rs603424	A	G	0.020	0.002	2.78E-16	0.175	67.039
rs1923403	G	A	0.020	0.004	1.68E-08	0.922	31.881
rs7924213	A	G	-0.013	0.002	1.18E-10	0.307	41.579
rs10823631	G	A	-0.017	0.002	3.11E-17	0.325	71.412

rs7071131	G	A	-0.016	0.002	3.85E-16	0.495	66.387
rs10794175	T	G	-0.021	0.002	2.50E-26	0.416	112.799
rs2403246	G	C	0.012	0.002	4.81E-10	0.414	38.796
rs74679312	G	A	0.031	0.004	2.28E-13	0.057	53.823
rs79805393	C	T	0.017	0.003	1.04E-11	0.173	46.330
rs11246147	T	C	0.011	0.002	9.52E-09	0.549	32.975
rs744222	C	T	-0.012	0.002	1.26E-08	0.666	32.436
rs11231804	T	C	0.074	0.012	2.85E-09	0.007	35.337
rs11224302	T	C	-0.038	0.003	1.73E-33	0.099	145.607
rs1893033	T	C	-0.028	0.004	5.70E-15	0.075	61.093
rs34662054	C	G	0.014	0.002	4.80E-12	0.299	47.828
rs73000965	A	T	0.013	0.002	2.63E-10	0.315	39.991
rs2294081	C	T	0.016	0.002	8.30E-15	0.481	60.373
rs11033388	T	A	0.020	0.002	2.07E-24	0.419	104.082
rs10838634	G	A	-0.026	0.003	3.00E-16	0.902	66.894
rs2298552	T	C	0.017	0.002	2.71E-18	0.526	76.218
rs949349	C	T	0.014	0.002	3.26E-11	0.271	44.085
rs74911261	A	G	0.045	0.006	8.68E-14	0.026	55.730
rs11218725	G	A	0.033	0.002	1.03E-63	0.377	284.192
rs7127911	T	G	0.024	0.002	3.44E-29	0.266	125.958
rs61867141	A	G	-0.017	0.003	1.37E-11	0.195	45.796
rs1017875	T	C	-0.036	0.002	5.01E-53	0.202	235.184
rs77876222	T	C	0.045	0.007	2.53E-10	0.020	40.077
rs10466905	A	G	0.062	0.002	7.10E-140	0.190	634.341
rs11168249	C	T	-0.019	0.002	6.04E-24	0.461	101.947
rs73105786	A	G	-0.015	0.002	7.76E-14	0.319	55.933
rs34229224	A	G	-0.048	0.008	2.78E-09	0.014	35.388
rs73207610	T	G	-0.034	0.006	6.65E-09	0.027	33.685
rs67516712	A	G	0.015	0.002	9.11E-15	0.423	60.145
rs10783340	G	A	-0.012	0.002	1.34E-09	0.639	36.805
rs1371076	C	T	0.015	0.002	4.22E-12	0.740	48.081
rs11052877	G	A	0.069	0.002	1.00E-200	0.372	1236.965
rs36108061	G	C	-0.025	0.004	5.54E-10	0.057	38.533
rs147700878	A	G	0.026	0.005	2.39E-08	0.044	31.191
rs78983078	A	G	-0.062	0.010	2.07E-10	0.010	40.460
rs28533432	T	C	-0.025	0.002	7.17E-33	0.700	142.835
rs2369391	C	A	-0.017	0.002	7.43E-19	0.494	78.758
rs2068485	G	T	0.011	0.002	2.55E-08	0.447	31.078
rs4578474	G	A	-0.012	0.002	7.47E-09	0.265	33.471
rs4149569	G	C	-0.021	0.002	2.24E-26	0.414	113.116
rs12811832	A	G	-0.016	0.002	5.99E-16	0.354	65.560

rs4760278	A	C	-0.014	0.002	6.48E-10	0.223	38.228
rs1118866	T	C	0.015	0.002	1.10E-12	0.704	50.729
rs7308380	C	A	0.016	0.002	2.47E-13	0.751	53.659
rs74344617	A	G	-0.013	0.002	3.79E-08	0.192	30.312
rs6488548	A	C	0.017	0.002	9.43E-19	0.599	78.292
rs6487543	A	G	0.020	0.002	6.20E-18	0.771	74.568
rs7312770	T	C	0.012	0.002	2.80E-10	0.506	39.883
rs937283	G	A	0.011	0.002	3.72E-08	0.405	30.324
rs17041439	C	A	0.041	0.004	2.11E-23	0.057	99.503
rs10774624	A	G	-0.088	0.002	1.00E-200	0.514	2097.030
rs10846577	C	T	-0.014	0.002	2.37E-13	0.442	53.760
rs12586002	G	T	-0.014	0.002	4.87E-12	0.353	47.815
rs1320472	C	T	-0.014	0.002	1.57E-12	0.461	50.029
rs9525619	T	C	-0.020	0.002	1.08E-26	0.534	114.566
rs67483792	T	C	-0.035	0.005	8.06E-14	0.043	55.879
rs9592965	C	A	-0.014	0.002	8.83E-13	0.327	51.158
rs9534338	C	T	0.015	0.002	1.38E-15	0.460	63.873
rs7983682	T	C	0.014	0.002	1.34E-11	0.323	45.816
rs3812849	C	A	0.043	0.002	1.32E-91	0.266	412.229
rs2847468	T	C	-0.012	0.002	2.94E-08	0.264	30.787
rs76428106	C	T	0.063	0.009	5.49E-13	0.013	52.104
rs9532679	C	A	-0.032	0.003	6.33E-33	0.149	143.007
rs1523178	C	A	-0.026	0.002	3.86E-29	0.221	125.670
rs9590390	A	G	0.030	0.002	2.51E-46	0.292	204.379
rs912416	C	A	-0.012	0.002	3.58E-10	0.445	39.380
rs11160706	A	G	0.030	0.002	2.12E-40	0.222	177.223
rs2057340	G	A	0.014	0.002	4.66E-12	0.653	47.883
rs10138752	T	C	-0.030	0.004	4.89E-17	0.077	70.471
rs2180369	C	T	0.020	0.003	1.47E-10	0.111	41.131
rs696	T	C	0.025	0.002	4.94E-38	0.365	166.378
rs112992671	A	G	-0.027	0.005	8.35E-09	0.046	33.244
rs175714	C	T	0.026	0.002	9.20E-42	0.577	183.454
rs45528934	T	C	-0.021	0.003	1.85E-15	0.162	63.332
rs1966865	A	G	0.027	0.002	1.36E-40	0.317	178.101
rs10145277	A	T	-0.015	0.002	3.19E-14	0.625	57.724
rs8004780	C	A	0.018	0.002	1.60E-20	0.366	86.343
rs2412544	T	C	-0.016	0.002	1.13E-16	0.394	68.824
rs12593807	C	T	-0.021	0.003	7.57E-14	0.136	56.016
rs149453951	T	C	0.049	0.007	4.04E-13	0.024	52.709
rs187856913	A	G	0.048	0.005	6.24E-22	0.039	92.787
rs3848132	A	T	-0.012	0.002	1.66E-08	0.292	31.892

rs7161799	T	C	0.034	0.004	2.79E-21	0.076	89.786
rs631864	C	T	0.014	0.002	1.48E-12	0.472	50.136
rs4129560	A	C	-0.011	0.002	1.21E-08	0.370	32.515
rs28539372	A	T	0.038	0.002	3.38E-78	0.319	350.657
rs34025077	G	A	-0.033	0.003	1.25E-25	0.101	109.644
rs17507693	T	A	0.016	0.002	9.86E-13	0.223	50.932
rs139974673	C	T	0.054	0.006	1.89E-19	0.026	81.454
rs2439408	C	G	-0.012	0.002	9.72E-11	0.561	41.964
rs11073903	A	G	-0.013	0.002	4.41E-11	0.567	43.498
rs9937837	G	T	-0.015	0.002	1.69E-12	0.270	49.902
rs1317575	C	G	0.014	0.002	2.80E-11	0.696	44.375
rs247826	T	C	0.039	0.002	1.19E-63	0.221	283.879
rs145719494	G	A	-0.031	0.004	3.71E-14	0.059	57.394
rs8046391	C	G	-0.024	0.002	9.68E-31	0.274	132.998
rs34140544	C	G	0.017	0.002	1.40E-17	0.648	72.916
rs7192652	G	A	0.022	0.002	4.32E-31	0.481	134.620
rs143875179	A	G	-0.030	0.005	1.08E-10	0.044	41.729
rs78487935	G	A	-0.024	0.004	3.20E-11	0.078	44.125
rs3810818	C	A	-0.018	0.002	9.13E-15	0.773	60.143
rs12598978	G	T	0.055	0.002	1.58E-181	0.520	825.967
rs1677490	C	G	0.016	0.002	1.25E-11	0.791	45.979
rs17229044	T	C	-0.019	0.002	1.78E-16	0.210	67.938
rs117556162	A	G	0.044	0.004	3.31E-27	0.058	116.849
rs9939124	T	C	-0.017	0.002	3.60E-16	0.307	66.568
rs8052370	T	C	-0.021	0.002	5.82E-25	0.609	106.659
rs11652760	G	T	-0.021	0.003	3.01E-11	0.100	44.232
rs2665960	G	A	0.012	0.002	4.03E-09	0.669	34.680
rs4789294	G	A	0.027	0.002	1.05E-30	0.219	132.835
rs116876036	T	C	0.021	0.003	9.57E-10	0.090	37.468
rs62091998	G	A	-0.022	0.002	1.33E-27	0.307	118.713
rs2453582	T	C	0.018	0.002	4.88E-19	0.391	79.567
rs10445308	T	C	-0.030	0.002	1.91E-56	0.479	250.883
rs1292069	C	T	-0.023	0.002	3.09E-32	0.450	139.843
rs11657448	T	C	0.013	0.002	8.47E-10	0.322	37.721
rs2297508	G	C	-0.022	0.002	2.20E-27	0.648	117.661
rs3136685	T	C	-0.020	0.003	6.51E-14	0.164	56.277
rs56308324	T	A	0.018	0.003	2.36E-10	0.131	40.213
rs8075090	C	T	0.021	0.002	2.01E-28	0.524	122.407
rs3744399	C	T	-0.022	0.003	4.61E-16	0.143	66.042
rs853198	C	A	-0.020	0.002	4.00E-25	0.640	107.299
rs145947882	C	A	-0.035	0.006	4.97E-09	0.026	34.249

rs9906320	A	G	0.025	0.002	8.67E-27	0.775	115.001
rs113513990	C	T	-0.038	0.003	6.37E-32	0.097	138.465
rs6502555	C	T	0.029	0.002	9.26E-38	0.269	165.131
rs35186877	A	G	0.032	0.002	1.94E-42	0.214	186.675
rs3760456	T	C	-0.015	0.002	1.40E-14	0.439	59.332
rs9898958	G	T	0.016	0.003	3.56E-09	0.153	34.909
rs1491765	A	G	-0.017	0.002	1.67E-16	0.293	68.045
rs8096327	G	A	-0.013	0.002	1.59E-10	0.391	40.982
rs3851820	T	A	-0.018	0.002	7.37E-16	0.745	65.115
rs4940572	A	G	0.023	0.003	1.17E-16	0.136	68.751
rs1456059	G	A	0.013	0.002	9.06E-09	0.766	33.078
rs4987855	T	C	-0.024	0.003	1.16E-13	0.096	55.154
rs9676181	A	T	0.012	0.002	9.73E-10	0.574	37.440
rs111626441	A	G	0.028	0.003	8.97E-17	0.097	69.293
rs7254517	A	G	0.029	0.002	1.05E-46	0.385	206.167
rs7249236	C	G	0.026	0.002	8.62E-26	0.802	110.357
rs4530278	T	G	0.017	0.002	5.24E-18	0.598	74.927
rs4805881	C	A	-0.015	0.002	1.41E-13	0.665	54.804
rs7252565	A	G	0.028	0.003	1.55E-27	0.788	118.368
rs1000329	T	C	-0.082	0.002	1.00E-200	0.676	1523.137
rs56179616	T	G	0.031	0.002	6.22E-47	0.259	207.202
rs11084096	A	G	-0.018	0.002	6.42E-19	0.296	79.035
rs2070745	G	C	-0.016	0.002	3.94E-16	0.367	66.353
rs141801008	T	C	-0.061	0.010	3.01E-09	0.010	35.231
rs11670621	T	C	0.011	0.002	3.11E-08	0.486	30.677
rs78487206	T	G	-0.019	0.003	9.12E-11	0.164	42.079
rs36084354	A	G	-0.061	0.003	6.60E-71	0.092	317.223
rs1043996	A	G	0.013	0.002	4.21E-10	0.717	39.081
rs75018496	G	C	0.025	0.004	1.60E-11	0.076	45.469
rs61387190	T	C	0.032	0.003	5.81E-34	0.157	147.743
rs12463256	A	G	0.015	0.002	4.32E-11	0.275	43.530
rs2569693	T	C	-0.042	0.002	3.36E-96	0.384	433.207
rs56252104	T	C	0.019	0.002	3.12E-18	0.261	75.887
rs766228	T	G	-0.011	0.002	3.37E-08	0.502	30.519
rs17093026	T	C	-0.022	0.003	3.13E-12	0.101	48.688
rs968478	G	A	0.015	0.002	3.11E-14	0.397	57.746
rs259981	A	T	-0.027	0.002	3.91E-42	0.360	185.148
rs2738783	G	T	-0.016	0.002	1.24E-10	0.790	41.472
rs1883801	G	A	-0.017	0.002	3.39E-12	0.195	48.508
rs6021231	C	T	-0.011	0.002	2.21E-09	0.471	35.818
rs4411786	C	T	-0.045	0.002	6.39E-98	0.265	441.311

	rs2145270	T	C	-0.011	0.002	2.70E-08	0.619	30.955
	rs2230604	T	C	0.030	0.003	3.65E-18	0.084	75.590
	rs611847	G	A	0.015	0.002	3.53E-13	0.636	52.978
	rs13040989	C	T	0.019	0.002	9.96E-20	0.292	82.728
	rs1997797	G	C	0.017	0.002	4.44E-19	0.446	79.823
	rs34323943	C	T	0.022	0.003	2.08E-13	0.111	53.998
	rs6072080	T	C	0.020	0.002	1.77E-24	0.585	104.393
	rs6103270	G	A	-0.014	0.002	1.91E-09	0.226	36.116
	rs4812804	T	C	0.013	0.002	2.10E-08	0.198	31.457
	rs1893592	C	A	0.015	0.002	2.13E-12	0.296	49.458
	rs1297265	G	A	0.012	0.002	7.44E-10	0.443	37.954
	rs150797	G	C	-0.014	0.002	2.19E-12	0.447	49.391
	rs721131	C	T	-0.019	0.002	1.36E-20	0.320	86.701
	rs12627489	A	G	0.017	0.003	3.88E-08	0.107	30.253
	rs9605047	T	G	0.020	0.002	2.99E-21	0.328	89.678
	rs5998509	T	C	-0.041	0.002	2.19E-61	0.188	273.499
	rs79503	G	A	-0.028	0.002	1.02E-46	0.447	206.222
	rs62241216	G	A	-0.012	0.002	3.88E-10	0.492	39.230
	rs71318973	T	G	-0.015	0.002	7.29E-12	0.241	47.038
	rs6001858	G	A	-0.015	0.002	2.37E-12	0.736	49.245
	rs16986308	A	G	-0.032	0.003	9.95E-28	0.116	119.286
	rs714027	G	A	-0.035	0.002	5.96E-75	0.552	335.744
	rs1807669	T	C	-0.027	0.002	9.00E-31	0.794	133.183
	rs760908	T	G	-0.015	0.003	9.70E-09	0.153	32.942
Monocyte count	rs11247908	A	G	-0.029	0.002	1.92E-32	0.173	140.871
	rs188393352	G	C	0.087	0.010	2.36E-17	0.010	71.927
	rs41268099	A	G	0.029	0.003	2.47E-22	0.111	94.635
	rs55684236	A	G	0.012	0.002	3.89E-09	0.348	34.747
	rs72675573	T	C	0.016	0.002	1.76E-16	0.366	67.949
	rs1535701	G	T	0.021	0.002	4.06E-27	0.621	116.426
	rs56795609	T	A	-0.015	0.002	1.87E-09	0.172	36.155
	rs4970966	T	G	-0.073	0.002	1.00E-200	0.222	1040.374
	rs3014807	T	G	0.026	0.003	1.31E-17	0.119	73.091
	rs3762281	G	A	0.013	0.002	2.97E-12	0.505	48.805
	rs2022003	T	A	0.017	0.002	5.41E-16	0.279	65.748
	rs12747432	T	G	-0.024	0.004	1.31E-10	0.067	41.361
	rs1772143	A	T	0.020	0.002	8.11E-26	0.413	110.549
	rs701905	C	G	0.022	0.002	1.63E-28	0.352	122.886
	rs4335411	A	G	-0.017	0.002	5.51E-13	0.762	52.118
	rs74765249	T	C	0.013	0.002	1.12E-09	0.281	37.173
	rs1933295	G	A	-0.024	0.002	1.74E-25	0.777	109.016

rs6664626	T	G	0.019	0.003	4.38E-11	0.143	43.494
rs41313381	A	C	0.043	0.005	8.85E-16	0.030	64.777
rs36209093	T	C	-0.014	0.002	2.16E-10	0.663	40.376
rs458699	A	T	0.014	0.002	4.52E-10	0.757	38.930
rs113292043	T	C	-0.025	0.004	5.45E-11	0.066	43.080
rs12144117	T	C	-0.014	0.002	7.63E-10	0.215	37.917
rs7524046	A	G	0.015	0.002	1.11E-11	0.251	46.197
rs628615	C	T	-0.017	0.003	9.16E-09	0.113	33.065
rs17387886	C	G	-0.028	0.003	1.45E-28	0.174	123.091
rs34417629	A	G	0.013	0.002	2.65E-09	0.234	35.479
rs12756133	A	G	0.013	0.002	2.98E-08	0.204	30.763
rs12118443	A	G	-0.018	0.002	4.00E-22	0.489	93.641
rs4658231	G	A	-0.016	0.002	2.23E-13	0.249	53.852
rs115340020	A	G	-0.094	0.006	2.22E-59	0.027	264.277
rs333947	A	G	0.037	0.003	2.21E-44	0.150	195.553
rs3027012	T	C	0.028	0.002	4.37E-31	0.183	134.604
rs11590380	C	T	0.025	0.002	1.69E-32	0.679	141.019
rs7522307	C	G	-0.023	0.003	4.36E-16	0.124	66.147
rs1335929	C	T	0.017	0.002	1.77E-18	0.571	77.076
rs4269828	G	A	0.012	0.002	1.89E-10	0.560	40.646
rs6429432	C	A	-0.080	0.003	6.30E-156	0.892	708.236
rs7516138	G	A	-0.023	0.002	1.31E-31	0.391	137.023
rs6687430	A	G	0.012	0.002	2.41E-10	0.543	40.183
rs284324	A	G	0.021	0.002	5.94E-27	0.495	115.698
rs2786487	C	G	0.015	0.002	3.33E-15	0.559	62.172
rs1889033	G	A	-0.016	0.002	3.18E-17	0.485	71.299
rs2274664	T	C	-0.015	0.002	2.75E-15	0.580	62.532
rs12742428	G	A	-0.017	0.002	1.68E-16	0.651	68.058
rs2810883	T	C	-0.013	0.002	1.94E-11	0.545	45.114
rs67224956	C	T	0.027	0.002	2.01E-27	0.170	117.826
rs4987353	A	G	-0.022	0.002	1.93E-27	0.310	117.973
rs3795503	T	C	-0.019	0.002	1.68E-20	0.315	86.250
rs6696074	T	C	0.018	0.002	1.49E-21	0.563	91.063
rs61165644	G	A	-0.026	0.003	2.59E-22	0.141	94.496
rs17575497	T	C	0.367	0.009	1.00E-200	0.013	1635.088
rs9787298	C	A	-0.016	0.002	2.66E-12	0.211	49.012
rs2784250	G	T	-0.017	0.003	3.11E-10	0.140	39.650
rs4626924	T	C	-0.018	0.002	8.20E-22	0.551	92.270
rs6429438	G	A	0.021	0.003	1.93E-12	0.886	49.622
rs4669869	C	T	0.020	0.002	3.76E-27	0.445	116.637
rs6753534	T	C	0.013	0.002	8.58E-12	0.562	46.693

rs647137	A	G	0.021	0.002	6.26E-24	0.697	101.919
rs75475627	G	C	0.024	0.004	1.80E-11	0.077	45.253
rs7569084	T	C	0.028	0.002	7.40E-48	0.584	211.498
rs7593080	T	C	0.011	0.002	1.80E-08	0.574	31.751
rs3789062	T	C	-0.020	0.002	3.22E-20	0.245	84.937
rs7574456	T	C	-0.040	0.002	5.20E-80	0.740	358.874
rs59295685	T	C	-0.020	0.002	7.99E-16	0.173	64.949
rs1863219	G	C	0.018	0.002	5.95E-23	0.512	97.456
rs145447411	C	T	0.046	0.006	5.80E-13	0.023	51.987
rs1400003	T	C	-0.012	0.002	4.70E-08	0.213	29.885
rs17860428	A	G	-0.016	0.002	1.39E-10	0.180	41.253
rs34236350	T	C	-0.032	0.003	8.44E-38	0.183	165.327
rs7572278	A	T	0.022	0.002	3.44E-20	0.207	84.804
rs1031091	G	A	-0.011	0.002	3.93E-08	0.336	30.214
rs28498283	T	A	0.021	0.002	2.41E-22	0.258	94.660
rs78218855	T	C	0.022	0.004	2.73E-09	0.068	35.426
rs2136451	A	G	0.015	0.002	2.00E-13	0.298	54.062
rs4907230	A	G	0.016	0.002	7.24E-15	0.324	60.594
rs6745920	C	T	0.013	0.002	2.02E-10	0.619	40.501
rs6736362	T	C	0.022	0.002	6.45E-32	0.559	138.377
rs1127101	G	A	0.014	0.002	3.98E-14	0.604	57.243
rs12993696	G	A	-0.014	0.002	6.32E-10	0.242	38.292
rs62176784	G	A	0.023	0.002	1.36E-29	0.319	127.773
rs30102	C	G	-0.013	0.002	9.89E-11	0.317	41.896
rs3111414	G	C	0.017	0.002	5.62E-14	0.794	56.604
rs1367153	A	G	-0.011	0.002	3.39E-08	0.708	30.523
rs4952782	A	T	0.019	0.002	1.17E-18	0.760	77.871
rs72836307	T	C	-0.041	0.003	1.37E-56	0.156	251.576
rs13027410	C	A	0.012	0.002	5.02E-09	0.317	34.220
rs13032491	T	C	0.016	0.003	4.72E-09	0.139	34.351
rs6733162	C	G	0.012	0.002	1.40E-09	0.624	36.732
rs8207	G	A	0.014	0.002	1.29E-10	0.267	41.374
rs34743120	A	G	-0.047	0.007	6.15E-12	0.020	47.356
rs6434817	G	A	0.012	0.002	2.17E-08	0.718	31.373
rs6545873	C	T	-0.015	0.002	1.18E-15	0.610	64.192
rs150449635	C	T	0.140	0.007	4.24E-97	0.022	437.597
rs3856364	G	C	0.013	0.002	3.45E-10	0.680	39.473
rs13032786	G	C	0.020	0.002	2.49E-23	0.300	99.206
rs4462758	A	G	-0.033	0.002	1.43E-52	0.750	233.117
rs6740847	G	A	0.127	0.002	1.00E-200	0.562	4558.489
rs10197805	C	T	-0.042	0.003	4.70E-59	0.157	262.884

rs12694733	A	G	-0.011	0.002	4.07E-09	0.582	34.647
rs1822534	G	A	-0.031	0.002	4.69E-59	0.394	262.792
rs2228467	C	T	0.157	0.004	1.00E-200	0.062	1652.109
rs111556916	G	C	-0.036	0.006	1.30E-09	0.027	36.872
rs875890	A	T	-0.017	0.002	3.38E-15	0.262	62.136
rs2213290	T	C	0.036	0.002	1.11E-75	0.408	339.249
rs10935473	T	G	0.025	0.002	2.30E-40	0.442	177.130
rs663202	C	G	-0.015	0.002	1.39E-09	0.171	36.752
rs11713343	A	G	-0.026	0.002	5.24E-28	0.197	120.513
rs73058593	C	G	0.038	0.002	1.98E-62	0.218	278.287
rs13094390	G	A	-0.021	0.003	1.11E-12	0.116	50.712
rs9809116	G	A	0.017	0.002	8.21E-20	0.409	83.086
rs1976633	G	A	-0.016	0.003	1.25E-10	0.835	41.433
rs7620353	T	C	0.014	0.002	6.42E-09	0.823	33.761
rs7633965	C	A	0.025	0.004	8.35E-12	0.929	46.752
rs869785	C	T	-0.017	0.002	3.97E-18	0.670	75.420
rs11130612	T	C	-0.012	0.002	4.71E-10	0.334	38.853
rs13073885	C	T	0.013	0.002	3.16E-10	0.315	39.621
rs71331613	A	C	-0.050	0.005	3.98E-23	0.036	98.235
rs6800122	T	C	-0.022	0.002	4.75E-30	0.400	129.835
rs16831132	T	C	-0.014	0.002	2.07E-12	0.397	49.487
rs6772164	A	C	-0.012	0.002	1.21E-08	0.359	32.525
rs7626444	C	G	-0.029	0.002	5.72E-52	0.421	230.294
rs6799804	A	T	-0.027	0.004	1.52E-14	0.075	59.145
rs62261974	G	A	-0.017	0.002	7.01E-12	0.183	47.110
rs2734047	G	A	0.077	0.002	1.00E-200	0.635	1562.147
rs34281413	T	C	-0.021	0.003	7.01E-15	0.137	60.703
rs231988	C	T	0.026	0.003	4.80E-19	0.868	79.616
rs56925909	G	C	0.014	0.002	1.33E-09	0.219	36.814
rs9815073	A	C	-0.022	0.002	4.16E-26	0.346	111.870
rs2371108	T	G	0.011	0.002	3.01E-08	0.388	30.755
rs13077681	G	T	0.041	0.006	8.99E-11	0.023	42.094
rs58011825	C	A	0.018	0.003	1.69E-11	0.137	45.360
rs2018092	C	T	-0.012	0.002	1.97E-09	0.615	36.050
rs1587222	G	A	0.014	0.002	1.72E-11	0.702	45.341
rs10518329	G	A	-0.014	0.002	6.18E-12	0.331	47.358
rs72720206	A	G	-0.016	0.002	5.82E-16	0.327	65.603
rs871134	T	C	0.038	0.002	7.06E-89	0.571	399.936
rs73809166	C	T	0.046	0.006	3.78E-13	0.023	52.839
rs2711981	T	C	-0.022	0.002	1.59E-27	0.663	118.290
rs723585	G	A	-0.017	0.002	9.43E-19	0.483	78.251

rs4566648	G	T	-0.025	0.002	8.14E-38	0.342	165.346
rs141936164	G	A	-0.019	0.002	2.29E-21	0.351	90.211
rs28723530	A	C	-0.015	0.003	2.93E-08	0.135	30.807
rs7687559	C	G	0.014	0.002	1.58E-12	0.398	50.040
rs6856799	C	T	-0.011	0.002	3.81E-08	0.315	30.291
rs9637714	C	T	-0.016	0.002	5.42E-15	0.289	61.213
rs11723621	G	A	-0.013	0.002	1.48E-10	0.291	41.100
rs17005891	A	G	-0.051	0.002	1.15E-98	0.184	444.585
rs28612496	A	G	0.014	0.002	5.82E-09	0.173	33.937
rs199498839	G	C	-0.025	0.003	7.94E-14	0.085	55.890
rs56058420	G	A	0.012	0.002	2.96E-09	0.356	35.274
rs1425553	A	G	-0.011	0.002	5.34E-09	0.579	34.104
rs9992013	G	C	0.022	0.003	1.18E-15	0.137	64.189
rs144317085	T	A	0.062	0.005	3.10E-32	0.034	139.888
rs200910839	A	G	-0.018	0.002	8.82E-22	0.403	92.128
rs4240356	G	C	-0.024	0.002	1.94E-35	0.550	154.458
rs4865956	A	T	-0.019	0.002	1.97E-20	0.696	85.905
rs12522498	G	A	-0.011	0.002	2.05E-08	0.361	31.490
rs964752	G	C	-0.012	0.002	9.61E-11	0.540	41.972
rs28722705	T	A	0.034	0.003	1.00E-38	0.149	169.592
rs74735005	C	T	-0.017	0.002	3.14E-13	0.206	53.226
rs2548257	A	C	0.013	0.002	5.35E-11	0.653	43.114
rs10478058	G	A	0.015	0.002	3.01E-10	0.197	39.710
rs6883116	C	T	-0.018	0.002	8.21E-21	0.417	87.653
rs13167280	A	G	0.017	0.003	6.07E-09	0.128	33.866
rs11242109	T	G	-0.022	0.002	4.75E-33	0.478	143.526
rs80027325	C	T	-0.030	0.005	7.20E-09	0.035	33.535
rs1902796	G	A	-0.012	0.002	2.97E-08	0.736	30.782
rs360017	G	A	-0.024	0.002	1.62E-27	0.775	118.278
rs116619972	A	G	-0.040	0.006	6.73E-12	0.026	47.182
rs445611	G	A	0.016	0.003	8.58E-10	0.845	37.685
rs7720894	C	G	0.011	0.002	6.79E-09	0.503	33.650
rs6865582	G	C	-0.011	0.002	4.48E-09	0.461	34.471
rs190982	A	G	-0.012	0.002	1.77E-10	0.603	40.778
rs2338021	C	T	-0.032	0.003	3.64E-28	0.880	121.259
rs31243	G	A	-0.023	0.003	1.35E-12	0.096	50.339
rs6869021	T	C	-0.017	0.002	5.47E-17	0.313	70.245
rs12332674	G	T	-0.017	0.003	3.33E-10	0.152	39.529
rs329125	A	C	-0.017	0.002	1.38E-12	0.207	50.297
rs6579771	T	C	0.034	0.002	6.13E-57	0.269	253.042
rs17656204	T	C	-0.054	0.002	6.78E-156	0.322	708.080

rs707793	C	T	-0.030	0.002	2.13E-58	0.531	259.875
rs9295484	T	G	-0.029	0.005	6.10E-10	0.956	38.352
rs28367597	G	C	-0.020	0.002	4.39E-22	0.273	93.493
rs41552812	T	C	-0.072	0.005	4.10E-45	0.062	198.824
rs11965885	T	G	-0.011	0.002	2.09E-08	0.582	31.453
rs644492	G	A	-0.020	0.002	1.84E-17	0.196	72.437
rs12198236	C	T	-0.010	0.002	4.46E-08	0.562	29.989
rs1970364	T	C	0.019	0.002	1.32E-19	0.265	82.181
rs73784287	G	A	0.015	0.003	4.93E-09	0.149	34.261
rs2273215	A	G	-0.018	0.002	2.72E-20	0.458	85.301
rs2927	C	T	-0.017	0.002	2.85E-18	0.580	76.116
rs4712652	A	G	0.015	0.002	2.49E-15	0.580	62.742
rs12207691	G	A	-0.012	0.002	9.49E-09	0.304	33.004
rs761841	C	T	0.012	0.002	1.31E-10	0.577	41.383
rs7776054	G	A	-0.038	0.002	6.09E-71	0.261	317.284
rs11155787	T	C	0.014	0.002	3.92E-12	0.638	48.248
rs2817441	T	C	-0.022	0.002	4.67E-25	0.279	107.073
rs71550194	C	T	-0.029	0.002	7.53E-51	0.595	225.187
rs3128959	A	G	0.032	0.003	3.74E-28	0.121	121.213
rs115202835	A	G	0.027	0.004	4.52E-13	0.068	52.488
rs9494573	C	T	-0.016	0.002	2.18E-16	0.589	67.541
rs2797681	C	G	-0.015	0.002	1.14E-10	0.256	41.627
rs12530071	C	T	-0.015	0.002	5.78E-11	0.225	42.963
rs3761986	C	T	-0.012	0.002	4.88E-10	0.394	38.797
rs1283945	C	T	-0.035	0.006	1.38E-08	0.976	32.258
rs10948314	G	A	-0.034	0.003	1.04E-27	0.103	119.168
rs9480737	G	A	-0.022	0.002	2.74E-27	0.321	117.197
rs12055642	C	T	0.018	0.002	1.04E-14	0.199	59.911
rs9390460	C	T	0.012	0.002	3.01E-10	0.538	39.722
rs9379077	G	A	0.029	0.002	1.46E-34	0.201	150.456
rs1611236	A	G	-0.034	0.002	2.28E-63	0.323	282.614
rs2523562	T	C	-0.068	0.002	1.00E-200	0.246	995.704
rs56007794	T	A	0.039	0.002	4.77E-72	0.244	322.331
rs79894332	A	G	-0.048	0.003	6.30E-54	0.100	239.325
rs915125	T	C	0.034	0.002	4.34E-59	0.282	263.058
rs9375150	G	T	-0.018	0.002	1.84E-22	0.447	95.243
rs149110519	T	C	0.075	0.005	1.51E-48	0.036	214.579
rs3012415	T	C	0.020	0.003	1.37E-15	0.823	63.880
rs4721764	A	G	0.016	0.002	2.12E-11	0.210	44.924
rs798563	C	A	0.018	0.002	3.23E-17	0.297	71.280
rs112248289	C	A	0.022	0.003	4.83E-15	0.129	61.416

rs4385425	G	A	-0.060	0.002	1.28E-198	0.324	904.023
rs62491927	A	C	-0.013	0.002	1.42E-09	0.257	36.691
rs17156536	A	C	0.025	0.003	8.66E-22	0.159	92.113
rs7786376	G	A	0.012	0.002	4.73E-09	0.276	34.338
rs1091815	A	G	-0.014	0.002	3.14E-10	0.765	39.651
rs147341073	G	A	-0.075	0.006	2.25E-37	0.028	163.376
rs13225324	A	G	0.013	0.002	3.69E-10	0.305	39.343
rs7803075	G	A	-0.014	0.002	2.28E-11	0.735	44.768
rs13229868	A	G	0.016	0.003	1.73E-09	0.197	36.320
rs62454420	G	A	-0.035	0.004	2.39E-21	0.070	90.108
rs73142138	G	A	0.013	0.002	2.57E-11	0.400	44.558
rs7785014	C	T	-0.025	0.002	2.04E-36	0.671	158.923
rs13238198	T	C	-0.022	0.003	7.88E-13	0.108	51.389
rs149007767	T	C	-0.072	0.003	1.97E-163	0.162	742.421
rs2106135	T	C	-0.053	0.007	6.76E-14	0.018	56.225
rs62470670	G	A	-0.023	0.003	5.30E-13	0.092	52.184
rs2885735	G	A	0.014	0.003	4.71E-08	0.823	29.876
rs6796	C	T	0.046	0.002	1.66E-102	0.274	462.451
rs17700436	T	C	-0.028	0.004	1.53E-12	0.057	50.080
rs10238435	T	C	-0.024	0.002	4.23E-25	0.240	107.235
rs17544073	T	C	0.011	0.002	1.92E-09	0.449	36.128
rs41430449	G	C	-0.030	0.004	1.82E-15	0.066	63.352
rs3731332	T	C	-0.108	0.006	4.01E-64	0.023	286.062
rs146039611	A	G	0.071	0.007	3.07E-24	0.019	103.301
rs7787179	G	A	0.021	0.002	3.31E-19	0.207	80.381
rs35108724	A	G	-0.023	0.004	2.63E-08	0.057	31.006
rs113155021	A	G	-0.048	0.008	2.21E-09	0.015	35.837
rs16939607	A	G	-0.025	0.003	2.43E-21	0.146	90.078
rs45577137	G	A	-0.065	0.005	9.05E-39	0.044	169.771
rs12542907	G	C	-0.024	0.002	1.01E-36	0.399	160.351
rs72673751	C	T	-0.017	0.002	4.35E-12	0.190	48.020
rs1954735	C	T	0.021	0.003	1.01E-16	0.168	69.048
rs2978889	A	G	-0.019	0.002	3.43E-24	0.504	103.079
rs7836456	G	T	-0.015	0.002	1.26E-14	0.514	59.535
rs62501136	A	G	0.020	0.002	1.72E-19	0.221	81.668
rs113015223	T	C	0.019	0.002	1.06E-15	0.211	64.402
rs7824937	G	A	0.018	0.002	1.03E-19	0.369	82.695
rs149902937	A	G	-0.019	0.003	1.21E-08	0.095	32.522
rs1863651	T	A	-0.017	0.002	4.32E-16	0.699	66.180
rs7836786	A	G	-0.013	0.002	4.46E-08	0.815	29.975
rs79335847	C	G	-0.025	0.003	4.14E-14	0.094	57.198

rs6470759	A	G	-0.018	0.002	5.04E-21	0.589	88.683
rs13267464	T	C	-0.020	0.002	8.90E-27	0.399	114.885
rs4871844	C	T	0.016	0.002	1.27E-14	0.343	59.489
rs62502392	A	G	-0.018	0.003	2.08E-11	0.143	44.980
rs4737009	A	G	0.015	0.002	3.44E-12	0.236	48.500
rs145718079	A	G	-0.080	0.010	4.68E-16	0.010	66.030
rs62513343	T	C	-0.015	0.003	2.17E-08	0.142	31.381
rs10094039	G	A	-0.091	0.002	1.00E-200	0.617	2251.109
rs10099546	G	A	-0.020	0.003	4.33E-13	0.143	52.565
rs7826487	G	A	-0.041	0.003	2.17E-43	0.116	190.936
rs3808609	C	G	0.017	0.002	3.86E-17	0.318	70.927
rs13271228	G	T	-0.031	0.002	4.35E-59	0.567	262.900
rs2385094	C	T	-0.024	0.003	9.73E-17	0.122	69.137
rs11557154	T	C	0.032	0.003	2.64E-30	0.128	131.075
rs10814193	C	A	0.015	0.002	1.07E-12	0.716	50.809
rs34478611	A	G	0.013	0.002	7.35E-10	0.242	37.970
rs79446921	A	G	-0.023	0.004	3.38E-08	0.054	30.525
rs7030655	T	G	0.020	0.002	2.66E-23	0.309	98.992
rs10780209	A	G	0.045	0.002	5.69E-131	0.479	593.397
rs183363123	T	C	0.063	0.007	3.44E-21	0.021	89.394
rs10123018	A	G	0.075	0.002	1.00E-200	0.582	1597.369
rs1930303	A	G	-0.062	0.002	1.00E-200	0.663	952.957
rs11792030	A	G	0.022	0.003	1.00E-12	0.104	50.915
rs505922	C	T	-0.044	0.002	5.75E-106	0.319	478.098
rs3731211	A	T	0.028	0.002	2.51E-42	0.720	186.012
rs12376511	C	T	-0.049	0.003	3.44E-82	0.164	369.067
rs7861055	G	A	-0.014	0.002	5.66E-14	0.488	56.547
rs9410425	A	G	-0.030	0.002	3.23E-51	0.325	226.793
rs72759267	T	C	0.021	0.003	5.36E-16	0.159	65.773
rs2253843	G	A	-0.013	0.002	9.39E-10	0.278	37.512
rs41381344	A	G	0.030	0.005	2.02E-08	0.037	31.525
rs290243	A	G	0.014	0.002	3.00E-10	0.215	39.750
rs475616	G	A	0.016	0.002	2.97E-15	0.656	62.402
rs7919533	C	T	0.020	0.002	1.55E-26	0.488	113.852
rs7095778	G	A	-0.020	0.003	3.56E-10	0.091	39.397
rs11252148	A	G	0.012	0.002	4.79E-10	0.484	38.822
rs1749824	A	C	-0.012	0.002	1.26E-09	0.432	36.918
rs11189154	A	G	0.051	0.002	1.25E-122	0.256	554.672
rs11191206	C	G	0.011	0.002	8.15E-09	0.453	33.297
rs111456533	A	G	-0.014	0.003	1.70E-08	0.164	31.853
rs11255548	C	T	0.020	0.002	2.99E-25	0.379	107.918

rs10828725	T	G	-0.047	0.002	3.56E-121	0.368	548.031
rs17011726	G	C	-0.023	0.002	1.23E-24	0.233	105.124
rs1180658	A	C	-0.013	0.002	1.19E-10	0.634	41.534
rs224111	A	G	-0.021	0.002	1.16E-27	0.390	118.878
rs1781799	T	C	0.011	0.002	7.83E-09	0.613	33.383
rs1992057	A	G	0.012	0.002	2.34E-10	0.432	40.220
rs74233809	C	T	-0.027	0.003	3.34E-15	0.081	62.146
rs17776203	C	A	-0.011	0.002	3.07E-08	0.443	30.726
rs17731	A	G	-0.014	0.002	1.45E-13	0.374	54.728
rs2646421	C	G	-0.026	0.002	3.28E-41	0.378	181.047
rs72790862	C	T	-0.020	0.002	1.92E-23	0.307	99.695
rs7097656	C	T	0.020	0.002	2.80E-18	0.795	76.162
rs2184697	G	T	-0.015	0.002	1.23E-12	0.293	50.506
rs11190141	T	C	-0.044	0.002	4.02E-113	0.372	511.151
rs7094871	G	C	-0.014	0.002	4.71E-14	0.543	56.942
rs11246065	G	A	-0.022	0.002	1.60E-26	0.339	113.726
rs907612	T	C	-0.033	0.002	5.19E-60	0.380	267.213
rs9783374	C	T	0.021	0.003	6.69E-15	0.849	60.791
rs1885525	A	G	0.013	0.002	7.62E-12	0.506	46.946
rs11235689	T	C	-0.016	0.002	4.79E-16	0.414	65.945
rs6591578	A	G	0.022	0.002	2.03E-29	0.634	126.942
rs7111769	C	A	-0.029	0.004	1.99E-15	0.075	63.174
rs10892342	C	T	-0.018	0.002	7.02E-18	0.276	74.291
rs10902106	C	T	0.013	0.002	1.53E-12	0.540	50.096
rs2957873	A	G	-0.021	0.002	9.44E-19	0.809	78.282
rs573790	C	T	-0.016	0.002	1.48E-16	0.614	68.319
rs10831507	C	A	0.011	0.002	2.15E-08	0.390	31.399
rs71482156	T	A	0.056	0.008	4.71E-12	0.016	47.881
rs662333	A	G	-0.014	0.002	1.05E-09	0.780	37.295
rs695113	T	C	0.021	0.002	1.04E-24	0.697	105.507
rs10796828	G	T	-0.018	0.002	3.26E-20	0.635	84.951
rs11602323	G	T	0.065	0.003	9.23E-115	0.123	518.825
rs7120300	T	C	-0.017	0.002	6.41E-16	0.735	65.393
rs9734613	G	A	0.029	0.005	1.24E-08	0.964	32.472
rs7971382	A	G	0.012	0.002	3.34E-09	0.374	35.020
rs4763944	A	G	0.013	0.002	1.77E-08	0.204	31.765
rs4082413	G	C	0.015	0.002	2.06E-16	0.512	67.669
rs6581124	A	G	-0.013	0.002	8.40E-09	0.245	33.242
rs11104881	C	T	-0.016	0.002	3.35E-14	0.702	57.615
rs4365101	A	G	-0.027	0.004	1.14E-12	0.068	50.666
rs3184504	C	T	-0.047	0.002	1.58E-139	0.517	632.364

rs10849020	G	C	-0.024	0.002	1.28E-25	0.210	109.579
rs706819	C	T	0.018	0.002	1.29E-17	0.738	73.136
rs11170652	A	G	-0.018	0.002	2.77E-14	0.195	57.969
rs35979828	T	C	-0.046	0.004	1.28E-35	0.071	155.311
rs10437954	A	G	-0.018	0.003	4.59E-08	0.905	29.934
rs1800973	A	C	0.113	0.004	1.64E-187	0.062	853.244
rs12306790	T	C	0.019	0.002	2.22E-20	0.715	85.728
rs10849448	G	A	-0.050	0.002	2.77E-111	0.753	502.913
rs2734442	A	G	-0.042	0.003	3.00E-49	0.876	217.774
rs919217	C	T	-0.015	0.003	3.38E-08	0.149	30.517
rs7308348	C	T	0.015	0.002	4.78E-10	0.182	38.820
rs73067080	C	T	-0.016	0.003	7.11E-10	0.160	38.038
rs2651369	G	C	0.014	0.002	1.45E-12	0.326	50.178
rs9943753	G	A	0.014	0.002	4.79E-12	0.628	47.831
rs73201961	C	A	0.027	0.003	1.44E-16	0.089	68.361
rs2535393	C	T	-0.015	0.002	3.14E-15	0.599	62.303
rs7975680	C	A	0.013	0.002	2.12E-10	0.604	40.430
rs11057841	T	C	0.020	0.003	8.16E-14	0.144	55.859
rs17086239	C	G	-0.069	0.005	5.45E-47	0.041	207.450
rs9526475	C	T	-0.012	0.002	4.90E-09	0.728	34.292
rs60699901	C	T	0.022	0.003	2.02E-13	0.118	54.077
rs2504209	G	A	0.018	0.002	3.58E-14	0.811	57.480
rs76428106	C	T	0.536	0.009	1.00E-200	0.013	3868.507
rs138028125	G	C	0.135	0.006	6.19E-131	0.035	593.142
rs73217470	G	A	0.065	0.004	2.11E-47	0.047	209.324
rs1146933	C	T	-0.014	0.002	8.88E-10	0.786	37.623
rs9555596	C	T	0.023	0.002	1.55E-32	0.383	141.201
rs1892548	C	T	-0.063	0.002	1.00E-200	0.655	998.804
rs58814158	G	T	-0.024	0.003	3.24E-19	0.157	80.410
rs55860328	A	G	-0.014	0.002	2.88E-11	0.682	44.336
rs9532580	C	T	-0.028	0.002	9.72E-40	0.264	174.257
rs201905276	A	G	-0.026	0.004	1.25E-10	0.064	41.455
rs9564870	C	A	0.032	0.005	5.24E-12	0.053	47.671
rs188175496	T	C	-0.093	0.007	2.93E-43	0.024	190.380
rs150042404	T	C	0.055	0.010	2.92E-08	0.013	30.812
rs77733744	A	G	-0.014	0.002	1.96E-08	0.172	31.577
rs17498743	G	T	-0.018	0.002	2.27E-13	0.174	53.815
rs12874404	G	A	-0.065	0.004	4.19E-55	0.056	244.636
rs113143864	T	C	0.035	0.004	3.61E-21	0.071	89.281
rs11844354	G	A	-0.013	0.002	2.32E-08	0.226	31.251
rs2063996	C	T	-0.011	0.002	6.20E-09	0.626	33.811

rs1052484	C	G	0.014	0.002	1.28E-09	0.216	36.889
rs61985545	A	G	-0.011	0.002	1.18E-09	0.495	37.076
rs4905043	A	G	0.019	0.002	1.31E-20	0.400	86.755
rs754388	C	G	0.030	0.002	6.43E-36	0.811	156.689
rs11629297	G	C	0.061	0.002	3.73E-178	0.733	809.923
rs12147629	G	A	0.017	0.002	2.90E-16	0.312	66.966
rs2239630	G	A	0.049	0.002	4.68E-146	0.556	662.931
rs2038700	C	T	0.040	0.002	2.81E-96	0.394	433.936
rs4983387	A	G	0.019	0.003	2.20E-09	0.899	35.834
rs6493575	T	C	-0.012	0.002	1.86E-10	0.396	40.666
rs11631419	T	C	-0.012	0.002	7.16E-10	0.573	38.030
rs62011334	G	A	0.012	0.002	2.10E-09	0.355	35.952
rs7180079	G	A	0.059	0.003	3.41E-95	0.877	428.863
rs60695341	T	C	-0.018	0.002	1.14E-14	0.197	59.730
rs11854390	T	C	0.042	0.002	1.26E-111	0.562	504.110
rs7180804	A	G	-0.077	0.002	1.00E-200	0.257	1308.332
rs72743796	A	G	0.041	0.005	9.92E-14	0.031	55.472
rs62018159	A	G	0.015	0.002	1.42E-10	0.209	41.184
rs1002774	A	G	-0.054	0.003	4.48E-73	0.112	327.166
rs11857609	C	T	0.023	0.002	3.99E-26	0.760	111.966
rs4886615	G	A	-0.019	0.002	4.10E-19	0.724	79.959
rs73467599	C	T	0.018	0.002	5.95E-15	0.199	61.016
rs205422	C	T	0.013	0.002	9.49E-10	0.697	37.466
rs731707	C	T	0.047	0.002	5.31E-134	0.489	606.964
rs1967309	G	A	-0.022	0.002	1.43E-30	0.603	132.321
rs12934481	T	G	0.016	0.002	5.28E-12	0.202	47.642
rs391023	T	C	-0.076	0.002	1.00E-200	0.366	1527.629
rs2176777	C	G	0.055	0.002	5.52E-112	0.813	506.146
rs7185007	T	C	-0.017	0.002	2.40E-14	0.240	58.242
rs34746889	C	G	-0.058	0.007	2.00E-15	0.017	63.157
rs16940328	A	G	0.039	0.003	5.15E-38	0.112	166.300
rs11644125	T	C	-0.019	0.002	4.73E-24	0.598	102.461
rs72805138	A	G	0.030	0.003	3.70E-18	0.091	75.570
rs8049116	T	C	0.023	0.003	2.52E-15	0.118	62.711
rs7196129	C	T	0.025	0.002	2.45E-39	0.530	172.442
rs11508026	T	C	-0.012	0.002	2.70E-10	0.431	39.950
rs36026517	C	G	-0.017	0.002	1.02E-12	0.199	50.883
rs4411554	G	C	0.013	0.002	2.58E-09	0.714	35.540
rs62057793	A	G	-0.019	0.003	8.00E-11	0.112	42.321
rs2302774	T	G	-0.024	0.002	5.68E-36	0.383	156.936
rs62054807	T	C	0.020	0.002	2.45E-22	0.295	94.581

rs11655888	T	C	-0.014	0.003	2.83E-08	0.180	30.878
rs12941356	G	A	-0.017	0.002	2.45E-18	0.588	76.415
rs2729450	C	T	-0.031	0.002	1.36E-59	0.524	265.251
rs1594541	C	T	0.016	0.002	3.12E-11	0.196	44.161
rs34097845	T	C	-0.101	0.004	2.99E-124	0.056	562.426
rs2665405	A	G	0.038	0.002	5.37E-93	0.548	418.851
rs9898876	T	G	0.018	0.002	9.89E-14	0.197	55.482
rs71368117	C	T	-0.024	0.003	1.58E-11	0.087	45.490
rs745570	G	A	0.014	0.002	5.38E-13	0.514	52.154
rs9915112	G	A	-0.027	0.002	4.21E-30	0.203	130.124
rs1869365	C	T	0.021	0.002	4.54E-21	0.231	88.847
rs8077619	A	T	-0.019	0.003	5.39E-12	0.133	47.622
rs16978176	C	T	-0.025	0.002	2.89E-40	0.465	176.647
rs9674881	T	C	0.063	0.002	2.16E-189	0.486	861.764
rs118083884	A	G	-0.097	0.009	1.01E-29	0.017	128.380
rs1016680	C	A	-0.026	0.002	4.31E-39	0.637	171.232
rs370639456	A	C	0.021	0.002	2.12E-23	0.679	99.494
rs2084312	T	C	0.038	0.002	8.97E-58	0.800	256.925
rs2846573	T	C	-0.011	0.002	4.51E-08	0.352	29.949
rs745822	G	T	0.016	0.002	3.16E-17	0.376	71.333
rs238136	G	A	-0.015	0.002	1.68E-11	0.250	45.390
rs2013605	T	G	0.018	0.003	5.27E-09	0.102	34.132
rs11082397	G	T	0.019	0.003	1.36E-11	0.129	45.799
rs718515	A	G	-0.020	0.002	5.66E-26	0.552	111.201
rs9963693	C	T	0.013	0.002	5.39E-10	0.261	38.579
rs3177609	C	T	0.031	0.004	8.26E-19	0.078	78.549
rs12327253	G	A	0.018	0.002	4.05E-17	0.294	70.888
rs17758695	T	C	-0.109	0.006	3.89E-80	0.029	359.641
rs10460159	C	T	-0.016	0.002	9.88E-17	0.429	69.102
rs2007483	A	T	-0.024	0.002	1.63E-34	0.382	150.347
rs72970406	G	A	-0.019	0.003	3.24E-08	0.087	30.606
rs954954	C	A	-0.029	0.003	4.20E-21	0.105	89.005
rs10403909	G	A	0.019	0.002	1.95E-17	0.724	72.311
rs79407714	A	G	-0.064	0.006	1.28E-28	0.030	123.323
rs4577206	T	C	-0.021	0.003	6.50E-11	0.889	42.720
rs12461422	T	C	-0.016	0.002	3.77E-13	0.283	52.849
rs149287489	G	A	-0.032	0.004	9.36E-18	0.079	73.752
rs76427287	C	T	0.025	0.002	3.04E-26	0.254	112.423
rs79939614	T	C	0.012	0.002	4.70E-08	0.646	29.890
rs11086102	C	G	0.031	0.002	2.26E-54	0.633	241.347
rs56344893	A	C	-0.044	0.002	2.12E-114	0.373	516.966

rs143273199	C	T	-0.067	0.002	1.00E-200	0.259	969.368
rs72987040	A	C	-0.025	0.003	2.71E-18	0.124	76.227
rs10409752	G	T	0.013	0.002	4.74E-08	0.794	29.861
rs7249692	C	T	-0.017	0.002	8.83E-18	0.671	73.853
rs10420703	C	T	0.018	0.002	1.49E-13	0.808	54.653
rs74259566	G	C	0.029	0.003	7.51E-20	0.101	83.311
rs413141	G	A	0.043	0.003	1.42E-54	0.863	242.171
rs571497	A	G	-0.028	0.003	8.94E-27	0.155	114.933
rs3859570	C	T	-0.028	0.002	3.97E-44	0.425	194.279
rs62143205	A	G	-0.058	0.003	2.18E-70	0.104	314.791
rs2327028	T	C	-0.011	0.002	2.80E-09	0.475	35.359
rs6055955	T	C	-0.029	0.002	8.72E-54	0.508	238.731
rs6120950	T	C	-0.019	0.002	1.45E-16	0.203	68.357
rs12480462	T	C	-0.031	0.002	1.44E-52	0.316	233.065
rs932641	A	G	-0.014	0.002	4.99E-10	0.251	38.732
rs17196752	T	C	-0.072	0.002	8.09E-199	0.190	905.337
rs6512627	A	G	-0.047	0.002	1.83E-88	0.194	397.949
rs6044081	G	A	0.012	0.002	2.00E-08	0.758	31.531
rs12480732	T	C	0.054	0.002	8.57E-136	0.258	615.482
rs628965	G	A	-0.013	0.002	1.84E-10	0.631	40.703
rs2295481	T	C	-0.015	0.002	7.15E-11	0.207	42.551
rs34600126	T	G	0.022	0.002	6.03E-23	0.218	97.439
rs4813619	T	G	0.017	0.002	4.24E-18	0.511	75.302
rs4142441	G	A	0.031	0.003	1.22E-31	0.152	137.158
rs4810832	A	G	0.013	0.002	4.34E-10	0.325	39.027
rs932905	A	G	0.021	0.002	6.11E-24	0.282	101.949
rs35068491	C	T	0.015	0.002	5.68E-10	0.209	38.498
rs2834670	G	A	0.027	0.003	1.11E-19	0.176	82.524
rs57221391	G	T	-0.022	0.004	1.51E-09	0.071	36.584
rs1997577	T	A	-0.030	0.003	5.80E-30	0.155	129.431
rs73203055	G	C	-0.026	0.004	1.27E-12	0.072	50.467
rs2836220	C	A	-0.011	0.002	1.01E-08	0.338	32.891
rs139271	C	T	-0.015	0.002	1.11E-15	0.442	64.327
rs41409548	A	G	-0.096	0.006	1.49E-64	0.034	288.091
rs181407	G	C	0.011	0.002	4.47E-09	0.512	34.477
rs739241	A	G	0.018	0.002	1.72E-20	0.607	86.162
rs9625746	C	G	-0.021	0.002	7.42E-28	0.413	119.881
rs146681239	C	T	-0.012	0.002	2.01E-09	0.399	36.018
rs1468488	C	T	0.033	0.002	3.36E-52	0.273	231.383
rs34505104	G	A	-0.033	0.002	6.38E-57	0.305	252.982
rs146474125	A	G	-0.027	0.005	2.99E-08	0.041	30.760

	rs47341	T	C	0.031	0.002	1.46E-54	0.396	242.232
Neutrophil count	rs17440390	T	C	0.016	0.003	1.35E-08	0.126	32.312
	rs116631966	A	G	0.040	0.006	1.02E-10	0.025	41.855
	rs60124939	T	C	-0.018	0.003	1.52E-12	0.176	50.090
	rs4844622	T	C	-0.023	0.002	1.44E-25	0.240	109.322
	rs4626924	T	C	0.019	0.002	4.99E-23	0.551	97.751
	rs284317	G	A	0.014	0.002	1.60E-11	0.496	45.485
	rs10917107	A	G	-0.007	0.001	1.14E-08	0.687	32.632
	rs12118277	T	C	0.015	0.002	1.15E-13	0.352	55.185
	rs74076327	C	T	-0.039	0.005	7.85E-17	0.045	69.545
	rs55851397	A	C	0.012	0.002	1.18E-09	0.365	37.044
	rs520910	C	T	-0.022	0.004	6.63E-09	0.067	33.701
	rs41272536	G	A	-0.036	0.005	2.33E-13	0.046	53.794
	rs16843353	C	G	-0.036	0.006	8.84E-10	0.030	37.618
	rs17728	C	T	0.024	0.003	1.23E-12	0.909	50.502
	rs35915186	C	T	0.024	0.002	3.67E-25	0.215	107.487
	rs11580229	A	G	-0.023	0.002	2.76E-30	0.462	130.960
	rs486650	T	C	-0.014	0.002	5.19E-13	0.469	52.188
	rs112203773	A	C	-0.012	0.002	7.70E-09	0.083	33.395
	rs3917932	G	C	-0.052	0.002	3.00E-154	0.577	700.465
	rs55873273	C	T	0.059	0.007	6.24E-18	0.020	74.563
	rs150649461	C	G	0.053	0.008	1.90E-10	0.015	40.638
	rs694180	G	A	0.019	0.002	1.24E-20	0.656	86.849
	rs3014874	A	G	0.012	0.002	2.81E-08	0.265	30.873
	rs3843301	G	T	-0.019	0.002	8.70E-20	0.365	82.989
	rs1886654	C	T	-0.058	0.003	3.08E-80	0.892	360.013
	rs4925756	T	C	-0.013	0.002	5.14E-10	0.692	38.664
	rs301817	A	C	-0.014	0.002	5.89E-13	0.583	51.940
	rs1135172	G	A	0.012	0.002	2.46E-08	0.730	31.151
	rs3754224	C	T	-0.017	0.002	2.70E-14	0.270	58.034
	rs6672898	A	G	-0.019	0.002	2.92E-23	0.435	98.884
	rs41313381	A	C	0.059	0.005	1.16E-26	0.031	114.364
	rs7555995	G	C	-0.015	0.002	2.86E-12	0.285	48.855
	rs34599082	T	C	-0.173	0.008	7.27E-94	0.013	422.654
	rs114427170	A	G	0.032	0.005	2.81E-09	0.037	35.368
	rs2312675	C	T	-0.020	0.002	2.61E-17	0.212	71.739
	rs34990593	C	T	0.014	0.002	1.13E-08	0.189	32.655
	rs56188865	C	T	-0.027	0.002	1.93E-43	0.374	191.105
	rs10864368	C	T	0.024	0.002	4.01E-35	0.503	153.104
	rs3762297	T	C	0.024	0.003	2.83E-21	0.183	89.776
	rs6678033	A	G	-0.043	0.002	1.46E-102	0.368	462.443

rs6679677	A	C	-0.038	0.003	8.69E-33	0.100	142.379
rs68002561	G	A	0.020	0.003	2.43E-09	0.091	35.657
rs2808519	T	G	0.016	0.002	1.50E-13	0.738	54.654
rs35020138	C	T	-0.018	0.002	1.42E-17	0.675	72.939
rs7590263	T	C	0.012	0.002	1.76E-09	0.497	36.272
rs77552263	A	G	0.049	0.004	7.19E-42	0.078	183.943
rs71409899	T	A	0.023	0.003	2.66E-15	0.134	62.612
rs114050631	T	C	-0.163	0.010	8.74E-59	0.011	261.549
rs79047930	T	C	0.053	0.008	1.64E-10	0.015	40.914
rs12328347	G	T	-0.016	0.002	7.97E-16	0.373	64.957
rs2729707	G	A	-0.036	0.003	2.68E-44	0.831	195.046
rs2632372	C	T	-0.021	0.002	6.68E-28	0.504	120.048
rs2052249	A	C	-0.013	0.002	1.23E-08	0.251	32.484
rs6432335	G	T	0.012	0.002	2.42E-10	0.452	40.153
rs72789923	C	A	0.020	0.002	6.95E-23	0.307	97.092
rs1260326	C	T	-0.033	0.002	6.91E-62	0.602	275.715
rs116447416	G	A	-0.037	0.005	1.47E-12	0.035	50.157
rs11691193	T	C	0.015	0.002	2.52E-10	0.218	40.061
rs6734238	G	A	0.039	0.002	1.58E-90	0.403	407.417
rs354703	C	T	0.015	0.002	4.08E-14	0.593	57.213
rs3856364	G	C	0.014	0.002	5.28E-12	0.680	47.649
rs6740847	G	A	0.022	0.002	3.95E-29	0.562	125.671
rs14243	G	A	0.014	0.002	4.66E-08	0.211	29.904
rs4074882	C	T	0.028	0.002	5.61E-37	0.742	161.583
rs4632345	A	G	-0.012	0.002	3.24E-09	0.678	35.068
rs6723009	C	T	0.012	0.002	2.15E-08	0.717	31.408
rs75475627	G	C	0.033	0.004	3.76E-19	0.077	80.119
rs11894199	A	C	0.011	0.002	2.07E-08	0.388	31.477
rs11689257	A	G	-0.025	0.004	2.51E-10	0.070	40.074
rs62189859	C	T	0.014	0.002	2.96E-10	0.244	39.760
rs1047891	A	C	-0.019	0.002	8.79E-21	0.315	87.500
rs11690365	G	T	0.012	0.002	3.21E-09	0.640	35.115
rs7575465	A	G	-0.013	0.002	5.71E-11	0.553	42.989
rs2421200	T	G	-0.021	0.002	1.03E-26	0.487	114.636
rs6731993	T	A	0.023	0.002	2.45E-31	0.408	135.726
rs17026212	G	T	-0.017	0.002	1.62E-18	0.513	77.196
rs796056	C	A	0.022	0.002	1.43E-29	0.624	127.734
rs35789178	G	T	0.018	0.002	3.41E-13	0.186	53.037
rs11683933	A	C	0.031	0.004	5.72E-13	0.055	52.030
rs188653407	G	C	-0.051	0.006	2.58E-15	0.023	62.649
rs13392977	A	G	0.028	0.004	3.73E-11	0.054	43.812

rs55729107	G	A	-0.018	0.002	7.87E-14	0.226	55.914
rs1366045	C	T	-0.022	0.002	1.20E-28	0.385	123.493
rs6414435	G	A	0.017	0.002	5.57E-17	0.592	70.250
rs12487658	T	C	0.017	0.002	2.06E-12	0.802	49.516
rs11721064	T	G	0.014	0.002	3.22E-13	0.609	53.140
rs2734031	C	T	-0.068	0.003	4.20E-91	0.908	409.906
rs2046934	A	G	0.015	0.002	1.66E-09	0.823	36.397
rs9842724	C	T	-0.014	0.002	3.67E-09	0.800	34.849
rs56174170	C	T	0.017	0.003	2.04E-09	0.139	35.983
rs59519784	A	G	0.012	0.002	1.85E-09	0.410	36.178
rs7626444	C	G	-0.021	0.002	1.27E-26	0.422	114.257
rs56217149	A	G	0.017	0.003	4.45E-10	0.151	38.963
rs6779340	G	C	-0.015	0.002	8.69E-14	0.337	55.751
rs7639292	T	C	-0.022	0.003	4.83E-18	0.169	75.075
rs6764912	A	G	-0.037	0.004	3.14E-20	0.065	85.033
rs11712552	G	A	0.030	0.003	6.45E-26	0.126	110.951
rs9819371	T	C	-0.043	0.004	3.64E-28	0.065	121.264
rs789858	T	C	0.014	0.002	1.07E-12	0.404	50.779
rs58605236	A	T	-0.014	0.002	2.53E-12	0.373	49.077
rs10049210	C	T	0.015	0.002	7.54E-15	0.403	60.565
rs11716015	T	C	-0.013	0.002	1.16E-10	0.636	41.619
rs3749440	G	A	0.017	0.002	2.20E-18	0.372	76.580
rs218264	T	A	0.032	0.002	5.59E-46	0.251	202.829
rs7684253	T	C	0.014	0.002	3.29E-13	0.550	53.105
rs16850073	T	C	0.052	0.002	2.12E-149	0.374	678.080
rs4145952	A	C	-0.013	0.002	1.06E-11	0.399	46.273
rs2290846	A	G	0.019	0.002	9.92E-19	0.284	78.156
rs6817881	T	C	0.014	0.002	1.35E-13	0.499	54.856
rs35734242	C	T	0.023	0.002	5.42E-31	0.428	134.110
rs6855981	A	G	-0.016	0.002	1.85E-16	0.373	67.857
rs7658676	T	C	0.011	0.002	3.58E-08	0.353	30.406
rs11723621	G	A	-0.027	0.002	2.67E-37	0.291	163.073
rs7687658	G	A	0.011	0.002	1.14E-08	0.434	32.632
rs7679673	A	C	-0.024	0.002	2.15E-33	0.380	145.216
rs140311179	G	C	-0.025	0.004	1.36E-08	0.051	32.288
rs28530750	A	G	0.056	0.005	2.36E-31	0.043	135.820
rs723585	G	A	-0.022	0.002	2.97E-31	0.484	135.397
rs2522440	C	T	0.019	0.003	2.90E-08	0.912	30.819
rs11735662	T	C	0.048	0.005	5.59E-19	0.034	79.330
rs6831590	T	G	-0.020	0.002	1.15E-24	0.367	105.219
rs10012827	T	C	0.016	0.003	3.81E-08	0.124	30.300

rs1371794	C	A	-0.073	0.002	1.00E-200	0.622	1341.848
rs17213043	G	C	0.046	0.006	1.97E-15	0.028	63.195
rs6877725	C	T	-0.025	0.002	3.97E-38	0.445	166.916
rs6859727	C	T	-0.040	0.003	4.29E-43	0.878	189.613
rs62375385	C	T	0.014	0.002	5.46E-09	0.194	34.066
rs249677	A	C	0.023	0.002	1.47E-31	0.634	136.715
rs9885207	C	T	0.016	0.002	1.22E-16	0.416	68.695
rs10447304	A	G	-0.017	0.002	5.58E-18	0.506	74.747
rs155795	C	G	0.013	0.002	1.71E-11	0.384	45.321
rs464609	A	G	0.011	0.002	3.02E-08	0.544	30.734
rs12658947	G	A	0.013	0.002	3.01E-09	0.257	35.239
rs141010613	A	G	-0.044	0.008	2.08E-08	0.017	31.464
rs4535497	A	C	-0.014	0.002	1.75E-12	0.570	49.828
rs7705526	A	C	0.034	0.002	9.19E-54	0.327	238.555
rs62360185	G	A	-0.016	0.002	8.05E-11	0.191	42.298
rs2432142	A	G	0.015	0.002	1.89E-14	0.435	58.764
rs1445171	C	T	0.014	0.002	1.15E-13	0.517	55.161
rs6878780	C	T	0.013	0.002	6.01E-12	0.426	47.423
rs172240	A	G	-0.020	0.003	3.71E-14	0.850	57.395
rs2522051	C	T	0.024	0.002	1.88E-35	0.454	154.634
rs6891328	C	G	0.012	0.002	2.47E-10	0.583	40.098
rs2561758	G	A	-0.034	0.002	3.49E-55	0.723	244.973
rs13190036	G	A	-0.022	0.003	3.63E-12	0.891	48.377
rs1966479	G	A	0.014	0.002	1.21E-12	0.660	50.525
rs2082382	A	G	-0.029	0.002	5.39E-52	0.552	230.360
rs11753041	C	T	0.011	0.002	1.27E-08	0.395	32.423
rs80296299	T	C	0.026	0.003	1.00E-17	0.116	73.619
rs62429983	A	G	0.016	0.003	1.77E-09	0.145	36.282
rs1490384	T	C	-0.015	0.002	2.19E-14	0.501	58.434
rs12214269	A	G	0.019	0.002	6.60E-24	0.545	101.818
rs72978754	C	T	-0.026	0.004	5.38E-11	0.066	43.113
rs10945542	T	C	0.012	0.002	4.82E-10	0.521	38.814
rs4712614	G	T	-0.020	0.002	1.04E-23	0.620	100.912
rs9267575	C	T	-0.026	0.002	3.82E-41	0.398	180.611
rs9378212	T	C	0.060	0.002	1.00E-200	0.469	960.320
rs9277764	A	G	0.024	0.003	3.68E-16	0.122	66.488
rs10948036	A	C	0.030	0.002	3.49E-36	0.210	157.969
rs9479025	T	A	0.011	0.002	4.47E-08	0.519	29.996
rs212409	A	G	-0.021	0.002	4.71E-28	0.554	120.681
rs6915310	T	C	-0.032	0.003	1.71E-36	0.174	159.311
rs9265980	A	G	0.057	0.003	1.62E-115	0.177	522.340

rs12189880	T	C	-0.016	0.002	1.54E-12	0.241	50.079
rs6927569	C	T	0.026	0.002	2.40E-41	0.523	181.526
rs3777755	T	C	0.014	0.002	4.78E-11	0.311	43.341
rs68016381	T	C	-0.034	0.004	2.23E-14	0.051	58.418
rs68137036	G	A	-0.013	0.002	1.02E-09	0.285	37.350
rs915125	T	C	-0.018	0.002	1.92E-16	0.282	67.812
rs6454596	C	G	-0.025	0.002	1.38E-39	0.490	173.482
rs207253	A	G	-0.012	0.002	9.42E-10	0.408	37.484
rs12203592	T	C	0.014	0.002	1.66E-08	0.214	31.907
rs6936191	C	T	0.018	0.002	9.87E-20	0.608	82.784
rs1611236	A	G	-0.019	0.002	3.35E-20	0.323	84.887
rs58005484	T	C	0.033	0.003	5.14E-33	0.144	143.438
rs7738554	A	T	0.015	0.002	5.70E-11	0.251	42.974
rs9402685	C	T	-0.034	0.002	1.05E-52	0.256	233.700
rs6924387	G	A	0.017	0.002	1.73E-18	0.412	77.077
rs9390461	G	A	0.018	0.002	3.49E-20	0.539	84.791
rs56388170	T	G	0.070	0.002	1.00E-200	0.294	1079.205
rs6463566	G	C	-0.017	0.002	2.16E-13	0.230	53.953
rs1474419	C	T	0.018	0.002	2.55E-19	0.579	80.862
rs2710804	C	T	0.020	0.002	3.76E-23	0.376	98.363
rs4727392	G	A	-0.018	0.002	5.65E-16	0.736	65.628
rs7777484	G	A	0.012	0.002	1.96E-09	0.420	36.068
rs2158799	G	C	0.052	0.002	6.53E-152	0.611	689.776
rs3735485	G	A	0.029	0.003	4.20E-28	0.845	120.934
rs778732	T	C	0.027	0.003	6.53E-18	0.107	74.477
rs4729046	C	T	-0.043	0.004	6.84E-32	0.924	138.303
rs2301557	T	C	-0.126	0.007	2.53E-68	0.018	305.369
rs9656395	G	A	-0.023	0.003	4.08E-12	0.095	48.148
rs7803075	G	A	-0.019	0.002	2.19E-18	0.735	76.636
rs73049276	A	G	-0.040	0.004	5.13E-22	0.059	93.185
rs12530608	G	A	-0.016	0.003	3.63E-09	0.146	34.861
rs10252457	G	A	-0.014	0.002	1.82E-12	0.426	49.752
rs17139597	G	T	-0.034	0.005	3.23E-12	0.040	48.614
rs33951980	T	C	-0.028	0.003	6.63E-22	0.129	92.662
rs76873475	A	C	-0.015	0.003	1.18E-08	0.156	32.575
rs3731332	T	C	-0.120	0.007	9.69E-75	0.023	334.851
rs7776857	T	G	-0.015	0.002	3.16E-13	0.657	53.181
rs981132	G	T	0.021	0.002	7.88E-25	0.678	106.017
rs342242	C	T	-0.016	0.002	3.62E-16	0.453	66.541
rs35759345	T	C	0.013	0.002	6.19E-11	0.450	42.846
rs3735311	C	T	-0.020	0.002	2.82E-22	0.649	94.334

rs1991651	G	C	0.023	0.002	7.40E-31	0.618	133.546
rs34215892	A	G	0.038	0.006	2.30E-10	0.028	40.254
rs6468341	C	T	0.019	0.002	7.10E-18	0.743	74.307
rs7815046	C	A	-0.013	0.002	4.65E-10	0.303	38.873
rs11997631	G	C	0.017	0.002	1.74E-14	0.274	58.905
rs12541521	G	A	0.013	0.002	4.77E-11	0.450	43.342
rs6985508	A	G	-0.027	0.002	3.36E-39	0.358	171.736
rs12716647	C	G	0.014	0.002	1.03E-12	0.637	50.879
rs609264	G	T	0.013	0.002	7.93E-11	0.528	42.347
rs11993347	C	T	-0.022	0.002	3.36E-21	0.230	89.419
rs12550612	A	G	-0.031	0.003	1.09E-33	0.822	146.478
rs45577137	G	A	0.036	0.005	2.63E-12	0.045	49.013
rs7846314	T	A	0.064	0.002	4.70E-149	0.187	676.272
rs4276676	C	T	-0.018	0.002	1.16E-20	0.470	87.030
rs2923416	G	A	0.011	0.002	4.21E-08	0.610	30.097
rs28571765	C	T	-0.022	0.002	3.80E-21	0.206	89.202
rs7816785	T	C	-0.021	0.002	4.31E-27	0.606	116.392
rs4734879	G	A	-0.021	0.002	2.63E-23	0.276	99.069
rs55964818	C	T	-0.036	0.002	3.94E-78	0.568	350.369
rs7005996	T	C	0.023	0.003	3.33E-11	0.906	44.047
rs6998846	A	G	0.015	0.002	4.54E-11	0.783	43.422
rs16939607	A	G	-0.028	0.003	1.99E-25	0.146	108.731
rs73271394	C	T	0.026	0.005	3.39E-08	0.045	30.517
rs3739873	A	G	-0.014	0.002	3.45E-09	0.220	34.964
rs17831500	C	A	-0.020	0.002	5.64E-18	0.224	74.767
rs10811668	A	C	-0.014	0.002	1.56E-08	0.208	32.037
rs626416	C	G	-0.020	0.002	5.77E-18	0.762	74.673
rs796007	A	G	-0.021	0.002	9.41E-21	0.253	87.398
rs10992394	A	G	0.014	0.002	1.14E-09	0.231	37.146
rs1411424	A	G	-0.011	0.002	2.51E-09	0.523	35.577
rs4413892	A	G	0.034	0.002	1.65E-51	0.278	228.161
rs9411293	G	C	-0.014	0.002	4.19E-11	0.337	43.567
rs409801	C	T	0.021	0.002	8.59E-28	0.503	119.521
rs3793537	C	G	0.013	0.002	2.55E-09	0.291	35.548
rs10868825	A	T	0.012	0.002	2.60E-08	0.338	31.027
rs10760690	T	G	0.011	0.002	1.85E-08	0.374	31.697
rs117468663	T	A	0.019	0.003	8.74E-12	0.134	46.658
rs7852409	G	C	0.018	0.002	7.62E-14	0.765	55.986
rs6476883	A	G	-0.014	0.002	4.96E-13	0.439	52.306
rs1887428	C	G	-0.014	0.002	1.39E-12	0.625	50.287
rs13291664	G	A	0.029	0.002	1.19E-32	0.188	141.766

	rs7866863	A	G	0.013	0.002	1.00E-10	0.341	41.881
	rs12378064	T	C	-0.014	0.002	1.99E-11	0.650	45.043
	rs2519093	T	C	-0.040	0.002	9.83E-59	0.185	261.434
	rs2157770	G	A	0.021	0.002	2.67E-21	0.287	89.881
	rs692594	C	G	0.012	0.002	8.23E-11	0.490	42.284
	rs2807742	A	G	0.033	0.002	1.16E-45	0.771	201.369
	rs1885474	G	T	-0.024	0.003	1.08E-13	0.103	55.305
	rs1412445	T	C	0.021	0.002	2.64E-25	0.338	108.145
	rs1977289	C	T	-0.018	0.002	2.14E-21	0.479	90.309
	rs10509912	A	T	-0.021	0.003	7.02E-14	0.135	56.127
	rs10906393	T	A	0.017	0.002	9.66E-19	0.578	78.272
	rs72790862	C	T	-0.021	0.002	6.71E-24	0.307	101.743
	rs1250568	C	T	-0.014	0.002	8.89E-12	0.435	46.622
	rs72836628	T	C	-0.015	0.002	4.07E-12	0.248	48.149
	rs3781454	A	G	0.024	0.002	6.26E-31	0.678	133.935
	rs1571956	A	G	0.014	0.002	1.43E-11	0.646	45.690
	rs10995477	C	T	-0.030	0.002	2.86E-56	0.474	250.159
	rs3747869	C	A	0.033	0.003	1.62E-22	0.901	95.440
	rs72825306	G	A	0.015	0.003	4.56E-09	0.167	34.417
	rs7917772	A	G	0.016	0.002	5.41E-16	0.631	65.714
	rs180941	A	G	-0.013	0.002	8.33E-11	0.625	42.240
	rs9419387	A	G	-0.012	0.002	2.66E-10	0.405	39.956
	rs9804265	C	A	-0.040	0.002	2.79E-89	0.367	401.602
	rs10786325	G	C	0.042	0.002	6.05E-104	0.596	469.214
	rs10833024	T	C	0.016	0.002	4.51E-15	0.310	61.578
	rs9988894	G	C	0.015	0.002	5.52E-13	0.324	52.104
	rs10750623	A	G	-0.011	0.002	1.28E-08	0.458	32.412
	rs8705	A	G	-0.018	0.002	1.97E-18	0.317	76.797
	rs7115703	A	T	0.044	0.002	5.38E-107	0.514	483.084
	rs4909932	G	A	0.013	0.002	8.36E-12	0.598	46.772
	rs58984522	C	T	-0.011	0.002	4.71E-08	0.346	29.888
	rs11039195	A	G	0.018	0.002	4.53E-19	0.317	79.709
	rs636317	T	C	0.014	0.002	2.34E-13	0.591	53.755
	rs174548	G	C	-0.024	0.002	7.91E-31	0.313	133.362
	rs1939212	T	C	-0.014	0.002	1.62E-11	0.297	45.454
	rs2282611	G	T	-0.013	0.002	1.03E-09	0.320	37.321
	rs7947419	T	C	0.022	0.004	3.27E-08	0.060	30.585
	rs2468832	A	C	0.015	0.002	2.33E-11	0.239	44.752
	rs12802157	C	A	0.013	0.002	1.20E-08	0.252	32.551
	rs7934719	T	C	0.018	0.002	2.82E-20	0.413	85.215
	rs73000965	A	T	0.029	0.002	6.63E-45	0.316	197.851

rs7940176	T	C	-0.017	0.003	4.23E-09	0.124	34.557
rs567385	C	G	-0.011	0.002	4.99E-08	0.610	29.763
rs148713124	T	G	0.088	0.011	5.85E-17	0.009	70.128
rs1159649	G	C	-0.014	0.002	6.57E-11	0.316	42.704
rs2852800	G	C	0.016	0.002	4.65E-13	0.739	52.406
rs10849020	G	C	-0.024	0.002	2.34E-24	0.210	103.865
rs7488780	C	G	-0.014	0.002	1.12E-08	0.205	32.663
rs1245035	A	C	0.016	0.002	3.39E-16	0.628	66.637
rs1042725	T	C	0.013	0.002	3.33E-11	0.494	44.018
rs4761234	C	T	0.022	0.002	4.25E-29	0.484	125.502
rs11104881	C	T	-0.026	0.002	7.84E-35	0.702	151.736
rs610578	G	A	-0.015	0.002	6.27E-13	0.663	51.853
rs61955089	C	T	0.044	0.006	2.79E-13	0.027	53.427
rs61754230	T	C	0.040	0.007	1.96E-08	0.019	31.578
rs10846411	G	A	0.017	0.002	1.08E-15	0.687	64.393
rs28588142	T	C	0.015	0.002	6.60E-10	0.188	38.207
rs3861100	G	A	0.018	0.002	3.43E-19	0.348	80.296
rs706809	C	T	0.021	0.002	2.78E-18	0.777	76.145
rs35864914	T	C	-0.011	0.002	1.01E-08	0.377	32.886
rs3184504	C	T	-0.029	0.002	1.90E-52	0.517	232.581
rs11064881	A	G	-0.034	0.004	2.37E-20	0.073	85.576
rs632887	G	A	0.014	0.002	1.06E-12	0.411	50.797
rs7969023	C	T	-0.011	0.002	6.23E-09	0.406	33.799
rs76603681	A	G	0.041	0.006	2.65E-13	0.032	53.523
rs7987258	A	G	-0.011	0.002	2.67E-08	0.662	30.967
rs9543219	T	C	0.013	0.002	5.06E-11	0.574	43.236
rs150861794	T	C	-0.053	0.008	2.47E-12	0.020	49.145
rs7322886	C	T	0.013	0.002	2.71E-11	0.624	44.426
rs2296028	C	G	-0.015	0.003	2.16E-09	0.175	35.868
rs78738581	A	G	0.021	0.002	1.13E-20	0.232	87.064
rs76428106	C	T	0.104	0.009	6.25E-32	0.013	138.482
rs9508005	G	T	-0.020	0.004	2.18E-08	0.094	31.369
rs2260850	G	T	0.024	0.002	4.43E-28	0.280	120.880
rs4903580	T	C	0.017	0.002	1.16E-17	0.456	73.333
rs72664840	T	C	0.021	0.003	9.21E-17	0.179	69.214
rs2241621	C	A	-0.015	0.002	2.37E-14	0.588	58.283
rs2038700	C	T	0.033	0.002	1.25E-64	0.394	288.516
rs72731564	T	C	0.020	0.002	2.95E-16	0.187	66.935
rs12588718	C	G	0.015	0.002	4.29E-12	0.666	48.048
rs10498635	T	C	-0.030	0.002	8.73E-34	0.184	146.972
rs2494747	T	G	0.013	0.002	3.37E-10	0.615	39.524

rs11625865	A	G	-0.015	0.002	1.26E-13	0.610	54.994
rs34765661	C	T	0.042	0.004	8.58E-27	0.065	114.976
rs72726027	C	T	-0.043	0.003	1.71E-45	0.112	200.652
rs780142	G	T	0.013	0.002	3.84E-09	0.274	34.760
rs7496362	G	C	0.018	0.002	4.26E-18	0.366	75.294
rs8030089	T	C	-0.019	0.002	7.79E-15	0.195	60.501
rs62011281	A	G	0.012	0.002	7.03E-09	0.310	33.592
rs2062250	A	G	0.047	0.004	9.62E-31	0.939	133.049
rs4924450	A	G	0.014	0.002	3.40E-10	0.705	39.481
rs12909505	C	T	-0.021	0.002	3.00E-28	0.429	121.600
rs17381821	A	T	0.013	0.002	1.88E-09	0.281	36.157
rs4843073	T	C	0.014	0.002	1.01E-11	0.645	46.358
rs7183988	G	T	-0.017	0.002	6.26E-18	0.527	74.538
rs67175901	T	C	0.044	0.003	1.99E-43	0.107	191.134
rs2955958	A	G	0.012	0.002	9.67E-09	0.315	32.954
rs11648664	A	G	-0.014	0.002	5.43E-13	0.402	52.151
rs12927351	A	C	0.022	0.002	2.83E-19	0.199	80.656
rs72832055	A	G	-0.012	0.002	3.09E-09	0.313	35.180
rs4984803	A	G	-0.018	0.002	1.34E-17	0.596	73.065
rs6500550	T	C	-0.022	0.002	5.53E-26	0.304	111.272
rs72803323	T	C	0.034	0.005	3.56E-12	0.040	48.437
rs16958642	A	G	0.022	0.004	2.77E-10	0.083	39.894
rs12923918	G	A	-0.010	0.002	4.81E-08	0.509	29.853
rs11574938	C	G	0.020	0.002	1.57E-25	0.520	109.190
rs12930850	G	A	0.019	0.002	1.29E-22	0.537	95.934
rs61739285	T	C	-0.040	0.005	1.15E-13	0.033	55.180
rs1362623	T	C	0.018	0.002	1.49E-14	0.208	59.178
rs305082	C	T	0.037	0.003	3.04E-45	0.172	199.436
rs35929659	C	T	-0.020	0.003	1.21E-13	0.180	55.094
rs75974417	T	C	-0.017	0.002	2.32E-12	0.211	49.279
rs9905106	C	T	0.023	0.002	1.62E-24	0.734	104.577
rs16961474	A	G	-0.017	0.003	1.11E-09	0.137	37.173
rs11080133	A	G	0.012	0.002	2.59E-08	0.306	31.054
rs2314339	T	C	0.033	0.003	8.32E-29	0.125	124.143
rs6503727	C	T	0.019	0.002	2.39E-20	0.661	85.562
rs78813154	T	G	0.019	0.003	7.13E-12	0.144	47.055
rs2525110	T	A	-0.012	0.002	1.93E-09	0.563	36.105
rs149157044	T	C	-0.031	0.005	7.67E-10	0.039	37.905
rs7225843	C	T	-0.022	0.002	2.69E-20	0.203	85.301
rs7214290	A	G	0.018	0.003	1.20E-12	0.173	50.566
rs56378716	G	A	0.129	0.009	3.72E-50	0.013	221.990

rs749780	A	C	0.019	0.002	4.94E-18	0.732	75.026
rs10852834	G	C	-0.037	0.002	9.67E-83	0.478	371.703
rs55767800	C	T	-0.011	0.002	3.87E-08	0.634	30.255
rs2250320	C	G	-0.020	0.002	9.12E-25	0.496	105.689
rs2665405	A	G	0.030	0.002	6.74E-53	0.548	234.516
rs820384	C	G	0.012	0.002	2.40E-08	0.663	31.194
rs72901753	C	G	-0.015	0.002	1.23E-11	0.327	45.989
rs7502100	C	T	0.019	0.002	4.07E-20	0.366	84.502
rs4794820	G	A	0.076	0.002	1.00E-200	0.551	1535.084
rs8079215	T	C	0.012	0.002	2.68E-08	0.722	30.971
rs2001613	T	C	0.012	0.002	2.12E-09	0.472	35.928
rs72973711	T	A	-0.036	0.004	5.53E-21	0.067	88.459
rs4468717	T	C	-0.025	0.004	4.59E-12	0.078	47.919
rs7235882	T	C	0.023	0.003	2.40E-12	0.093	49.189
rs78285907	T	A	0.022	0.003	3.11E-10	0.089	39.669
rs9965539	A	G	0.031	0.003	1.35E-27	0.134	118.631
rs17742008	A	C	-0.018	0.002	4.10E-14	0.212	57.203
rs763362	G	A	0.016	0.002	2.23E-15	0.399	62.936
rs671835	G	A	0.015	0.002	6.86E-14	0.360	56.179
rs303753	A	G	-0.023	0.002	9.57E-29	0.345	123.878
rs11664534	T	C	-0.016	0.002	1.16E-16	0.525	68.761
rs7237878	A	G	-0.012	0.002	1.53E-08	0.690	32.071
rs12460279	G	A	-0.011	0.002	6.43E-09	0.569	33.761
rs56408111	C	T	0.031	0.004	1.11E-18	0.084	77.971
rs8112528	A	G	-0.014	0.002	2.35E-08	0.190	31.238
rs11673093	A	G	-0.049	0.002	1.45E-109	0.260	494.716
rs6511703	C	T	-0.023	0.002	1.66E-24	0.244	104.491
rs386243	T	C	-0.026	0.002	1.03E-30	0.250	132.943
rs7409459	T	C	0.013	0.002	4.15E-09	0.297	34.599
rs571497	A	G	-0.038	0.003	9.76E-45	0.155	197.183
rs4760	G	A	-0.075	0.003	1.42E-171	0.153	780.030
rs930232	A	G	0.021	0.002	2.91E-25	0.460	107.946
rs4808683	G	C	-0.012	0.002	3.67E-10	0.458	39.334
rs1985157	C	T	0.026	0.002	1.29E-38	0.410	169.025
rs4805881	C	A	-0.014	0.002	3.05E-12	0.665	48.723
rs28540102	C	T	0.017	0.002	8.51E-17	0.659	69.417
rs619450	C	T	0.014	0.002	1.29E-12	0.503	50.408
rs4812447	G	A	0.017	0.002	1.49E-18	0.440	77.357
rs12481262	C	T	-0.015	0.002	4.79E-13	0.460	52.356
rs156334	T	C	-0.012	0.002	6.93E-09	0.429	33.598
rs1555275	G	A	-0.014	0.002	2.30E-11	0.650	44.771

	rs2315008	G	T	0.016	0.002	1.64E-15	0.667	63.526
	rs2254458	T	C	-0.013	0.002	1.76E-10	0.645	40.802
	rs6067411	T	C	-0.012	0.002	2.64E-09	0.529	35.479
	rs2179593	A	C	-0.012	0.002	8.62E-09	0.716	33.166
	rs1800961	T	C	-0.052	0.006	8.99E-21	0.031	87.494
	rs2839183	A	G	-0.013	0.002	2.10E-11	0.405	44.961
	rs2824372	C	G	-0.016	0.002	2.21E-10	0.203	40.327
	rs9977672	A	G	-0.022	0.002	7.34E-24	0.259	101.568
	rs1788493	T	C	0.013	0.002	1.88E-11	0.500	45.153
	rs7283930	T	C	-0.013	0.002	7.43E-09	0.739	33.456
	rs5747308	C	A	0.022	0.002	2.30E-30	0.504	131.301
	rs139386	C	T	-0.011	0.002	3.21E-08	0.445	30.630
	rs5753576	T	C	-0.018	0.002	2.43E-20	0.480	85.519
	rs35284073	A	G	0.015	0.002	5.15E-10	0.217	38.674
	rs4347951	C	T	0.012	0.002	1.87E-08	0.311	31.663
	rs738408	T	C	-0.016	0.002	7.74E-12	0.217	46.912
	rs47341	T	C	0.013	0.002	2.26E-10	0.396	40.278
	rs41433144	A	T	-0.035	0.005	1.45E-12	0.043	50.200
	rs200242521	T	C	-0.014	0.002	9.41E-10	0.286	37.494
White blood cell count	rs35755865	T	C	-0.041	0.005	3.18E-16	0.034	66.790
	rs74076327	C	T	-0.029	0.004	1.55E-10	0.045	41.026
	rs61804160	A	T	-0.030	0.003	8.08E-29	0.140	124.214
	rs1779809	T	C	0.014	0.002	1.36E-13	0.334	54.831
	rs4844565	T	A	-0.011	0.002	1.01E-09	0.410	37.343
	rs2615061	A	G	-0.028	0.003	3.82E-22	0.117	93.772
	rs6426584	T	A	-0.012	0.002	3.66E-09	0.335	34.865
	rs34298354	T	C	-0.024	0.003	5.90E-18	0.124	74.676
	rs284317	G	A	0.017	0.002	3.52E-19	0.498	80.261
	rs6684709	C	G	-0.021	0.002	1.84E-27	0.620	118.046
	rs6667709	T	C	-0.014	0.002	6.30E-13	0.370	51.828
	rs778125	G	A	0.014	0.002	7.75E-14	0.355	55.938
	rs10889574	A	G	-0.044	0.002	6.64E-106	0.357	478.114
	rs630505	C	T	0.012	0.002	3.13E-09	0.265	35.160
	rs520910	C	T	-0.025	0.004	1.87E-11	0.067	45.161
	rs4844390	G	A	-0.027	0.002	4.97E-33	0.220	143.534
	rs10864368	C	T	0.022	0.002	1.15E-31	0.503	137.239
	rs12138789	C	T	-0.015	0.002	2.08E-11	0.238	44.957
	rs6429582	C	T	-0.019	0.002	3.25E-24	0.435	103.187
	rs41313381	A	C	0.048	0.006	1.47E-18	0.030	77.394
	rs34599082	T	C	-0.151	0.008	1.93E-77	0.013	347.249
	rs3795503	T	C	-0.015	0.002	1.83E-14	0.315	58.770

rs903123	T	C	0.013	0.002	1.67E-08	0.818	31.910
rs4925756	T	C	-0.015	0.002	3.83E-13	0.692	52.809
rs7549164	T	C	0.025	0.002	1.33E-24	0.189	104.962
rs3917932	G	C	-0.045	0.002	1.22E-127	0.577	577.671
rs3754224	C	T	-0.015	0.002	4.30E-12	0.271	48.046
rs1933295	G	A	-0.016	0.002	1.81E-11	0.777	45.252
rs12142474	G	A	-0.022	0.003	3.05E-10	0.086	39.714
rs150649461	C	G	0.068	0.008	1.33E-17	0.015	73.057
rs4970996	C	G	0.015	0.002	8.97E-13	0.751	51.115
rs10494783	A	G	-0.044	0.004	4.43E-26	0.052	111.726
rs35571080	C	T	0.025	0.002	1.32E-28	0.213	123.266
rs301817	A	C	-0.016	0.002	2.02E-17	0.582	72.250
rs9430574	A	G	-0.014	0.002	1.55E-12	0.330	50.071
rs112750178	C	T	0.015	0.002	2.06E-12	0.239	49.476
rs2104415	A	G	-0.012	0.002	1.99E-09	0.676	36.041
rs12566440	G	A	-0.015	0.002	8.79E-15	0.365	60.256
rs6696259	C	G	0.018	0.002	3.74E-22	0.409	93.821
rs9429767	A	G	0.017	0.002	1.03E-13	0.197	55.396
rs6679677	A	C	-0.052	0.003	1.14E-64	0.100	288.498
rs2312675	C	T	-0.018	0.002	5.11E-15	0.212	61.324
rs55671712	T	C	0.019	0.003	2.59E-09	0.096	35.517
rs41272536	G	A	-0.034	0.005	3.65E-13	0.046	52.913
rs2790122	C	T	0.013	0.002	4.08E-11	0.712	43.629
rs533483	A	G	-0.017	0.002	1.91E-15	0.243	63.234
rs1886654	C	T	-0.057	0.003	5.08E-84	0.892	377.427
rs56188865	C	T	-0.027	0.002	7.00E-46	0.374	202.361
rs7572278	A	T	0.016	0.002	1.01E-11	0.207	46.376
rs72780125	C	T	0.035	0.003	6.75E-36	0.131	156.580
rs60190483	C	T	-0.031	0.004	2.43E-15	0.057	62.790
rs57871178	C	T	-0.013	0.002	3.55E-08	0.176	30.419
rs2729707	G	A	-0.031	0.002	1.28E-36	0.830	159.920
rs10931934	C	T	0.015	0.002	1.38E-15	0.600	63.864
rs1863176	C	G	0.019	0.002	6.27E-24	0.497	101.881
rs1260326	C	T	-0.033	0.002	9.83E-69	0.601	307.192
rs10208769	A	T	0.022	0.002	6.48E-33	0.516	143.048
rs6731993	T	A	0.023	0.002	3.70E-33	0.408	144.140
rs71409899	T	A	0.024	0.003	2.69E-16	0.135	67.130
rs6734238	G	A	0.036	0.002	2.59E-77	0.402	346.673
rs114427331	G	C	-0.036	0.005	6.36E-14	0.038	56.352
rs72951729	A	C	-0.018	0.003	1.08E-08	0.096	32.742
rs34332006	T	C	-0.011	0.002	1.52E-08	0.390	32.089

rs10164769	T	C	0.027	0.002	7.73E-38	0.743	165.556
rs62194505	A	G	-0.011	0.002	2.00E-08	0.382	31.532
rs1509396	C	G	0.012	0.002	1.64E-10	0.511	40.913
rs935655	G	T	0.015	0.002	8.11E-12	0.762	46.797
rs796056	C	A	0.021	0.002	2.31E-27	0.623	117.536
rs8176528	A	G	-0.017	0.002	6.31E-17	0.297	69.971
rs12997100	C	T	0.027	0.004	9.38E-14	0.071	55.562
rs10203838	C	T	0.012	0.002	4.66E-11	0.418	43.393
rs17026212	G	T	-0.015	0.002	1.06E-15	0.514	64.410
rs34562738	A	G	0.019	0.002	1.99E-17	0.772	72.230
rs11688303	T	C	0.021	0.003	3.19E-13	0.120	53.151
rs79716587	A	G	-0.036	0.003	9.22E-37	0.124	160.590
rs1371045	C	T	-0.014	0.002	1.26E-11	0.744	45.937
rs7573465	T	G	0.036	0.002	4.66E-83	0.554	373.178
rs1047891	A	C	-0.021	0.002	1.60E-26	0.315	113.731
rs1250215	C	G	0.012	0.002	1.08E-09	0.641	37.237
rs6755895	C	T	-0.023	0.002	2.14E-25	0.232	108.545
rs139369470	C	T	0.025	0.004	8.34E-09	0.048	33.248
rs633323	T	A	0.015	0.002	2.87E-13	0.698	53.398
rs75475627	G	C	0.033	0.004	1.92E-20	0.077	86.015
rs12999905	A	G	0.025	0.004	5.58E-10	0.062	38.521
rs6746368	T	A	0.011	0.002	4.36E-09	0.334	34.519
rs2068330	G	C	0.019	0.002	2.87E-22	0.361	94.312
rs527393	A	G	-0.022	0.002	4.34E-33	0.468	143.817
rs17270882	C	G	0.031	0.002	4.07E-46	0.239	203.491
rs10205474	A	C	-0.013	0.002	2.65E-08	0.201	31.000
rs4672564	C	A	-0.010	0.002	3.67E-08	0.501	30.375
rs114050631	T	C	-0.138	0.010	1.38E-45	0.011	201.025
rs2943650	T	C	0.011	0.002	6.36E-09	0.645	33.785
rs1822534	G	A	-0.026	0.002	8.29E-43	0.394	188.252
rs35592432	C	G	0.040	0.006	5.47E-11	0.027	43.071
rs12630592	T	G	-0.012	0.002	6.27E-10	0.373	38.301
rs112880875	A	G	0.026	0.003	9.68E-16	0.090	64.600
rs10936588	A	G	-0.014	0.002	6.95E-14	0.636	56.183
rs9867398	T	C	0.022	0.003	2.80E-12	0.094	48.890
rs1621453	G	A	0.010	0.002	1.71E-08	0.563	31.862
rs1366045	C	T	-0.025	0.002	5.44E-39	0.384	170.879
rs6445826	C	T	0.012	0.002	4.03E-11	0.498	43.670
rs115433278	A	G	-0.024	0.004	1.09E-08	0.053	32.715
rs7639292	T	C	-0.024	0.002	2.48E-22	0.168	94.592
rs9835104	A	G	-0.014	0.002	4.35E-08	0.188	30.025

rs2012610	T	G	0.028	0.003	2.99E-23	0.127	98.819
rs11927257	T	C	-0.013	0.002	2.97E-10	0.282	39.766
rs35713889	T	C	0.028	0.005	5.79E-10	0.043	38.457
rs2713573	T	C	0.025	0.002	3.46E-35	0.689	153.315
rs9819371	T	C	-0.044	0.004	5.94E-32	0.065	138.560
rs4074672	T	C	0.018	0.002	3.87E-21	0.370	89.132
rs3773312	A	G	0.018	0.003	3.67E-10	0.134	39.333
rs73028871	T	C	-0.017	0.003	9.30E-10	0.134	37.528
rs2371108	T	G	0.019	0.002	3.11E-24	0.389	103.273
rs3732378	A	G	0.020	0.002	7.70E-17	0.172	69.609
rs62270938	T	C	0.016	0.002	6.59E-11	0.177	42.696
rs10049210	C	T	0.015	0.002	7.69E-15	0.405	60.488
rs2284837	G	A	0.014	0.002	6.76E-10	0.220	38.150
rs9829114	A	G	-0.034	0.002	7.45E-72	0.414	321.734
rs4234465	C	G	-0.026	0.002	6.07E-40	0.423	175.168
rs6779340	G	C	-0.018	0.002	4.51E-19	0.337	79.738
rs832190	T	C	-0.013	0.002	3.88E-12	0.633	48.276
rs830623	C	A	0.014	0.002	3.88E-09	0.170	34.746
rs4504118	C	G	0.013	0.002	6.34E-12	0.559	47.271
rs6440053	A	T	-0.014	0.003	2.76E-08	0.842	30.914
rs6440732	A	C	0.023	0.002	1.35E-21	0.828	91.215
rs140311179	G	C	-0.026	0.004	1.08E-09	0.051	37.237
rs28530750	A	G	0.056	0.005	1.83E-33	0.043	145.497
rs1371794	C	A	-0.063	0.002	1.00E-200	0.622	1109.142
rs73191188	A	G	-0.016	0.002	1.23E-17	0.353	73.188
rs12502008	T	G	0.011	0.002	1.24E-08	0.361	32.485
rs62311395	G	A	-0.025	0.004	5.09E-11	0.062	43.205
rs1506609	G	A	0.021	0.002	1.61E-19	0.799	81.752
rs6831590	T	G	-0.018	0.002	4.54E-22	0.367	93.390
rs987121	T	A	0.015	0.002	4.76E-16	0.606	65.982
rs13132853	G	A	0.020	0.002	1.88E-25	0.355	108.818
rs6554195	T	G	-0.019	0.002	3.90E-26	0.484	111.922
rs113473633	G	A	-0.051	0.006	3.24E-16	0.026	66.744
rs28588746	G	A	-0.010	0.002	3.61E-08	0.378	30.404
rs62345144	C	G	-0.034	0.006	3.58E-08	0.025	30.408
rs35734242	C	T	0.022	0.002	6.30E-29	0.428	124.778
rs11723621	G	A	-0.029	0.002	7.57E-47	0.290	206.821
rs2305980	A	T	0.014	0.002	6.67E-11	0.255	42.664
rs6854855	G	A	0.016	0.002	9.69E-19	0.487	78.201
rs218264	T	A	0.026	0.002	2.52E-33	0.251	144.864
rs2412771	C	T	-0.018	0.002	4.42E-22	0.417	93.430

rs16850073	T	C	0.043	0.002	8.77E-109	0.374	491.398
rs13149209	C	T	-0.015	0.002	4.50E-11	0.218	43.460
rs144317085	T	A	0.050	0.005	3.00E-22	0.034	94.218
rs10006495	G	T	-0.016	0.002	1.35E-15	0.354	63.959
rs11735662	T	C	0.052	0.005	4.74E-24	0.033	102.444
rs79237520	T	C	0.040	0.006	2.13E-10	0.023	40.403
rs11744663	A	G	-0.019	0.002	1.72E-15	0.190	63.484
rs2910580	A	T	-0.015	0.002	2.83E-15	0.659	62.474
rs4391200	G	A	0.023	0.002	7.86E-34	0.619	147.134
rs13180726	A	G	-0.021	0.002	2.71E-18	0.826	76.213
rs987107	A	G	0.020	0.002	2.52E-22	0.261	94.562
rs79692389	C	G	0.026	0.004	9.39E-12	0.061	46.518
rs2082382	A	G	-0.018	0.002	8.67E-22	0.554	92.170
rs4704826	A	C	-0.016	0.002	9.71E-17	0.634	69.157
rs464609	A	G	0.013	0.002	3.00E-12	0.544	48.773
rs10940474	T	C	-0.013	0.002	6.53E-12	0.659	47.232
rs6877725	C	T	-0.021	0.002	2.12E-28	0.445	122.340
rs4703541	A	G	-0.036	0.003	7.45E-38	0.879	165.533
rs257063	T	C	-0.014	0.002	4.91E-11	0.741	43.296
rs6878780	C	T	0.013	0.002	1.02E-12	0.427	50.886
rs11242109	T	G	-0.033	0.002	5.08E-72	0.477	322.301
rs4535497	A	C	-0.013	0.002	2.34E-11	0.570	44.733
rs7705526	A	C	0.029	0.002	8.30E-43	0.327	188.271
rs2432142	A	G	0.013	0.002	1.09E-11	0.434	46.246
rs10515269	G	C	0.014	0.002	1.92E-14	0.484	58.717
rs79272926	A	C	-0.022	0.002	1.04E-23	0.222	100.866
rs9313822	A	G	0.025	0.004	9.35E-10	0.052	37.518
rs9885207	C	T	0.017	0.002	3.94E-19	0.416	80.003
rs2561758	G	A	-0.036	0.002	4.20E-68	0.723	304.401
rs6458354	T	C	0.014	0.002	8.36E-13	0.708	51.257
rs73738524	G	A	0.023	0.003	1.43E-11	0.080	45.700
rs74679834	G	C	0.013	0.002	3.49E-09	0.277	34.936
rs10872223	T	C	0.014	0.002	3.57E-09	0.183	34.901
rs12214269	A	G	0.018	0.002	5.06E-23	0.546	97.783
rs9390461	G	A	0.015	0.002	8.51E-17	0.538	69.363
rs2394990	A	C	-0.020	0.002	7.10E-27	0.633	115.369
rs507778	T	C	-0.053	0.002	1.41E-159	0.333	725.120
rs1383265	C	T	0.019	0.002	1.68E-15	0.179	63.516
rs9375447	G	A	-0.014	0.002	6.34E-14	0.495	56.348
rs6924387	G	A	0.017	0.002	1.48E-18	0.412	77.385
rs7751717	A	G	-0.014	0.002	2.23E-09	0.200	35.823

rs2935104	G	A	0.020	0.003	3.62E-09	0.907	34.864
rs4712614	G	T	-0.022	0.002	2.79E-30	0.620	130.921
rs2523468	G	C	-0.029	0.002	4.00E-52	0.515	230.972
rs11965885	T	G	-0.015	0.002	5.41E-16	0.582	65.737
rs9382100	A	T	-0.012	0.002	1.38E-09	0.325	36.764
rs4707609	C	T	-0.021	0.002	3.21E-28	0.360	121.437
rs1285886	A	G	0.026	0.002	5.10E-30	0.197	129.779
rs560194	C	T	-0.014	0.002	1.76E-13	0.530	54.341
rs1322599	T	C	-0.031	0.002	9.97E-36	0.174	155.873
rs72843590	A	C	0.025	0.004	1.25E-10	0.063	41.455
rs1611236	A	G	-0.036	0.002	7.26E-74	0.323	330.921
rs2853951	T	C	0.077	0.002	1.00E-200	0.600	1691.314
rs9348970	C	G	-0.033	0.002	3.09E-42	0.178	185.691
rs3857488	G	A	-0.016	0.002	5.95E-18	0.528	74.686
rs364663	A	T	-0.010	0.002	4.41E-08	0.559	30.007
rs549302	A	G	0.020	0.002	8.48E-28	0.545	119.548
rs1738074	C	T	-0.020	0.002	3.74E-28	0.568	121.237
rs12203592	T	C	0.022	0.002	1.61E-21	0.210	90.886
rs2328667	G	A	0.012	0.002	4.85E-11	0.561	43.322
rs185774696	T	C	-0.029	0.003	2.25E-26	0.273	113.039
rs2012011	A	G	-0.026	0.003	2.15E-21	0.136	90.345
rs9487043	T	C	0.023	0.002	4.35E-36	0.487	157.455
rs7776054	G	A	-0.047	0.002	2.48E-110	0.261	498.256
rs72978754	C	T	-0.028	0.004	2.45E-14	0.066	58.233
rs798555	C	T	0.019	0.002	4.81E-20	0.295	84.150
rs1476081	T	G	0.011	0.002	3.85E-08	0.295	30.255
rs12540307	T	C	-0.039	0.004	4.76E-18	0.049	75.095
rs4729046	C	T	-0.037	0.003	1.01E-25	0.924	110.072
rs182090955	A	G	-0.076	0.010	5.33E-15	0.010	61.228
rs3731332	T	C	-0.121	0.006	5.53E-84	0.023	377.248
rs10808139	A	G	0.018	0.003	6.06E-10	0.119	38.348
rs6952262	G	C	-0.016	0.002	2.19E-12	0.205	49.403
rs17138597	A	G	-0.013	0.002	1.30E-08	0.195	32.382
rs2158799	G	C	0.043	0.002	7.85E-114	0.612	514.246
rs6583435	A	C	0.017	0.002	8.70E-18	0.680	73.874
rs4948097	G	A	-0.018	0.002	3.03E-16	0.758	66.892
rs6948695	C	T	-0.011	0.002	4.70E-09	0.414	34.374
rs13237635	C	T	-0.010	0.002	2.24E-08	0.565	31.312
rs62466318	T	C	-0.021	0.002	9.80E-19	0.202	78.196
rs7781268	A	G	-0.013	0.002	3.66E-09	0.246	34.851
rs9656395	G	A	-0.028	0.003	2.97E-18	0.095	76.009

rs1474419	C	T	0.018	0.002	3.11E-22	0.580	94.129
rs7776857	T	G	-0.019	0.002	1.89E-21	0.658	90.537
rs56388170	T	G	0.059	0.002	2.73E-185	0.295	843.241
rs2710804	C	T	0.021	0.002	1.92E-27	0.376	117.992
rs10260281	C	G	0.013	0.002	5.33E-10	0.237	38.610
rs2282986	C	T	-0.124	0.007	1.80E-73	0.019	329.049
rs74607840	A	G	-0.015	0.002	8.82E-16	0.626	64.780
rs4721650	G	A	0.015	0.002	3.76E-11	0.210	43.804
rs60466842	A	C	-0.030	0.003	1.04E-19	0.088	82.632
rs3735485	G	A	0.041	0.003	2.34E-57	0.845	254.954
rs2692540	G	C	-0.011	0.002	1.19E-09	0.428	37.058
rs342294	C	T	-0.017	0.002	4.90E-21	0.456	88.724
rs38859	T	C	0.011	0.002	9.71E-10	0.449	37.453
rs7803075	G	A	-0.024	0.002	5.47E-31	0.734	134.164
rs12716647	C	G	0.012	0.002	1.12E-10	0.638	41.656
rs755951	C	A	-0.013	0.002	1.02E-12	0.403	50.879
rs6474514	C	T	0.011	0.002	3.59E-09	0.412	34.874
rs4276676	C	T	-0.019	0.002	2.22E-25	0.469	108.547
rs1947897	C	G	-0.014	0.002	1.71E-13	0.336	54.405
rs10102877	A	G	-0.013	0.002	3.91E-11	0.685	43.744
rs921313	A	G	0.027	0.004	1.32E-09	0.045	36.849
rs6985508	A	G	-0.027	0.002	1.57E-39	0.358	173.200
rs1511724	G	C	-0.017	0.002	1.15E-18	0.422	77.888
rs17209630	A	G	0.014	0.002	3.25E-12	0.281	48.617
rs10087240	T	C	0.020	0.002	6.13E-27	0.458	115.675
rs12550612	A	G	-0.033	0.002	1.09E-41	0.821	183.154
rs28571765	C	T	-0.022	0.002	1.32E-20	0.207	86.717
rs7846314	T	A	0.047	0.002	5.71E-88	0.187	395.443
rs11993347	C	T	-0.020	0.002	2.03E-19	0.230	81.296
rs4870937	T	A	0.014	0.002	9.50E-15	0.544	60.081
rs55964818	C	T	-0.036	0.002	1.54E-84	0.567	379.913
rs145209947	A	C	-0.075	0.009	3.19E-18	0.014	75.875
rs7005996	T	C	0.024	0.003	4.06E-13	0.906	52.704
rs11250076	G	A	0.021	0.002	2.44E-30	0.575	131.125
rs13248936	G	A	0.013	0.002	1.17E-12	0.498	50.587
rs2875974	A	G	0.021	0.002	3.54E-29	0.373	125.913
rs10808536	A	G	0.012	0.002	4.56E-09	0.295	34.418
rs4734879	G	A	-0.022	0.002	1.34E-26	0.276	114.116
rs12216862	T	C	0.011	0.002	1.81E-08	0.290	31.746
rs62510269	G	A	-0.036	0.003	1.77E-43	0.146	191.369
rs3847147	A	G	-0.012	0.002	2.99E-09	0.307	35.243

rs13291664	G	A	0.026	0.002	1.57E-28	0.187	122.897
rs6476883	A	G	-0.011	0.002	6.27E-10	0.437	38.297
rs1887428	C	G	-0.015	0.002	9.58E-15	0.625	60.050
rs626416	C	G	-0.017	0.002	2.81E-14	0.763	57.965
rs7855091	A	G	0.015	0.002	1.11E-13	0.307	55.261
rs7852409	G	C	0.016	0.002	8.71E-12	0.765	46.654
rs12376511	C	T	-0.034	0.002	6.16E-43	0.164	188.822
rs10973700	C	G	-0.013	0.002	1.36E-12	0.486	50.338
rs4877107	C	T	0.010	0.002	2.45E-08	0.457	31.151
rs12683699	G	A	0.022	0.003	5.16E-18	0.160	74.918
rs7036656	T	C	0.028	0.002	1.25E-42	0.722	187.523
rs10814191	T	C	0.011	0.002	3.84E-08	0.713	30.288
rs3793537	C	G	0.020	0.002	1.53E-22	0.290	95.555
rs72747074	A	G	0.018	0.002	3.63E-19	0.312	80.148
rs7864482	G	A	0.013	0.002	2.00E-11	0.377	45.049
rs10980797	G	A	-0.027	0.002	1.19E-48	0.488	215.020
rs2519093	T	C	-0.041	0.002	8.55E-68	0.186	302.821
rs796003	T	G	-0.025	0.002	8.84E-33	0.258	142.333
rs12343532	A	T	-0.015	0.002	2.49E-13	0.272	53.655
rs2506699	G	A	0.014	0.002	4.87E-14	0.484	56.886
rs4413892	A	G	0.031	0.002	2.87E-46	0.276	204.066
rs447124	T	C	0.017	0.002	4.86E-20	0.491	84.118
rs2148537	A	G	0.018	0.002	1.31E-12	0.166	50.378
rs7850247	C	T	0.010	0.002	2.84E-08	0.515	30.871
rs77215665	G	A	-0.040	0.007	5.13E-09	0.018	34.194
rs10986338	A	G	-0.017	0.002	1.93E-17	0.650	72.350
rs41317014	T	C	-0.020	0.002	3.25E-19	0.248	80.390
rs2993986	T	C	0.030	0.002	4.75E-43	0.774	189.483
rs2091084	C	T	0.017	0.002	6.25E-19	0.653	79.083
rs180941	A	G	-0.017	0.002	1.63E-18	0.625	77.245
rs41295055	T	C	-0.014	0.002	4.95E-09	0.187	34.259
rs3781454	A	G	0.025	0.002	3.68E-36	0.677	157.792
rs10828725	T	G	-0.050	0.002	1.10E-148	0.365	674.966
rs72790862	C	T	-0.026	0.002	5.05E-38	0.307	166.336
rs6479830	C	T	-0.012	0.002	1.05E-09	0.715	37.286
rs7917772	A	G	0.017	0.002	3.60E-18	0.631	75.628
rs72836628	T	C	-0.014	0.002	3.02E-11	0.249	44.215
rs11198788	C	T	0.012	0.002	1.47E-10	0.431	41.112
rs11252331	G	T	0.017	0.002	1.80E-11	0.837	45.246
rs10906393	T	A	0.015	0.002	2.44E-16	0.576	67.316
rs692594	C	G	0.013	0.002	1.70E-12	0.489	49.905

rs703005	T	C	-0.015	0.002	4.55E-16	0.598	66.048
rs3747869	C	A	0.039	0.003	1.79E-33	0.900	145.509
rs61863767	T	C	0.043	0.002	4.56E-117	0.596	529.342
rs12784071	T	C	0.018	0.002	4.79E-16	0.222	66.000
rs17011726	G	C	-0.024	0.002	3.58E-27	0.233	116.713
rs7082470	A	G	-0.029	0.002	5.75E-56	0.474	248.695
rs1885474	G	T	-0.022	0.003	1.11E-12	0.103	50.705
rs11002309	T	C	0.013	0.002	8.28E-11	0.340	42.263
rs10749539	G	C	-0.012	0.002	1.23E-09	0.293	36.991
rs59085061	G	A	-0.050	0.005	9.00E-27	0.040	114.889
rs9633675	C	G	-0.019	0.002	9.60E-25	0.479	105.626
rs6421984	C	T	0.039	0.002	2.79E-87	0.515	392.371
rs1468102	G	C	0.015	0.002	9.95E-14	0.306	55.465
rs10769968	C	G	-0.011	0.002	1.73E-08	0.456	31.833
rs11022177	G	C	-0.018	0.002	3.43E-19	0.311	80.278
rs1228024	A	C	0.025	0.002	2.32E-38	0.661	167.918
rs174548	G	C	-0.025	0.002	6.38E-36	0.314	156.721
rs617791	C	G	0.019	0.002	1.59E-24	0.483	104.609
rs7177	A	C	-0.013	0.002	1.43E-12	0.529	50.193
rs148713124	T	G	0.096	0.010	1.35E-21	0.009	91.257
rs4909945	C	T	0.011	0.002	8.23E-09	0.689	33.274
rs73000965	A	T	0.029	0.002	1.86E-47	0.315	209.615
rs672058	T	C	0.018	0.003	1.81E-10	0.880	40.729
rs11221394	C	T	0.016	0.003	1.54E-08	0.118	32.052
rs4909932	G	A	0.014	0.002	2.01E-13	0.598	54.051
rs12792460	G	C	0.016	0.002	4.13E-14	0.271	57.188
rs1232050	C	G	-0.012	0.002	2.48E-09	0.430	35.605
rs934177	G	C	-0.011	0.002	1.48E-09	0.436	36.622
rs8705	A	G	-0.026	0.002	1.35E-39	0.317	173.505
rs579721	A	G	-0.012	0.002	1.55E-09	0.284	36.527
rs113519804	A	C	-0.032	0.006	9.92E-09	0.029	32.904
rs6590806	A	G	0.016	0.002	4.28E-14	0.250	57.099
rs7934719	T	C	0.018	0.002	1.55E-21	0.414	90.946
rs59241311	A	G	-0.017	0.003	2.24E-08	0.100	31.316
rs873218	A	G	-0.012	0.002	1.34E-10	0.554	41.319
rs11104881	C	T	-0.025	0.002	1.59E-34	0.702	150.387
rs73190675	A	G	0.029	0.004	1.15E-11	0.050	46.122
rs9863	C	T	-0.019	0.002	1.53E-21	0.329	91.039
rs10844682	C	T	0.025	0.002	1.43E-38	0.342	168.813
rs739842	C	T	-0.016	0.002	3.44E-15	0.639	62.084
rs11169302	G	T	0.019	0.002	9.45E-23	0.392	96.567

rs9330650	G	A	0.014	0.002	1.77E-10	0.219	40.759
rs632887	G	A	0.012	0.002	3.75E-10	0.411	39.282
rs67684915	G	C	-0.023	0.002	9.67E-22	0.195	91.905
rs2286599	A	G	0.039	0.003	1.43E-43	0.141	191.807
rs11048425	G	C	0.013	0.002	1.03E-12	0.491	50.888
rs1895994	A	G	-0.013	0.002	4.35E-11	0.324	43.510
rs4761234	C	T	0.015	0.002	2.03E-16	0.482	67.645
rs3184504	C	T	-0.067	0.002	1.00E-200	0.517	1339.292
rs7308123	C	T	0.019	0.002	2.29E-14	0.181	58.344
rs706809	C	T	0.022	0.002	4.71E-23	0.778	97.894
rs759488	C	T	0.014	0.002	2.58E-11	0.705	44.523
rs76545872	A	G	-0.013	0.002	2.50E-08	0.194	31.112
rs1007938	G	A	-0.011	0.002	3.26E-09	0.405	35.063
rs1245035	A	C	0.014	0.002	4.79E-14	0.627	56.879
rs1042725	T	C	0.011	0.002	3.71E-09	0.495	34.821
rs11114149	T	A	-0.014	0.002	7.30E-12	0.683	47.023
rs17041439	C	A	0.038	0.004	2.96E-21	0.057	89.706
rs11064881	A	G	-0.032	0.004	9.92E-20	0.074	82.750
rs76428106	C	T	0.158	0.009	3.45E-77	0.013	346.085
rs2260850	G	T	0.024	0.002	1.68E-29	0.281	127.414
rs138028125	G	C	0.054	0.006	2.54E-22	0.034	94.543
rs150861794	T	C	-0.065	0.007	4.31E-20	0.020	84.401
rs9590390	A	G	0.015	0.002	7.32E-14	0.291	56.057
rs9508005	G	T	-0.023	0.003	4.92E-11	0.094	43.275
rs806321	T	C	0.016	0.002	5.66E-18	0.530	74.774
rs12429714	T	C	-0.011	0.002	2.45E-09	0.424	35.647
rs55978995	A	C	0.014	0.002	1.90E-08	0.169	31.630
rs3812849	C	A	0.020	0.002	5.85E-21	0.267	88.374
rs7326825	A	G	0.021	0.002	1.73E-24	0.704	104.492
rs9526795	C	T	-0.014	0.002	2.68E-11	0.259	44.465
rs2439963	G	A	-0.012	0.002	3.29E-09	0.737	35.054
rs2038700	C	T	0.034	0.002	4.61E-74	0.395	331.740
rs175714	C	T	0.010	0.002	2.64E-08	0.577	31.002
rs9806027	C	G	-0.016	0.002	3.73E-18	0.554	75.557
rs2180369	C	T	0.023	0.003	7.65E-14	0.111	55.994
rs696	T	C	0.015	0.002	4.53E-15	0.366	61.571
rs11159261	C	T	-0.014	0.002	8.68E-15	0.534	60.270
rs9323285	A	C	0.012	0.002	8.19E-10	0.338	37.764
rs10138752	T	C	-0.040	0.003	1.85E-31	0.077	136.340
rs10498635	T	C	-0.031	0.002	6.58E-39	0.183	170.363
rs7159281	G	A	-0.013	0.002	1.35E-10	0.672	41.280

rs72664840	T	C	0.021	0.002	8.44E-18	0.179	73.958
rs10146962	C	T	-0.015	0.002	6.45E-15	0.338	60.848
rs12898000	C	G	0.019	0.002	7.85E-20	0.701	83.166
rs62007171	C	T	-0.015	0.002	6.22E-14	0.291	56.366
rs12905862	A	G	0.011	0.002	2.15E-08	0.316	31.409
rs7183988	G	T	-0.016	0.002	8.74E-17	0.527	69.315
rs4924450	A	G	0.014	0.002	1.14E-10	0.706	41.621
rs60695341	T	C	-0.030	0.002	3.20E-38	0.197	167.261
rs780142	G	T	0.015	0.002	2.02E-12	0.274	49.543
rs28576226	A	G	0.031	0.003	6.14E-28	0.122	120.245
rs2062250	A	G	0.051	0.004	2.12E-38	0.938	168.069
rs2469147	G	A	-0.015	0.003	1.67E-09	0.158	36.375
rs62019353	C	T	0.012	0.002	1.54E-08	0.273	32.044
rs72726027	C	T	-0.043	0.003	4.17E-48	0.112	212.602
rs11854390	T	C	0.014	0.002	2.13E-13	0.562	53.975
rs9926183	T	C	0.019	0.002	2.86E-17	0.263	71.513
rs11574938	C	G	0.038	0.002	8.13E-91	0.521	408.802
rs117556162	A	G	0.027	0.004	1.99E-11	0.057	45.050
rs9933582	G	T	0.021	0.002	9.18E-22	0.230	91.973
rs75859969	C	A	-0.017	0.003	5.94E-11	0.156	42.908
rs4984768	T	G	0.012	0.002	3.34E-09	0.363	35.040
rs9925985	C	A	-0.014	0.002	1.09E-11	0.269	46.244
rs11644125	T	C	-0.018	0.002	6.36E-21	0.597	88.199
rs12918121	T	C	-0.051	0.007	1.08E-12	0.018	50.777
rs7198940	T	C	0.016	0.002	3.33E-17	0.522	71.202
rs9938104	T	C	-0.017	0.003	3.25E-09	0.126	35.074
rs12929950	A	G	-0.030	0.004	7.83E-17	0.077	69.572
rs6500550	T	C	-0.021	0.002	2.65E-24	0.304	103.567
rs61739285	T	C	-0.034	0.005	9.98E-11	0.033	41.885
rs112282032	T	C	-0.018	0.002	9.93E-18	0.271	73.646
rs247826	T	C	0.034	0.002	9.59E-52	0.221	229.319
rs17232826	T	C	-0.022	0.003	1.60E-12	0.102	50.012
rs2259855	C	T	-0.024	0.002	2.66E-37	0.525	163.076
rs9900613	T	C	-0.011	0.002	1.30E-09	0.431	36.879
rs2314339	T	C	0.027	0.003	1.52E-21	0.125	90.981
rs56378716	G	A	0.108	0.008	1.70E-38	0.013	168.523
rs2665405	A	G	0.035	0.002	5.63E-82	0.548	367.977
rs2282677	C	T	0.011	0.002	3.79E-09	0.405	34.803
rs4789229	T	C	0.012	0.002	1.18E-09	0.645	37.067
rs1109278	G	A	-0.014	0.002	9.32E-14	0.484	55.584
rs1285254	C	T	-0.012	0.002	4.70E-08	0.775	29.870

rs6502608	A	G	0.015	0.002	1.50E-12	0.707	50.118
rs7225843	C	T	-0.032	0.002	8.58E-43	0.203	188.180
rs35592645	A	C	0.017	0.002	2.38E-16	0.249	67.340
rs7502100	C	T	0.018	0.002	8.40E-20	0.367	83.047
rs1024091	T	C	0.014	0.002	8.71E-14	0.558	55.740
rs12936529	T	C	-0.025	0.002	2.74E-42	0.479	185.919
rs16964983	C	T	0.022	0.002	2.89E-19	0.173	80.630
rs2428608	T	C	-0.011	0.002	1.05E-09	0.563	37.288
rs35111447	T	C	-0.018	0.003	8.56E-09	0.098	33.186
rs62089697	A	G	0.018	0.003	2.48E-12	0.172	49.144
rs4790752	T	C	-0.018	0.002	2.11E-21	0.451	90.368
rs146207734	T	A	-0.054	0.008	4.58E-11	0.017	43.415
rs55767800	C	T	-0.018	0.002	4.71E-20	0.632	84.185
rs17660636	G	A	0.011	0.002	4.67E-09	0.358	34.363
rs4794820	G	A	0.072	0.002	1.00E-200	0.552	1501.817
rs4239149	G	A	0.016	0.002	5.58E-17	0.656	70.249
rs2084312	T	C	0.029	0.002	7.67E-36	0.800	156.320
rs9747839	G	C	0.019	0.002	4.46E-18	0.476	75.253
rs11874453	A	G	-0.012	0.002	1.36E-09	0.533	36.805
rs67491878	C	A	0.011	0.002	6.15E-09	0.604	33.821
rs78285907	T	A	0.020	0.003	3.10E-09	0.089	35.164
rs75354229	T	C	0.024	0.003	6.22E-19	0.129	79.106
rs718515	A	G	-0.016	0.002	1.20E-18	0.552	77.825
rs4468717	T	C	-0.020	0.003	8.41E-09	0.078	33.227
rs303753	A	G	-0.020	0.002	1.78E-23	0.345	99.867
rs8084255	T	C	0.020	0.002	1.26E-25	0.376	109.639
rs12956324	A	C	0.014	0.002	2.10E-13	0.403	53.981
rs7235882	T	C	0.018	0.003	2.60E-08	0.094	31.028
rs9304460	A	G	0.012	0.002	4.14E-10	0.444	39.111
rs72982988	A	G	-0.013	0.002	7.10E-09	0.233	33.570
rs17758695	T	C	-0.056	0.006	1.67E-22	0.029	95.396
rs72973711	T	A	-0.032	0.004	1.28E-17	0.067	73.125
rs11669443	A	G	0.014	0.002	9.50E-10	0.237	37.497
rs73016721	T	A	0.016	0.002	6.40E-14	0.278	56.328
rs2290669	C	A	-0.048	0.002	1.52E-94	0.786	425.630
rs76378167	A	G	0.023	0.004	1.07E-08	0.061	32.754
rs4805881	C	A	-0.016	0.002	1.32E-16	0.665	68.495
rs2194067	T	C	0.013	0.002	2.31E-11	0.416	44.761
rs4760	G	A	-0.070	0.003	2.21E-161	0.153	733.083
rs71352239	T	C	-0.013	0.002	3.36E-09	0.324	35.006
rs571497	A	G	-0.036	0.003	6.32E-45	0.155	197.965

rs2358581	G	T	-0.028	0.002	9.36E-38	0.733	165.185
rs430989	G	T	-0.014	0.002	5.67E-11	0.269	42.992
rs35112940	A	G	-0.026	0.002	5.21E-31	0.216	134.274
rs73036517	G	A	-0.053	0.002	1.08E-137	0.259	624.453
rs309190	C	T	-0.030	0.003	7.05E-22	0.894	92.510
rs7246841	C	T	0.022	0.002	3.78E-24	0.731	102.881
rs6055955	T	C	-0.022	0.002	2.63E-33	0.509	144.807
rs6062468	T	C	0.011	0.002	2.46E-08	0.508	31.139
rs2327028	T	C	-0.013	0.002	3.36E-13	0.476	53.067
rs6029234	C	G	0.024	0.002	7.57E-37	0.624	160.963
rs6051431	T	C	0.012	0.002	8.44E-10	0.351	37.710
rs34952318	A	G	-0.033	0.004	4.24E-14	0.050	57.151
rs1891033	C	T	0.015	0.002	1.44E-09	0.169	36.665
rs3752562	A	G	0.014	0.002	8.97E-10	0.775	37.609
rs2904270	A	G	-0.015	0.002	1.03E-16	0.517	69.005
rs2427599	T	C	0.056	0.010	5.21E-09	0.011	34.158
rs6045615	C	A	-0.019	0.002	1.99E-22	0.330	95.008
rs990558	T	C	-0.011	0.002	1.00E-08	0.361	32.885
rs9977672	A	G	-0.022	0.002	7.08E-25	0.259	106.239
rs7283930	T	C	-0.012	0.002	2.57E-08	0.739	31.056
rs28574812	G	A	-0.016	0.003	2.66E-10	0.158	39.978
rs35990176	C	A	0.012	0.002	6.46E-10	0.451	38.233
rs2298695	C	A	-0.016	0.002	6.51E-17	0.396	69.941
rs5746990	A	G	-0.015	0.002	2.05E-15	0.623	63.103
rs399757	C	T	0.014	0.002	1.16E-11	0.383	46.103
rs2857633	C	T	-0.013	0.002	2.52E-10	0.727	40.085
rs1033415	G	A	0.013	0.002	6.01E-10	0.650	38.384
rs2076211	T	C	-0.018	0.003	1.53E-12	0.158	50.084
rs5746451	C	T	0.020	0.002	4.61E-26	0.504	111.634
rs34505104	G	A	-0.020	0.002	3.36E-23	0.305	98.546
rs47341	T	C	0.016	0.002	1.01E-15	0.396	64.527
rs9625746	C	G	-0.018	0.002	8.77E-22	0.412	92.090
rs12157427	A	G	-0.021	0.004	1.56E-08	0.064	32.020
rs713909	C	G	-0.020	0.002	2.75E-26	0.432	112.668

β represents the change in a potential mediator (in standard deviation unit, except for CRP, whose unit was one-unit natural-log-transformed CRP) per additional copy of the effect allele.

Abbreviations: SNPs, single nucleotide polymorphisms; SE, standard error; EAF, effect allele frequency; FEV1, forced expiratory volume in the first second; FVC, forced vital capacity; CRP, C-reactive protein; GlycA, glycoprotein acetyls; IL-1Ra, interleukin-1-receptor antagonist; IL-6, interleukin-6; IL-8, interleukin-8; IL-18, interleukin-18; IL-27, interleukin-27.

Table S4. Effects of obesity-related traits on lower respiratory tract infections estimated using different methods.

Obesity-related traits (data sources)	Lower respiratory tract infections	Methods	No. of SNPs	$\hat{\beta}$	SE	OR (95% CI)	*P*
BMI (GIANT and UKB[b])	Acute bronchitis	Inverse variance weighted[a]	494	0.259	0.040	1.30 (1.20, 1.40)	5.36E-11
		MR Egger	494	0.116	0.106	1.12 (0.91, 1.38)	2.74E-01
		Weighted median	494	0.227	0.061	1.26 (1.11, 1.41)	1.78E-04
		Weighted mode	494	0.044	0.139	1.04 (0.80, 1.37)	7.53E-01
	Acute bronchiolitis	Inverse variance weighted	494	0.046	0.100	1.05 (0.86, 1.27)	6.45E-01
		MR Egger	494	0.240	0.269	1.27 (0.75, 2.16)	3.73E-01
		Weighted median	494	0.074	0.168	1.08 (0.77, 1.50)	6.61E-01
		Weighted mode	494	0.202	0.318	1.22 (0.66, 2.28)	5.25E-01
	Bronchiectasis	Inverse variance weighted[a]	494	-0.342	0.099	0.71 (0.58, 0.86)	5.77E-04
		MR Egger	494	-0.460	0.267	0.63 (0.37, 1.07)	8.57E-02
		Weighted median	494	-0.449	0.162	0.64 (0.47, 0.88)	5.51E-03
		Weighted mode	494	-0.647	0.297	0.52 (0.29, 0.94)	3.00E-02
	Influenza	Inverse variance weighted[a]	494	0.240	0.054	1.27 (1.14, 1.41)	8.39E-06
		MR Egger	494	0.132	0.145	1.14 (0.86, 1.52)	3.63E-01
		Weighted median	494	0.160	0.082	1.17 (1.00, 1.38)	5.23E-02
		Weighted mode	494	0.160	0.133	1.17 (0.90, 1.52)	2.29E-01
	Pneumonia	Inverse variance weighted[a]	494	0.179	0.023	1.20 (1.14, 1.25)	1.02E-14
		MR Egger	494	0.145	0.062	1.16 (1.02, 1.31)	2.00E-02
		Weighted median	494	0.170	0.034	1.19 (1.11, 1.27)	4.40E-07
		Weighted mode	494	0.130	0.074	1.14 (0.99, 1.32)	7.92E-02
WC (UKB)	Acute bronchitis	Inverse variance weighted[a]	353	0.234	0.050	1.26 (1.15, 1.39)	2.64E-06
		MR Egger	353	0.111	0.143	1.12 (0.85, 1.48)	4.37E-01
		Weighted median	353	0.183	0.071	1.20 (1.05, 1.38)	9.74E-03
		Weighted mode	353	-0.053	0.164	0.95 (0.69, 1.31)	7.46E-01
	Acute bronchiolitis	Inverse variance weighted	353	0.227	0.123	1.25 (0.99, 1.60)	6.56E-02
		MR Egger	353	0.242	0.351	1.27 (0.64, 2.53)	4.92E-01
		Weighted median	353	0.164	0.221	1.18 (0.76, 1.82)	4.59E-01
		Weighted mode	353	0.390	0.405	1.48 (0.67, 3.27)	3.36E-01
	Bronchiectasis	Inverse variance weighted[a]	353	-0.140	0.125	0.87 (0.68, 1.11)	2.62E-01
		MR Egger	353	-0.815	0.354	0.44 (0.22, 0.89)	2.21E-02
		Weighted median	353	-0.309	0.191	0.73 (0.50, 1.07)	1.06E-01
		Weighted mode	353	-0.652	0.388	0.52 (0.24, 1.12)	9.41E-02
	Influenza	Inverse variance weighted[a]	353	0.181	0.063	1.20 (1.06, 1.36)	4.06E-03
		MR Egger	353	0.253	0.180	1.29 (0.90, 1.83)	1.62E-01
		Weighted median	353	0.201	0.106	1.22 (0.99, 1.51)	5.83E-02
		Weighted mode	353	0.235	0.184	1.27 (0.88, 1.82)	2.02E-01
	Pneumonia	Inverse variance weighted[a]	353	0.210	0.028	1.23 (1.17, 1.30)	6.72E-14
		MR Egger	353	0.166	0.080	1.18 (1.01, 1.38)	3.95E-02
		Weighted median	353	0.176	0.039	1.19 (1.10, 1.29)	6.10E-06

		Weighted mode	353	0.020	0.097	1.02 (0.84, 1.23)	8.38E-01
WHR (GIANT and UKB[b])	Acute bronchitis	Inverse variance weighted[a]	334	0.164	0.049	1.18 (1.07, 1.30)	8.11E-04
		MR Egger	334	-0.213	0.130	0.81 (0.63, 1.04)	1.02E-01
		Weighted median	334	0.065	0.069	1.07 (0.93, 1.22)	3.41E-01
		Weighted mode	334	-0.196	0.139	0.82 (0.63, 1.08)	1.58E-01
	Acute bronchiolitis	Inverse variance weighted	334	0.063	0.121	1.07 (0.84, 1.35)	6.02E-01
		MR Egger	334	-0.093	0.324	0.91 (0.48, 1.72)	7.74E-01
		Weighted median	334	-0.197	0.220	0.82 (0.53, 1.26)	3.70E-01
		Weighted mode	334	-0.178	0.318	0.84 (0.45, 1.56)	5.75E-01
	Bronchiectasis	Inverse variance weighted[a]	334	-0.197	0.123	0.82 (0.64, 1.04)	1.09E-01
		MR Egger	334	-0.050	0.331	0.95 (0.50, 1.82)	8.79E-01
		Weighted median	334	-0.249	0.188	0.78 (0.54, 1.13)	1.87E-01
		Weighted mode	334	-0.295	0.362	0.74 (0.37, 1.51)	4.16E-01
	Influenza	Inverse variance weighted	334	0.129	0.058	1.14 (1.02, 1.27)	2.57E-02
		MR Egger	334	-0.069	0.158	0.93 (0.68, 1.27)	6.65E-01
		Weighted median	334	0.102	0.092	1.11 (0.93, 1.33)	2.64E-01
		Weighted mode	334	0.162	0.223	1.18 (0.76, 1.82)	4.68E-01
	Pneumonia	Inverse variance weighted[a]	334	0.079	0.029	1.08 (1.02, 1.15)	7.22E-03
		MR Egger	334	-0.131	0.078	0.88 (0.75, 1.02)	9.42E-02
		Weighted median	334	-0.054	0.042	0.95 (0.87, 1.03)	2.00E-01
		Weighted mode	334	-0.100	0.067	0.90 (0.79, 1.03)	1.36E-01

$\hat{\beta}$ means the change in the log-odds of a type of LRTIs associated with per standard deviation increase in an obesity-related trait.

[a] denotes the use of a random-effects inverse variance weighted model.

[b] The data were originated from a meta-analysis combining the GWAS data provided by GIANT and UKB.

Abbreviations: SE, standard error; OR, odds ratio; CI, confidence interval; BMI, body mass index; WC, waist circumference; WHR, waist-to-hip ratio; GIANT, Genetic Investigation of ANthropometric Traits; UKB, UK Biobank.

Table S5. Results of sensitivity analyses for the effects of obesity-related traits on lower respiratory tract infections.

Obesity-related traits (data sources)	Lower respiratory tract infections	Pleiotropy			Heterogeneity			MR-PRESSO					
		Egger intercept	SE	*P*	*Q*	*dfs*	*P*	$\hat{\beta}$	SE	*P*	$\hat{\beta}$ (Outlier SNPs Removed)	SE (Outlier SNPs Removed)	*P* (Outlier SNPs Removed)
BMI (GIANT and UKB[a])	Acute bronchitis	0.002	0.002	0.147	599.387	493	7.07E-04	0.262	0.039	4.17E-11	0.249	0.038	1.35E-10
	Acute bronchiolitis	-0.003	0.004	0.439	488.849	493	5.44E-01	0.045	0.098	6.46E-01	NA	NA	NA
	Bronchiectasis	0.002	0.004	0.633	553.992	493	2.95E-02	-0.335	0.098	6.50E-04	NA	NA	NA
	Influenza	0.002	0.002	0.422	623.590	493	5.66E-05	0.231	0.053	1.40E-05	0.215	0.052	3.75E-05
	Pneumonia	0.001	0.001	0.560	658.092	493	8.56E-07	0.172	0.023	2.01E-13	NA	NA	NA
WC (UKB)	Acute bronchitis	0.002	0.002	0.356	452.857	352	2.21E-04	0.240	0.048	9.52E-07	0.235	0.047	8.63E-07
	Acute bronchiolitis	0.000	0.005	0.964	343.153	352	6.22E-01	0.150	0.121	2.17E-01	NA	NA	NA
	Bronchiectasis	0.011	0.005	0.043	413.101	352	1.37E-02	-0.112	0.122	3.60E-01	NA	NA	NA
	Influenza	-0.001	0.003	0.672	404.351	352	2.82E-02	0.146	0.063	2.07E-02	NA	NA	NA
	Pneumonia	0.001	0.001	0.554	456.202	352	1.47E-04	0.207	0.028	1.21E-12	NA	NA	NA
WHR (GIANT and UKB[a])	Acute bronchitis	0.007	0.002	0.002	427.310	333	3.59E-04	0.156	0.048	1.13E-03	0.156	0.046	8.21E-04
	Acute bronchiolitis	0.003	0.005	0.603	318.392	333	7.08E-01	0.096	0.117	4.14E-01	NA	NA	NA
	Bronchiectasis	-0.003	0.005	0.632	393.685	333	1.23E-02	-0.174	0.119	1.45E-01	NA	NA	NA
	Influenza	0.003	0.003	0.179	346.310	333	2.96E-01	0.136	0.058	1.88E-02	NA	NA	NA
	Pneumonia	0.004	0.001	0.004	492.595	333	2.82E-08	0.084	0.029	3.70E-03	NA	NA	NA

$\hat{\beta}$ means the change in the log-odds of a type of LRTIs associated with per standard deviation increase in an obesity-related trait.

[a] The data were originated from a meta-analysis combining the GWAS data provided by GIANT and UKB.

Abbreviations: MR-PRESSO, MR-pleiotropy residual sum and outlier method; Egger intercept, intercept term of MR Egger regression; SE, standard error; *dfs*, degrees of freedom; SNPs, single nucleotide polymorphisms; BMI, body mass index; WC, waist circumference; WHR, waist-to-hip ratio; GIANT, Genetic Investigation of ANthropometric Traits; UKB, UK Biobank.

Table S6. Effects of obesity-related traits on potential mediators estimated using different methods.

Obesity-related traits (data sources)	Potential mediators	Methods	No. of SNPs	$\widehat{\beta}$ (95% CI)	SE	*P*
BMI (GIANT)	FEV1	Inverse variance weighted[a]	76	-0.10 (-0.14, -0.05)	0.023	1.21E-05
		MR Egger	76	-0.02 (-0.13, 0.08)	0.054	6.90E-01
		Weighted median	76	-0.08 (-0.12, -0.03)	0.022	3.97E-04
		Weighted mode	76	-0.07 (-0.12, -0.02)	0.024	3.92E-03
BMI (GIANT)	FVC	Inverse variance weighted[a]	77	-0.16 (-0.21, -0.11)	0.025	3.39E-10
		MR Egger	77	-0.11 (-0.23, 0.01)	0.061	7.43E-02
		Weighted median	77	-0.14 (-0.19, -0.10)	0.022	3.39E-11
		Weighted mode	77	-0.16 (-0.20, -0.12)	0.022	5.08E-10
BMI (GIANT and UKB[b])	CRP	Inverse variance weighted[a]	453	0.37 (0.34, 0.40)	0.014	4.30E-145
		MR Egger	453	0.37 (0.29, 0.44)	0.038	3.10E-20
		Weighted median	453	0.40 (0.36, 0.44)	0.021	5.59E-80
		Weighted mode	453	0.42 (0.35, 0.49)	0.036	4.93E-28
BMI (GIANT)	GlycA	Inverse variance weighted[a]	78	0.17 (0.12, 0.22)	0.025	5.69E-12
		MR Egger	78	0.06 (-0.06, 0.17)	0.058	3.46E-01
		Weighted median	78	0.14 (0.08, 0.20)	0.032	5.97E-06
		Weighted mode	78	0.09 (0.01, 0.17)	0.041	2.63E-02
BMI (GIANT and UKB[b])	IL-1Ra	Inverse variance weighted	373	0.17 (0.10, 0.25)	0.037	2.08E-06
		MR Egger	373	0.39 (0.21, 0.57)	0.092	2.56E-05
		Weighted median	373	0.20 (0.08, 0.32)	0.061	8.74E-04
		Weighted mode	373	0.23 (0.01, 0.45)	0.111	4.04E-02
BMI (GIANT and UKB[b])	IL-6	Inverse variance weighted	385	0.19 (0.11, 0.27)	0.042	7.81E-06
		MR Egger	385	0.38 (0.17, 0.59)	0.107	4.30E-04
		Weighted median	385	0.25 (0.12, 0.37)	0.065	1.69E-04
		Weighted mode	385	0.37 (0.10, 0.64)	0.137	7.62E-03
BMI (GIANT and UKB[b])	IL-8	Inverse variance weighted	398	0.05 (-0.03, 0.12)	0.038	2.16E-01
		MR Egger	398	0.02 (-0.17, 0.21)	0.098	8.44E-01
		Weighted median	398	0.09 (-0.04, 0.21)	0.065	1.86E-01
		Weighted mode	398	0.07 (-0.17, 0.31)	0.122	5.46E-01
BMI (GIANT and UKB[b])	IL-18	Inverse variance weighted	390	-0.01 (-0.08, 0.06)	0.036	8.01E-01
		MR Egger	390	-0.04 (-0.22, 0.14)	0.092	6.50E-01
		Weighted median	390	-0.02 (-0.12, 0.09)	0.056	7.86E-01
		Weighted mode	390	0.02 (-0.21, 0.25)	0.116	8.63E-01
BMI (GIANT and UKB[b])	IL-27	Inverse variance weighted	389	0.02 (-0.05, 0.09)	0.036	6.49E-01
		MR Egger	389	-0.02 (-0.21, 0.16)	0.092	7.91E-01
		Weighted median	389	0.02 (-0.10, 0.13)	0.060	7.71E-01
		Weighted mode	389	-0.01 (-0.22, 0.19)	0.103	8.96E-01
BMI (GIANT)	Basophil count	Inverse variance weighted[a]	73	-0.01 (-0.03, 0.02)	0.013	6.39E-01
		MR Egger	73	0.01 (-0.05, 0.07)	0.030	6.83E-01

		Weighted median	73	0.00 (-0.04, 0.03)	0.019	8.46E-01
		Weighted mode	73	-0.02 (-0.07, 0.03)	0.023	3.85E-01
BMI (GIANT)	Eosinophil count	Inverse variance weighted[a]	72	0.00 (-0.03, 0.04)	0.017	8.16E-01
		MR Egger	72	0.00 (-0.08, 0.08)	0.040	9.37E-01
		Weighted median	72	-0.02 (-0.06, 0.01)	0.017	1.53E-01
		Weighted mode	72	-0.02 (-0.07, 0.03)	0.025	3.57E-01
BMI (GIANT)	Lymphocyte count	Inverse variance weighted[a]	70	-0.03 (-0.07, 0.01)	0.019	1.30E-01
		MR Egger	70	-0.07 (-0.16, 0.01)	0.045	1.02E-01
		Weighted median	70	-0.04 (-0.08, -0.01)	0.018	1.74E-02
		Weighted mode	70	-0.06 (-0.10, -0.02)	0.019	1.87E-03
BMI (GIANT)	Monocyte count	Inverse variance weighted[a]	73	-0.06 (-0.11, -0.02)	0.022	3.75E-03
		MR Egger	73	-0.09 (-0.19, 0.02)	0.053	1.01E-01
		Weighted median	73	-0.03 (-0.07, 0.00)	0.017	6.05E-02
		Weighted mode	73	-0.01 (-0.05, 0.02)	0.019	4.99E-01
BMI (GIANT)	Neutrophil count	Inverse variance weighted[a]	72	0.00 (-0.04, 0.04)	0.022	9.79E-01
		MR Egger	72	-0.01 (-0.11, 0.09)	0.052	8.37E-01
		Weighted median	72	0.01 (-0.02, 0.05)	0.019	4.63E-01
		Weighted mode	72	0.06 (0.01, 0.11)	0.027	2.54E-02
BMI (GIANT)	White blood cell count	Inverse variance weighted[a]	70	-0.01 (-0.05, 0.03)	0.021	5.88E-01
		MR Egger	70	-0.03 (-0.13, 0.07)	0.050	5.09E-01
		Weighted median	70	0.04 (0.00, 0.07)	0.018	2.68E-02
		Weighted mode	70	0.06 (0.01, 0.11)	0.025	2.40E-02
WC (GIANT)	FEV1	Inverse variance weighted[a]	41	-0.13 (-0.20, -0.07)	0.035	9.95E-05
		MR Egger	41	-0.01 (-0.25, 0.23)	0.122	9.61E-01
		Weighted median	41	-0.12 (-0.18, -0.07)	0.028	1.04E-05
		Weighted mode	41	-0.12 (-0.23, -0.01)	0.057	4.48E-02
WC (GIANT)	FVC	Inverse variance weighted[a]	40	-0.20 (-0.26, -0.14)	0.031	9.41E-11
		MR Egger	40	-0.23 (-0.44, -0.01)	0.110	4.59E-02
		Weighted median	40	-0.23 (-0.28, -0.18)	0.027	2.65E-17
		Weighted mode	40	-0.27 (-0.37, -0.17)	0.051	5.17E-06
WC (UKB)	CRP	Inverse variance weighted[a]	153	0.36 (0.30, 0.42)	0.031	2.53E-30
		MR Egger	153	0.37 (0.16, 0.58)	0.106	5.88E-04
		Weighted median	153	0.41 (0.34, 0.48)	0.038	1.62E-27
		Weighted mode	153	0.47 (0.32, 0.62)	0.076	4.24E-09
WC (GIANT)	GlycA	Inverse variance weighted[a]	41	0.20 (0.13, 0.26)	0.033	2.44E-09
		MR Egger	41	0.21 (-0.03, 0.44)	0.120	9.10E-02
		Weighted median	41	0.21 (0.13, 0.30)	0.042	3.10E-07
		Weighted mode	41	0.23 (0.08, 0.39)	0.080	5.60E-03
WC (UKB)	IL-1Ra	Inverse variance weighted	302	0.25 (0.16, 0.33)	0.044	1.83E-08
		MR Egger	302	0.53 (0.29, 0.76)	0.120	1.69E-05
		Weighted median	302	0.30 (0.15, 0.44)	0.075	8.33E-05

		Weighted mode	302	0.25 (-0.02, 0.52)	0.138	7.03E-02
WC (UKB)	IL-6	Inverse variance weighted	301	0.28 (0.18, 0.38)	0.051	2.86E-08
		MR Egger	301	0.49 (0.22, 0.77)	0.140	4.83E-04
		Weighted median	301	0.29 (0.12, 0.47)	0.088	7.98E-04
		Weighted mode	301	0.30 (-0.03, 0.62)	0.164	7.17E-02
WC (UKB)	IL-8	Inverse variance weighted	323	0.09 (0.00, 0.18)	0.046	5.93E-02
		MR Egger	323	0.04 (-0.21, 0.29)	0.126	7.61E-01
		Weighted median	323	0.12 (-0.03, 0.27)	0.078	1.19E-01
		Weighted mode	323	0.17 (-0.10, 0.44)	0.137	2.23E-01
WC (UKB)	IL-18	Inverse variance weighted	300	0.06 (-0.03, 0.15)	0.044	1.72E-01
		MR Egger	300	-0.02 (-0.26, 0.21)	0.122	8.47E-01
		Weighted median	300	0.01 (-0.14, 0.15)	0.076	9.43E-01
		Weighted mode	300	-0.06 (-0.30, 0.19)	0.125	6.37E-01
WC (UKB)	IL-27	Inverse variance weighted	302	-0.07 (-0.16, 0.01)	0.043	8.50E-02
		MR Egger	302	-0.17 (-0.41, 0.06)	0.119	1.46E-01
		Weighted median	302	-0.15 (-0.29, 0.00)	0.076	5.35E-02
		Weighted mode	302	-0.11 (-0.38, 0.16)	0.138	4.24E-01
WC (GIANT)	Basophil count	Inverse variance weighted[a]	35	-0.03 (-0.07, 0.02)	0.023	2.49E-01
		MR Egger	35	0.08 (-0.08, 0.25)	0.085	3.32E-01
		Weighted median	35	-0.03 (-0.07, 0.02)	0.023	2.65E-01
		Weighted mode	35	-0.05 (-0.13, 0.03)	0.041	2.55E-01
WC (GIANT)	Eosinophil count	Inverse variance weighted[a]	35	-0.02 (-0.08, 0.04)	0.031	5.35E-01
		MR Egger	35	0.17 (-0.05, 0.40)	0.116	1.44E-01
		Weighted median	35	-0.04 (-0.09, 0.02)	0.026	1.69E-01
		Weighted mode	35	-0.03 (-0.15, 0.08)	0.058	5.81E-01
WC (GIANT)	Lymphocyte count	Inverse variance weighted[a]	33	-0.03 (-0.10, 0.04)	0.035	3.65E-01
		MR Egger	33	-0.14 (-0.39, 0.11)	0.130	2.91E-01
		Weighted median	33	-0.04 (-0.09, 0.00)	0.023	5.62E-02
		Weighted mode	33	-0.05 (-0.12, 0.01)	0.034	1.27E-01
WC (GIANT)	Monocyte count	Inverse variance weighted[a]	35	-0.04 (-0.10, 0.03)	0.035	2.94E-01
		MR Egger	35	-0.30 (-0.55, -0.06)	0.124	2.00E-02
		Weighted median	35	-0.04 (-0.09, 0.01)	0.025	1.28E-01
		Weighted mode	35	-0.05 (-0.13, 0.03)	0.043	2.53E-01
WC (GIANT)	Neutrophil count	Inverse variance weighted[a]	35	-0.02 (-0.06, 0.03)	0.023	5.19E-01
		MR Egger	35	-0.10 (-0.27, 0.08)	0.089	2.77E-01
		Weighted median	35	-0.01 (-0.06, 0.03)	0.023	6.52E-01
		Weighted mode	35	0.00 (-0.11, 0.10)	0.052	9.24E-01
WC (GIANT)	White blood cell count	Inverse variance weighted[a]	35	-0.02 (-0.08, 0.03)	0.028	3.98E-01
		MR Egger	35	-0.19 (-0.39, 0.02)	0.105	8.60E-02
		Weighted median	35	-0.01 (-0.06, 0.03)	0.024	5.68E-01
		Weighted mode	35	0.01 (-0.09, 0.10)	0.049	9.04E-01

WHR (GIANT)	FEV1	Inverse variance weighted[a]	29	-0.07 (-0.14, 0.00)	0.035	4.30E-02
		MR Egger	29	-0.01 (-0.32, 0.30)	0.158	9.50E-01
		Weighted median	29	-0.03 (-0.10, 0.04)	0.035	3.57E-01
		Weighted mode	29	0.01 (-0.13, 0.15)	0.072	9.17E-01
WHR (GIANT)	FVC	Inverse variance weighted[a]	30	-0.19 (-0.26, -0.13)	0.033	6.30E-09
		MR Egger	30	-0.13 (-0.42, 0.17)	0.151	4.12E-01
		Weighted median	30	-0.18 (-0.25, -0.12)	0.033	3.48E-08
		Weighted mode	30	-0.21 (-0.33, -0.10)	0.059	1.22E-03
WHR (GIANT and UKB[b])	CRP	Inverse variance weighted[a]	245	0.24 (0.19, 0.29)	0.027	2.14E-19
		MR Egger	245	0.15 (-0.02, 0.32)	0.086	7.73E-02
		Weighted median	245	0.21 (0.15, 0.26)	0.030	5.57E-12
		Weighted mode	245	0.14 (0.00, 0.28)	0.071	4.95E-02
WHR (GIANT)	GlycA	Inverse variance weighted[a]	29	0.17 (0.04, 0.29)	0.064	8.18E-03
		MR Egger	29	-0.16 (-0.72, 0.40)	0.284	5.83E-01
		Weighted median	29	0.17 (0.05, 0.30)	0.063	6.55E-03
		Weighted mode	29	0.23 (0.02, 0.45)	0.108	3.85E-02
WHR (GIANT and UKB[b])	IL-1Ra	Inverse variance weighted	250	0.14 (0.05, 0.23)	0.045	1.68E-03
		MR Egger	250	0.14 (-0.09, 0.37)	0.118	2.29E-01
		Weighted median	250	0.20 (0.05, 0.34)	0.074	7.90E-03
		Weighted mode	250	0.26 (-0.01, 0.52)	0.135	5.93E-02
WHR (GIANT and UKB[b])	IL-6	Inverse variance weighted	283	0.12 (0.02, 0.22)	0.051	2.34E-02
		MR Egger	283	0.16 (-0.11, 0.43)	0.138	2.55E-01
		Weighted median	283	0.08 (-0.08, 0.24)	0.081	3.14E-01
		Weighted mode	283	0.07 (-0.24, 0.37)	0.153	6.71E-01
WHR (GIANT and UKB[b])	IL-8	Inverse variance weighted	271	0.08 (-0.02, 0.17)	0.047	1.05E-01
		MR Egger	271	0.16 (-0.08, 0.40)	0.122	1.84E-01
		Weighted median	271	0.10 (-0.04, 0.25)	0.075	1.61E-01
		Weighted mode	271	0.20 (-0.09, 0.50)	0.149	1.72E-01
WHR (GIANT and UKB[b])	IL-18	Inverse variance weighted	276	0.09 (0.01, 0.18)	0.043	3.01E-02
		MR Egger	276	0.24 (0.02, 0.46)	0.113	3.52E-02
		Weighted median	276	0.15 (0.01, 0.29)	0.071	3.49E-02
		Weighted mode	276	0.14 (-0.17, 0.44)	0.154	3.77E-01
WHR (GIANT and UKB[b])	IL-27	Inverse variance weighted	259	-0.04 (-0.13, 0.04)	0.044	3.44E-01
		MR Egger	259	-0.16 (-0.38, 0.06)	0.114	1.61E-01
		Weighted median	259	-0.05 (-0.19, 0.08)	0.070	4.33E-01
		Weighted mode	259	-0.03 (-0.25, 0.20)	0.115	7.97E-01
WHR (GIANT)	Basophil count	Inverse variance weighted[a]	26	0.02 (-0.04, 0.08)	0.029	5.51E-01
		MR Egger	26	-0.05 (-0.31, 0.21)	0.132	7.12E-01
		Weighted median	26	0.00 (-0.05, 0.06)	0.028	8.95E-01
		Weighted mode	26	-0.01 (-0.07, 0.06)	0.033	8.59E-01
WHR (GIANT)	Eosinophil count	Inverse variance weighted[a]	25	0.07 (0.00, 0.15)	0.038	5.58E-02

		MR Egger	25	-0.09 (-0.42, 0.23)	0.166	5.86E-01
		Weighted median	25	0.03 (-0.03, 0.10)	0.033	3.03E-01
		Weighted mode	25	0.00 (-0.11, 0.10)	0.052	9.25E-01
WHR (GIANT)	Lymphocyte count	Inverse variance weighted[a]	25	0.07 (-0.05, 0.19)	0.061	2.28E-01
		MR Egger	25	-0.26 (-0.78, 0.25)	0.264	3.27E-01
		Weighted median	25	-0.04 (-0.11, 0.02)	0.035	2.02E-01
		Weighted mode	25	-0.06 (-0.14, 0.03)	0.043	1.97E-01
WHR (GIANT)	Monocyte count	Inverse variance weighted[a]	26	0.00 (-0.09, 0.10)	0.049	9.54E-01
		MR Egger	26	-0.04 (-0.47, 0.39)	0.221	8.59E-01
		Weighted median	26	0.02 (-0.04, 0.08)	0.030	4.30E-01
		Weighted mode	26	0.02 (-0.05, 0.10)	0.038	5.80E-01
WHR (GIANT)	Neutrophil count	Inverse variance weighted[a]	26	-0.01 (-0.12, 0.10)	0.057	9.07E-01
		MR Egger	26	0.47 (0.01, 0.93)	0.235	5.76E-02
		Weighted median	26	0.03 (-0.04, 0.10)	0.036	3.81E-01
		Weighted mode	26	0.11 (0.02, 0.19)	0.043	1.86E-02
WHR (GIANT)	White blood cell count	Inverse variance weighted[a]	26	0.05 (-0.07, 0.18)	0.063	3.89E-01
		MR Egger	26	0.24 (-0.31, 0.79)	0.280	3.94E-01
		Weighted median	26	0.02 (-0.04, 0.09)	0.034	5.23E-01
		Weighted mode	26	0.04 (-0.04, 0.12)	0.041	3.68E-01

$\hat{\beta}$ represents the change in a potential mediator (in standard deviation unit, except for CRP, whose unit was one-unit natural-log-transformed CRP) associated with per standard deviation increase in an obesity-related trait.

[a] denotes the use of a random-effects Inverse variance weighted.

[b] The data were originated from a meta-analysis combining the GWAS data provided by GIANT and UKB.

Abbreviations: SNPs, single nucleotide polymorphisms; CI, confidence interval; SE, standard error; BMI, body mass index; WC, waist circumference; WHR, waist-to-hip ratio; GIANT, Genetic Investigation of ANthropometric Traits; UKB, UK Biobank; FEV1, forced expiratory volume in the first second; FVC, forced vital capacity; CRP, C-reactive protein; GlycA, glycoprotein acetyls; IL-1Ra, interleukin-1-receptor antagonist; IL-6, interleukin-6; IL-8, interleukin-8; IL-18, interleukin-18; IL-27, interleukin-27.

Table S7. Results of sensitivity analyses for the effects of obesity-related traits on potential mediators.

Obesity-related traits (data sources)	Potential mediators	Pleiotropy			Heterogeneity			MR-PRESSO					
		Egger intercept	SE	*P*	*Q*	*dfs*	*P*	$\hat{\beta}$	SE	*P*	$\hat{\beta}$ (Outlier SNPs Removed)	SE (Outlier SNPs Removed)	*P* (Outlier SNPs Removed)
BMI (GIANT)	FEV1	-2.36E-03	0.002	0.122	320.804	75	3.86E-32	-0.099	0.023	3.85E-05	-0.088	0.019	1.44E-05
BMI (GIANT)	FVC	-1.43E-03	0.002	0.396	394.732	76	1.45E-44	-0.157	0.025	1.93E-08	-0.168	0.019	1.05E-12
BMI (GIANT and UKB[a])	CRP	6.27E-05	0.001	0.918	659.603	452	5.82E-10	0.371	0.014	7.35E-92	0.385	0.014	7.28E-103
BMI (GIANT)	GlycA	3.45E-03	0.002	0.036	128.363	77	2.18E-04	0.169	0.025	1.36E-09	0.175	0.024	1.71E-10
BMI (GIANT and UKB[a])	IL-1Ra	-4.04E-03	0.002	0.010	191.805	372	1.00E+00	0.173	0.026	1.29E-10	NA	NA	NA
BMI (GIANT and UKB[a])	IL-6	-3.52E-03	0.002	0.051	188.126	384	1.00E+00	0.186	0.029	4.01E-10	NA	NA	NA
BMI (GIANT and UKB[a])	IL-8	5.02E-04	0.002	0.760	211.820	397	1.00E+00	0.050	0.027	7.11E-02	NA	NA	NA
BMI (GIANT and UKB[a])	IL-18	6.00E-04	0.002	0.699	198.559	389	1.00E+00	-0.003	0.026	9.08E-01	NA	NA	NA
BMI (GIANT and UKB[a])	IL-27	7.38E-04	0.002	0.632	191.831	388	1.00E+00	0.021	0.025	3.99E-01	NA	NA	NA
BMI (GIANT)	Basophil count	-5.60E-04	0.001	0.508	117.437	72	5.80E-04	-0.006	0.013	6.40E-01	NA	NA	NA
BMI (GIANT)	Eosinophil count	2.13E-05	0.001	0.985	220.886	71	2.77E-17	0.004	0.017	8.17E-01	0.014	0.014	2.96E-01
BMI (GIANT)	Lymphocyte count	1.44E-03	0.001	0.264	297.709	69	3.53E-30	-0.029	0.019	1.34E-01	-0.049	0.014	7.36E-04
BMI (GIANT)	Monocyte count	7.41E-04	0.001	0.617	431.598	72	1.08E-52	-0.064	0.022	4.97E-03	-0.029	0.017	8.40E-02
BMI (GIANT)	Neutrophil count	3.53E-04	0.001	0.811	396.228	71	1.11E-46	0.001	0.022	9.79E-01	-0.002	0.016	9.25E-01
BMI (GIANT)	White blood cell count	6.87E-04	0.001	0.631	381.675	69	8.00E-45	-0.011	0.021	5.90E-01	-0.003	0.016	8.66E-01
WC (GIANT)	FEV1	-3.59E-03	0.003	0.281	206.808	40	2.35E-24	-0.126	0.031	2.13E-04	-0.117	0.023	1.68E-05
WC (GIANT)	FVC	7.71E-04	0.003	0.796	154.855	39	9.81E-16	-0.190	0.028	2.56E-08	-0.208	0.021	8.54E-12
WC (UKB)	CRP	-1.91E-04	0.002	0.902	296.594	152	2.17E-11	0.358	0.031	1.05E-22	0.368	0.028	1.80E-26
WC (GIANT)	GlycA	-2.40E-04	0.003	0.941	63.129	40	1.13E-02	0.175	0.031	1.27E-06	NA	NA	NA
WC (UKB)	IL-1Ra	-4.83E-03	0.002	0.013	180.731	301	1.00E+00	0.246	0.033	1.42E-12	NA	NA	NA
WC (UKB)	IL-6	-3.66E-03	0.002	0.104	150.194	300	1.00E+00	0.274	0.036	4.06E-13	NA	NA	NA
WC (UKB	IL-8	8.06E-04	0.002	0.687	205.545	322	1.00E+00	0.075	0.036	3.68E-02	NA	NA	NA
WC (UKB	IL-18	1.43E-03	0.002	0.462	169.500	299	1.00E+00	0.061	0.033	6.00E-02	NA	NA	NA
WC (UKB)	IL-27	1.71E-03	0.002	0.372	203.617	301	1.00E+00	-0.087	0.035	1.36E-02	NA	NA	NA
WC (GIANT)	Basophil count	-2.99E-03	0.002	0.190	81.212	34	9.76E-06	-0.019	0.020	3.40E-01	-0.012	0.015	4.30E-01
WC (GIANT)	Eosinophil count	-5.25E-03	0.003	0.094	166.974	34	1.81E-19	-0.022	0.028	4.29E-01	-0.028	0.021	1.89E-01
WC (GIANT)	Lymphocyte count	2.95E-03	0.003	0.393	201.329	32	1.90E-26	-0.034	0.030	2.64E-01	-0.046	0.015	6.47E-03
WC (GIANT)	Monocyte count	7.25E-03	0.003	0.033	225.254	34	4.55E-30	-0.024	0.030	4.37E-01	-0.009	0.019	6.56E-01
WC (GIANT)	Neutrophil count	2.26E-03	0.002	0.340	98.637	34	3.26E-08	0.011	0.022	6.33E-01	0.015	0.021	4.97E-01
WC (GIANT)	White blood cell count	4.38E-03	0.003	0.119	154.949	34	2.28E-17	0.000	0.026	9.93E-01	0.000	0.022	9.85E-01
WHR (GIANT)	FEV1	-1.52E-03	0.004	0.701	89.194	28	2.65E-08	-0.084	0.033	1.81E-02	-0.088	0.031	8.01E-03
WHR (GIANT)	FVC	-1.67E-03	0.004	0.657	86.396	29	1.29E-07	-0.190	0.031	1.13E-06	-0.202	0.030	2.09E-07
WHR (GIANT and UKB[a])	CRP	1.44E-03	0.001	0.279	607.321	244	6.34E-33	0.241	0.026	7.36E-18	0.230	0.024	4.17E-19
WHR (GIANT)	GlycA	8.28E-03	0.007	0.249	99.050	28	7.22E-10	0.161	0.060	1.17E-02	0.151	0.057	1.28E-02
WHR (GIANT and UKB[a])	IL-1Ra	-1.13E-05	0.002	0.995	138.986	249	1.00E+00	0.143	0.033	2.18E-05	NA	NA	NA

WHR (GIANT and UKB[a])	IL-6	-7.47E-04	0.002	0.742	140.726	282	1.00E+00	0.124	0.035	4.65E-04	NA	NA	NA
WHR (GIANT and UKB[a])	IL-8	-1.58E-03	0.002	0.443	143.052	270	1.00E+00	0.071	0.034	3.59E-02	NA	NA	NA
WHR (GIANT and UKB[a])	IL-18	-2.68E-03	0.002	0.164	148.023	275	1.00E+00	0.099	0.031	1.57E-03	NA	NA	NA
WHR (GIANT and UKB[a])	IL-27	2.21E-03	0.002	0.260	124.579	258	1.00E+00	-0.028	0.030	3.54E-01	NA	NA	NA
WHR (GIANT)	Basophil count	1.66E-03	0.003	0.607	68.134	25	7.26E-06	0.013	0.028	6.43E-01	0.010	0.023	6.59E-01
WHR (GIANT)	Eosinophil count	4.13E-03	0.004	0.322	111.334	24	3.30E-13	0.060	0.036	1.08E-01	0.046	0.028	1.18E-01
WHR (GIANT)	Lymphocyte count	8.44E-03	0.006	0.201	326.868	24	6.27E-55	0.033	0.062	6.05E-01	-0.006	0.041	8.92E-01
WHR (GIANT)	Monocyte count	1.06E-03	0.005	0.845	229.130	25	6.82E-35	-0.023	0.049	6.35E-01	-0.004	0.033	9.13E-01
WHR (GIANT)	Neutrophil count	-1.19E-02	0.006	0.049	286.972	25	2.44E-46	-0.014	0.053	7.97E-01	-0.023	0.035	5.19E-01
WHR (GIANT)	White blood cell count	-4.71E-03	0.007	0.495	384.521	25	4.54E-66	0.029	0.061	6.32E-01	0.044	0.031	1.75E-01

$\hat{\beta}$ represents the change in a potential mediator (in standard deviation unit, except for CRP, whose unit was one-unit natural-log-transformed CRP) associated with per standard deviation increase in an obesity-related trait.

[a] The data were originated from a meta-analysis combining the GWAS data provided by GIANT and UKB.

Abbreviations: MR-PRESSO, MR-pleiotropy residual sum and outlier method; Egger intercept, intercept term of MR Egger regression; SE, standard error; dfs, degrees of freedom; SNPs, single nucleotide polymorphisms; BMI, body mass index; WC, waist circumference; WHR, waist-to-hip ratio; GIANT, Genetic Investigation of ANthropometric Traits; UKB, UK Biobank; FEV1, forced expiratory volume in the first second; FVC, forced vital capacity; CRP, C-reactive protein; GlycA, glycoprotein acetyls; IL-1Ra, interleukin-1-receptor antagonist; IL-6, interleukin-6; IL-8, interleukin-8; IL-18, interleukin-18; IL-27, interleukin-27.

Table S8. Effects of potential mediators on lower respiratory tract infections estimated using different methods.

Potential mediators	Lower respiratory tract infections	Methods	No. of SNPs	$\widehat{\beta}$	SE	OR (95%CI)	*P*
FEV1	Acute bronchitis	Inverse variance weighted[a]	253	-0.220	0.045	0.80 (0.73, 0.88)	1.02E-06
		MR Egger	253	-0.117	0.149	0.89 (0.66, 1.19)	4.34E-01
		Weighted median	253	-0.215	0.067	0.81 (0.71, 0.92)	1.41E-03
		Weighted mode	253	-0.219	0.135	0.80 (0.62, 1.05)	1.05E-01
	Acute bronchiolitis	Inverse variance weighted	253	-0.365	0.114	0.69 (0.56, 0.87)	1.35E-03
		MR Egger	253	-0.296	0.396	0.74 (0.34, 1.61)	4.54E-01
		Weighted median	253	-0.391	0.181	0.68 (0.47, 0.96)	3.05E-02
		Weighted mode	253	-0.476	0.407	0.62 (0.28, 1.38)	2.43E-01
	Bronchiectasis	Inverse variance weighted	253	0.119	0.106	1.13 (0.91, 1.39)	2.62E-01
		MR Egger	253	0.470	0.369	1.60 (0.78, 3.30)	2.05E-01
		Weighted median	253	0.002	0.172	1.00 (0.72, 1.40)	9.91E-01
		Weighted mode	253	-0.063	0.450	0.94 (0.39, 2.27)	8.89E-01
	Influenza	Inverse variance weighted	253	-0.028	0.054	0.97 (0.87, 1.08)	6.08E-01
		MR Egger	253	0.022	0.184	1.02 (0.71, 1.47)	9.07E-01
		Weighted median	253	-0.023	0.082	0.98 (0.83, 1.15)	7.77E-01
		Weighted mode	253	-0.009	0.306	0.99 (0.54, 1.81)	9.78E-01
	Pneumonia	Inverse variance weighted[a]	253	-0.073	0.028	0.93 (0.88, 0.98)	9.76E-03
		MR Egger	253	0.096	0.093	1.10 (0.92, 1.32)	3.05E-01
		Weighted median	253	-0.074	0.037	0.93 (0.86, 1.00)	4.65E-02
		Weighted mode	253	0.128	0.118	1.14 (0.90, 1.43)	2.79E-01
FVC	Acute bronchitis	Inverse variance weighted[a]	229	-0.144	0.048	0.87 (0.79, 0.95)	2.88E-03
		MR Egger	229	-0.209	0.171	0.81 (0.58, 1.14)	2.24E-01
		Weighted median	229	-0.141	0.066	0.87 (0.76, 0.99)	3.24E-02
		Weighted mode	229	-0.129	0.212	0.88 (0.58, 1.33)	5.43E-01
	Acute bronchiolitis	Inverse variance weighted	229	-0.187	0.123	0.83 (0.65, 1.05)	1.28E-01
		MR Egger	229	0.174	0.435	1.19 (0.51, 2.79)	6.90E-01
		Weighted median	229	-0.340	0.193	0.71 (0.49, 1.04)	7.78E-02
		Weighted mode	229	-0.609	0.532	0.54 (0.19, 1.54)	2.54E-01
	Bronchiectasis	Inverse variance weighted	229	-0.110	0.114	0.90 (0.72, 1.12)	3.36E-01
		MR Egger	229	0.288	0.411	1.33 (0.60, 2.98)	4.84E-01
		Weighted median	229	-0.009	0.170	0.99 (0.71, 1.38)	9.56E-01
		Weighted mode	229	0.051	0.461	1.05 (0.43, 2.60)	9.12E-01
	Influenza	Inverse variance weighted	229	-0.090	0.059	0.91 (0.81, 1.03)	1.26E-01
		MR Egger	229	-0.215	0.217	0.81 (0.53, 1.23)	3.23E-01
		Weighted median	229	-0.147	0.088	0.86 (0.73, 1.03)	9.48E-02
		Weighted mode	229	-0.164	0.232	0.85 (0.54, 1.34)	4.79E-01
	Pneumonia	Inverse variance weighted[a]	229	-0.064	0.032	0.94 (0.88, 1.00)	4.15E-02
		MR Egger	229	-0.162	0.112	0.85 (0.68, 1.06)	1.48E-01
		Weighted median	229	-0.012	0.040	0.99 (0.91, 1.07)	7.76E-01

		Weighted mode	229	0.056	0.111	1.06 (0.85, 1.31)	6.12E-01
CRP	Acute bronchitis	Inverse variance weighted	54	0.130	0.029	1.14 (1.08, 1.21)	6.89E-06
		MR Egger	54	0.114	0.048	1.12 (1.02, 1.23)	2.03E-02
		Weighted median	54	0.064	0.050	1.07 (0.97, 1.18)	2.05E-01
		Weighted mode	54	0.118	0.038	1.13 (1.05, 1.21)	2.80E-03
	Acute bronchiolitis	Inverse variance weighted	54	0.027	0.081	1.03 (0.88, 1.20)	7.37E-01
		MR Egger	54	-0.085	0.118	0.92 (0.73, 1.16)	4.73E-01
		Weighted median	54	0.013	0.115	1.01 (0.81, 1.27)	9.08E-01
		Weighted mode	54	0.002	0.097	1.00 (0.83, 1.21)	9.87E-01
	Bronchiectasis	Inverse variance weighted	54	0.059	0.076	1.06 (0.91, 1.23)	4.34E-01
		MR Egger	54	0.101	0.123	1.11 (0.87, 1.41)	4.15E-01
		Weighted median	54	0.105	0.108	1.11 (0.90, 1.37)	3.31E-01
		Weighted mode	54	0.068	0.084	1.07 (0.91, 1.26)	4.19E-01
	Influenza	Inverse variance weighted[a]	54	0.185	0.051	1.20 (1.09, 1.33)	2.85E-04
		MR Egger	54	0.051	0.071	1.05 (0.92, 1.21)	4.73E-01
		Weighted median	54	0.121	0.058	1.13 (1.01, 1.27)	3.82E-02
		Weighted mode	54	0.120	0.046	1.13 (1.03, 1.23)	1.13E-02
	Pneumonia	Inverse variance weighted[a]	54	0.157	0.029	1.17 (1.11, 1.24)	7.71E-08
		MR Egger	54	0.126	0.043	1.13 (1.04, 1.23)	4.86E-03
		Weighted median	54	0.090	0.033	1.09 (1.03, 1.17)	5.61E-03
		Weighted mode	54	0.163	0.027	1.18 (1.12, 1.24)	1.53E-07
GlycA	Acute bronchitis	Inverse variance weighted	53	0.031	0.039	1.03 (0.96, 1.11)	4.28E-01
		MR Egger	53	0.019	0.067	1.02 (0.89, 1.16)	7.73E-01
		Weighted median	53	0.044	0.062	1.04 (0.93, 1.18)	4.77E-01
		Weighted mode	53	0.066	0.059	1.07 (0.95, 1.20)	2.72E-01
	Acute bronchiolitis	Inverse variance weighted	53	-0.010	0.109	0.99 (0.80, 1.23)	9.30E-01
		MR Egger	53	-0.172	0.188	0.84 (0.58, 1.22)	3.65E-01
		Weighted median	53	-0.007	0.174	0.99 (0.71, 1.40)	9.70E-01
		Weighted mode	53	0.016	0.177	1.02 (0.72, 1.44)	9.28E-01
	Bronchiectasis	Inverse variance weighted[a]	53	-0.163	0.130	0.85 (0.66, 1.10)	2.12E-01
		MR Egger	53	-0.211	0.224	0.81 (0.52, 1.26)	3.51E-01
		Weighted median	53	0.190	0.161	1.21 (0.88, 1.66)	2.38E-01
		Weighted mode	53	0.227	0.152	1.26 (0.93, 1.69)	1.40E-01
	Influenza	Inverse variance weighted	53	0.015	0.052	1.02 (0.92, 1.12)	7.69E-01
		MR Egger	53	-0.114	0.095	0.89 (0.74, 1.08)	2.39E-01
		Weighted median	53	-0.046	0.078	0.95 (0.82, 1.11)	5.51E-01
		Weighted mode	53	-0.026	0.071	0.97 (0.85, 1.12)	7.12E-01
	Pneumonia	Inverse variance weighted[a]	53	0.037	0.027	1.04 (0.98, 1.09)	1.76E-01
		MR Egger	53	-0.050	0.045	0.95 (0.87, 1.04)	2.70E-01
		Weighted median	53	0.015	0.036	1.01 (0.95, 1.09)	6.86E-01
		Weighted mode	53	0.031	0.037	1.03 (0.96, 1.11)	4.03E-01

IL-1Ra	Acute bronchitis	Inverse variance weighted	23	0.048	0.028	1.05 (0.99, 1.11)	8.90E-02
		MR Egger	23	0.019	0.053	1.02 (0.92, 1.13)	7.30E-01
		Weighted median	23	0.011	0.040	1.01 (0.94, 1.09)	7.87E-01
		Weighted mode	23	0.019	0.049	1.02 (0.93, 1.12)	6.96E-01
	Acute bronchiolitis	Inverse variance weighted	23	-0.012	0.079	0.99 (0.85, 1.15)	8.80E-01
		MR Egger	23	-0.171	0.149	0.84 (0.63, 1.13)	2.64E-01
		Weighted median	23	-0.112	0.115	0.89 (0.71, 1.12)	3.29E-01
		Weighted mode	23	-0.096	0.134	0.91 (0.70, 1.18)	4.78E-01
	Bronchiectasis	Inverse variance weighted	23	0.122	0.074	1.13 (0.98, 1.31)	9.83E-02
		MR Egger	23	-0.091	0.139	0.91 (0.70, 1.20)	5.17E-01
		Weighted median	23	0.103	0.105	1.11 (0.90, 1.36)	3.28E-01
		Weighted mode	23	0.036	0.107	1.04 (0.84, 1.28)	7.38E-01
	Influenza	Inverse variance weighted	23	0.017	0.038	1.02 (0.94, 1.10)	6.49E-01
		MR Egger	23	-0.064	0.071	0.94 (0.82, 1.08)	3.74E-01
		Weighted median	23	-0.009	0.053	0.99 (0.89, 1.10)	8.63E-01
		Weighted mode	23	-0.035	0.055	0.97 (0.87, 1.08)	5.35E-01
	Pneumonia	Inverse variance weighted[a]	23	-0.013	0.022	0.99 (0.95, 1.03)	5.43E-01
		MR Egger	23	-0.025	0.041	0.97 (0.90, 1.06)	5.45E-01
		Weighted median	23	-0.023	0.027	0.98 (0.93, 1.03)	3.91E-01
		Weighted mode	23	-0.056	0.031	0.95 (0.89, 1.00)	8.10E-02
IL-6	Acute bronchitis	Inverse variance weighted	12	0.059	0.041	1.06 (0.98, 1.15)	1.50E-01
		MR Egger	12	0.364	0.097	1.44 (1.19, 1.74)	3.86E-03
		Weighted median	12	0.031	0.058	1.03 (0.92, 1.16)	5.96E-01
		Weighted mode	12	0.022	0.062	1.02 (0.91, 1.15)	7.31E-01
	Acute bronchiolitis	Inverse variance weighted	12	0.064	0.114	1.07 (0.85, 1.33)	5.74E-01
		MR Egger	12	0.382	0.274	1.47 (0.86, 2.51)	1.93E-01
		Weighted median	12	0.237	0.153	1.27 (0.94, 1.71)	1.22E-01
		Weighted mode	12	0.286	0.161	1.33 (0.97, 1.83)	1.05E-01
	Bronchiectasis	Inverse variance weighted	12	-0.130	0.107	0.88 (0.71, 1.08)	2.24E-01
		MR Egger	12	0.148	0.222	1.16 (0.75, 1.79)	5.19E-01
		Weighted median	12	-0.125	0.151	0.88 (0.66, 1.19)	4.08E-01
		Weighted mode	12	-0.160	0.171	0.85 (0.61, 1.19)	3.70E-01
	Influenza	Inverse variance weighted	12	-0.019	0.055	0.98 (0.88, 1.09)	7.25E-01
		MR Egger	12	-0.062	0.111	0.94 (0.76, 1.17)	5.88E-01
		Weighted median	12	-0.116	0.073	0.89 (0.77, 1.03)	1.14E-01
		Weighted mode	12	-0.127	0.081	0.88 (0.75, 1.03)	1.43E-01
	Pneumonia	Inverse variance weighted	12	0.076	0.023	1.08 (1.03, 1.13)	8.51E-04
		MR Egger	12	0.091	0.046	1.10 (1.00, 1.20)	7.46E-02
		Weighted median	12	0.073	0.031	1.08 (1.01, 1.14)	1.77E-02
		Weighted mode	12	0.073	0.037	1.08 (1.00, 1.16)	7.11E-02
IL-8	Acute bronchitis	Inverse variance weighted[a]	14	-0.007	0.074	0.99 (0.86, 1.15)	9.21E-01

		MR Egger	14	0.073	0.166	1.08 (0.78, 1.49)	6.69E-01
		Weighted median	14	0.070	0.084	1.07 (0.91, 1.26)	4.01E-01
		Weighted mode	14	0.185	0.149	1.20 (0.90, 1.61)	2.37E-01
	Acute bronchiolitis	Inverse variance weighted	14	0.132	0.153	1.14 (0.85, 1.54)	3.89E-01
		MR Egger	14	0.107	0.403	1.11 (0.51, 2.45)	7.95E-01
		Weighted median	14	0.337	0.205	1.40 (0.94, 2.09)	1.00E-01
		Weighted mode	14	0.356	0.314	1.43 (0.77, 2.64)	2.78E-01
	Bronchiectasis	Inverse variance weighted	14	-0.191	0.143	0.83 (0.62, 1.09)	1.84E-01
		MR Egger	14	-0.334	0.382	0.72 (0.34, 1.51)	3.98E-01
		Weighted median	14	-0.036	0.204	0.96 (0.65, 1.44)	8.59E-01
		Weighted mode	14	0.005	0.261	1.00 (0.60, 1.67)	9.86E-01
	Influenza	Inverse variance weighted	14	-0.002	0.073	1.00 (0.86, 1.15)	9.83E-01
		MR Egger	14	0.195	0.161	1.22 (0.89, 1.67)	2.49E-01
		Weighted median	14	-0.119	0.098	0.89 (0.73, 1.08)	2.25E-01
		Weighted mode	14	-0.150	0.158	0.86 (0.63, 1.17)	3.61E-01
	Pneumonia	Inverse variance weighted	14	-0.023	0.031	0.98 (0.92, 1.04)	4.61E-01
		MR Egger	14	0.112	0.081	1.12 (0.95, 1.31)	1.94E-01
		Weighted median	14	0.019	0.047	1.02 (0.93, 1.12)	6.84E-01
		Weighted mode	14	0.021	0.074	1.02 (0.88, 1.18)	7.80E-01
IL-18	Acute bronchitis	Inverse variance weighted	24	0.039	0.026	1.04 (0.99, 1.09)	1.31E-01
		MR Egger	24	0.109	0.050	1.12 (1.01, 1.23)	3.87E-02
		Weighted median	24	0.041	0.040	1.04 (0.96, 1.13)	3.09E-01
		Weighted mode	24	0.094	0.045	1.10 (1.01, 1.20)	4.76E-02
	Acute bronchiolitis	Inverse variance weighted	24	0.026	0.072	1.03 (0.89, 1.18)	7.14E-01
		MR Egger	24	0.040	0.156	1.04 (0.77, 1.41)	8.00E-01
		Weighted median	24	0.088	0.102	1.09 (0.89, 1.33)	3.86E-01
		Weighted mode	24	0.084	0.107	1.09 (0.88, 1.34)	4.43E-01
	Bronchiectasis	Inverse variance weighted	24	0.041	0.067	1.04 (0.91, 1.19)	5.45E-01
		MR Egger	24	0.239	0.139	1.27 (0.97, 1.67)	9.95E-02
		Weighted median	24	0.032	0.098	1.03 (0.85, 1.25)	7.43E-01
		Weighted mode	24	0.052	0.088	1.05 (0.89, 1.25)	5.65E-01
	Influenza	Inverse variance weighted	24	-0.061	0.034	0.94 (0.88, 1.01)	7.66E-02
		MR Egger	24	0.019	0.078	1.02 (0.87, 1.19)	8.12E-01
		Weighted median	24	-0.066	0.050	0.94 (0.85, 1.03)	1.88E-01
		Weighted mode	24	-0.044	0.045	0.96 (0.88, 1.05)	3.35E-01
	Pneumonia	Inverse variance weighted	24	-0.010	0.014	0.99 (0.96, 1.02)	4.70E-01
		MR Egger	24	-0.022	0.033	0.98 (0.92, 1.04)	5.20E-01
		Weighted median	24	-0.004	0.019	1.00 (0.96, 1.03)	8.23E-01
		Weighted mode	24	-0.013	0.021	0.99 (0.95, 1.03)	5.35E-01
IL-27	Acute bronchitis	Inverse variance weighted	21	-0.006	0.028	0.99 (0.94, 1.05)	8.42E-01
		MR Egger	21	-0.007	0.086	0.99 (0.84, 1.18)	9.36E-01

		Weighted median	21	0.003	0.039	1.00 (0.93, 1.08)	9.33E-01
		Weighted mode	21	0.011	0.040	1.01 (0.93, 1.09)	7.82E-01
	Acute bronchiolitis	Inverse variance weighted	21	-0.005	0.079	0.99 (0.85, 1.16)	9.45E-01
		MR Egger	21	0.068	0.214	1.07 (0.70, 1.63)	7.53E-01
		Weighted median	21	0.001	0.105	1.00 (0.81, 1.23)	9.95E-01
		Weighted mode	21	0.001	0.106	1.00 (0.81, 1.23)	9.91E-01
	Bronchiectasis	Inverse variance weighted	21	0.048	0.074	1.05 (0.91, 1.21)	5.14E-01
		MR Egger	21	0.262	0.188	1.30 (0.90, 1.88)	1.81E-01
		Weighted median	21	0.070	0.104	1.07 (0.87, 1.31)	5.02E-01
		Weighted mode	21	0.088	0.108	1.09 (0.88, 1.35)	4.28E-01
	Influenza	Inverse variance weighted	21	-0.109	0.038	0.90 (0.83, 0.97)	3.88E-03
		MR Egger	21	-0.192	0.103	0.83 (0.67, 1.01)	7.84E-02
		Weighted median	21	-0.148	0.054	0.86 (0.78, 0.96)	5.80E-03
		Weighted mode	21	-0.142	0.056	0.87 (0.78, 0.97)	2.04E-02
	Pneumonia	Inverse variance weighted[a]	21	-0.030	0.022	0.97 (0.93, 1.01)	1.82E-01
		MR Egger	21	0.010	0.058	1.01 (0.90, 1.13)	8.70E-01
		Weighted median	21	-0.037	0.022	0.96 (0.92, 1.01)	9.92E-02
		Weighted mode	21	-0.033	0.023	0.97 (0.93, 1.01)	1.61E-01
Basophil count	Acute bronchitis	Inverse variance weighted	191	-0.036	0.039	0.96 (0.89, 1.04)	3.56E-01
		MR Egger	191	-0.039	0.078	0.96 (0.83, 1.12)	6.20E-01
		Weighted median	191	-0.090	0.067	0.91 (0.80, 1.04)	1.82E-01
		Weighted mode	191	-0.066	0.084	0.94 (0.79, 1.10)	4.37E-01
	Acute bronchiolitis	Inverse variance weighted	191	0.086	0.110	1.09 (0.88, 1.35)	4.33E-01
		MR Egger	191	0.041	0.217	1.04 (0.68, 1.60)	8.49E-01
		Weighted median	191	-0.093	0.182	0.91 (0.64, 1.30)	6.09E-01
		Weighted mode	191	-0.164	0.227	0.85 (0.54, 1.32)	4.72E-01
	Bronchiectasis	Inverse variance weighted	191	0.137	0.103	1.15 (0.94, 1.40)	1.81E-01
		MR Egger	191	0.338	0.204	1.40 (0.94, 2.09)	9.90E-02
		Weighted median	191	0.125	0.188	1.13 (0.78, 1.64)	5.06E-01
		Weighted mode	191	0.132	0.214	1.14 (0.75, 1.73)	5.37E-01
	Influenza	Inverse variance weighted	191	0.011	0.053	1.01 (0.91, 1.12)	8.31E-01
		MR Egger	191	-0.017	0.100	0.98 (0.81, 1.20)	8.68E-01
		Weighted median	191	-0.048	0.096	0.95 (0.79, 1.15)	6.17E-01
		Weighted mode	191	-0.002	0.110	1.00 (0.81, 1.24)	9.87E-01
	Pneumonia	Inverse variance weighted[a]	191	0.058	0.026	1.06 (1.01, 1.11)	2.23E-02
		MR Egger	191	0.053	0.047	1.05 (0.96, 1.16)	2.57E-01
		Weighted median	191	0.033	0.041	1.03 (0.95, 1.12)	4.15E-01
		Weighted mode	191	0.005	0.047	1.00 (0.92, 1.10)	9.23E-01
Eosinophil count	Acute bronchitis	Inverse variance weighted[a]	429	0.106	0.027	1.11 (1.06, 1.17)	7.19E-05
		MR Egger	429	0.079	0.052	1.08 (0.98, 1.20)	1.28E-01
		Weighted median	429	0.117	0.042	1.12 (1.04, 1.22)	5.18E-03

		Weighted mode	429	0.065	0.054	1.07 (0.96, 1.19)	2.26E-01
	Acute bronchiolitis	Inverse variance weighted	429	0.186	0.069	1.20 (1.05, 1.38)	6.77E-03
		MR Egger	429	0.161	0.136	1.17 (0.90, 1.53)	2.39E-01
		Weighted median	429	0.176	0.116	1.19 (0.95, 1.50)	1.31E-01
		Weighted mode	429	0.214	0.156	1.24 (0.91, 1.68)	1.70E-01
	Bronchiectasis	Inverse variance weighted[a]	429	0.170	0.070	1.19 (1.03, 1.36)	1.48E-02
		MR Egger	429	0.163	0.135	1.18 (0.90, 1.53)	2.29E-01
		Weighted median	429	0.274	0.109	1.31 (1.06, 1.63)	1.23E-02
		Weighted mode	429	0.278	0.131	1.32 (1.02, 1.71)	3.48E-02
	Influenza	Inverse variance weighted	429	0.037	0.033	1.04 (0.97, 1.11)	2.58E-01
		MR Egger	429	0.072	0.066	1.07 (0.94, 1.22)	2.80E-01
		Weighted median	429	0.038	0.058	1.04 (0.93, 1.16)	5.13E-01
		Weighted mode	429	0.074	0.074	1.08 (0.93, 1.24)	3.15E-01
	Pneumonia	Inverse variance weighted[a]	429	0.061	0.017	1.06 (1.03, 1.10)	2.22E-04
		MR Egger	429	0.106	0.032	1.11 (1.04, 1.18)	9.91E-04
		Weighted median	429	0.064	0.023	1.07 (1.02, 1.11)	5.10E-03
		Weighted mode	429	0.088	0.036	1.09 (1.02, 1.17)	1.39E-02
Lymphocyte count	Acute bronchitis	Inverse variance weighted[a]	476	0.071	0.027	1.07 (1.02, 1.13)	9.33E-03
		MR Egger	476	0.064	0.056	1.07 (0.96, 1.19)	2.50E-01
		Weighted median	476	0.104	0.046	1.11 (1.01, 1.21)	2.37E-02
		Weighted mode	476	0.126	0.069	1.13 (0.99, 1.30)	6.96E-02
	Acute bronchiolitis	Inverse variance weighted[a]	476	-0.052	0.082	0.95 (0.81, 1.11)	5.21E-01
		MR Egger	476	-0.085	0.167	0.92 (0.66, 1.27)	6.13E-01
		Weighted median	476	-0.133	0.136	0.88 (0.67, 1.14)	3.26E-01
		Weighted mode	476	-0.062	0.194	0.94 (0.64, 1.37)	7.48E-01
	Bronchiectasis	Inverse variance weighted	476	0.041	0.067	1.04 (0.91, 1.19)	5.46E-01
		MR Egger	476	-0.016	0.138	0.98 (0.75, 1.29)	9.09E-01
		Weighted median	476	0.134	0.116	1.14 (0.91, 1.44)	2.48E-01
		Weighted mode	476	0.199	0.162	1.22 (0.89, 1.68)	2.20E-01
	Influenza	Inverse variance weighted	476	0.047	0.034	1.05 (0.98, 1.12)	1.69E-01
		MR Egger	476	0.069	0.072	1.07 (0.93, 1.23)	3.42E-01
		Weighted median	476	0.068	0.059	1.07 (0.95, 1.20)	2.46E-01
		Weighted mode	476	0.088	0.077	1.09 (0.94, 1.27)	2.50E-01
	Pneumonia	Inverse variance weighted[a]	476	0.058	0.017	1.06 (1.02, 1.10)	7.25E-04
		MR Egger	476	0.103	0.035	1.11 (1.03, 1.19)	3.48E-03
		Weighted median	476	0.045	0.026	1.05 (0.99, 1.10)	8.61E-02
		Weighted mode	476	0.077	0.036	1.08 (1.01, 1.16)	3.13E-02
Monocyte count	Acute bronchitis	Inverse variance weighted[a]	479	-0.002	0.022	1.00 (0.96, 1.04)	9.46E-01
		MR Egger	479	-0.004	0.036	1.00 (0.93, 1.07)	9.06E-01
		Weighted median	479	0.006	0.039	1.01 (0.93, 1.08)	8.82E-01
		Weighted mode	479	0.040	0.037	1.04 (0.97, 1.12)	2.78E-01

	Acute bronchiolitis	Inverse variance weighted	479	0.024	0.058	1.02 (0.91, 1.15)	6.81E-01
		MR Egger	479	-0.055	0.095	0.95 (0.79, 1.14)	5.65E-01
		Weighted median	479	0.098	0.100	1.10 (0.91, 1.34)	3.27E-01
		Weighted mode	479	0.056	0.107	1.06 (0.86, 1.30)	6.02E-01
	Bronchiectasis	Inverse variance weighted	479	0.030	0.055	1.03 (0.93, 1.15)	5.76E-01
		MR Egger	479	0.023	0.092	1.02 (0.85, 1.22)	8.06E-01
		Weighted median	479	0.074	0.098	1.08 (0.89, 1.31)	4.51E-01
		Weighted mode	479	0.145	0.111	1.16 (0.93, 1.44)	1.92E-01
	Influenza	Inverse variance weighted	479	0.029	0.028	1.03 (0.97, 1.09)	3.05E-01
		MR Egger	479	-0.019	0.046	0.98 (0.90, 1.07)	6.85E-01
		Weighted median	479	-0.010	0.050	0.99 (0.90, 1.09)	8.45E-01
		Weighted mode	479	-0.029	0.048	0.97 (0.88, 1.07)	5.50E-01
	Pneumonia	Inverse variance weighted[a]	479	0.035	0.013	1.04 (1.01, 1.06)	7.72E-03
		MR Egger	479	0.044	0.021	1.05 (1.00, 1.09)	3.74E-02
		Weighted median	479	0.035	0.020	1.04 (0.99, 1.08)	8.85E-02
		Weighted mode	479	0.055	0.021	1.06 (1.01, 1.10)	9.94E-03
Neutrophil count	Acute bronchitis	Inverse variance weighted[a]	400	0.035	0.032	1.04 (0.97, 1.10)	2.76E-01
		MR Egger	400	0.119	0.066	1.13 (0.99, 1.28)	7.47E-02
		Weighted median	400	0.022	0.051	1.02 (0.93, 1.13)	6.65E-01
		Weighted mode	400	0.014	0.073	1.01 (0.88, 1.17)	8.53E-01
	Acute bronchiolitis	Inverse variance weighted	400	0.136	0.082	1.15 (0.98, 1.34)	9.62E-02
		MR Egger	400	0.484	0.177	1.62 (1.15, 2.30)	6.52E-03
		Weighted median	400	0.142	0.141	1.15 (0.87, 1.52)	3.16E-01
		Weighted mode	400	0.128	0.257	1.14 (0.69, 1.88)	6.19E-01
	Bronchiectasis	Inverse variance weighted	400	0.050	0.076	1.05 (0.91, 1.22)	5.14E-01
		MR Egger	400	0.232	0.163	1.26 (0.92, 1.73)	1.55E-01
		Weighted median	400	0.091	0.120	1.10 (0.87, 1.38)	4.46E-01
		Weighted mode	400	0.137	0.171	1.15 (0.82, 1.61)	4.24E-01
	Influenza	Inverse variance weighted	400	0.059	0.039	1.06 (0.98, 1.14)	1.33E-01
		MR Egger	400	0.143	0.084	1.15 (0.98, 1.36)	8.86E-02
		Weighted median	400	0.054	0.064	1.05 (0.93, 1.20)	4.06E-01
		Weighted mode	400	0.081	0.104	1.08 (0.89, 1.33)	4.35E-01
	Pneumonia	Inverse variance weighted[a]	400	0.042	0.020	1.04 (1.00, 1.08)	3.57E-02
		MR Egger	400	0.059	0.042	1.06 (0.98, 1.15)	1.55E-01
		Weighted median	400	0.049	0.028	1.05 (0.99, 1.11)	7.77E-02
		Weighted mode	400	0.055	0.049	1.06 (0.96, 1.16)	2.66E-01
White blood cell count	Acute bronchitis	Inverse variance weighted[a]	466	0.063	0.030	1.07 (1.01, 1.13)	3.22E-02
		MR Egger	466	0.094	0.062	1.10 (0.97, 1.24)	1.28E-01
		Weighted median	466	0.034	0.047	1.03 (0.94, 1.13)	4.79E-01
		Weighted mode	466	0.044	0.069	1.05 (0.91, 1.20)	5.19E-01
	Acute bronchiolitis	Inverse variance weighted	466	0.122	0.075	1.13 (0.98, 1.31)	1.02E-01

	MR Egger	466	0.337	0.157	1.40 (1.03, 1.90)	3.23E-02
	Weighted median	466	0.087	0.132	1.09 (0.84, 1.41)	5.08E-01
	Weighted mode	466	0.043	0.268	1.04 (0.62, 1.77)	8.71E-01
Bronchiectasis	Inverse variance weighted	466	0.068	0.070	1.07 (0.93, 1.23)	3.32E-01
	MR Egger	466	0.115	0.148	1.12 (0.84, 1.50)	4.40E-01
	Weighted median	466	0.147	0.122	1.16 (0.91, 1.47)	2.28E-01
	Weighted mode	466	0.188	0.153	1.21 (0.89, 1.63)	2.21E-01
Influenza	Inverse variance weighted[a]	466	0.067	0.038	1.07 (0.99, 1.15)	8.13E-02
	MR Egger	466	0.090	0.080	1.09 (0.94, 1.28)	2.59E-01
	Weighted median	466	0.054	0.063	1.06 (0.93, 1.19)	3.87E-01
	Weighted mode	466	0.065	0.096	1.07 (0.88, 1.29)	4.97E-01
Pneumonia	Inverse variance weighted[a]	466	0.063	0.018	1.07 (1.03, 1.10)	6.02E-04
	MR Egger	466	0.067	0.038	1.07 (0.99, 1.15)	8.31E-02
	Weighted median	466	0.046	0.028	1.05 (0.99, 1.11)	1.01E-01
	Weighted mode	466	0.046	0.049	1.05 (0.95, 1.15)	3.44E-01

$\hat{\beta}$ represents the change in the log-odds of a type of LRTIs associated with per standard deviation increase in a potential mediator (except for CRP whose unit was one-unit natural-log-transformed CRP).

[a] denotes the use of a random-effects inverse variance weighted model.

Abbreviations: SNPs, single nucleotide polymorphisms; SE, standard error; OR, odds ratio; CI, confidence interval; FEV1, forced expiratory volume in the first second; FVC, forced vital capacity; CRP, C-reactive protein; GlycA, glycoprotein acetyls; IL-1Ra, interleukin-1-receptor antagonist; IL-6, interleukin-6; IL-8, interleukin-8; IL-18, interleukin-18; IL-27, interleukin-27; LRTIs, lower respiratory tract infections.

Table S9. Results of sensitivity analyses for the effects of potential mediators on lower respiratory tract infections.

Potential mediators	Lower respiratory tract infections	Pleiotropy			Heterogeneity			MR-PRESSO					
		Egger intercept	SE	*P*	*Q*	*dfs*	*P*	$\hat{\beta}$	SE	*P*	$\hat{\beta}$ (Outlier SNPs Removed)	SE (Outlier SNPs Removed)	*P* (Outlier SNPs Removed)
FEV1	Acute bronchitis	-2.175E-03	0.003	0.468	308.754	252	8.45E-03	-0.220	0.045	1.87E-06	-0.209	0.044	3.66E-06
	Acute bronchiolitis	-1.441E-03	0.008	0.856	276.993	252	1.34E-01	-0.347	0.116	2.96E-03	NA	NA	NA
	Bronchiectasis	-7.386E-03	0.007	0.320	278.164	252	1.24E-01	0.118	0.110	2.82E-01	NA	NA	NA
	Influenza	-1.043E-03	0.004	0.778	262.920	252	3.05E-01	-0.013	0.054	8.03E-01	NA	NA	NA
	Pneumonia	-3.552E-03	0.002	0.058	388.279	252	7.31E-08	-0.068	0.027	1.33E-02	-0.062	0.027	2.22E-02
FVC	Acute bronchitis	1.299E-03	0.003	0.694	277.322	228	1.42E-02	-0.145	0.048	2.54E-03	-0.131	0.047	5.19E-03
	Acute bronchiolitis	-7.232E-03	0.008	0.388	228.658	228	4.75E-01	-0.178	0.121	1.43E-01	NA	NA	NA
	Bronchiectasis	-7.984E-03	0.008	0.313	234.742	228	3.65E-01	-0.131	0.115	2.55E-01	NA	NA	NA
	Influenza	2.520E-03	0.004	0.547	249.372	228	1.58E-01	-0.087	0.060	1.49E-01	NA	NA	NA
	Pneumonia	1.960E-03	0.002	0.362	377.447	228	1.77E-09	-0.070	0.031	2.57E-02	-0.060	0.030	4.27E-02
CRP	Acute bronchitis	1.335E-03	0.003	0.638	66.113	53	1.07E-01	0.134	0.032	1.06E-04	NA	NA	NA
	Acute bronchiolitis	9.192E-03	0.007	0.196	51.596	53	5.29E-01	0.031	0.078	6.88E-01	NA	NA	NA
	Bronchiectasis	-3.463E-03	0.007	0.639	65.439	53	1.17E-01	0.053	0.083	5.26E-01	NA	NA	NA
	Influenza	1.096E-02	0.004	0.012	92.292	53	6.70E-04	0.182	0.050	6.47E-04	0.171	0.047	6.14E-04
	Pneumonia	2.589E-03	0.003	0.312	173.115	53	1.15E-14	0.157	0.029	1.86E-06	0.188	0.035	2.45E-06
GlycA	Acute bronchitis	7.496E-04	0.004	0.838	50.870	52	5.18E-01	0.035	0.038	3.58E-01	NA	NA	NA
	Acute bronchiolitis	1.082E-02	0.010	0.296	45.513	52	7.25E-01	-0.010	0.100	9.20E-01	NA	NA	NA
	Bronchiectasis	3.311E-03	0.012	0.790	88.628	52	1.16E-03	-0.144	0.130	2.71E-01	0.067	0.102	5.11E-01
	Influenza	8.568E-03	0.005	0.104	60.328	52	2.00E-01	0.010	0.055	8.59E-01	NA	NA	NA
	Pneumonia	5.815E-03	0.002	0.022	81.974	52	5.03E-03	0.045	0.028	1.15E-01	0.063	0.028	2.84E-02
IL-1Ra	Acute bronchitis	4.234E-03	0.006	0.521	21.736	22	4.76E-01	0.048	0.028	1.01E-01	NA	NA	NA
	Acute bronchiolitis	2.279E-02	0.018	0.221	20.285	22	5.65E-01	-0.012	0.076	8.76E-01	NA	NA	NA
	Bronchiectasis	3.069E-02	0.017	0.082	24.264	22	3.34E-01	0.122	0.078	1.30E-01	NA	NA	NA
	Influenza	1.175E-02	0.009	0.188	19.579	22	6.09E-01	0.017	0.036	6.34E-01	NA	NA	NA
	Pneumonia	1.771E-03	0.005	0.729	41.290	22	7.61E-03	-0.013	0.022	5.50E-01	0.010	0.023	6.68E-01
IL-6	Acute bronchitis	-3.741E-02	0.010	0.005	31.922	11	7.85E-04	0.059	0.070	4.16E-01	0.032	0.060	6.03E-01
	Acute bronchiolitis	-3.933E-02	0.029	0.210	16.934	11	1.10E-01	0.064	0.142	6.59E-01	NA	NA	NA
	Bronchiectasis	-3.436E-02	0.024	0.179	13.066	11	2.89E-01	-0.130	0.116	2.88E-01	NA	NA	NA
	Influenza	5.277E-03	0.012	0.665	10.426	11	4.93E-01	-0.019	0.053	7.25E-01	NA	NA	NA
	Pneumonia	-1.838E-03	0.005	0.715	6.821	11	8.13E-01	0.076	0.018	1.40E-03	NA	NA	NA
IL-8	Acute bronchitis	-7.934E-03	0.015	0.597	23.705	13	3.40E-02	-0.007	0.074	9.23E-01	NA	NA	NA
	Acute bronchiolitis	2.433E-03	0.036	0.947	17.644	13	1.72E-01	0.132	0.178	4.73E-01	NA	NA	NA
	Bronchiectasis	1.419E-02	0.034	0.679	17.998	13	1.58E-01	-0.191	0.169	2.79E-01	NA	NA	NA
	Influenza	-1.939E-02	0.014	0.195	10.608	13	6.44E-01	-0.002	0.066	9.82E-01	NA	NA	NA
	Pneumonia	-1.328E-02	0.007	0.087	22.629	13	4.64E-02	-0.023	0.040	5.86E-01	NA	NA	NA

IL-18	Acute bronchitis	-1.048E-02	0.006	0.111	24.683	23	3.67E-01	0.039	0.027	1.59E-01	NA	NA	NA
	Acute bronchiolitis	-2.037E-03	0.020	0.920	27.895	23	2.20E-01	0.026	0.079	7.42E-01	NA	NA	NA
	Bronchiectasis	-2.952E-02	0.018	0.110	28.458	23	1.99E-01	0.041	0.074	5.92E-01	NA	NA	NA
	Influenza	-1.182E-02	0.010	0.247	32.273	23	9.46E-02	-0.061	0.041	1.49E-01	NA	NA	NA
	Pneumonia	1.660E-03	0.004	0.696	31.179	23	1.18E-01	-0.010	0.017	5.41E-01	NA	NA	NA
IL-27	Acute bronchitis	1.635E-04	0.009	0.986	26.992	20	1.35E-01	-0.006	0.033	8.65E-01	NA	NA	NA
	Acute bronchiolitis	-8.734E-03	0.023	0.712	21.464	20	3.70E-01	-0.005	0.082	9.47E-01	NA	NA	NA
	Bronchiectasis	-2.529E-02	0.021	0.233	19.747	20	4.74E-01	0.048	0.073	5.19E-01	NA	NA	NA
	Influenza	9.823E-03	0.011	0.393	22.622	20	3.08E-01	-0.109	0.040	1.33E-02	NA	NA	NA
	Pneumonia	-4.662E-03	0.006	0.468	40.137	20	4.80E-03	-0.030	0.022	1.97E-01	-0.041	0.019	4.16E-02
Basophil count	Acute bronchitis	6.924E-05	0.002	0.972	219.055	190	7.29E-02	-0.038	0.041	3.54E-01	NA	NA	NA
	Acute bronchiolitis	1.339E-03	0.005	0.806	218.315	190	7.78E-02	0.088	0.116	4.52E-01	NA	NA	NA
	Bronchiectasis	-5.997E-03	0.005	0.242	222.710	190	5.22E-02	0.141	0.110	2.03E-01	NA	NA	NA
	Influenza	8.309E-04	0.002	0.739	200.766	190	2.82E-01	-0.012	0.054	8.25E-01	NA	NA	NA
	Pneumonia	1.465E-04	0.001	0.901	254.902	190	1.16E-03	0.047	0.026	7.39E-02	0.053	0.025	3.86E-02
Eosinophil count	Acute bronchitis	8.813E-04	0.001	0.538	504.086	428	6.51E-03	0.116	0.026	1.38E-05	0.119	0.026	6.77E-06
	Acute bronchiolitis	8.355E-04	0.004	0.824	446.993	428	2.54E-01	0.198	0.069	4.53E-03	NA	NA	NA
	Bronchiectasis	2.435E-04	0.004	0.948	505.884	428	5.57E-03	0.173	0.068	1.19E-02	NA	NA	NA
	Influenza	-1.107E-03	0.002	0.545	461.801	428	1.25E-01	0.043	0.034	2.07E-01	NA	NA	NA
	Pneumonia	-1.443E-03	0.001	0.102	614.779	428	7.58E-09	0.066	0.016	6.16E-05	0.061	0.016	1.45E-04
Lymphocyte count	Acute bronchitis	1.810E-04	0.001	0.893	526.387	475	5.13E-02	0.062	0.027	2.10E-02	NA	NA	NA
	Acute bronchiolitis	8.976E-04	0.004	0.825	609.170	475	2.90E-05	-0.036	0.080	6.51E-01	-0.060	0.077	4.37E-01
	Bronchiectasis	1.566E-03	0.003	0.640	476.302	475	4.75E-01	0.030	0.067	6.50E-01	NA	NA	NA
	Influenza	-5.954E-04	0.002	0.734	499.140	475	2.14E-01	0.042	0.035	2.29E-01	NA	NA	NA
	Pneumonia	-1.245E-03	0.001	0.142	670.877	475	7.28E-09	0.058	0.017	6.57E-04	0.063	0.017	1.89E-04
Monocyte count	Acute bronchitis	1.056E-04	0.001	0.923	543.275	478	2.05E-02	0.001	0.022	9.78E-01	0.014	0.022	5.29E-01
	Acute bronchiolitis	3.008E-03	0.003	0.294	456.768	478	7.50E-01	0.028	0.057	6.19E-01	NA	NA	NA
	Bronchiectasis	3.007E-04	0.003	0.913	506.433	478	1.78E-01	0.028	0.056	6.22E-01	NA	NA	NA
	Influenza	1.812E-03	0.001	0.196	497.676	478	2.58E-01	0.031	0.028	2.76E-01	NA	NA	NA
	Pneumonia	-3.675E-04	0.001	0.566	596.953	478	1.66E-04	0.032	0.013	1.16E-02	NA	NA	NA
Neutrophil count	Acute bronchitis	-2.187E-03	0.002	0.151	483.655	399	2.32E-03	0.034	0.031	2.72E-01	0.034	0.030	2.59E-01
	Acute bronchiolitis	-9.101E-03	0.004	0.025	441.853	399	6.82E-02	0.131	0.082	1.09E-01	NA	NA	NA
	Bronchiectasis	-4.753E-03	0.004	0.202	423.744	399	1.89E-01	-0.012	0.076	8.76E-01	NA	NA	NA
	Influenza	-2.203E-03	0.002	0.251	427.289	399	1.58E-01	0.062	0.039	1.15E-01	NA	NA	NA
	Pneumonia	-4.495E-04	0.001	0.637	601.625	399	2.07E-10	0.045	0.019	2.07E-02	0.053	0.019	5.54E-03
White blood cell count	Acute bronchitis	-8.083E-04	0.001	0.573	571.830	465	5.06E-04	0.067	0.028	1.90E-02	0.067	0.028	1.65E-02
	Acute bronchiolitis	-5.686E-03	0.004	0.120	475.013	465	3.64E-01	0.145	0.072	4.61E-02	NA	NA	NA
	Bronchiectasis	-1.247E-03	0.003	0.718	484.195	465	2.60E-01	0.048	0.070	4.90E-01	NA	NA	NA
	Influenza	-6.212E-04	0.002	0.738	532.464	465	1.64E-02	0.072	0.037	5.14E-02	NA	NA	NA
	Pneumonia	-9.051E-05	0.001	0.919	709.197	465	1.91E-12	0.066	0.018	2.25E-04	0.075	0.017	2.05E-05

$\hat{\boldsymbol{\beta}}$ represents the change in the log-odds of a type of LRTIs associated with per standard deviation increase in a potential mediator (except for CRP whose unit was one-unit natural-log-transformed CRP).

Abbreviations: MR-PRESSO, MR-pleiotropy residual sum and outlier method; Egger intercept, intercept term of MR Egger regression; SE, standard error; dfs, degrees of freedom; SNPs, single nucleotide polymorphisms; FEV1, forced expiratory volume in the first second; FVC, forced vital capacity; CRP, C-reactive protein; GlycA, glycoprotein acetyls; IL-1Ra, interleukin-1-receptor antagonist; IL-6, interleukin-6; IL-8, interleukin-8; IL-18, interleukin-18; IL-27, interleukin-27.

Table S10. Results of mediation analyses.

Exposures	Potential mediators	Outcomes	$\hat{\beta}$ (95% CI)	$\hat{\beta}_1$ (95% CI)	$\hat{\beta}_2$ (95% CI)	Mediation proportion (%)[a] (95% CI)
BMI	FEV1	Acute bronchitis	0.259 (0.182, 0.337)	-0.099 (-0.144, -0.055)	-0.220 (-0.308, -0.132)	8.41 (2.77, 14.06)
BMI	FVC	Acute bronchitis	0.259 (0.182, 0.337)	-0.157 (-0.206, -0.108)	-0.144 (-0.238, -0.049)	8.72 (1.86, 15.58)
BMI	CRP	Acute bronchitis	0.259 (0.182, 0.337)	0.371 (0.343, 0.400)	0.130 (0.073, 0.187)	18.66 (8.70, 28.62)
BMI	FEV1	Pneumonia	0.179 (0.134, 0.225)	-0.099 (-0.144, -0.055)	-0.073 (-0.128, -0.018)	4.04 (0.34, 7.74)
BMI	FVC	Pneumonia	0.179 (0.134, 0.225)	-0.157 (-0.206, -0.108)	-0.064 (-0.126, -0.002)	5.63 (-0.24, 11.50)
BMI	CRP	Pneumonia	0.179 (0.134, 0.225)	0.371 (0.343, 0.400)	0.157 (0.100, 0.215)	32.59 (17.90, 47.27)
BMI	IL-6	Pneumonia	0.179 (0.134, 0.225)	0.187 (0.105, 0.269)	0.076 (0.031, 0.121)	7.96 (1.79, 14.14)
BMI	Monocyte count	Pneumonia	0.179 (0.134, 0.225)	-0.064 (-0.107, -0.021)	0.035 (0.009, 0.060)	1.23 (-0.04, 2.50)
WC	FEV1	Acute bronchitis	0.234 (0.137, 0.332)	-0.134 (-0.202, -0.067)	-0.220 (-0.308, -0.132)	12.61 (2.94, 22.29)
WC	FVC	Acute bronchitis	0.234 (0.137, 0.332)	-0.199 (-0.259, -0.139)	-0.144 (-0.238, -0.049)	12.19 (2.00, 22.39)
WC	CRP	Acute bronchitis	0.234 (0.137, 0.332)	0.359 (0.298, 0.421)	0.130 (0.073, 0.187)	19.96 (7.44, 32.48)
WC	FEV1	Pneumonia	0.210 (0.155, 0.265)	-0.134 (-0.202, -0.067)	-0.073 (-0.128, -0.018)	4.67 (0.25, 9.09)
WC	FVC	Pneumonia	0.210 (0.155, 0.265)	-0.199 (-0.259, -0.139)	-0.064 (-0.126, -0.002)	6.08 (-0.25, 12.41)
WC	CRP	Pneumonia	0.210 (0.155, 0.265)	0.359 (0.298, 0.421)	0.157 (0.100, 0.215)	26.90 (13.98, 39.83)
WC	IL-6	Pneumonia	0.210 (0.155, 0.265)	0.282 (0.182, 0.381)	0.076 (0.031, 0.121)	10.23 (2.72, 17.73)

$\hat{\beta}$ means the change in the log-odds of the type of LRTIs associated with per standard deviation (SD) increase in the obesity-related trait; $\hat{\beta}_1$ represents the change in a potential mediator (in standard deviation unit, except for CRP, whose unit was one-unit natural-log-transformed CRP) associated with per SD increase in an obesity-related trait; $\hat{\beta}_2$ represents the change in the log-odds of the type of LRTIs associated with per SD increase in a potential mediator (except for CRP whose unit was one-unit natural-log-transformed CRP).

[a] Mediation proportion was calculated as $(\hat{\beta}_1 \times \hat{\beta}_2) / \hat{\beta} \times 100\%$.

Abbreviations: CI, confidence interval; BMI, body mass index; WC, waist circumference; WHR,waist-to-hip ratio; FEV1, forced expiratory volume in the first second; FVC, forced vital capacity; CRP, C-reactive protein; IL-6, interleukin-6.
